# Assessment of Attainable Distances in Proteins by Through‐Space ^19^F–^19^F Couplings

**DOI:** 10.1002/chem.71456

**Published:** 2026-07-24

**Authors:** Iker Hernández, In‐Ja L. Byeon, Christina M. Monnie, Vadim A. Soloshonok, Aitor Landa, Mikel Oiarbide, Angela M. Gronenborn

**Affiliations:** ^1^ Department of Organic Chemistry I, Faculty of Chemistry University of the Basque Country (EHU) Donostia/San Sebastián Spain; ^2^ Department of Structural Biology University of Pittsburgh School of Medicine Pittsburgh Pennsylvania USA

**Keywords:** fluorine, ^19^F–^19^F distance, ^19^F–^19^F NOESY, NMR spectroscopy, nuclear Overhauser effect

## Abstract

^19^F NMR is becoming an increasingly powerful method for studying the conformation and ligand binding of proteins in the solid state and in solution. In liquids NMR, surprisingly, reports on through‐space ^19^F–^19^F coupling remain scarce. Here, we present the first ^19^F–^19^F NOESY study designed to calibrate inter‐fluorine distances in proteins. Three difluorinated tryptophan analogues (4,5‐F_2_‐Trp, 4,6‐F_2_‐Trp, 5,6‐F_2_‐Trp) were synthesized and site‐specifically incorporated into human cyclophilin A (CypA). Series of ^19^F–^19^F NOESY build‐up experiments were recorded for each labeled protein, and the extracted cross‐relaxation rates were compared to fixed reference distances. While small distances in the 2.5–3.0 Å range were easily accessible, the upper limit at 14.1 T is ∼5 Å. These results provide evidence that short distances can be extracted from ^19^F–^19^F NOESY spectra, expanding the toolbox for studying protein‐protein or protein‐ligand interactions by NMR.

## Graphical abstract

De novo synthesized difluorinated tryptophan analogues are site‐specifically incorporated into Cyclophilin A (CypA). ^19^F–^19^F NOESY build‐up experiments are recorded for each labeled protein, and the distances derived from cross‐relaxation rates are compared to reference distances. This work demonstrates that short interfluorine distances (≤5 Å) can be measured in proteins using ^19^F–^19^F NOESY spectra.

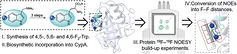

## 1 Introduction

Nuclear magnetic resonance (NMR) spectroscopy has become one of the most powerful techniques for the elucidation of the three‐dimensional (3D) structure, dynamics and interactions of biomolecules in solution. Among the different solution NMR observables, the ^1^H–^1^H Nuclear Overhauser Effect (NOE) [1, 2, 3] provides invaluable information on protein 3D structure and allows structure determination of biological macromolecules [4, 5, 6]. Indeed, hundreds of interproton distances up to 5 Å are routinely used in structure determination programs [7, 8, 9], with distances up to 8 Å becoming accessible in deuterated proteins due to reduction of dipolar proton‐proton (T_1_) relaxation [10]. The exploitation of NOEs involving other NMR‐active nuclei, such as ^19^F, have remained underexplored, although its high gyromagnetic ratio (≈94% that of ^1^H) and exceptional chemical‐shift sensitivity to even subtle variations in the local environment are well known [11]. Moreover, because fluorine is virtually absent from biological systems, ^19^F NMR spectra are entirely free of background signals. These features have driven a growing interest in applying ^19^F NMR to investigate protein structure, dynamics and interactions [12, 13].

A very early report by Kimber et al. demonstrated the presence of F–F through‐space coupling, specifically between two ^19^F atoms of a 6‐F‐Trp labeled *L. casei* dihydrofolate reductase in a ternary complex with NADPH and MTX [14]. More recently, the Otting group reported F–F couplings between CF_3_‐containing aromatic amino acids in a similarly sized protein. Interestingly, the NOE cross peaks were very small, while the through‐bond couplings were readily observed in a ^19^F–^19^F TOCSY experiment [15].

For calibrating H–H NOEs in proteins, the fixed distance of 1.8 Å between geminal β‐protons is potentially available. Unfortunately, spin diffusion makes this fraught with difficulties in non‐deuterated proteins and therefore, H–H NOEs are used primarily in a semi‐quantitative way in structural protein NMR, with the size of the NOE cross peaks converted into upper distance constraints. More recently, a quantitative approach using exact NOEs (eNOEs) [16], extracted from NOE build‐up curves for calculating cross‐relaxation rates, has been re‐introduced [17].

Here, we embarked on exploring the potential of ^19^F–^19^F eNOEs for determining short, accurate internuclear distances in proteins. We exploited a readily available methodology for incorporating fluorinated tryptophan residues (Trp) into proteins [18], since, for numerous proteins, the presence of fluorinated Trp introduces minimal perturbation of overall structure, interactions, and motions [19, 20, 21]. To this end, three difluorinated tryptophan analogues, 4,5‐F_2_‐Trp, 4,6‐F_2_‐Trp, and 5,6‐F_2_‐Trp, were synthesized and incorporated into human cyclophilin A (CypA). CypA, a peptidyl prolyl isomerase that regulates protein folding and trafficking, plays pivotal roles related to numerous diseases [22]. Its structure, dynamics and ligand binding have been studied extensively by NMR over the past 30 years [23, 24, 25, 26]. CypA possesses a single Trp in its amino acid sequence, and we previously showed that CypA proteins containing 4‐, 5‐, 6‐, or 7‐F‐Trp exhibit structures identical to the nonfluorinated protein [19].

## 2 Results and Discussion

Gram‐scale chemical synthesis of the three difluorinated tryptophan analogues was carried out following our previously established protocol, starting from the corresponding difluorogramine and 2‐benzylthio‐1,5‐dihydro‐4*H*‐imidazolone as glycine equivalent in three synthetic steps (Figure 1a; further details are given in the Supporting Information) [27]. The labeled proteins were produced using a standard *E. coli* expression protocol, as detailed in the Supporting Information. Intriguingly, for 4,5‐F_2_‐Trp CypA, tenfold higher protein yields were consistently obtained, compared to the two other variants, a difference possibly caused by less efficient binding of 5,6‐ and 4,6‐F_2_‐Trp into the Trp binding pocket of the Trp‐tRNA‐synthetase. Inspection of the binding pocket around the Trp in the X‐ray structure of the complex (PDB ID: 00002ake) reveals that position six of the indole ring is in close contact with atoms of the Val144 side chain. Therefore, the slightly larger and strongly electronegative F atom at the six position may cause some unfavorable steric/electronic effect.

**FIGURE 1:**
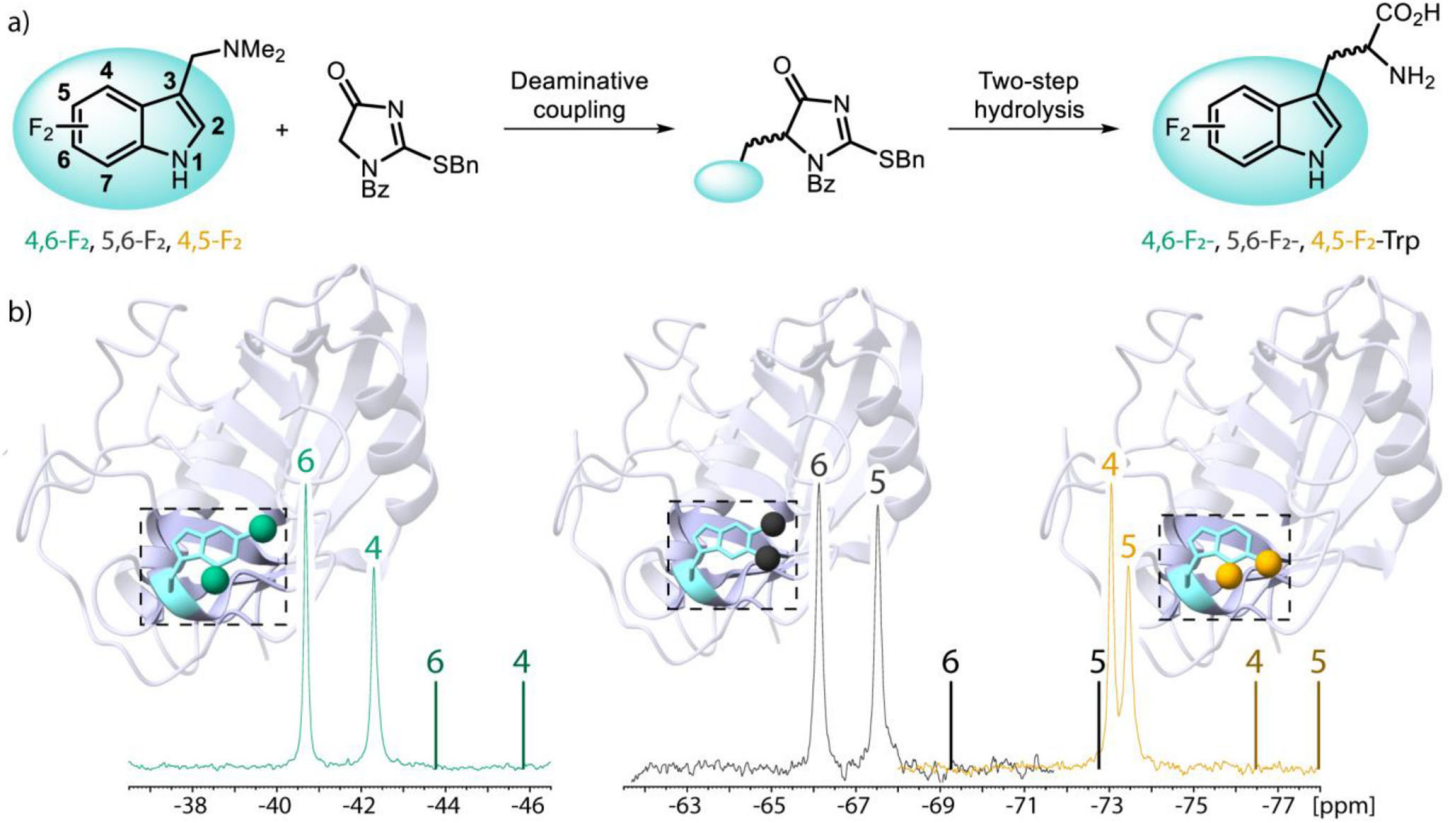
(a) Schematic depiction of the synthesis of 4,6‐, 5,6‐, and 4,5‐F_2_‐Trp. Overall yields (three steps): 65%, 54%, and 48%, respectively. (b) Superimposed ^19^F NMR spectra of 4,6‐F_2_‐Trp CypA (green), 5,6‐F_2_‐Trp CypA (gray), and 4,5‐F_2_‐Trp CypA (orange), with isotropic ^19^F chemical shifts of the free amino acids indicated by lines. The assignment of the individual ^19^F resonances was established from the spectra of the free F_2_‐Trp amino acids and subsequently transferred to the corresponding labeled protein spectra (see Supporting Information for details).

The ^19^F NMR spectrum of 4,6‐F_2_‐Trp CypA shows two fluorine resonances at −40.7 and −42.3 ppm (Figure 1b, green spectrum), whereas those of 5,6‐F_2_‐Trp CypA and 4,5‐F_2_‐Trp CypA exhibit peaks at −66.1 and −67.5 ppm (Figure 1b, gray spectrum), and −73.1 and −73.5 ppm (Figure 1b, orange spectrum), respectively. In all cases, the protein ^19^F resonances are shifted downfield compared to those of the corresponding free amino acids (represented by colored lines in Figure 1b). All protein signals appear as broad singlets, with linewidths on the order of 100 Hz, considerably larger than the typical ^1^H–^19^F and ^19^F–^19^F scalar coupling constants in an indole ring due to the strong contribution of CSA‐driven ^19^F relaxation. The resonance positions of both signals for free 5,6‐F_2_‐Trp and 4,5‐F_2_‐Trp as well as 5,6‐F_2_‐Trp CypA and 4,5‐F_2_‐Trp CypA in the spectra are ∼25−30 ppm upfield of the 4,6 resonances, consistent with the known ortho effect of fluorine on neighboring fluorine resonances in aromatic rings [28].

We decided to focus on di‐fluoro Trp amino acids since their defined geometry results in fixed fluorine–fluorine distances. Even if no X‐ray structures of the individual difluoro Trps are available in the Cambridge database, X‐ray structures of analogous molecules are available that can be used to extract the relevant distances between the fluorine atoms: 2.650 Å for 5,6‐F_2_‐Trp and 2.686 Å for 4,5‐F_2_‐Trp (Figures S8 and S9). Fortunately, during the course of this work, we obtained crystals of 4,6‐F_2_‐Trp, and the distance between the two fluorine atoms was measured as 4.702 Å (CCDC 2538127, Figure S10) [29].

A set of 2D ^19^F–^19^F NOESY spectra with increasing mixing times was recorded for each labeled protein. For 5,6‐F_2_‐Trp CypA, cross‐peak intensities increased progressively throughout the build‐up regime, reaching maximal magnetization transfer between 400 and 600 ms and displaying a well‐defined plateau thereafter (Figures 2 and S12). For 4,5‐F_2_‐Trp CypA, the two resonances in the ^19^F spectrum are close (Δ*δ* ∼0.4 ppm), making accurate signal intensity measurement more difficult. Irrespective, the build‐up of cross‐peak intensities could be followed, and intensities reached maximal values around 400 ms (Figures 2 and S17). For the 4,6‐ variant, longer mixing times were required to detect NOE cross peaks, and they plateaued only after 1 s (Figures 2 and S18).

**FIGURE 2:**
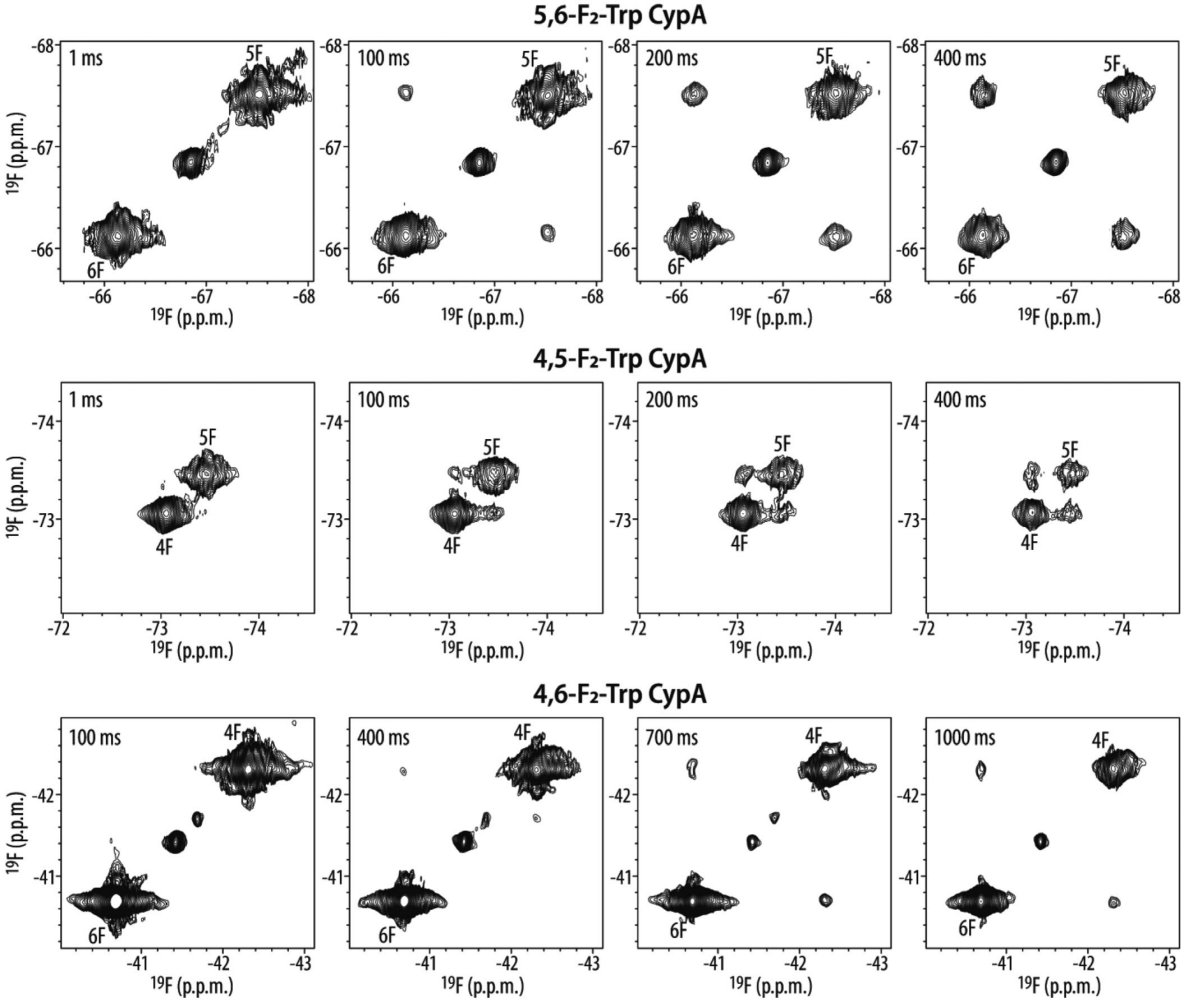
Representative ^19^F–^19^F NOESY spectra of 5,6‐, 4,5‐ and 4,6‐F_2_‐Trp CypA for selected mixing times. The protein concentrations were 660, 307, and 500 µM, respectively. All spectra were recorded at 25 °C, with a recycle delay of 2.5 or 2.6 s and without ^1^H decoupling. The full sets of ^19^F–^19^F NOESY spectra are provided in Supporting Information (Figures S12, S17, S18). For 5,6‐ and 4,6‐F_2_‐Trp CypA, minor sample degradation was observed over time, giving rise to additional signals at −66.8 ppm in the 5,6‐F_2_‐Trp CypA spectra and at −41.4 and −43.5 ppm in the 4,6‐F_2_‐Trp CypA spectra. These signals did not overlap with the protein resonances and therefore did not affect the NOE measurements. See the Supporting Information for further details.

Diagonal‐peak and cross‐peak intensities (peak heights) were extracted from the NOESY build‐up experiments. We performed diagonal normalization according to Macura et al. [30], also recently used by Orts et al. [31]. Diagonal‐normalized NOEs were plotted as a function of mixing time and fitted to a linear and a hyperbolic tangent equation (Figure 3).

**FIGURE 3:**
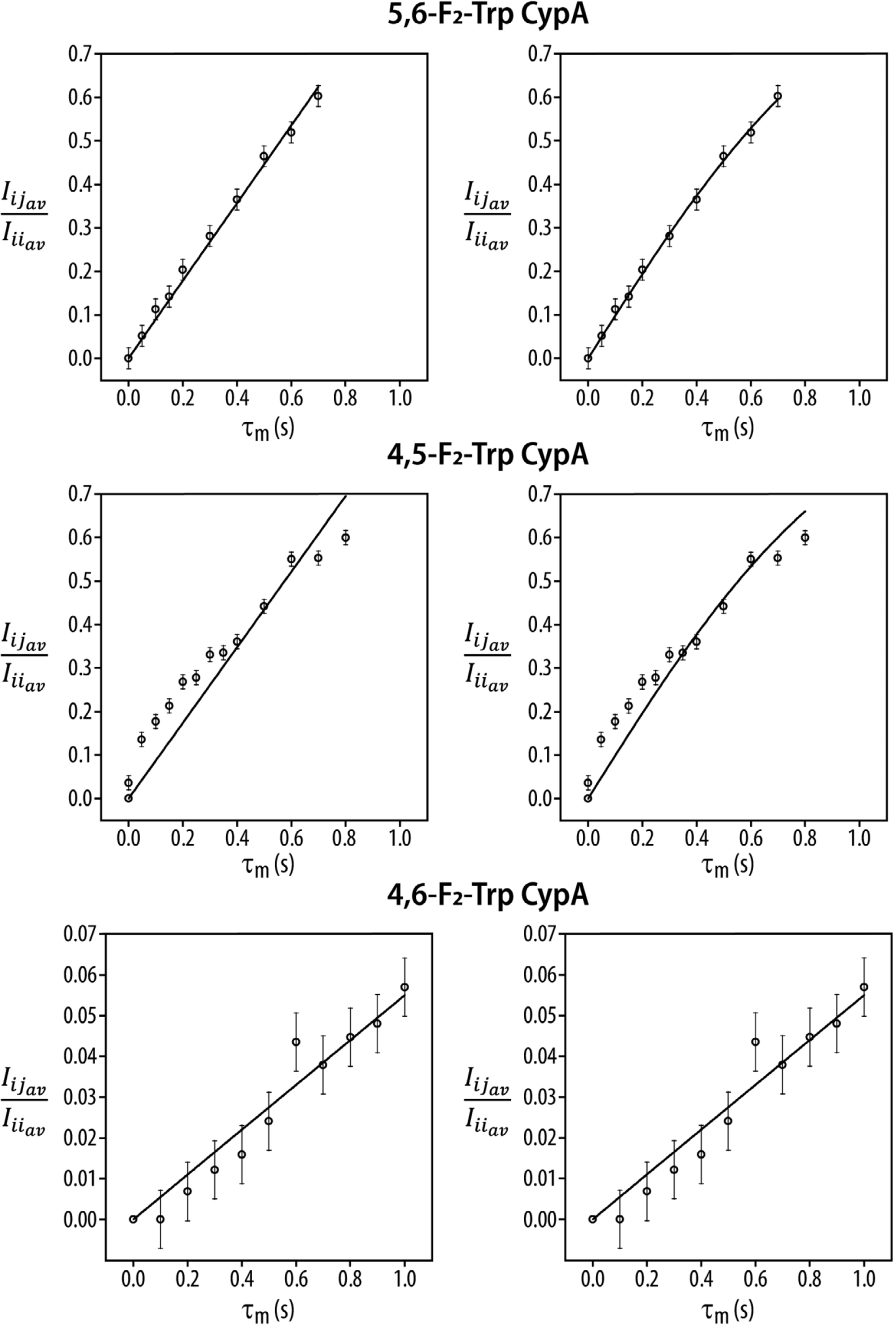
Diagonal‐normalized averaged cross‐peak intensities, plotted against mixing time. For each mixing time, the intensities of the two cross peaks were averaged and normalized to the corresponding averaged diagonal intensity $\left((I_{\mathrm{ij}}+I_{\mathrm{ji}})/2\right)/\left((I_{\mathrm{ii}}+I_{\mathrm{jj}})/2\right)$. The two panels for each protein correspond to alternative fits of the same experimental data. Equations used for fitting were $I_{\mathrm{NOE}}\left(\tau_{\mathrm{mix}}\right)=Bx$ in the left panels and $I_{\mathrm{NOE}}\left(\tau_{\mathrm{mix}}\right)=\left(e^{2x}-1\right)/\left(e^{2x}+1\right)$ in the right panels. Noise‐based error bars for each dataset were estimated from the average spectral noise intensity normalized by the average diagonal‐peak intensity at the longest mixing time.

For 5,6‐F_2_‐Trp CypA (Figure 3, upper panel), both fits are equally good. The fits for the 4,5‐F_2_‐Trp CypA data (Figure 3, middle panel) and the 4,6‐F_2_‐Trp CypA data (Figure 3, bottom panel) are associated with higher errors. In the case of 4,5‐F_2_‐Trp CypA, cross‐peak intensities could not be measured as accurately since the frequencies of the 4 and 5 fluorine resonances are too close, resulting in bleeding of intensity into the cross peaks. The data for 4,6‐F_2_‐Trp CypA (Figure 3, bottom panel) is inherently noisier, naturally reflecting the larger distance between these fluorine atoms on the indole ring with the resulting NOEs approaching the detection limit.

In the simplest approximation, cross‐relaxation rates can be extracted from the slopes of the build‐up curves. Details of the errors for the two fitting approaches are provided in Table 1. As can be appreciated, all errors are comparable, suggesting that linear extrapolation of the initial NOE build‐up curves suffices. In addition, it appears that averaging the cross‐peak intensities is advisable, and such approach is used in traditional ^1^H–^1^H NOESY spectra of proteins in bioNMR by symmetrization of the spectra.

**TABLE 1:** Summary of cross‐relaxation rates and associated F–F distances for 5,6‐F_2_‐Trp CypA, 4,5‐F_2_‐Trp CypA, and 4,6‐F_2_‐Trp CypA.

Protein	Normalization	Fitted to:	$r_{\mathrm{ij}}^{\mathrm{eff}}$	${\left(r_{\mathrm{ij}}^{\mathrm{eff}}\right)}_{\mathrm{avg}}$
A	$\frac{I_{\mathrm{ij}\;(\tau_{\mathrm{mix}})}}{I_{\mathrm{ii}\;(\tau_{\mathrm{mix}})}}$	$I_{\mathrm{NOE}}\left(\tau_{\mathrm{mix}}\right)=Bx$	$={\left(44.72\frac{8.2}{\sigma_{\mathrm{ij}}/s^{-1}}\right)}^{\frac{1}{6}}$	
**5,6**‐F_2_‐Trp CypA	$\sigma_{5\to 6}$= 0.951 ± 0.021		2.70	**2.73**
	$\sigma_{6\to 5}$= 0.840 ± 0.020		2.75	
**4,5**‐F_2_‐Trp CypA	$\sigma_{4\to 5}$= 0.633 ± 0.041		2.89	**2.70**
	$\sigma_{5\to 4}$= 1.424 ± 0.045		2.52	
**4,6**‐F_2_‐Trp CypA	$\sigma_{4\to 6}$= 0.1102 ± 0.0063		3.86	**4.26**
	$\sigma_{6\to 4}$= 0.0362 ± 0.0025		4.65	

B	$\frac{I_{\mathrm{ij}\;(\tau_{\mathrm{mix}})}}{I_{\mathrm{ii}\;(\tau_{\mathrm{mix}})}}$	$I_{\mathrm{NOE}}\left(\tau_{\mathrm{mix}}\right)=\frac{e^{2x}-1}{e^{2x}+1}$		
**5,6**‐F_2_‐Trp CypA	$\sigma_{5\to 6}$= 1.063 ± 0.021		2.65	**2.68**
	$\sigma_{6\to 5}$= 0.914 ± 0.023		2.72	
**4,5**‐F_2_‐Trp CypA	$\sigma_{4\to 5}$= 0.680 ± 0.044		2.85	**2.63**
	$\sigma_{5\to 4}$= 1.888 ± 0.133		2.41	
**4,6**‐F_2_‐Trp CypA	$\sigma_{4\to 6}$= 0.1105 ± 0.0064		3.86	**4.26**
	$\sigma_{6\to 4}$= 0.0362 ± 0.0025		4.65	

C	$\frac{2*I_{\mathrm{ij}\;(\tau_{\mathrm{mix}})}}{I_{\mathrm{ii}\;(\tau_{\mathrm{mix}})+}I_{\mathrm{jj}\;(\tau_{\mathrm{mix}})}}$	$I_{\mathrm{NOE}}\left(\tau_{\mathrm{mix}}\right)=\frac{e^{2x}-1}{e^{2x}+1}$		
**5,6**‐F_2_‐Trp CypA	$\sigma_{5\to 6}$= 1.013 ± 0.014		2.67	**2.68**
	$\sigma_{6\to 5}$= 0.954 ± 0.021		2.70	
**4,5**‐F_2_‐Trp CypA	$\sigma_{4\to 5}$= 1.001 ± 0.057		2.67	**2.68**
	$\sigma_{5\to 4}$= 0.996 ± 0.057		2.68	
**4,6**‐F_2_‐Trp CypA	$\sigma_{4\to 6}$= 0.0566 ± 0.0022		4.32	**4.34**
	$\sigma_{6\to 4}$= 0.0536 ± 0.0038		4.36	

Given that the cross‐relaxation rate between two spins is related to the distance between the associated nuclei, we calculated NMR‐derived distances (Equation 1) for the different difluoro‐Trp CypA samples. Using the experimental rotational correlation time of 8.2 ns for CypA at 25 ^°^C [24], experimental distances between the 5 and 6 fluorine atoms, the 4 and 5 fluorine atoms, and the 4 and 6 fluorine atoms on the indole ring are provided in Table 1.

(1) $$r_{\text{ij}}^{\text{eff}}={\left({\left(\frac{\mu_{0}}{4\pi }\right)}^{2}\frac{\gamma^{4}\hbar^{2}}{10}\frac{\tau_{C}}{\sigma_{\text{ij}}}\right)}^{\frac{1}{6}}={\left(44.72\frac{\tau_{C}/\text{ns}}{\sigma_{\text{ij}}/s^{-1}}\right)}^{\frac{1}{6}}Å$$

The NMR‐derived distances can be compared to distances in X‐ray structures of reference compounds with difluoro substitution patterns equivalent to those of 5,6‐ and 4,5‐F_2_‐Trp. For (*E*)‐5,5',6,6'‐tetrafluoro‐[2,2'‐biindolinylidene]‐3,3'‐dione and 2,3‐difluoro‐10‐(5‐(4‐fluorophenyl)‐4,5‐dihydro‐1*H*‐1,2,3‐triazol‐1‐yl)pyrido[1,2‐*a*]indole, F–F distances of 2.656 and 2.643 Å were measured, and for 4,5‐difluoro‐3‐(1‐(4‐methoxyphenyl)ethyl)‐1*H*‐indole and methyl 3a‐cyano‐4,5‐difluoro‐3,3a,8,8a‐tetrahydropyrrolo[2,3‐*b*]indole‐1(2*H*)‐carboxylate, the corresponding F–F distances were 2.663 and 2.708 Å (Figures S8, S9). This compares to NMR‐derived distances of 2.68 and 2.73 Å for 5,6‐F_2_‐Trp and of 2.63–2.70 Å for 4,5‐F_2_‐Trp. For 4,6‐F_2_‐Trp, the NMR‐derived distances were 4.26 Å and 4.34 Å, depending on the different normalizing procedures. This compares well to the distance from the X‐ray structure of 4,6‐F_2_‐Trp which was measured as 4.702 Å (Figure S10). Depending on the fitting and normalization procedure used, errors ranged from ∼1–3% for 5,6‐F_2_‐Trp, 0–2% for 4,5‐F_2_‐Trp, and 8–9% for 4,6‐F_2_‐Trp. These results demonstrate that reliable F–F distances can be extracted from the initial NOESY build‐up curves.

## 3 Conclusion

In summary, in the present work we examined whether accurate fluorine–fluorine distances can be extracted from 2D ^19^F–^19^F NOESY spectra using three differently fluorinated CypA proteins as benchmarks. Our data show that distances up to ∼5 Å are accessible and that the NMR‐determined distances agree well with those from small molecule X‐ray structures of model compounds. Inclusion of such fluorine‐based distances will open doors for the application to a wide range of biological systems, including protein assemblies, protein‐ligand complexes and signaling receptors.

## Conflicts of Interest

The authors declare no conflicts of interest.

## Supporting information



The authors have cited additional references within the Supporting Information [32, 33, 34, 35, 36, 37, 38, 39, 40, 41].
**Supporting File 1**: chem71456‐sup‐0001‐SuppMat.pdf

## Data Availability

The data that support the findings of this study are available in the supplementary material of this article.
