# Supplementary material for: Assessment of Attainable Distances in Proteins by Through‐Space 19F–19F Couplings
Source: Chemistry. 2026 Jul 24;32(40):e71456. doi: 10.1002/chem.71456 (PMC13715999; doi:10.1002/chem.71456)
Supplement: Supplementary file 1 [file CHEM-32-e71456-s001.pdf]

SUPPORTING INFORMATION

Assessment of Attainable Distances in Proteins by Through-Space ^{19}F–^{19}F Couplings.

Iker Hernández, In-Ja L. Byeon, Christina M. Monnie, Vadim A. Soloshonok, Aitor Landa, Mikel Oiarbide, and Angela M. Gronenborn*

Table of contents

1. Materials and general techniques ... 2
2. Synthesis of starting materials. ... 5
   2.1. Synthesis of gramines **1a** and **1b**.[33] ... 5
3. Ethyl propiolate-promoted addition of **2** to gramines **1a** and **1b**. ... 6
4. Hydrolysis of **4** into hydantoins **5**. ... 8
5. Conversion of hydantoins **5** into difluorinated tryptophans **6**. ... 9
6. ^{1}H and ^{13}C NMR spectra. ... 11
7. High Resolution Mass Spectra (HRMS) ... 29
8. Protein preparation protocol via biosynthetic expression in *E. coli*. ... 37
9. Protein Mass Spectra, SDS-PAGE and overlaid 1D ^{19}F NMR. ... 38
10. Reference distances ... 43
11. X-Ray analysis of 4,6-F_2-Trp **6c**. ... 45
    11.1. Method for sample preparation ... 45
    11.2. Instrumentation ... 45
    11.3. Crystal parameters ... 46
    11.4. Experimental ... 50
    11.5. Crystal structure determination of [b20260008_IH46F2TrpDUCu5] ... 50
    11.6. Refinement model description ... 50
12. Protein 2D ^{19}F–^{19}F NOESY Spectra ... 52
    12.1. 5,6-F_2-Trp CypA spectra. ... 52
    12.2. 4,5-F_2-Trp CypA spectra. ... 59
    12.3. 4,6-F_2-Trp CypA spectra. ... 62
13. NOE data analysis ... 65
14. References ... 78

1. Materials and general techniques

All non-aqueous reactions were performed under inert atmosphere using oven-dried glassware and were magnetically stirred. Yields refer to chromatographically purified and spectroscopically pure compounds, unless otherwise stated. Heat-requiring reactions were performed using a hotplate with an oil bath and a condenser. Reactions requiring low temperatures were performed using cooling bath circulators Huber T100E and isopropanol baths. Organic layers washed with aqueous phases were dried over $MgSO_4$ and filtered through filter paper. Organic solvents were evaporated under reduced pressure using Büchi R-210 rotary evaporators. For trace solvent removal, a Vacuubrand PC3/RC6 vacuum pump (≈0.0003 mmHg) was used.

Solvents and reagents

Reagents were purchased from different commercial suppliers (Merck, Across, BLDpharm, etc.), stored as specified by the manufacturer and used without previous purification. Et_3N was purified by distillation over KOH. CH_2Cl_2 and CH_3CN were dried over CaH_2.

Chromatography

Reactions and flash chromatographic column purifications were monitored by thin-layer chromatography (TLC) using Merck silica gel 60 F254 plates and visualized by fluorescence quenching under UV light. In addition, TLC plates were stained with a dipping solution of potassium permanganate (1 g) in 100 mL of water (limited lifetime), followed by heating. Chromatographic purification was performed on ROCC 60 silica gel 40-63 μm as stationary phase and a suitable mixture of solvents (see experimental procedures for more details) as eluent.

Melting points

Melting points were determined in open capillaries in a Stuart SHP3 melting point apparatus.

NMR spectra

^{1}H NMR and ^{13}C NMR spectra were recorded using 300 MHz or 600 MHz and 75 MHz, 101 MHz, 126 MHz or 151 MHz spectrometers, respectively. Chemical shifts (δ) are quoted

in parts per million referenced to DMSO-d_6 (δ = 2.50), CD_3OD (δ = 3.31), CD_2Cl_2 (δ = 5.32), $CDCl_3$ (δ = 7.26) and D_2O (δ = 4.79) for ^{1}H NMR and relative to the central resonances of CD_3OD (δ = 49.0) and DMSO-d_6 (δ = 39.5) for ^{13}C NMR. ^{19}F spectra of compounds **1**, **4**, **5** and **6** were recorded at 298K on a Bruker Avance Neo 400, equipped with Iprobe, at 376 MHz using fluorobenzene as internal reference (−113.05 ppm). The multiplicity of each signal is designated using the following abbreviations: s, singlet; d, doublet; dd, doublet of doublets; ddd, doublet of doublets of doublets; m, multiplet. Coupling constants (J) are reported in Hertz (Hz).

Protein NMR measurements were performed on a 14.1 T Bruker Avance spectrometer, equipped with a CP TXO F/C–H–D triple-resonance, z-axis gradient cryoprobe. The Larmor frequency of ^{1}H and ^{19}F are 600.1 and 564.6 MHz, respectively. The temperature in all NMR experiments was maintained at 298 K using the Bruker temperature controller. ^{1}H and ^{19}F chemical shifts were referenced with respect to DSS (4,4-dimethyl-4-silapentane-1-sulfonic acid) and TFA (trifluoroacetic acid). For comparative purposes, additional ^{19}F NMR spectra of the free difluorinated Trp derivatives were recorded in H_2O and referenced to TFA. The resulting ^{19}F chemical shifts correspond to those indicated by lines in Figure 1b of the main manuscript.

Data were processed and analyzed with Topspin 3.1 and MestReNova 14.2.3 softwares.

Mass spectra

Mass spectra of **1**, **4**, **5** and **6** were recorded on an ESI-ion trap Mass spectrometer (Agilent 1100 series LC/MSD, SL model) by the General Research Service (SGIker) of the University of the Basque Country (UPV/EHU).

IR spectra

Infrared spectra were measured employing a Bruker ALPHA-P compact FT-IR spectrometer.

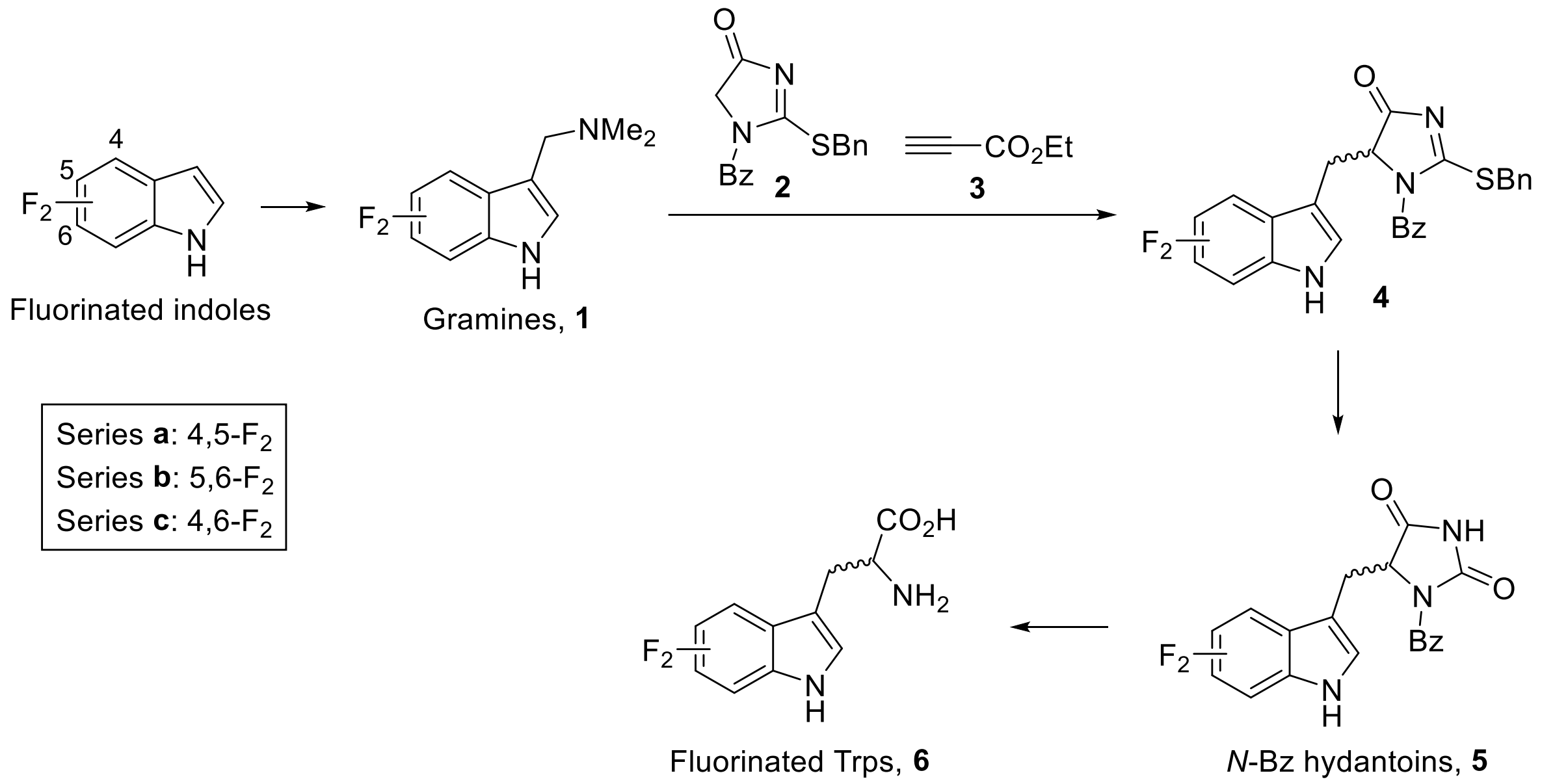

Scheme S1: Chemical structures and numbers of small molecules appearing in the SI.

2. Synthesis of starting materials.

Compound **3** (ethyl propiolate) was purchased from BLDpharm and used without further purification. Compounds, **2** (1-benzoyl-2-(benzylthio)-1,5-dihydro-4*H*-imidazol-4-one)[32] and **6c** (4,6-F_2-Trp)[27] are known compounds and were synthesized following reported experimental procedures.

2.1. Synthesis of gramines 1a and 1b.[33]

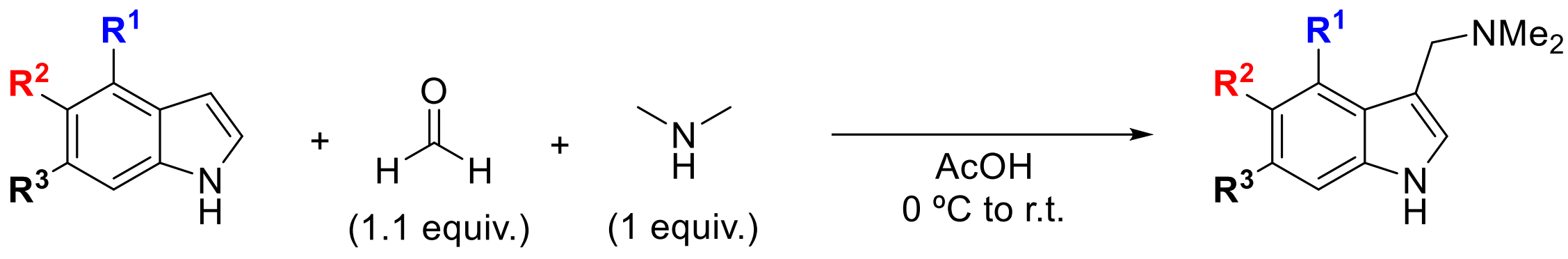

1a R1, R2 = F, R3: H
1b R2, R3 = F, R1: H

To a solution of dimethylamine (2.0 M in THF, 10.2 mL, 20.4 mmol) in a 50 mL round bottom flask at 0 ºC, formaldehyde (37 % w/v in H_2O, 1.725 mL, 22.4 mmol, 1.1 equiv.) and glacial acetic acid (1.8 mL, 47.5 mmol, 2.3 equiv.) were added and the solution was stirred for 10 min. To this mixture, a solution of the corresponding indole (20.4 mmol) dissolved in glacial acetic acid (2.4 mL) was added dropwise. Note: when the indole did not completely dissolve in the specified volume of acetic acid, up to 5 mL of additional THF was added. Once all components were combined, the reaction mixture was slowly brought to room temperature and stirred overnight. The reaction solution was then poured into water and brought to an alkaline pH by the addition of 10% aqueous NaOH. The resulting precipitate was filtered off, washed with water and hexane and dried under reduced pressure until all solvents and water were removed.

1-(4,5-Difluoro-1*H*-indol-3-yl)-*N*,*N*-dimethylmethanamine (1a)

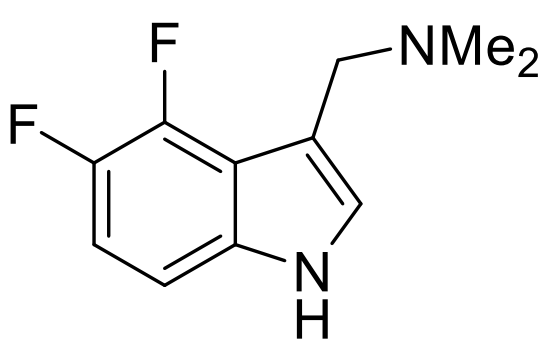

The title compound was prepared from 4,5-difluoro-1*H*-indole (3.12 g, 20.4 mmol, 1.0 equiv.) according to the general procedure. White solid. Yield: 3.29 g, 15.7 mmol, 77%. m.p. = 141–145 ºC. ^{1}H NMR (300 MHz, $CDCl_3$) δ 8.21 (s, 1H), 7.26 (s, 1H), 7.11 (d, *J* = 2.4 Hz, 1H), 7.04 – 6.94 (m, 1H), 3.70 (s, 2H), 2.32 (s, 6H). ^{13}C NMR (101 MHz, DMSO-d_6) δ 143.2 (dd, *J* = 230.9, 11.1 Hz), 142.8 (dd, *J* = 245.8, 14.0 Hz), 134.8 (d, *J* = 10.5 Hz), 127.1, 116.8 (d, *J* =

15.7 Hz), 110.6 (dd, J = 5.4, 3.7 Hz), 110.3 (d, J = 21.3 Hz), 107.4 (dd, J = 7.9, 4.0 Hz), 54.4, 44.6. ^{19}F NMR (376 MHz, DMSO-d_6) δ −150.57 (dd, $J^3_{(4F\text{-}5F)}$ = 22.1 Hz, $J^4_{(4F\text{-}6H)}$ = 7.5 Hz), −154.13 (ddd, $J^3_{(5F\text{-}4F)}$ = 21.8 Hz, $J^3_{(5F\text{-}6H)}$ = 11.3 Hz, $J^4_{(5F\text{-}7H)}$ = 3.6 Hz). IR (cm^{-1}) = 2649, 1525, 1473, 1347, 1238, 1094, 1014, 933, 757, 537, 406. HRMS (ESI) m/z: [M+H]$^+$ Calcd. for $C_{11}H_{13}F_2N_2$ 211.1041; Found 211.1047.

1-(5,6-Difluoro-1*H*-indol-3-yl)-*N*,*N*-dimethylmethanamine (1b)

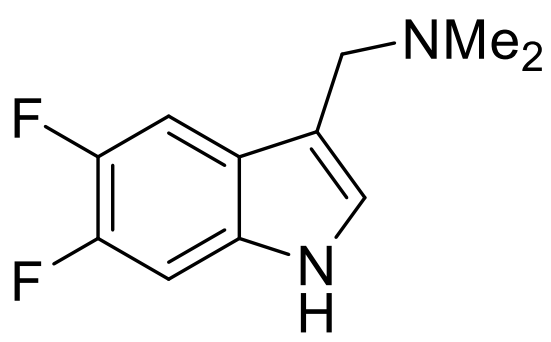

The title compound was prepared from 5,6-difluoro-1*H*-indole (3.12 g, 20.4 mmol, 1.0 equiv.) according to the general procedure. Off-white solid. Yield: 2.84 g, 13.5 mmol, 66%. m.p. = 143–147 ºC. ^{1}H NMR (300 MHz, DMSO-d_6) δ 11.08 (s, 1H), 7.50 (dd, J = 11.5, 8.2 Hz, 1H), 7.34 (dd, J = 11.3, 7.0 Hz, 1H), 7.27 (s, 1H), 3.47 (s, 2H), 2.12 (s, 6H). ^{13}C NMR (75 MHz, DMSO-d_6) δ 146.5 (dd, J_{CF} = 236.6, 15.7 Hz), 144.9 (dd, J_{CF} = 233.7, 14.9 Hz), 131.3 (d, J_{CF} = 10.7 Hz), 126.2 (d, J_{CF} = 3.6 Hz), 122.9 (d, J_{CF} = 8.0 Hz), 112.3 (d, J_{CF} = 4.4 Hz), 105.6 (d, J_{CF} = 18.7 Hz), 99.2 (d, J_{CF} = 21.1 Hz), 54.3, 44.8. ^{19}F NMR (376 MHz, DMSO-d_6) δ −146.0 (ddd, $J^3_{(6F\text{-}5F)}$ = 22.3 Hz, $J^3_{(6F\text{-}7H)}$ = 11.3 Hz, $J^4_{(6F\text{-}4H)}$ = 8.1 Hz), −149.4 (ddd, $J^3_{(5F\text{-}6F)}$ = 21.9 Hz, $J^3_{(5F\text{-}4H)}$ = 11.5 Hz, $J^4_{(5F\text{-}7H)}$ = 7.1 Hz). IR (cm^{-1}) = 2775, 1975, 1472, 1353, 1337, 1189, 997, 840, 632, 423. HRMS (ESI) m/z: [M+H]$^+$ Calcd. for $C_{11}H_{13}F_2N_2$ 211.1041; Found 211.1052.

3. Ethyl propiolate-promoted addition of 2-benzylthioimidazolone 2 to gramines 1a and 1b.

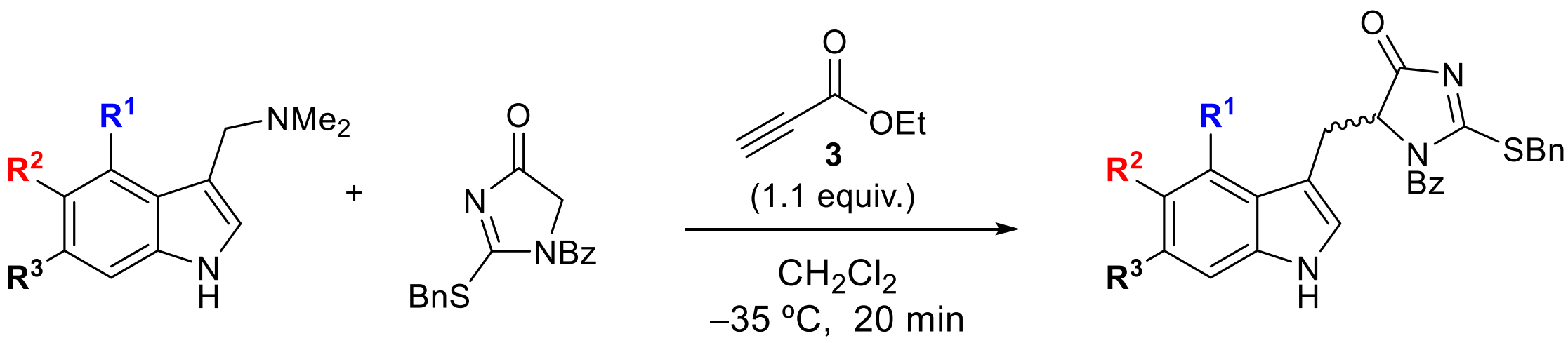

1a **R^1**, **R^2** = **F**, **R^3**: H
1b **R^2**, **R^3** = **F**, **R^1**: H

2

4a **R^1**, **R^2** = **F**, **R^3**: H
4b **R^2**, **R^3** = **F**, **R^1**: H

To a solution of the corresponding gramine **1** (8 mmol) in dry CH_2Cl_2 (80 mL) at room temperature, 2-benzylthioimidazolone **2** (4.97 g, 16 mmol, 2 equiv.) was added. After cooling the solution to −40 °C, ethyl propiolate **3** (0.86 g, 8.8 mmol, 1.1 equiv.) was added and the reaction mixture was stirred at the same temperature for 10 minutes. Afterwards, the mixture was warmed slowly to −35 °C over 30 minutes. The solvent was evaporated under reduced

pressure and the crude compound was purified by column chromatography (eluent: hexane/ethyl acetate from 5:1 to 1:1).

1-Benzoyl-2-(benzylthio)-5-((4,5-difluoro-1*H*-indol-3-yl)methyl)-1,5-dihydro-4*H*-imidazol-4-one (4a)

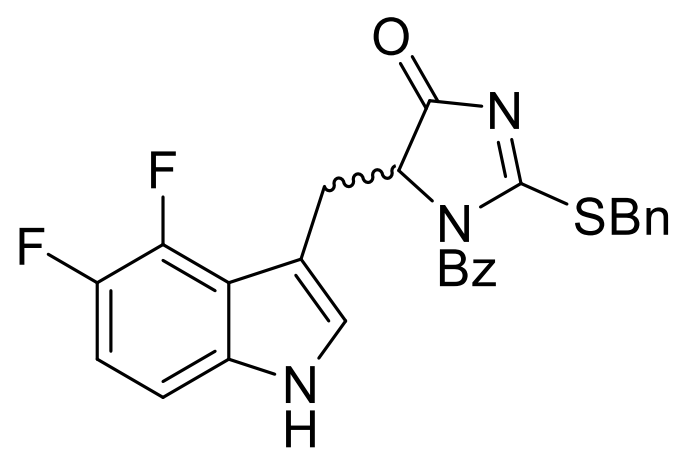

The title compound was prepared from gramine **1a** (1.68 g, 8 mmol) according to the general procedure. Yellow foam. Yield: 3.46 g, 7.27 mmol, 91%. ^{1}H NMR (300 MHz, DMSO-d_6) δ 11.22 (s, 1H), 7.60 – 7.51 (m, 2H), 7.45 – 7.36 (m, 1H), 7.35 – 7.23 (m, 7H), 7.08 – 6.91 (m, 3H), 5.15 (dd, *J* = 6.2, 4.8 Hz, 1H), 4.42 – 4.29 (m, 2H), 3.07 (dd, *J* = 15.1, 6.2 Hz, 1H), 2.91 (dd, *J* = 15.1, 4.7 Hz, 1H). ^{13}C NMR (101 MHz, DMSO-d_6) δ 184.5, 184.4, 167.0, 143.0 (dd, J_{CF} = 231.2, 11.3 Hz), 142.5 (dd, J_{CF} = 245.0, 14.4 Hz), 136.0, 134.6 (d, J_{CF} = 10.0 Hz), 132.8, 131.7, 129.1, 128.6, 128.3, 127.5, 127.4, 126.4, 116.2 (d, J_{CF} = 14.8 Hz), 110.5 (d, J_{CF} = 21.4 Hz), 107.5 (d, J_{CF} = 6.3 Hz), 105.7 (d, J_{CF} = 5.1 Hz), 65.3, 36.6, 27.6. ^{19}F NMR (376 MHz, DMSO-d_6) δ −151.71 (dd, $J^3_{(4F\text{-}5F)}$ = 22.0 Hz, $J^4_{(4F\text{-}6H)}$ = 7.2 Hz), −154.10 (ddd, $J^3_{(5F\text{-}4F)}$ = 22.2 Hz, $J^3_{(5F\text{-}6H)}$ = 11.0 Hz, $J^4_{(5F\text{-}7H)}$ = 4.0 Hz). IR (cm^{-1}) = 1731, 1670, 1519, 1440, 1266, 1146, 939, 704, 659. HRMS (ESI) m/z: [M+Na]$^+$ Calcd. for $C_{26}H_{19}F_2N_3O_2SNa$ 498.1058; Found 498.1067.

1-Benzoyl-2-(benzylthio)-5-((5,6-difluoro-1*H*-indol-3-yl)methyl)-1,5-dihydro-4*H*-imidazol-4-one (4b)

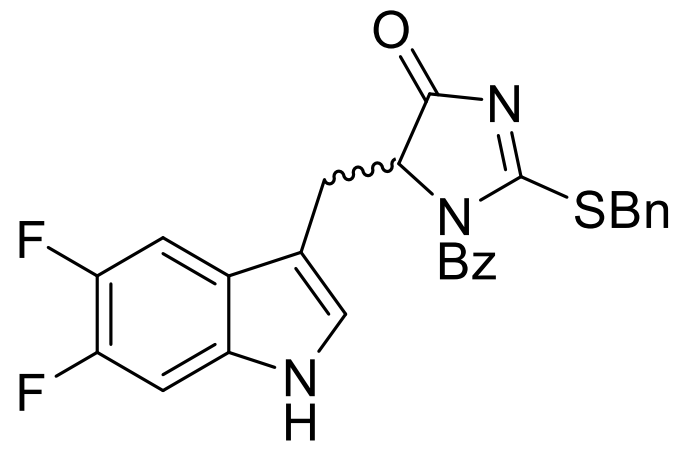

The title compound was prepared from gramine **1b** (1.68 g, 8 mmol) according to the general procedure. White foam. Yield: 3.36 g, 7.1 mmol, 88%. ^{1}H NMR (300 MHz, $CDCl_3$) δ 8.00 (s, 1H), 7.63 – 6.86 (m, 13H), 4.93 (dd, *J* = 6.1, 3.3 Hz, 1H), 4.31 (s, 2H), 3.28 (dd, *J* = 15.1, 3.4 Hz, 1H), 3.03 (dd, *J* = 15.1, 6.1 Hz, 1H). ^{13}C NMR (126 MHz, DMSO-d_6) δ 184.9, 184.7, 166.9, 146.5 (dd, J_{CF} = 237.2, 15.9 Hz), 144.9 (dd, J_{CF} = 234.1, 15.0 Hz), 136.0, 133.3, 132.4, 130.8 (d, J_{CF} = 10.7 Hz), 129.0, 128.9, 128.5, 127.7, 127.4, 126.0 (d, J_{CF} = 3.3 Hz), 122.2 (d, J_{CF} = 7.9 Hz), 106.6 (d, J_{CF} = 3.8 Hz), 105.0 (d, J_{CF} = 19.0 Hz), 99.2 (d, J_{CF} = 21.1 Hz), 64.7, 36.4, 25.6. ^{19}F NMR (376 MHz, DMSO-d_6) δ −145.6 (ddd, $J^3_{(6F\text{-}5F)}$ = 22.4 Hz, $J^3_{(6F\text{-}7H)}$ = 11.2 Hz, $J^4_{(6F\text{-}4H)}$ = 7.9 Hz), −149.1 (ddd, $J^3_{(5F\text{-}6F)}$ = 22.3 Hz, $J^3_{(5F\text{-}4H)}$ = 11.6 Hz, $J^4_{(5F\text{-}7H)}$ = 7.1 Hz). IR (cm^{-1}) = 2178, 1978, 1726, 1676, 1443, 1336, 1260, 1141, 841, 698, 660, 461. HRMS (ESI) m/z: [M+H]$^+$ Calcd. for $C_{26}H_{20}F_2N_3O_2S$ 476.1239; Found 476.1252.

4. Hydrolysis of 4 into hydantoins 5.

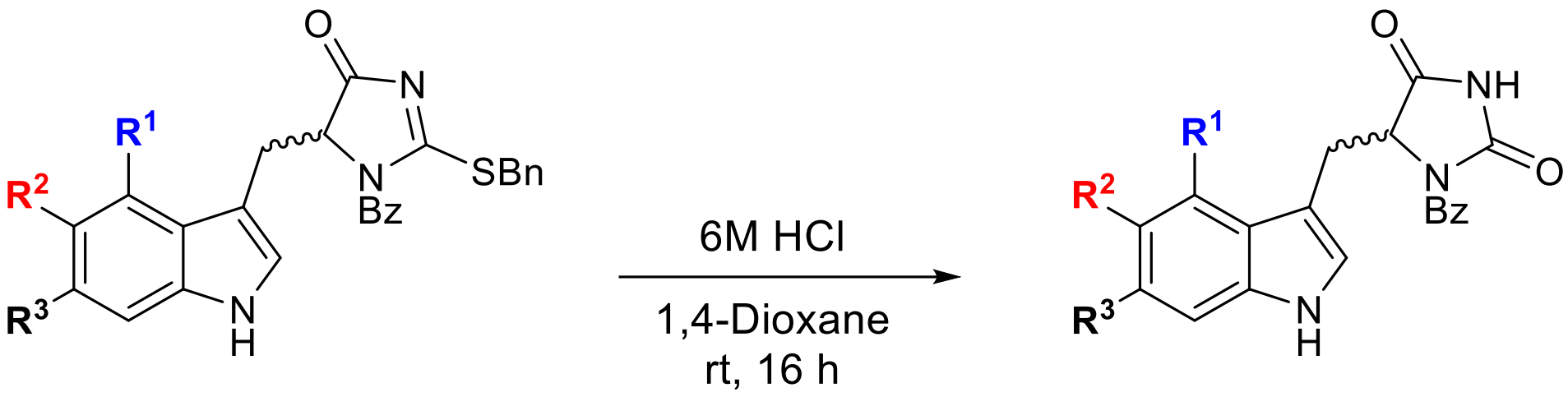

4a R1, R2 = F, R3: H
4b R2, R3 = F, R1: H

5a R1, R2 = F, R3: H
5b R2, R3 = F, R1: H

A solution of aq. HCl 6M (~2 ml/mmol of **4**) was added dropwise to a solution of the corresponding imidazolone **4** (~7 mmol) in 1,4-dioxane (15 mL per mmol of **4**) at 0 °C. Once the addition was complete, the reaction was stirred at room temperature overnight. Afterward, the mixture was cooled to 0 °C and a saturated solution of $NaHCO_3$ was added until basic pH was reached. The aqueous layer was extracted with CH_2Cl_2 (3 x 100 mL), the combined organic layers were dried over $MgSO_4$ and the solvent evaporated under reduced pressure. The crude product was purified by silica gel flash column chromatography (from CH_2Cl_2 to CH_2Cl_2/MeOH 99:1) followed by trituration with CH_2Cl_2.

1-Benzoyl-5-((4,5-difluoro-1*H*-indol-3-yl)methyl)imidazolidine-2,4-dione (5a)

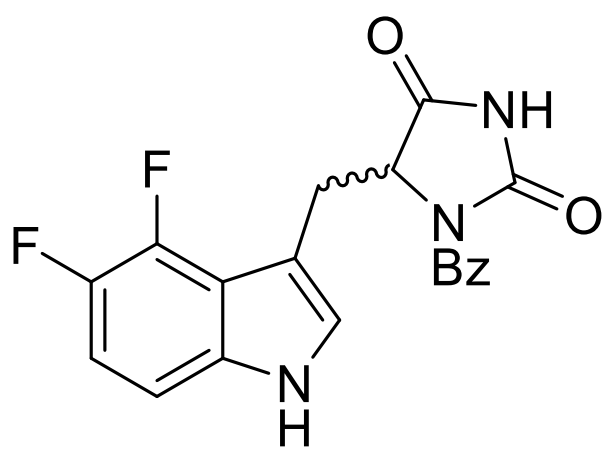

The title compound was prepared using aq. HCl 6 M (13.5 mL) and 1-benzoyl-2-(benzylthio)-5-((4,5-difluoro-1*H*-indol-3-yl)methyl)-1,5-dihydro-4*H*-imidazol-4-one (3.46 g, 7.27 mmol) according to the general procedure. Yellow foam. Yield: 1.59 g, 4.31 mmol, 59%. ^{1}H NMR (300 MHz, $CDCl_3$) δ 8.17 (s, 1H), 7.56 – 6.93 (m, 8H), 5.30 (dd, J = 5.2, 3.3 Hz, 1H), 3.83 (dd, J = 15.2, 5.2 Hz, 1H), 3.57 (dd, J = 15.2, 3.3 Hz, 1H). ^{13}C NMR (126 MHz, DMSO-d_6) δ 172.4, 167.7, 153.5, 143.1 (dd, J_{CF} = 231.7, 11.2 Hz), 134.4 (d, J_{CF} = 10.2 Hz), 142.5 (dd, J_{CF} = 245.6, 14.5 Hz), 134.5, 134.4 (d, J_{CF} = 10.2 Hz), 131.6, 128.6, 127.3, 126.9, 117.0 (d, J_{CF} = 14.6 Hz), 110.6 (d, J_{CF} = 21.5 Hz), 107.7 (dd, J_{CF} = 7.8, 3.7 Hz), 105.7 (dd, J_{CF} = 5.5, 2.1 Hz), 61.0, 24.8. ^{19}F NMR (376 MHz, DMSO-d_6) δ −150.65 (dd, $J^3_{(4F-5F)}$ = 22.0 Hz, $J^4_{(4F-6H)}$ = 7.4 Hz), −153.94 (ddd, $J^3_{(5F-4F)}$ = 22.0 Hz, $J^3_{(5F-6H)}$ = 11.1 Hz, $J^4_{(5F-7H)}$ = 3.7 Hz). IR (cm^{-1}) = 2158, 1732, 1518, 1291, 1177, 947, 691, 633, 455. HRMS (ESI) m/z: $[M+H]^+$ Calcd. for $C_{19}H_{14}F_2N_3O_3$ 370.0998; Found 370.1004.

1-Benzoyl-5-((5,6-difluoro-1*H*-indol-3-yl)methyl)imidazolidine-2,4-dione (5b)

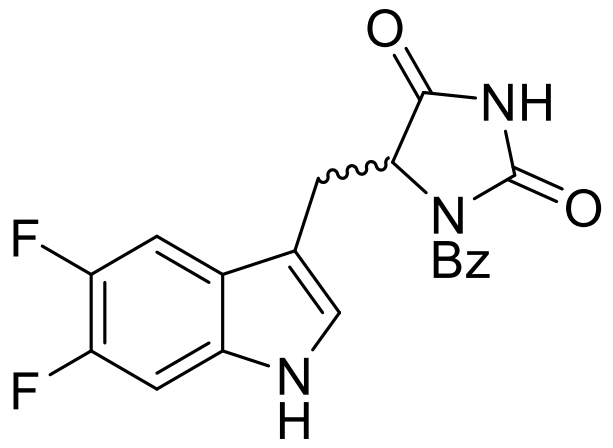

The title compound was prepared using aq. HCl 6 M (13 mL) and 1-benzoyl-2-(benzylthio)-5-((5,6-difluoro-1*H*-indol-3-yl)methyl)-1,5-dihydro-4*H*-imidazol-4-one (3.36 g, 7.1 mmol) according to the general procedure. White solid, m.p.: 211–214. Yield: 1.75 g, 4.75 mmol, 67%. ^{1}H NMR (300 MHz, CD_2Cl_2) δ 8.30 (s, 1H), 7.58 – 7.14 (m, 7H), 7.06 (d, J = 2.5 Hz, 1H), 5.14 (dd, J = 5.7, 2.5 Hz, 1H), 3.66 (dd, J = 15.2, 5.8 Hz, 1H), 3.53 (ddd, J = 15.1, 2.5, 0.8 Hz, 1H). ^{13}C NMR (101 MHz, DMSO-d_6) δ 173.1, 168.0, 153.4, 146.6 (dd, J_{CF} = 237.2, 15.9 Hz), 145.1 (dd, J_{CF} = 234.3, 15.0 Hz), 134.5, 131.5, 131.0 (d, J_{CF} = 10.9 Hz), 128.4, 127.5, 126.4, 122.8 (d, J_{CF} = 7.7 Hz), 107.5 (d, J_{CF} = 4.3 Hz), 105.0 (d, J_{CF} = 18.9 Hz), 99.5 (d, J_{CF} = 21.1 Hz), 60.7, 23.7. ^{19}F NMR (376 MHz, DMSO-d_6) δ −145.6 (ddd, $J^3_{(6F\text{-}5F)}$ = 22.4 Hz, $J^3_{(6F\text{-}7H)}$ = 11.2 Hz, $J^4_{(6F\text{-}4H)}$ = 8.1 Hz), −149.0 (ddd, $J^3_{(5F\text{-}6F)}$ = 22.4 Hz, $J^3_{(5F\text{-}4H)}$ = 11.6 Hz, $J^4_{(5F\text{-}7H)}$ = 7.0 Hz). IR (cm^{-1}) = 1732, 1654, 1474, 1342, 1310, 1131, 976, 844, 720, 642, 469. HRMS (ESI) m/z: $[M+H]^+$ Calcd. for $C_{19}H_{14}F_2N_3O_3$ 370.0998; Found 370.0998.

5. Conversion of hydantoins 5 into difluorinated tryptophans 6.

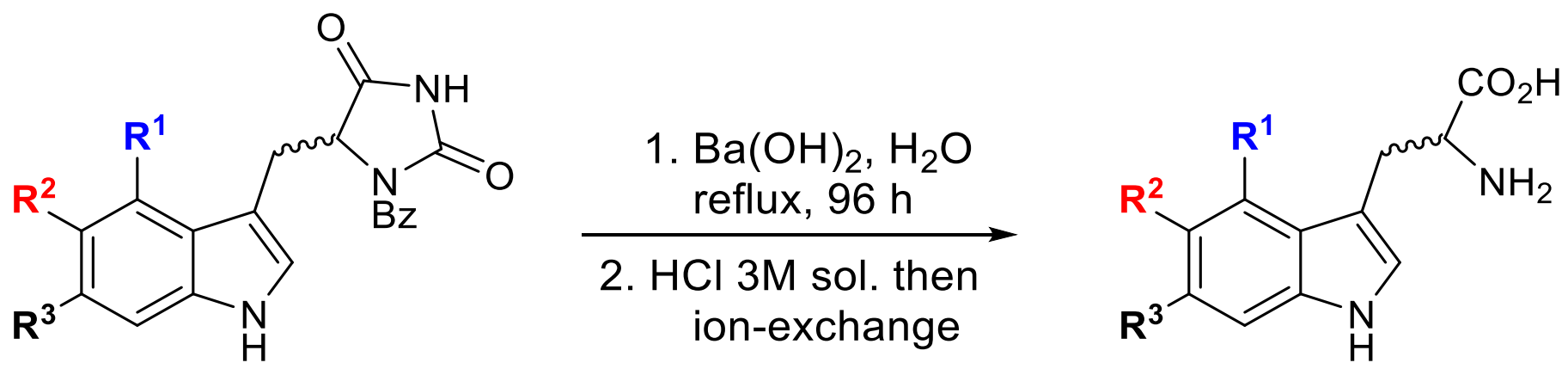

5a R^1, R^2 = F, R^3: H
5b R^2, R^3 = F, R^1: H

6a R^1, R^2 = F, R^3: H
6b R^2, R^3 = F, R^1: H

To a solution of the corresponding *N*-benzoyl hydantoin **5** (1 equiv.) in H_2O (10 mL per mmol of **5**) at 0 °C (ice bath), $Ba(OH)_2 \cdot H_2O$ (4 equiv.) was added. The resulting mixture was refluxed for 96 hours, during which ammonia gas evolved and barium carbonate precipitate was formed. The mixture was then cooled down to room temperature and the precipitate was filtered and washed with water. The aqueous layer was acidified with 3 M HCl (6 equiv.) after which water and HCl were removed under reduced pressure in the rotary evaporator, followed by vacuum pumping. The resulting solid was dissolved in water and then loaded into a dowex® 50WX2 (50–100 mesh) ion exchange resin (previously washed with 1.0 M HCl). Afterwards, the resin was treated with water until the pH of the eluent was neutral. The compound was

eluted with a 5% NH_3 aqueous solution. The obtained basic solution was concentrated under vacuum to yield the corresponding amino acid on its zwitterionic form.

2-Amino-3-(4,5-difluoro-1*H*-indol-3-yl)propanoic acid (6a)

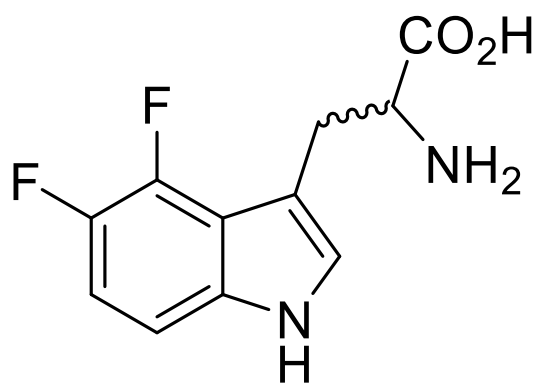

The title compound was prepared from 1-benzoyl-5-((4,5-difluoro-1*H*-indol-3-yl)methyl)imidazolidine-2,4-dione (1.59 g, 4.31 mmol) and $Ba(OH)_2 \cdot H_2O$ (3.26 g, 17.24 mmol, 4 equiv.) according to the general procedure. Light brown solid, m.p.: 245–248. Yield: 0.92 g, 3.83 mmol, 89%. ^{1}H NMR (300 MHz, D_2O) δ 7.37 – 7.05 (m, 3H), 4.06 (dd, *J* = 8.9, 4.9 Hz, 1H), 3.66 – 3.51 (dd, *J* = 15.2, 4.9 Hz, 1H), 3.26 (dd, *J* = 15.1, 8.9 Hz, 1H). ^{13}C NMR (126 MHz, CD_3OD) δ 174.1, 145.3 (dd, J_{CF} = 232.6, 11.0 Hz), 144.8 (dd, J_{CF} = 244.5, 14.7 Hz), 136.8 (d, J_{CF} = 10.1 Hz), 127.6, 118.0 (d, J_{CF} = 14.9 Hz), 112.2 (d, J_{CF} = 22.1 Hz), 108.9 (dd, J_{CF} = 5.6, 2.8 Hz), 108.3 (dd, J_{CF} = 7.7, 4.1 Hz), 57.3, 29.3. ^{19}F NMR (376 MHz, DMSO-d_6) δ −152.02 (dd, $J^3_{(4F-5F)}$ = 22.1 Hz, $J^4_{(4F-6H)}$ = 7.4 Hz), −154.52 (ddd, $J^3_{(5F-4F)}$ = 22.1 Hz, $J^3_{(5F-6H)}$ = 11.0 Hz, $J^4_{(5F-7H)}$ = 3.5 Hz). IR (cm^{-1}) = 3461, 1589, 1513, 1408, 1352, 942, 783, 618, 512. HRMS (ESI) m/z: $[M+H]^+$ Calcd. for $C_{11}H_{11}F_2N_2O_2$ 241.0783; Found 241.0792.

2-amino-3-(5,6-difluoro-1*H*-indol-3-yl)propanoic acid (6b)

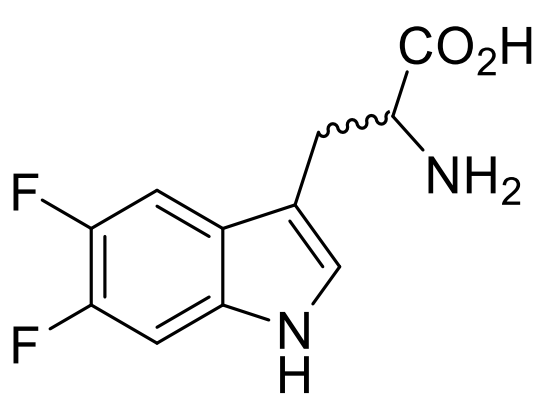

The title compound was prepared from 1-benzoyl-5-((5,6-difluoro-1*H*-indol-3-yl)methyl)imidazolidine-2,4-dione (1.75 g, 4.75 mmol) and $Ba(OH)_2 \cdot H_2O$ (3.60 g, 19 mmol, 4 equiv.) according to the general procedure. Off-white solid, m.p.: 230–235. Yield: 1.05 g, 4.35 mmol, 92%. ^{1}H NMR (300 MHz, D_2O) δ 7.52 (dd, *J* = 11.4, 7.9 Hz, 1H), 7.36 (dd, *J* = 11.2, 6.9 Hz, 1H), 7.32 (s, 1H), 4.03 (dd, *J* = 7.6, 5.0 Hz, 1H), 3.41 (dd, *J* = 15.4, 5.0 Hz, 1H), 3.28 (dd, *J* = 15.4, 7.6 Hz, 1H). ^{13}C NMR (151 MHz, CD_3OD) δ 174.2, 149.1 (dd, J_{CF} = 260.6, 15.5 Hz), 147.5 (dd, J_{CF} = 257.9, 15.5 Hz), 133.3 (d, J_{CF} = 10.4 Hz), 127.0 (d, J_{CF} = 3.5 Hz), 124.0 (d, J_{CF} = 7.7 Hz), 110.0 (d, J_{CF} = 4.4 Hz), 106.1 (d, J_{CF} = 19.5 Hz), 100.0 (d, J_{CF} = 21.7 Hz), 56.5, 28.2. ^{19}F NMR (376 MHz, DMSO-d_6) δ −146.2 (ddd, $J^3_{(6F-5F)}$ = 22.3 Hz, $J^3_{(6F-7H)}$ = 11.2 Hz, $J^4_{(6F-4H)}$ = 8.0 Hz), −149.5 (ddd, $J^3_{(5F-6F)}$ = 22.6 Hz, $J^3_{(5F-4H)}$ = 11.6 Hz, $J^4_{(5F-7H)}$ = 7.0 Hz). IR (cm^{-1}) = 2034, 1586, 1472, 1336, 842, 515, 418. HRMS (ESI) m/z: $[M+H]^+$ Calcd. for $C_{11}H_{11}F_2N_2O_2$ 241.0783; Found 241.0792.

6. ^{1}H and ^{13}C NMR spectra.

^{1}H NMR (300 MHz, $CDCl_3$) of **1a**:

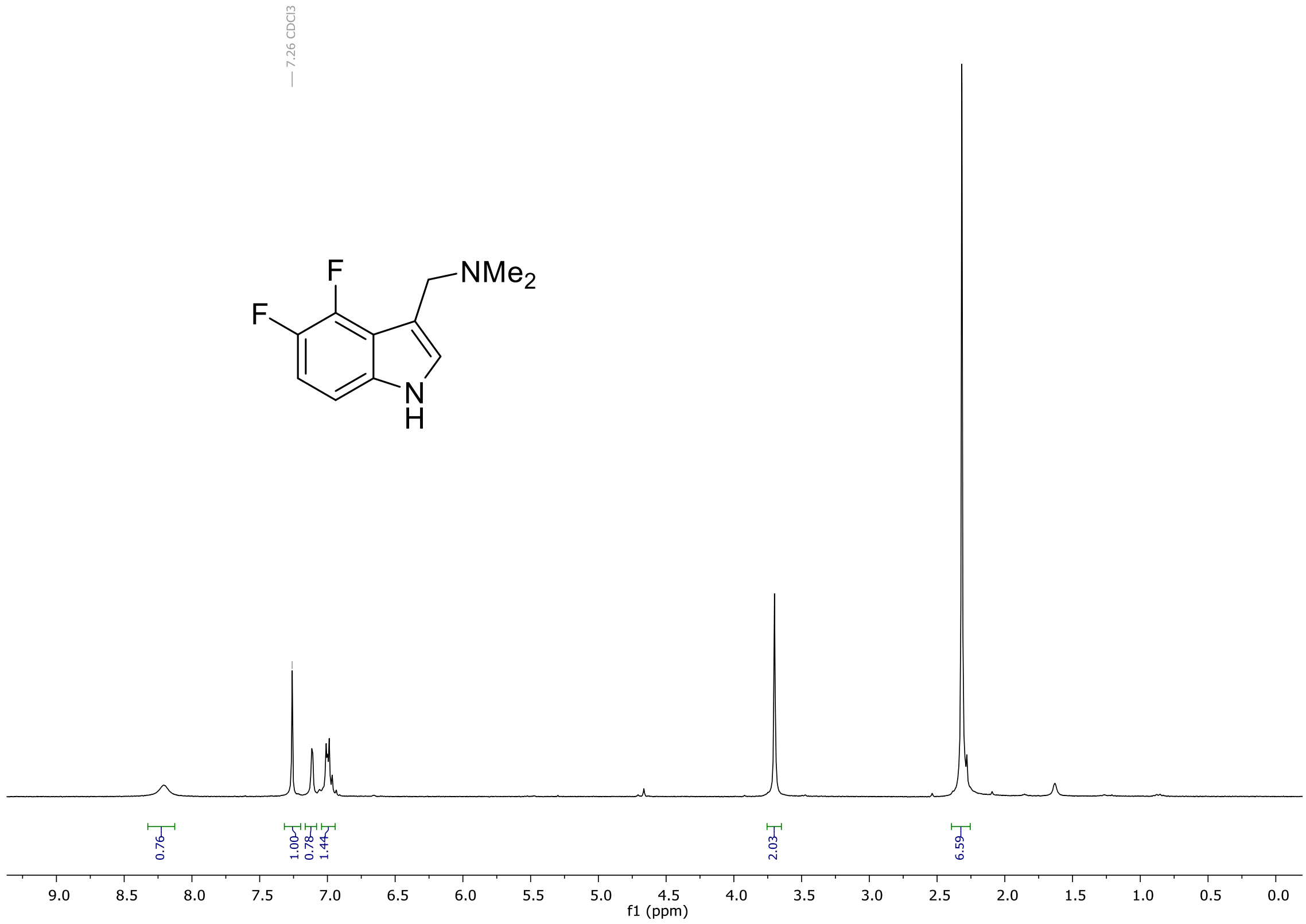

^{13}C{^{1}H} NMR (101 MHz, DMSO-d_6) of **1a**:

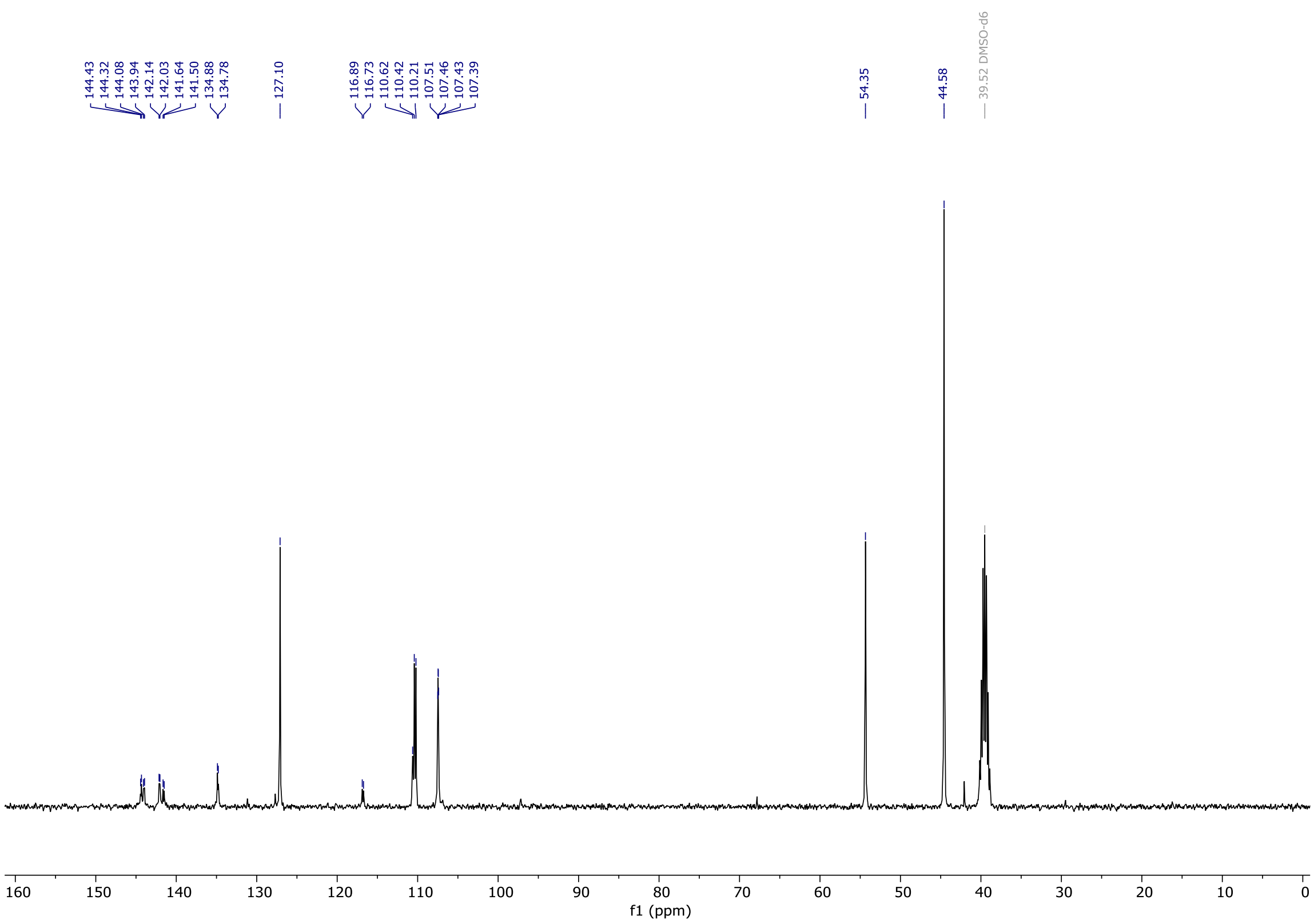

^{19}F NMR (376 MHz, DMSO-d6) of **1a**:

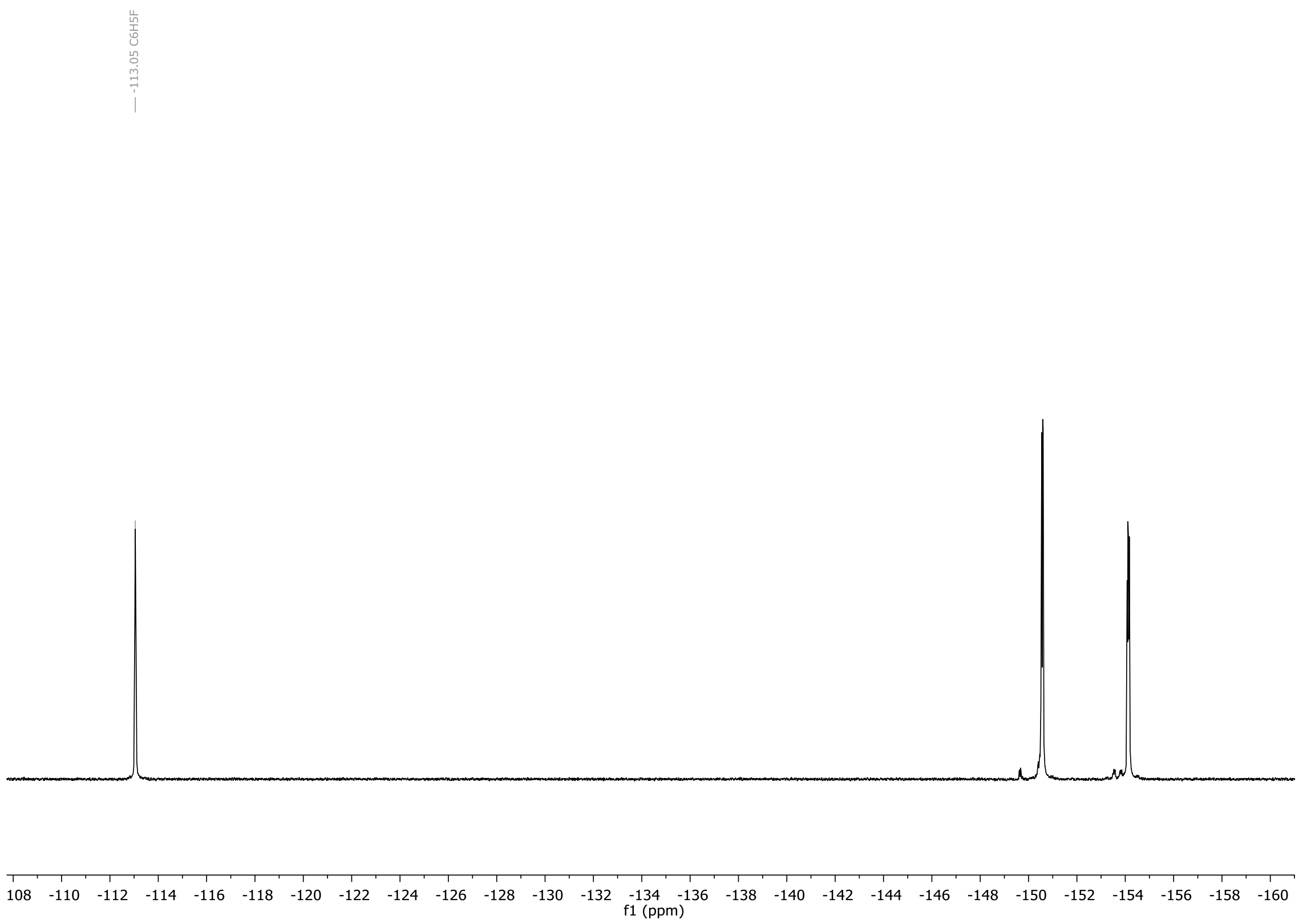

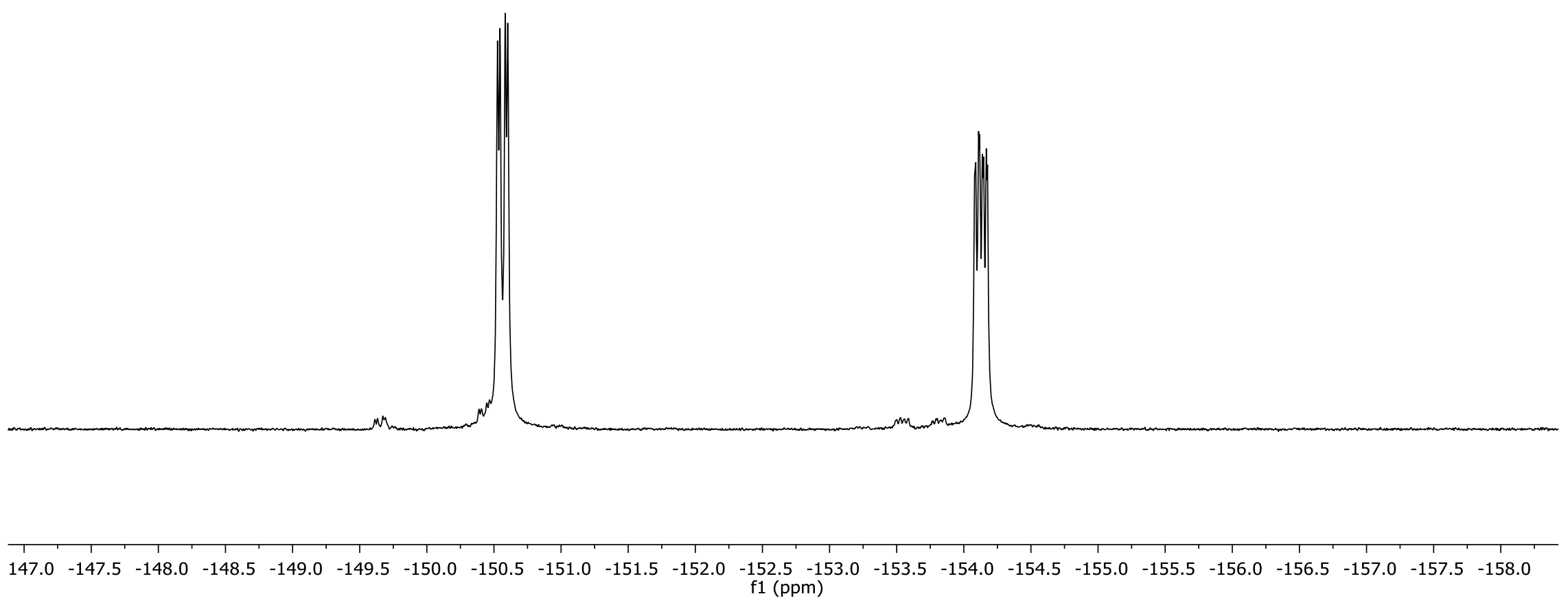

^{1}H NMR (300 MHz, DMSO-d6) of **1b:**

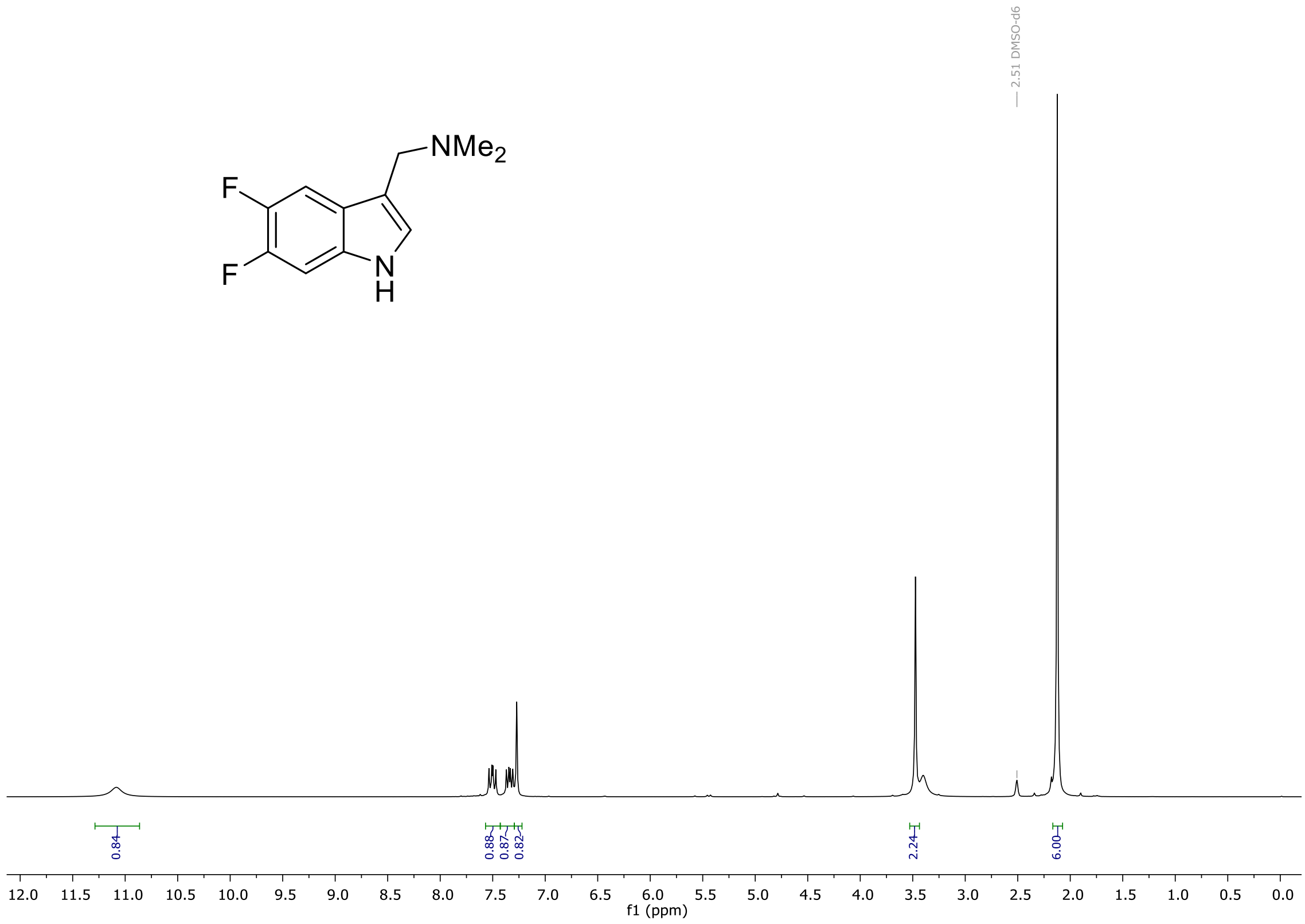

$^{13}C\{^{1}H\}$ NMR (75 MHz, DMSO-d6) of **1b:**

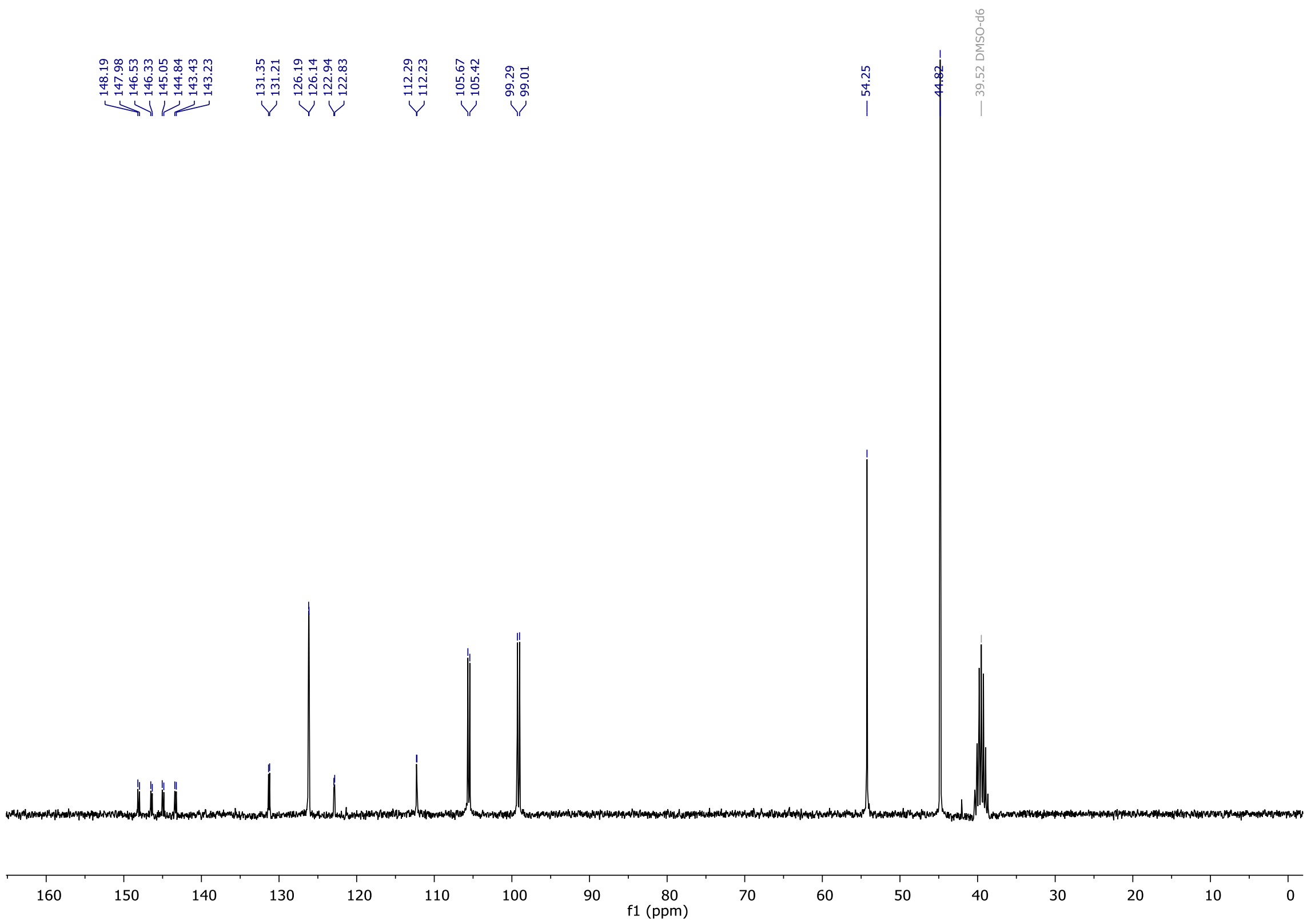

^{19}F NMR (376 MHz, DMSO-d6) of **1b:**

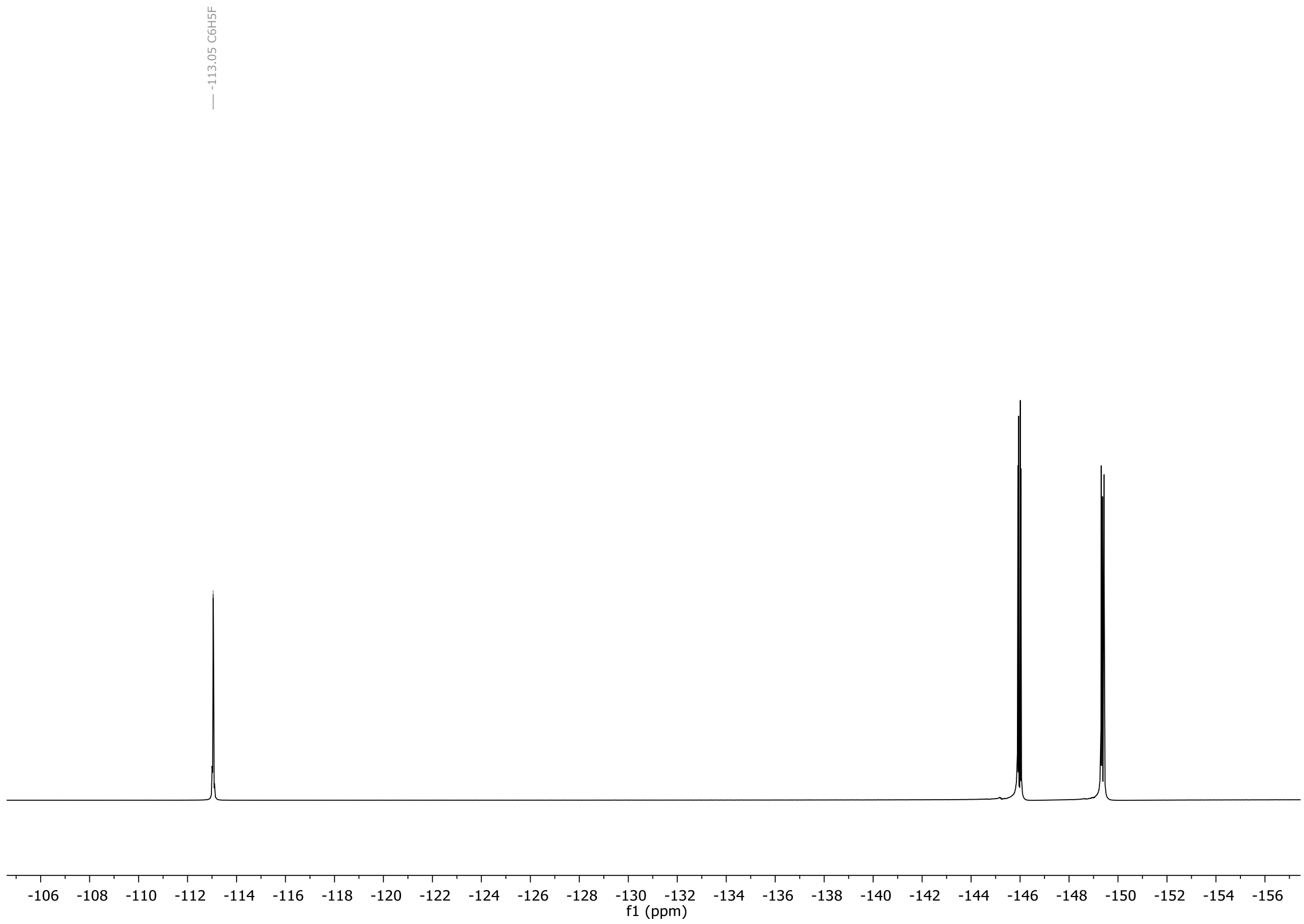

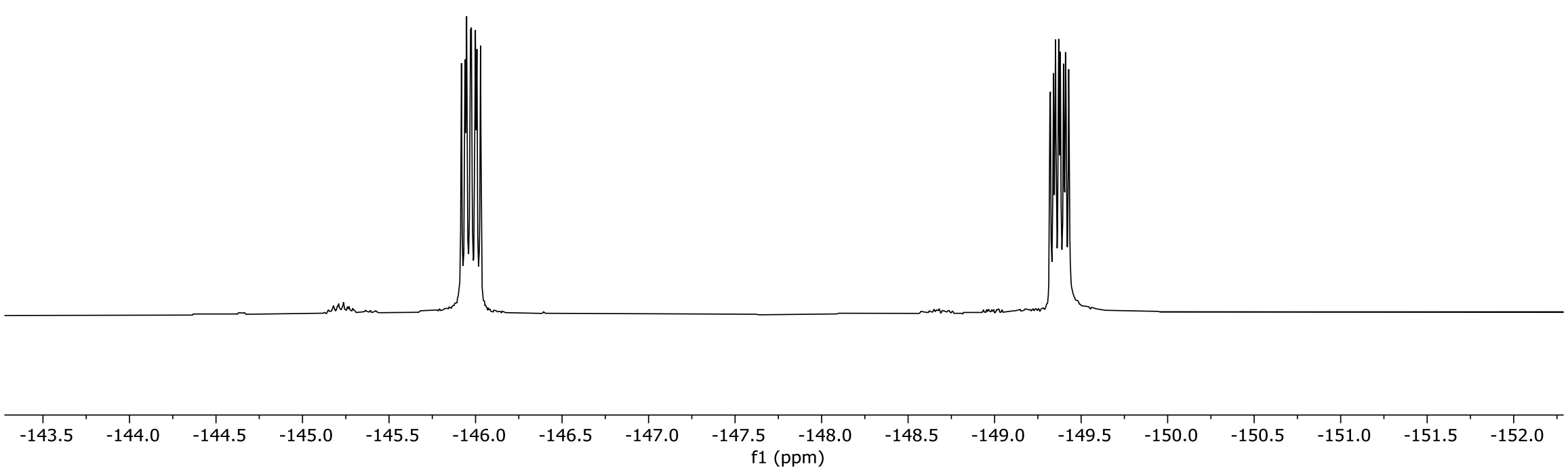

^{1}H NMR (300 MHz, DMSO-d6) of **4a:**

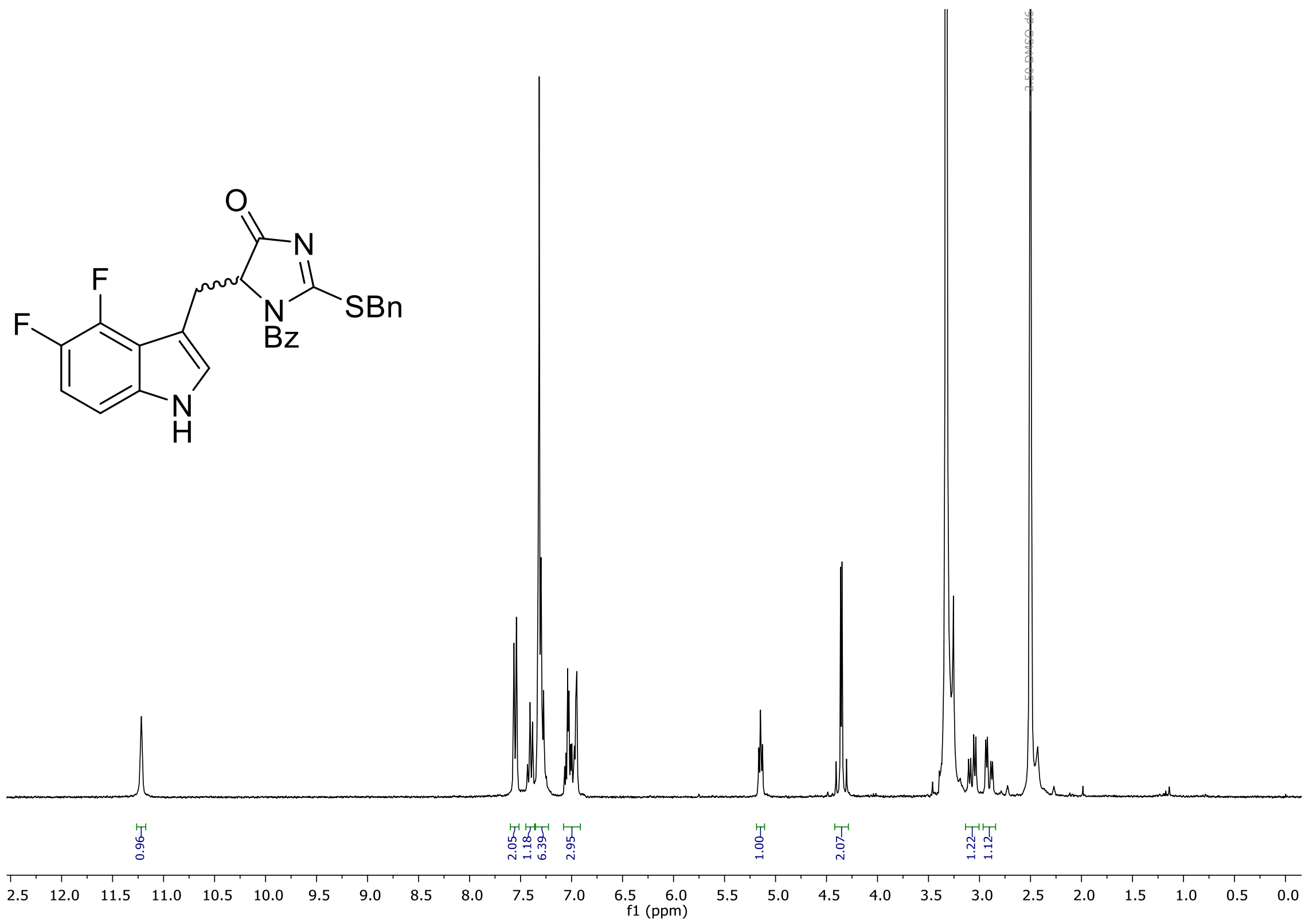

^{13}C{^{1}H} NMR (101 MHz, DMSO-d6) of **4a:**

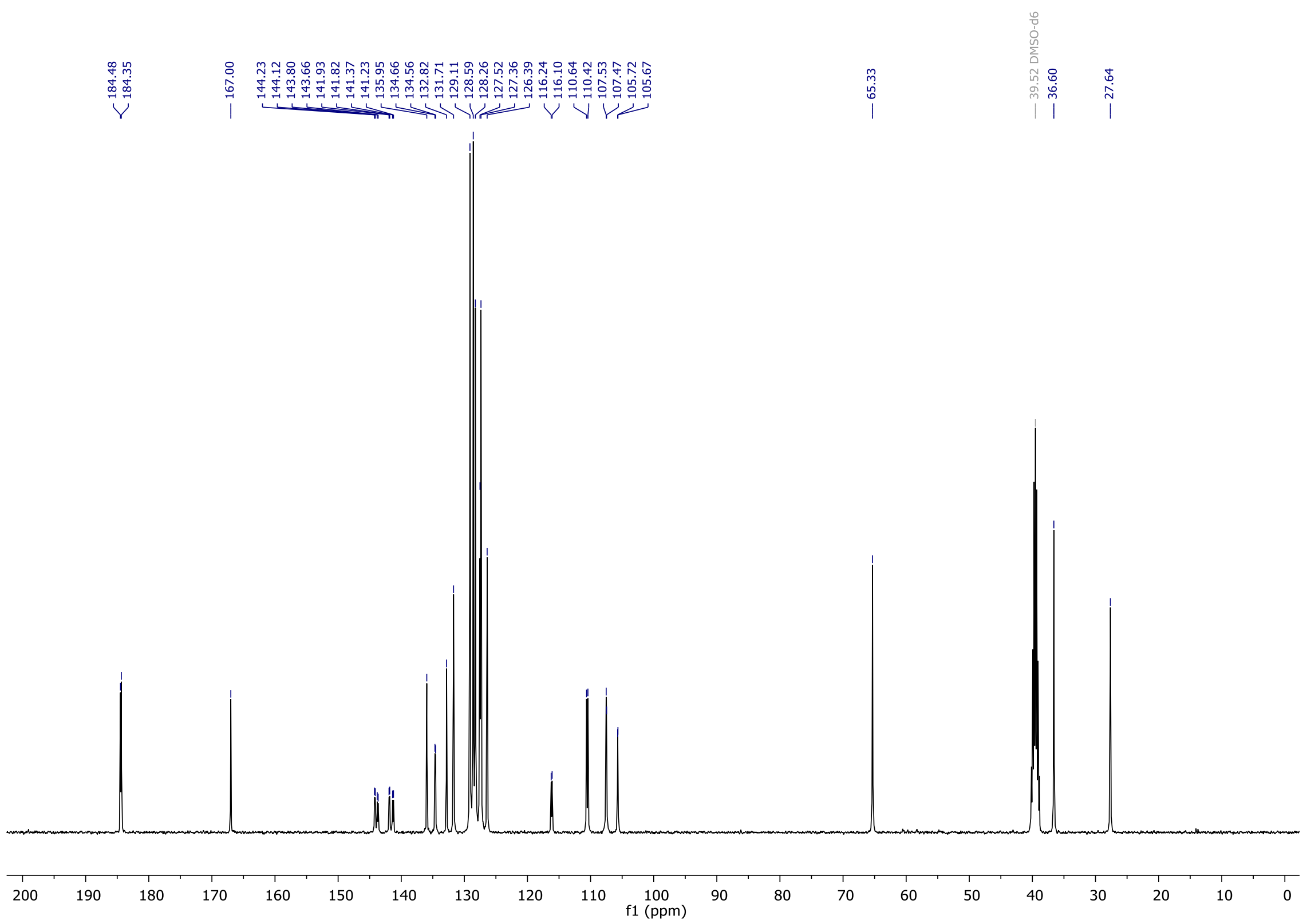

^{19}F NMR (376 MHz, DMSO-d$_6$) of **4a**:

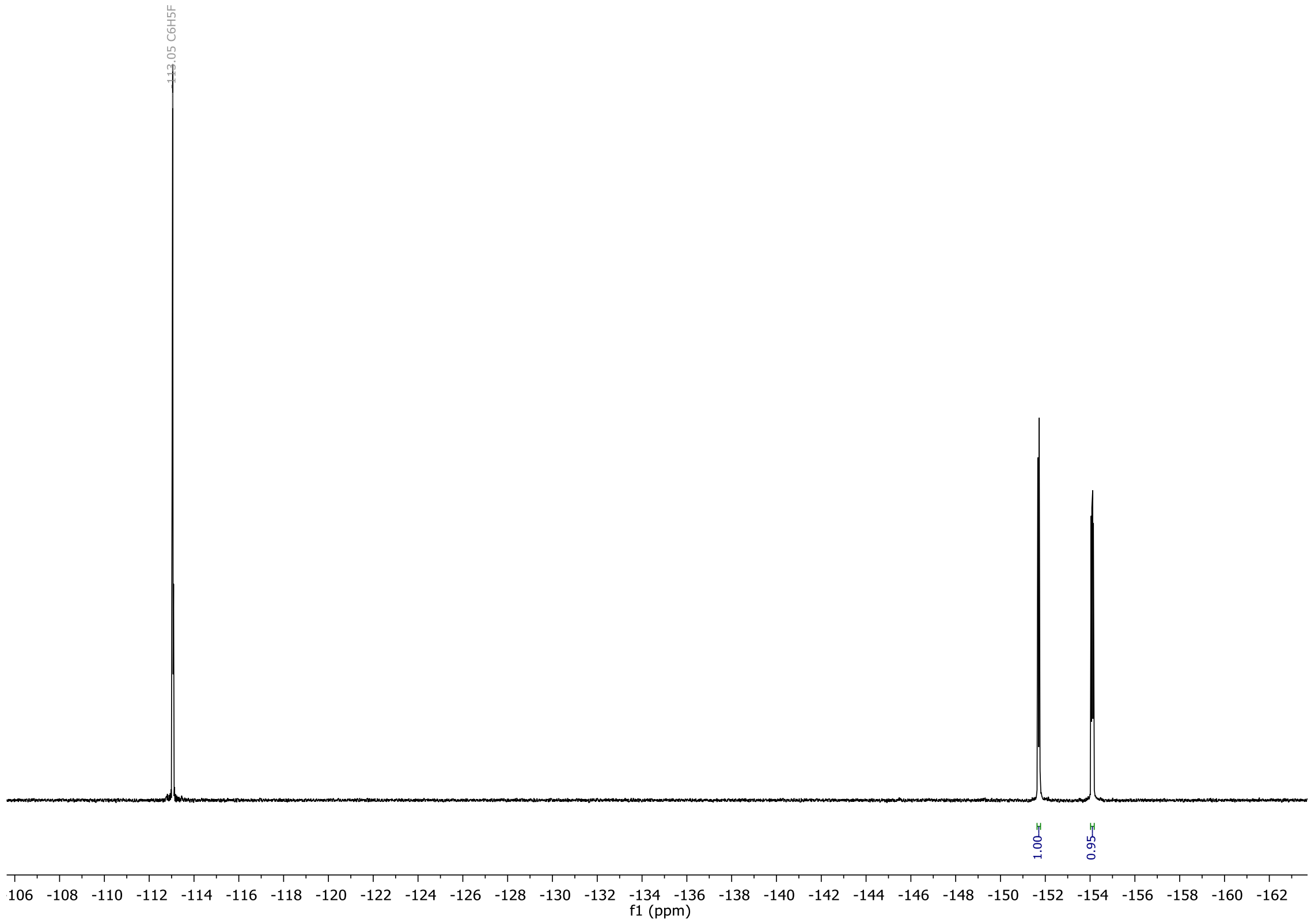

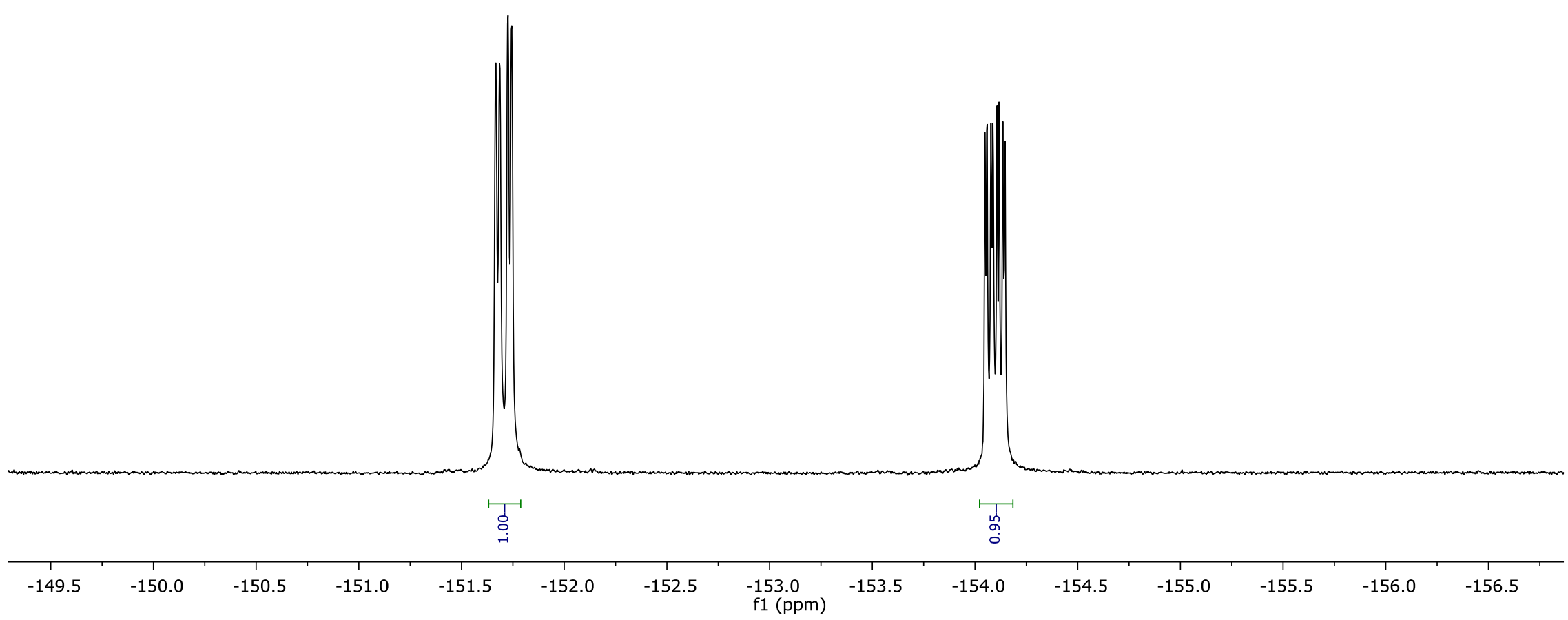

^{1}H NMR (300 MHz, $CDCl_3$) of **4b:**

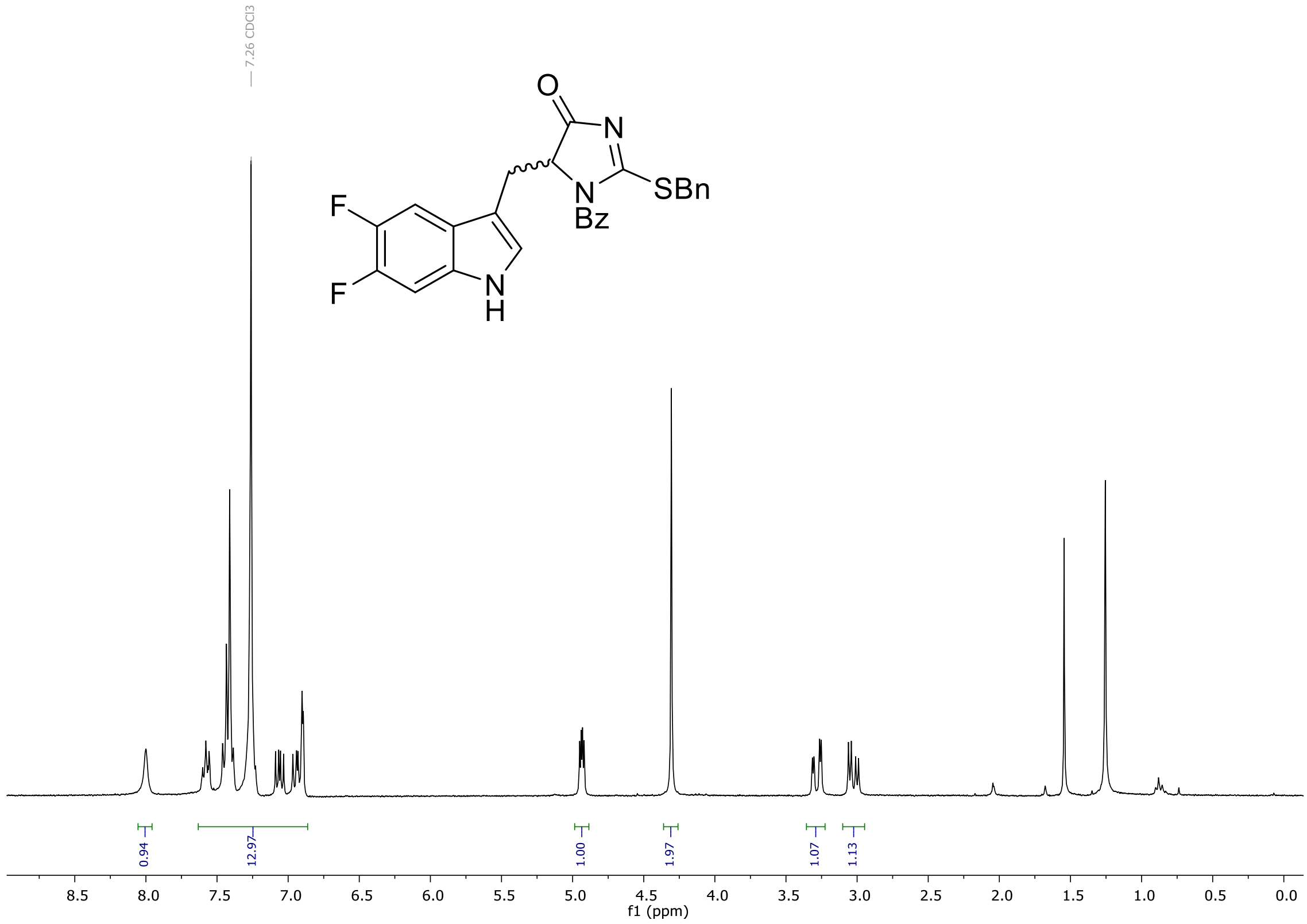

$^{13}C\{^{1}H\}$ NMR (126 MHz, DMSO-d6) of **4b**:

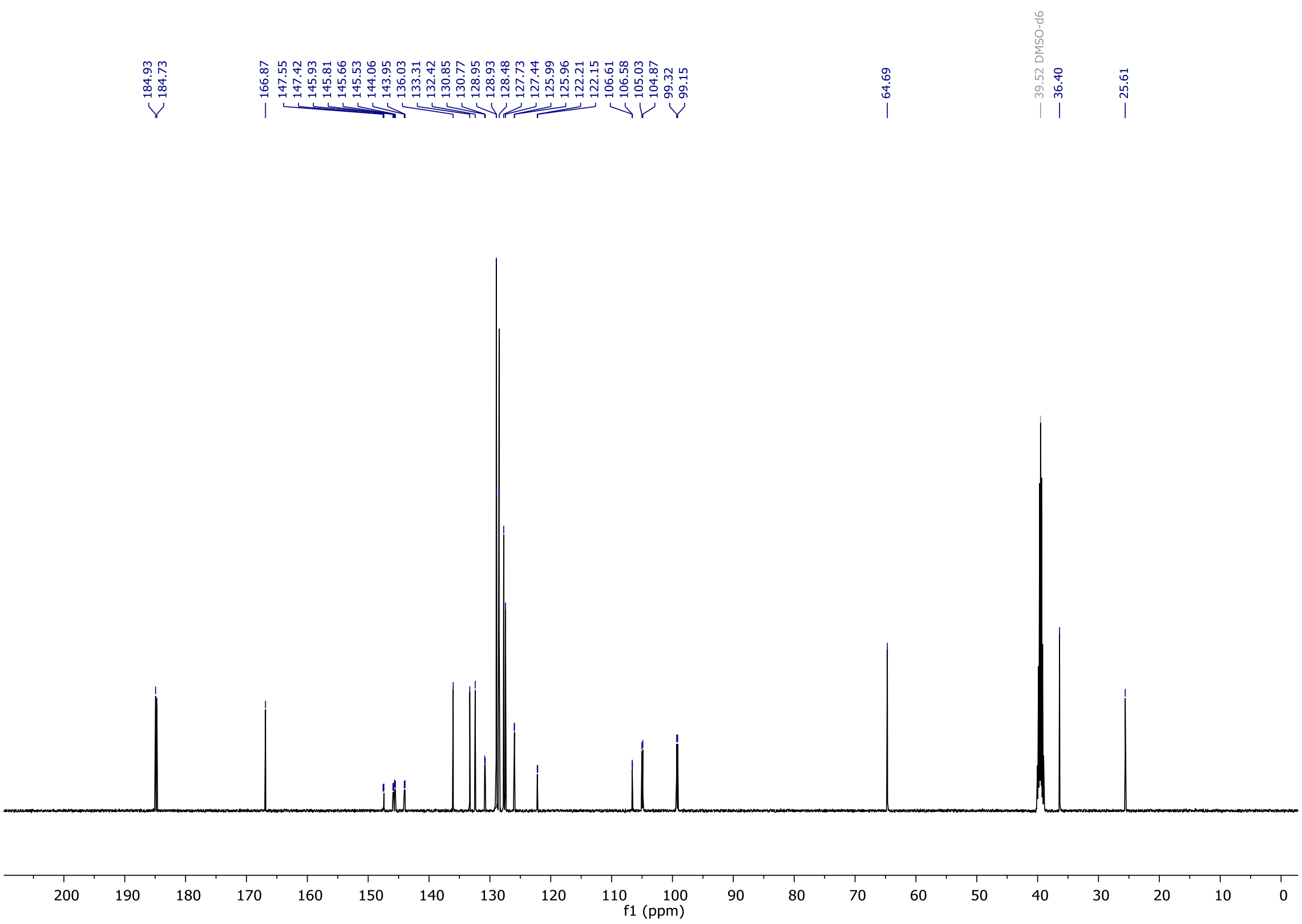

^{19}F NMR (376 MHz, DMSO-d$_6$) of **4b**:

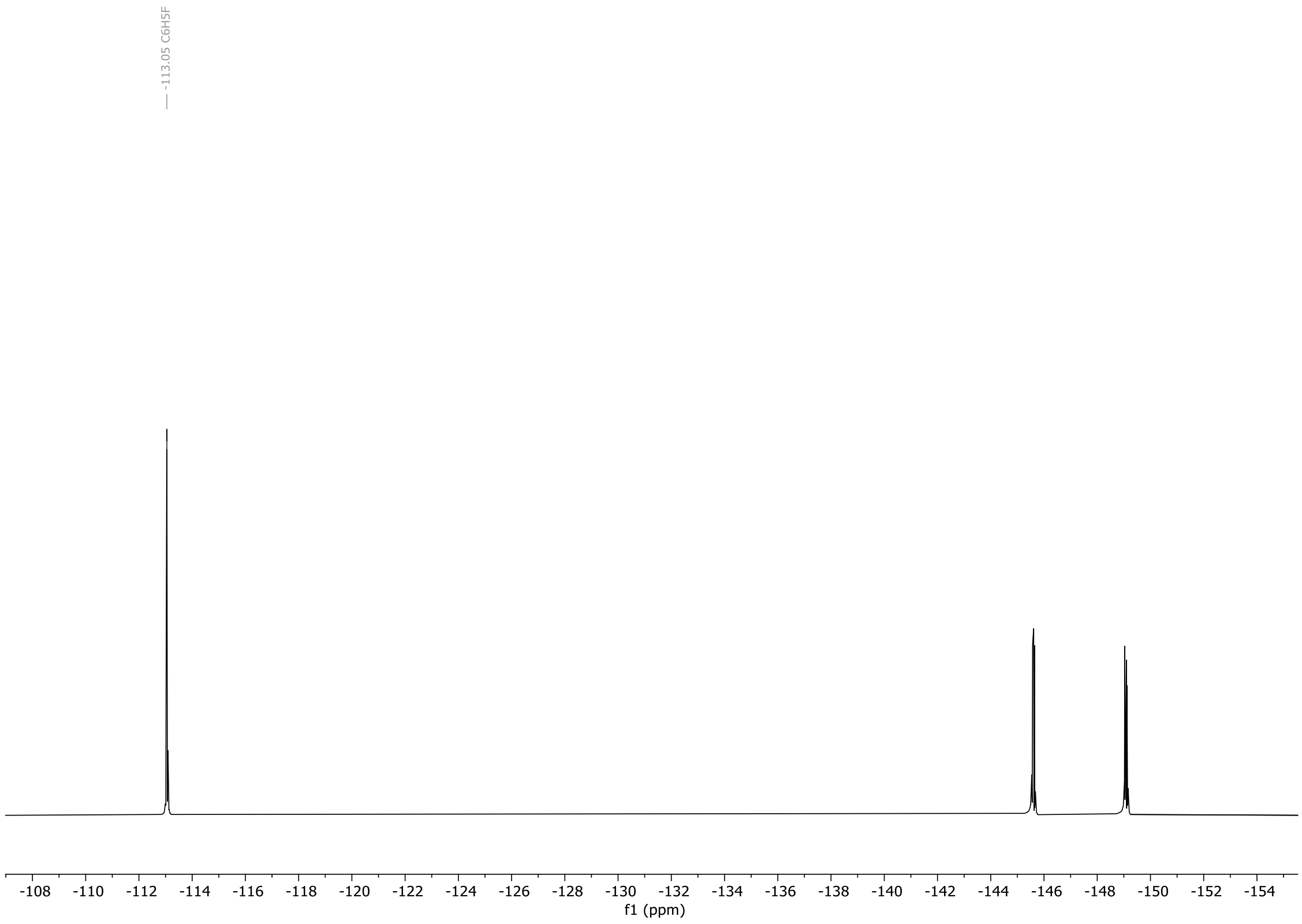

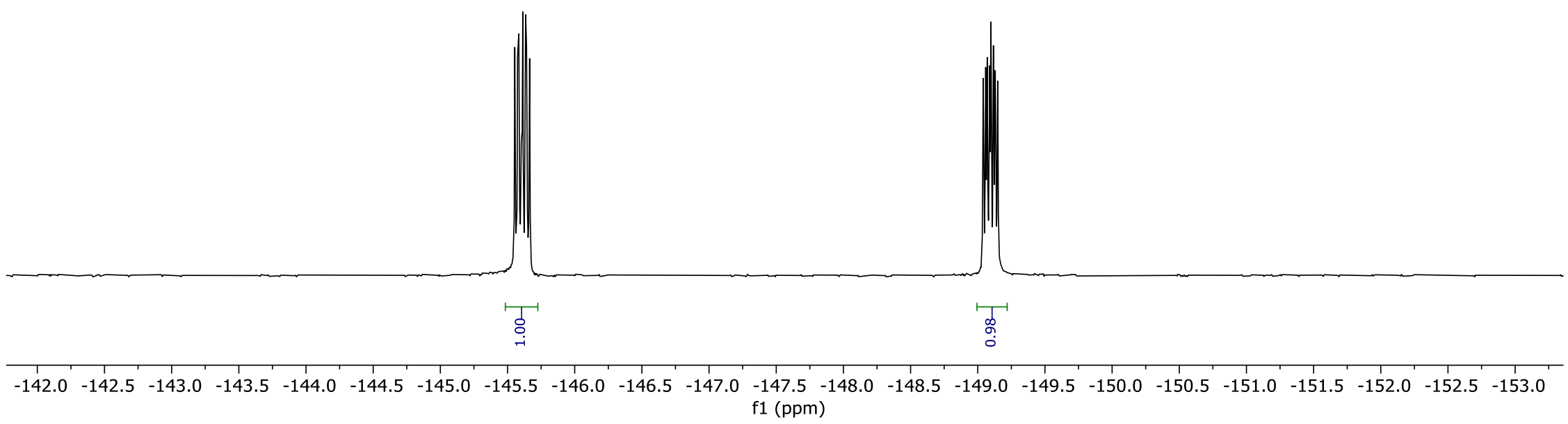

^{1}H NMR (300 MHz, $CDCl_3$) of **5a:**

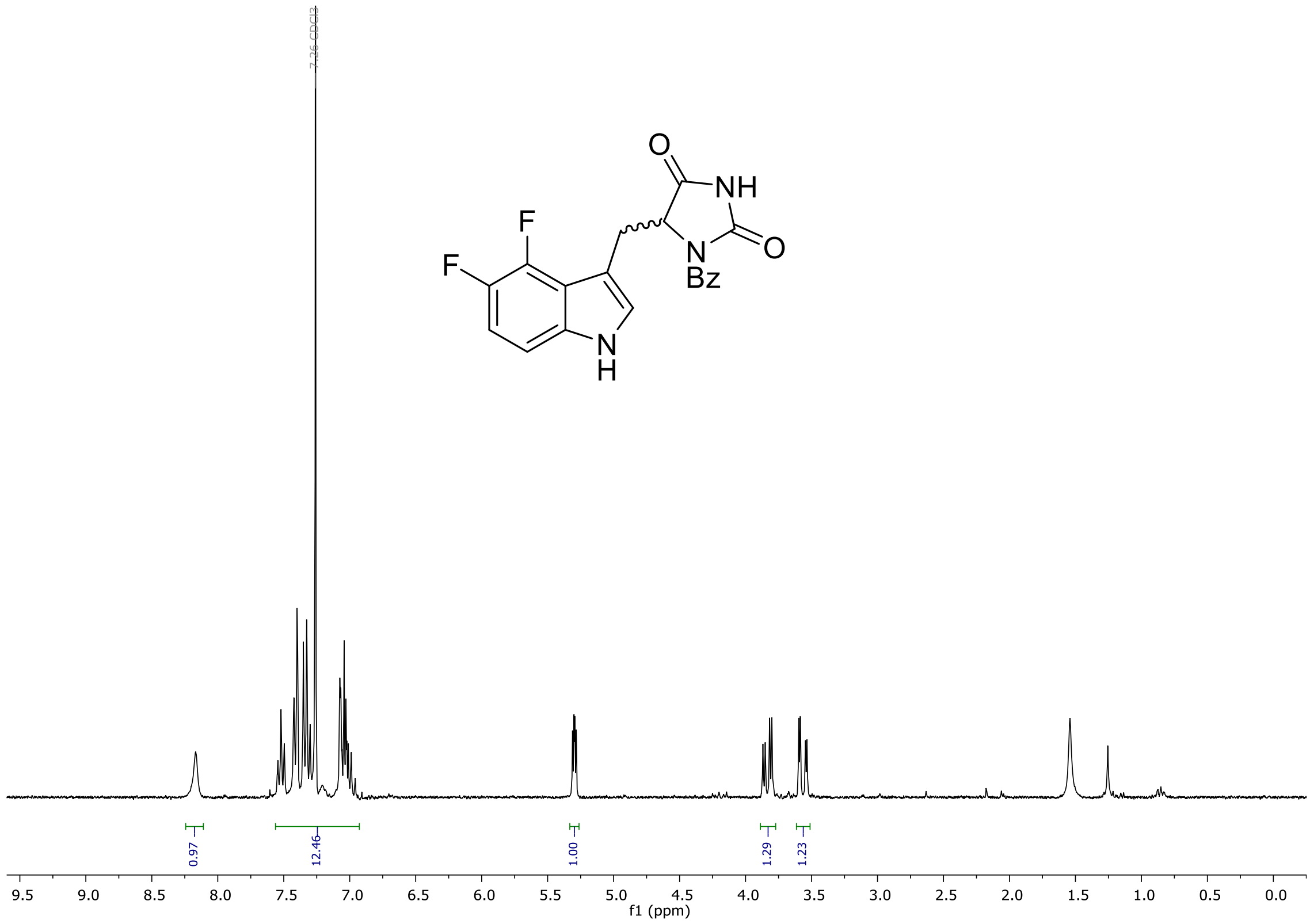

$^{13}C\{^{1}H\}$ NMR (126 MHz, DMSO-d6) of **5a**:

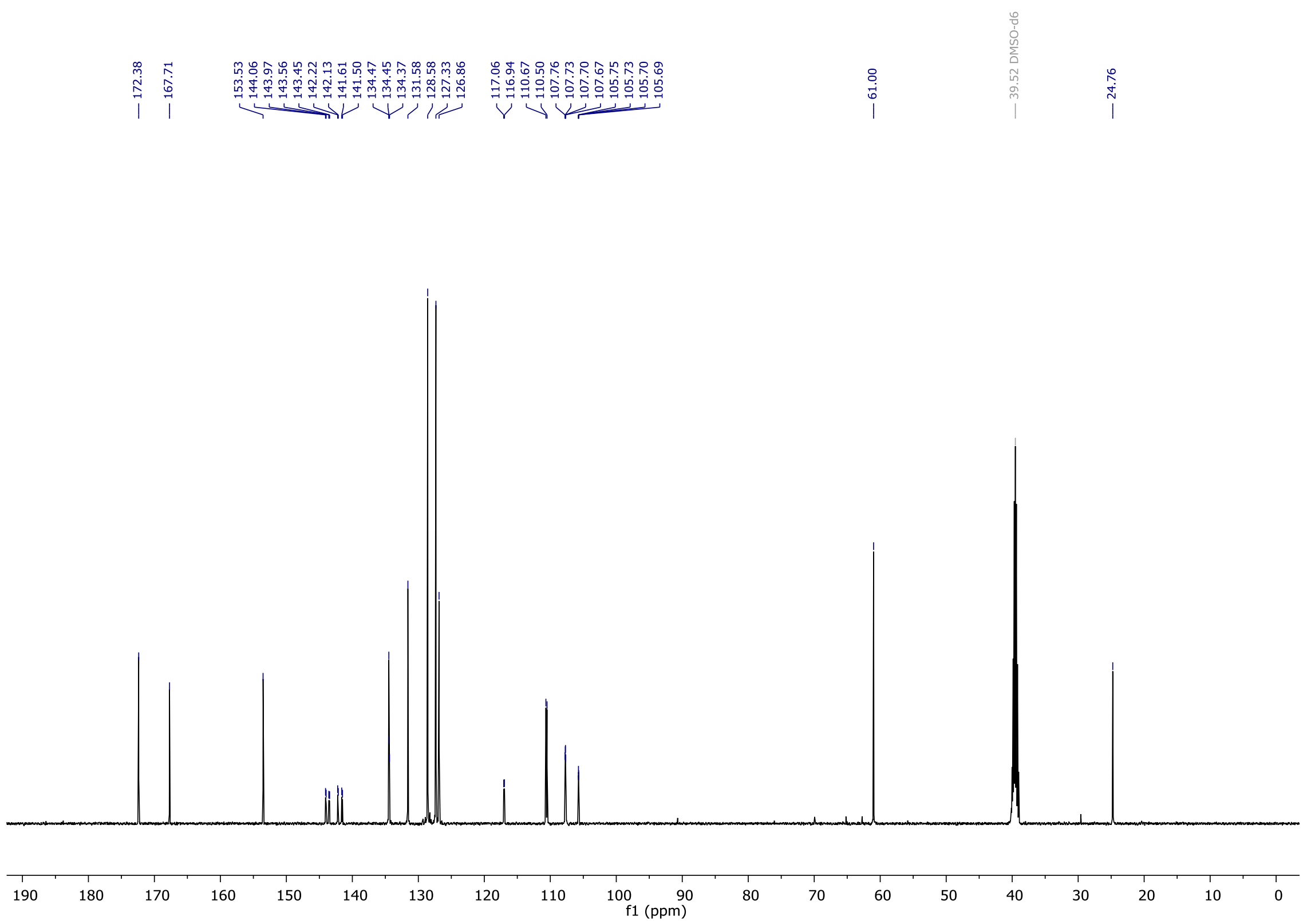

^{19}F NMR (376 MHz, DMSO-d$_6$) of **5a**:

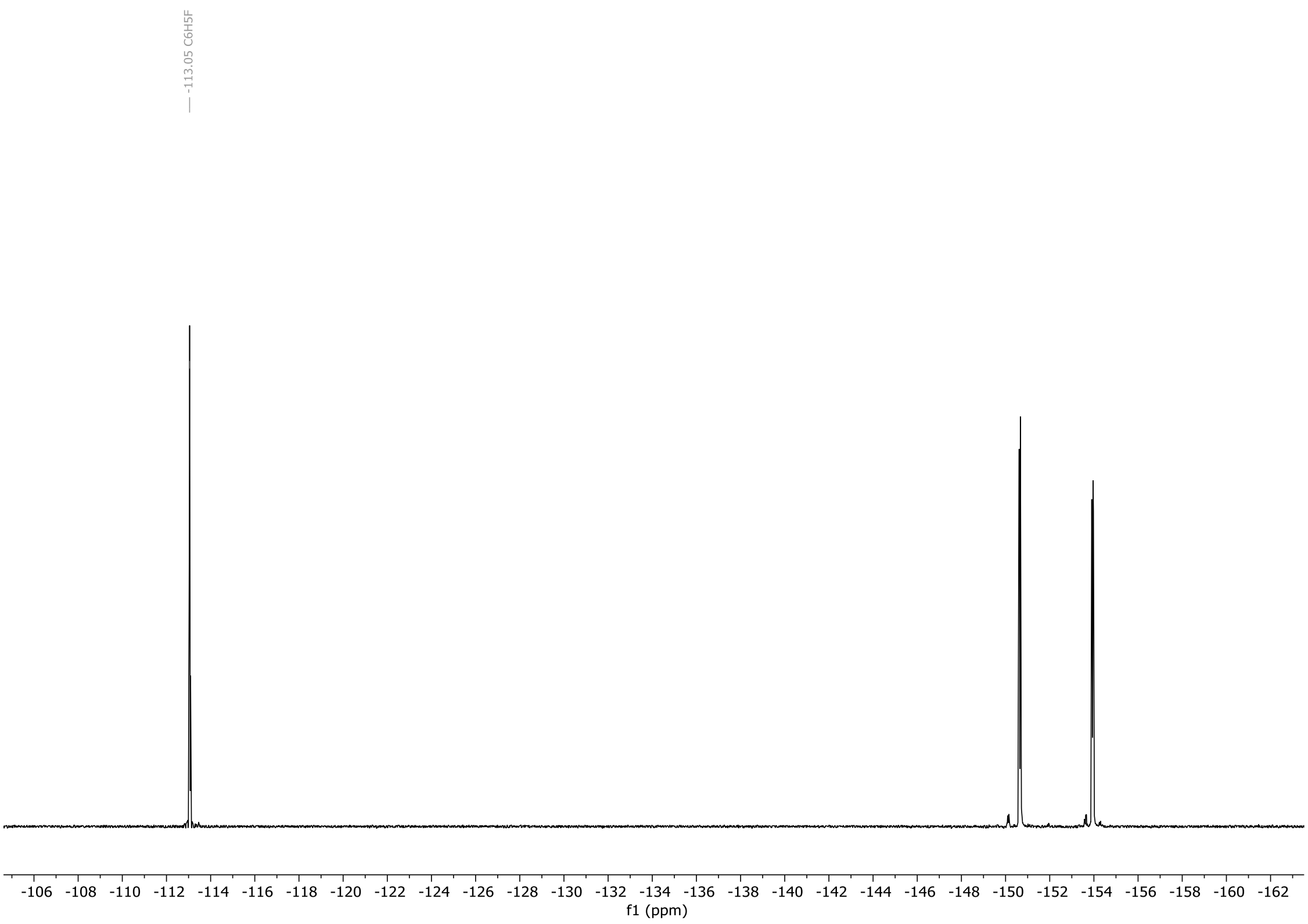

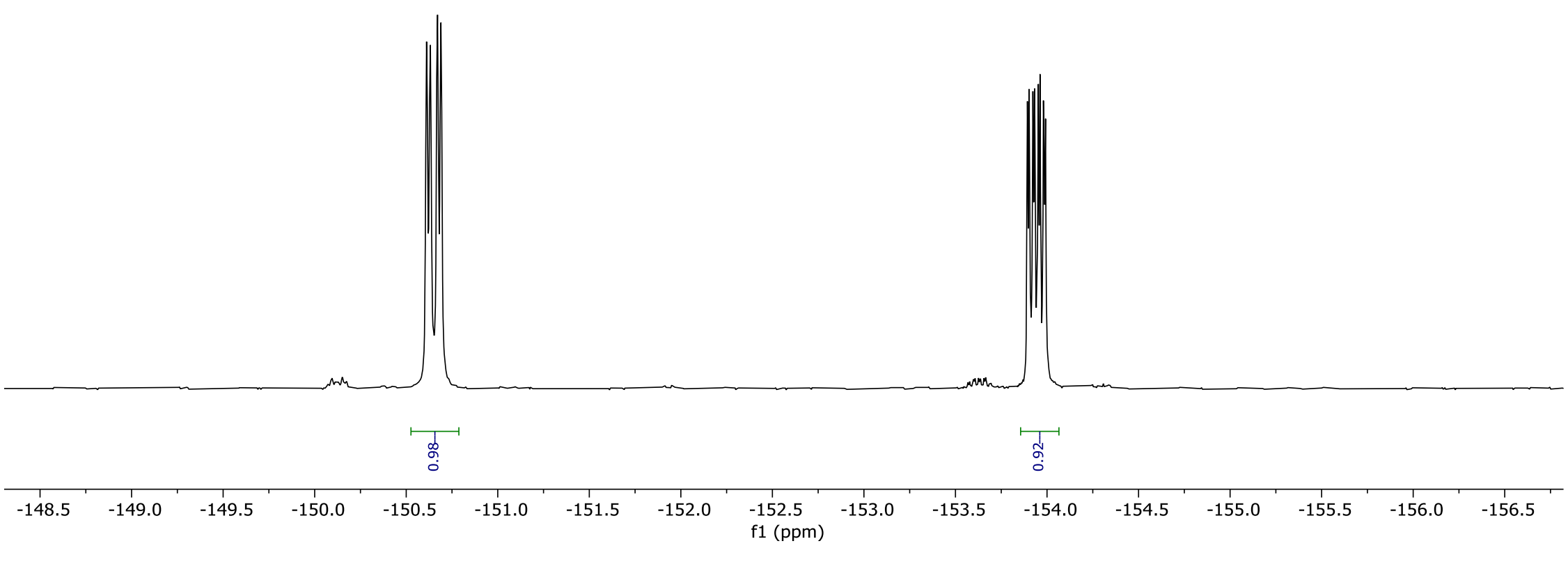

^{1}H NMR (300 MHz, CD_2Cl_2) of **5b:**

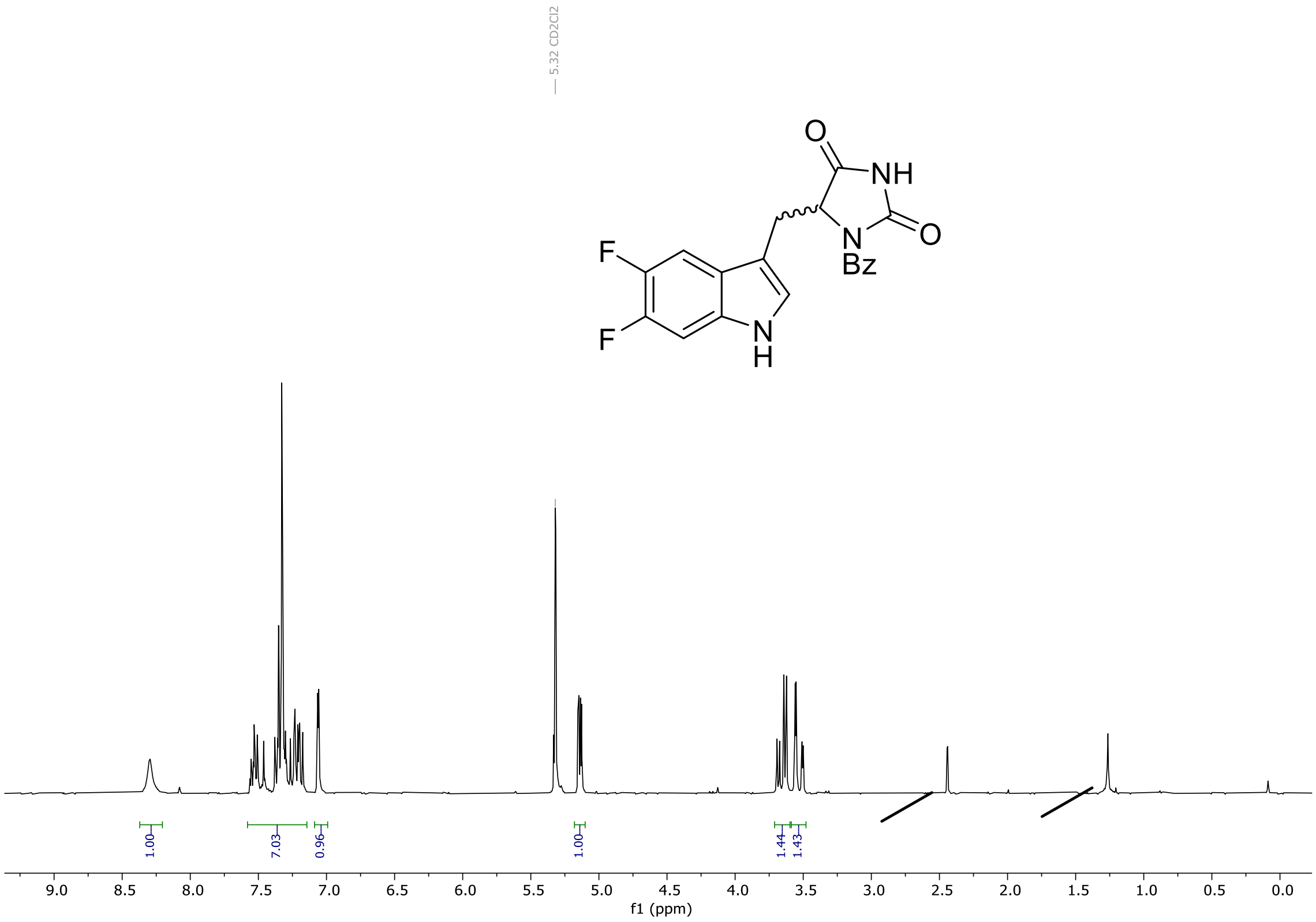

$^{13}C\{^{1}H\}$ NMR (101 MHz, DMSO-d_6) of **5b**:

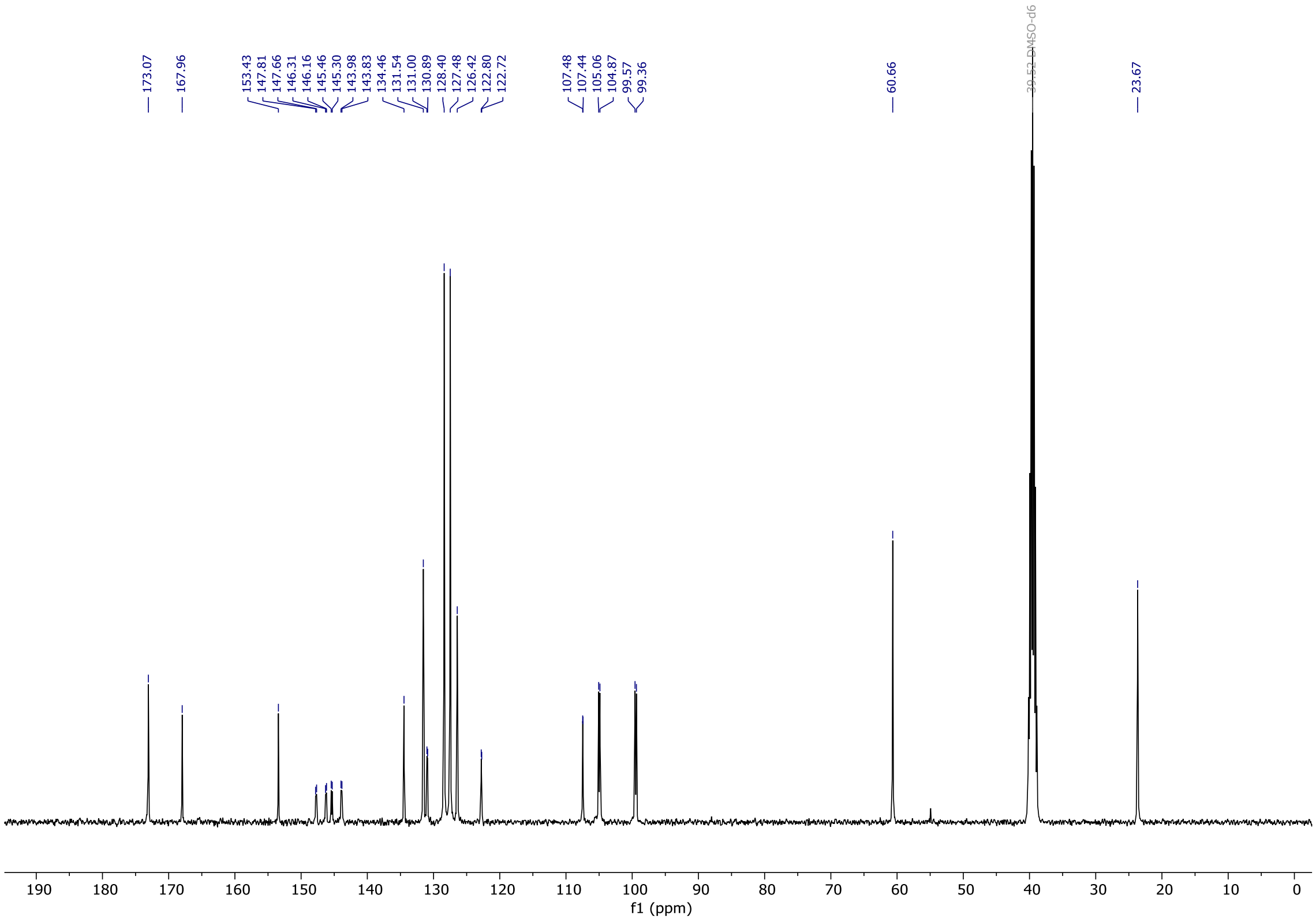

^{19}F NMR (376 MHz, DMSO-d_6) of **(5b)**:

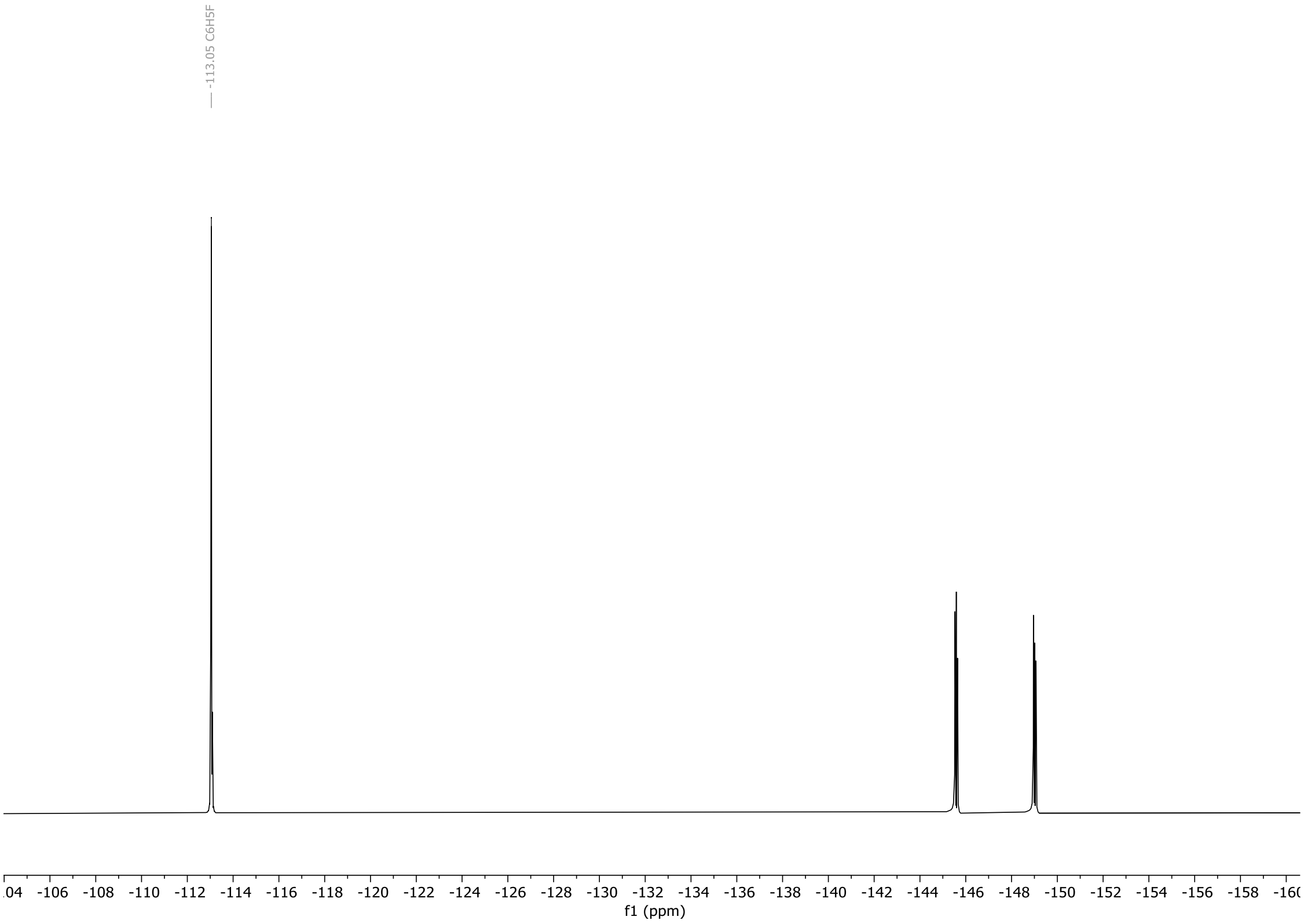

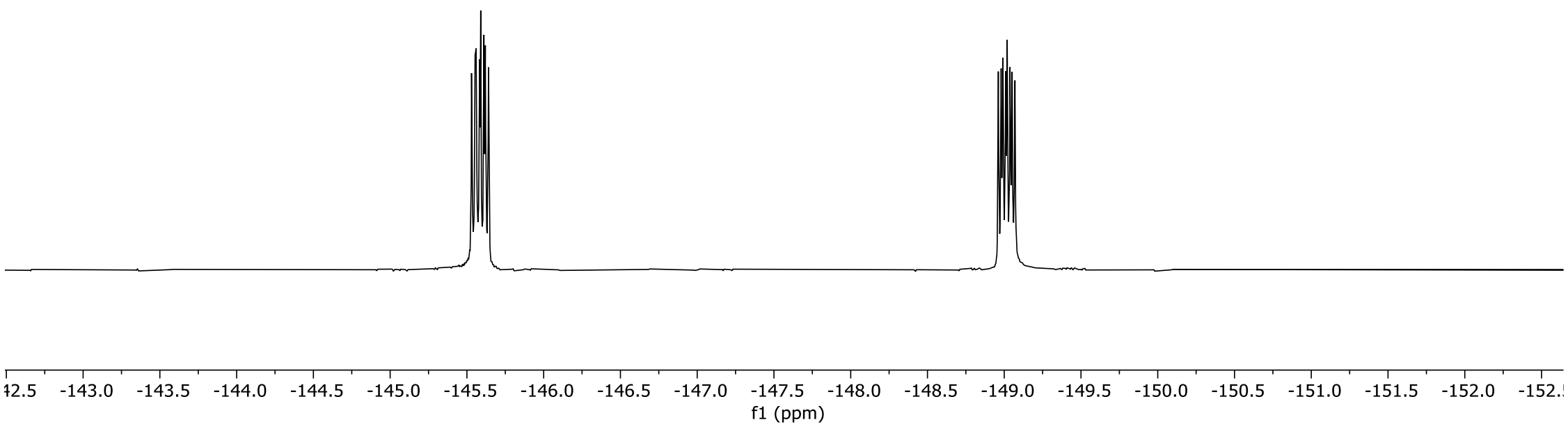

^{1}H NMR (300 MHz, D_2O) of **6a:**

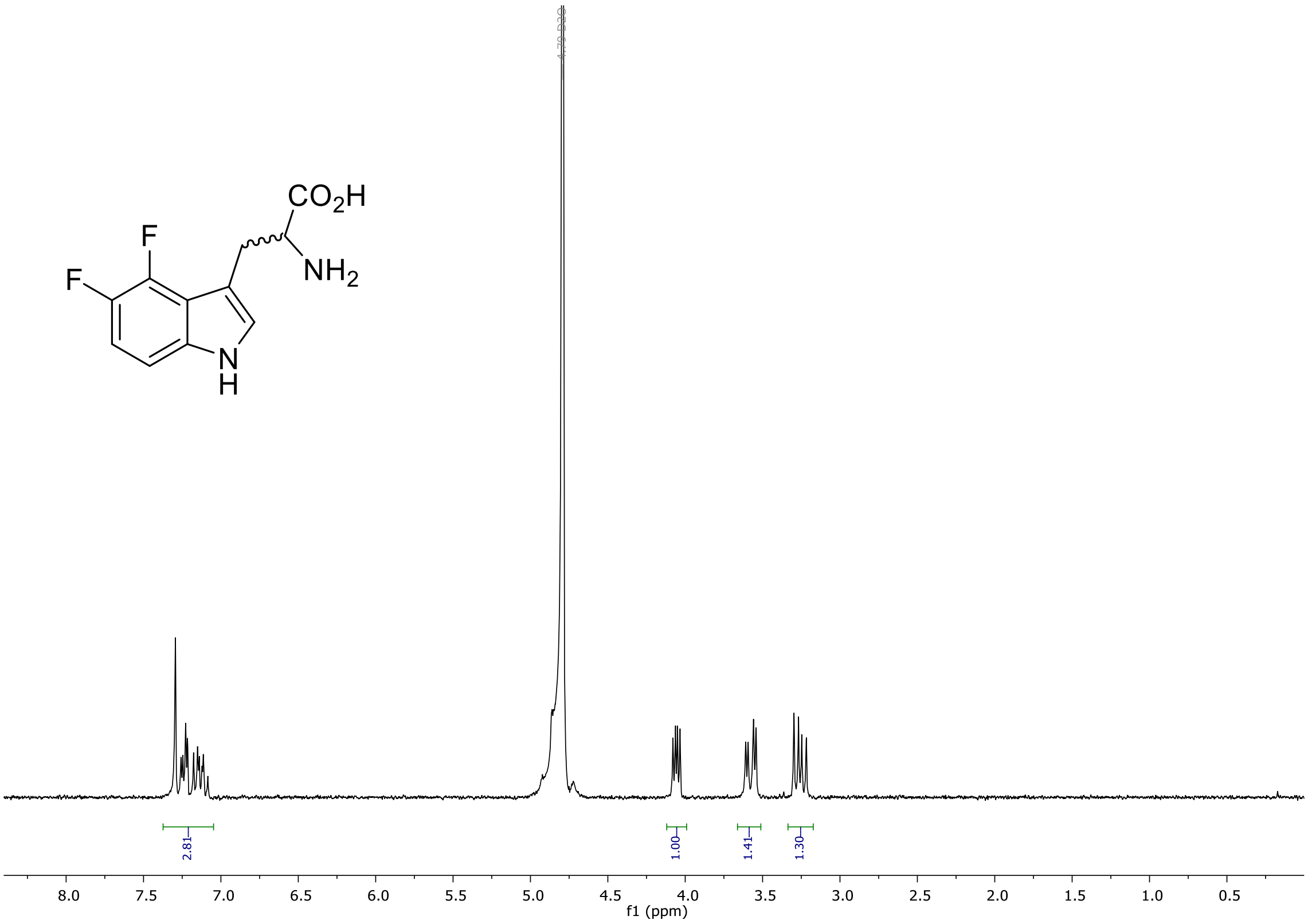

$^{13}C\{^{1}H\}$ NMR (126 MHz, CD_3OD) of **6a**:

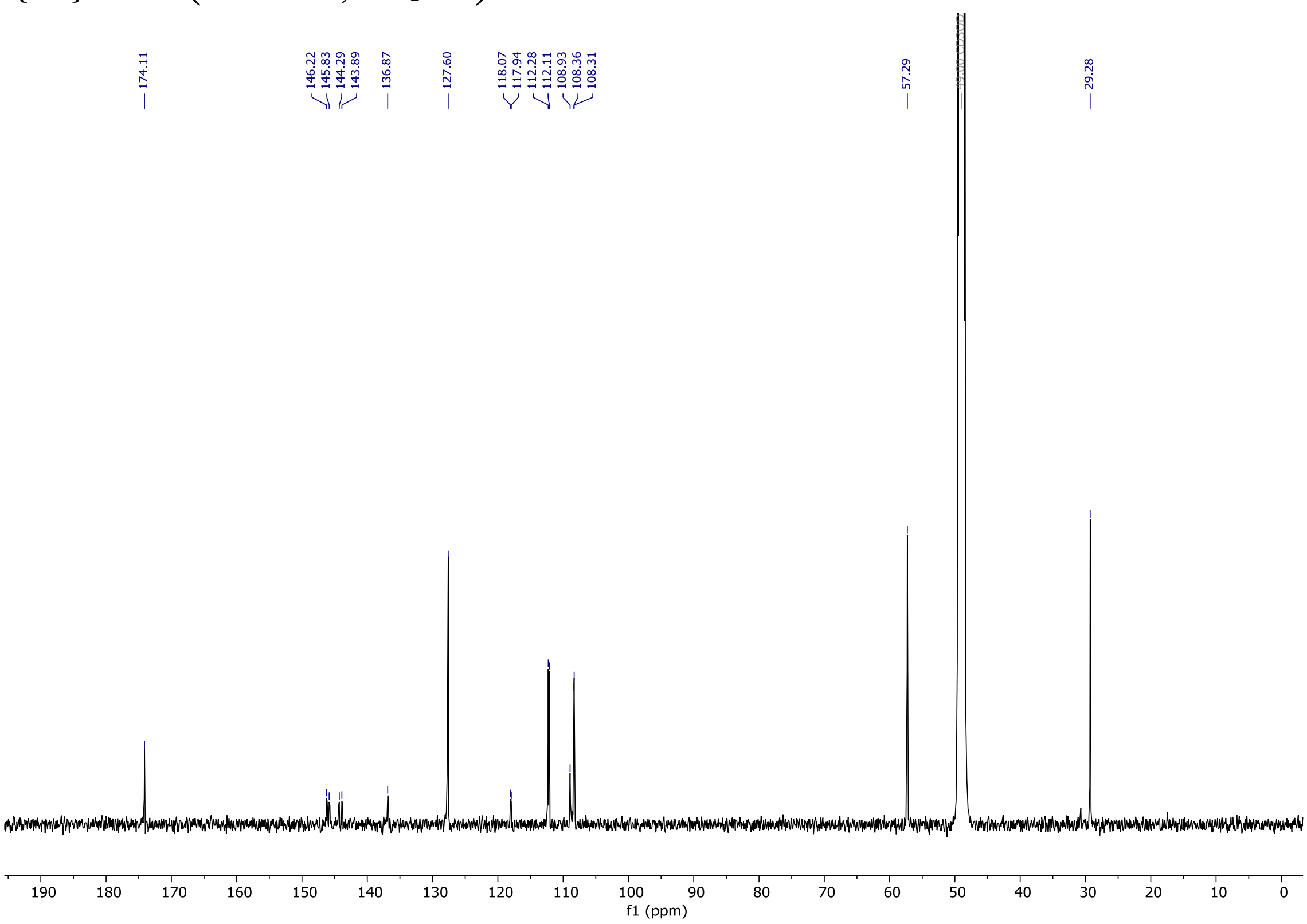

^{1}H-^{13}C HSQC NMR (600 MHz - 151 MHz, CD_3OD) of **6a:**

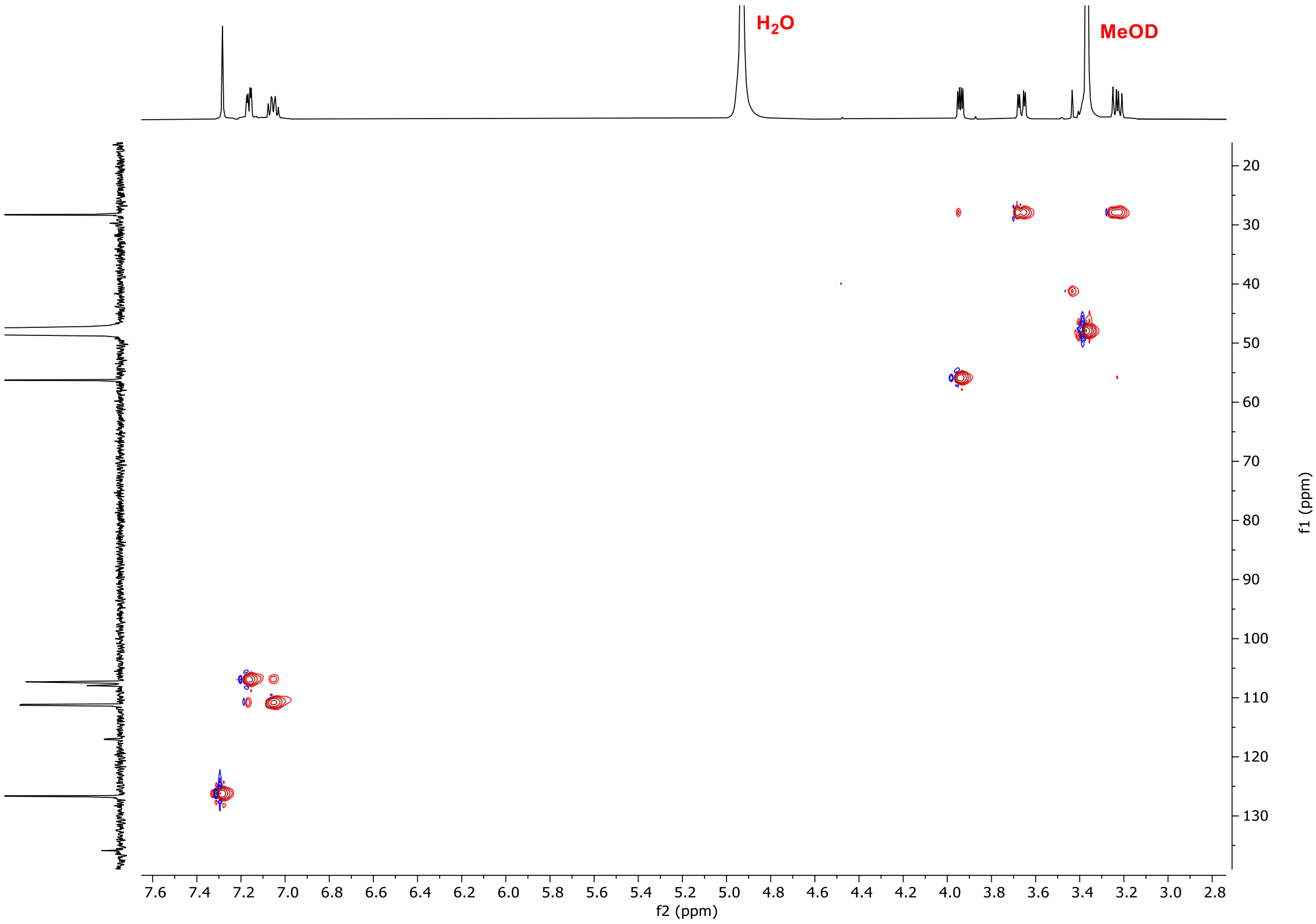

^{1}H-^{13}C HMBC NMR (600 MHz - 151 MHz, CD_3OD) of **6a**:

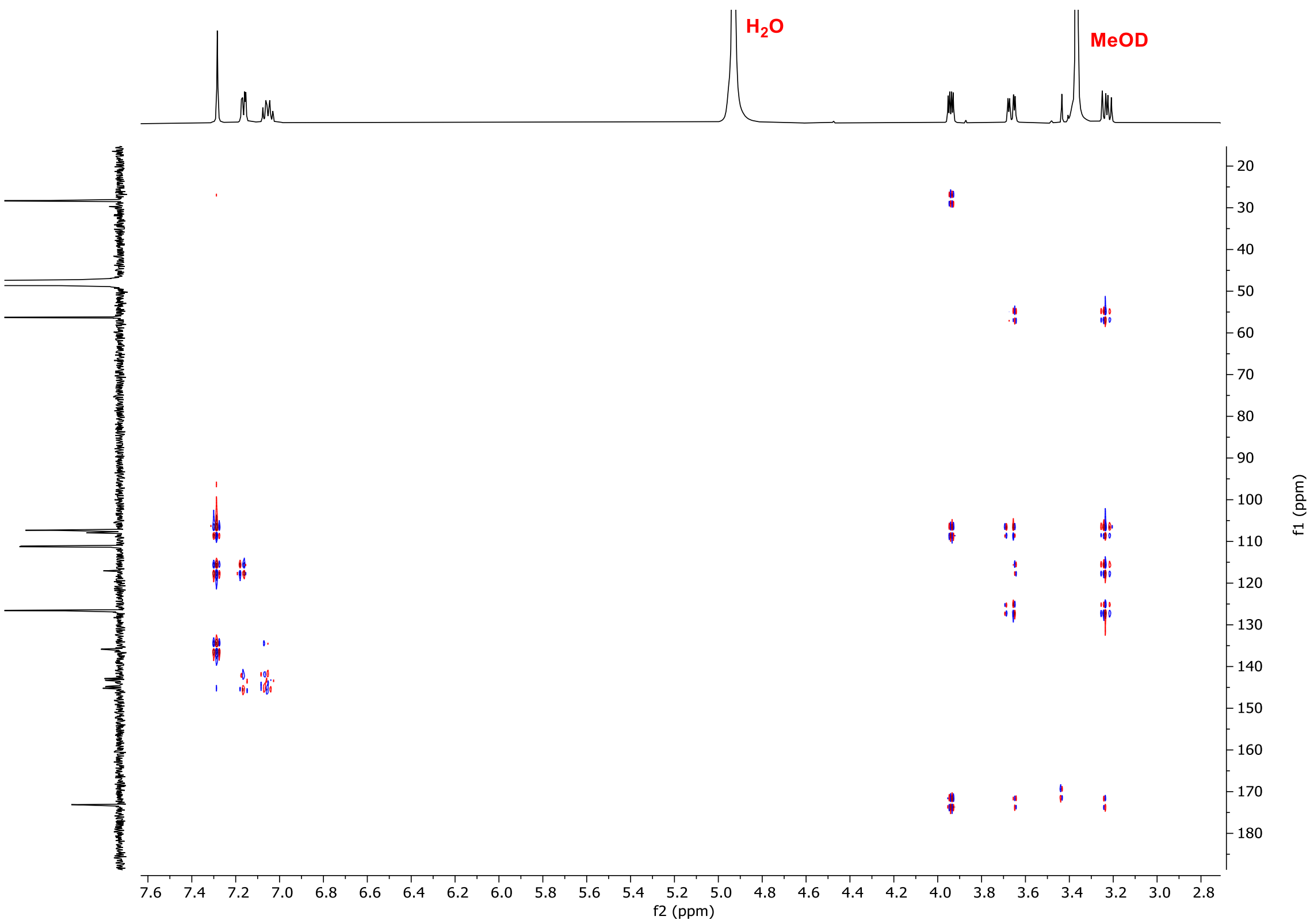

^{19}F NMR (376 MHz, DMSO-d6) of **6a**:

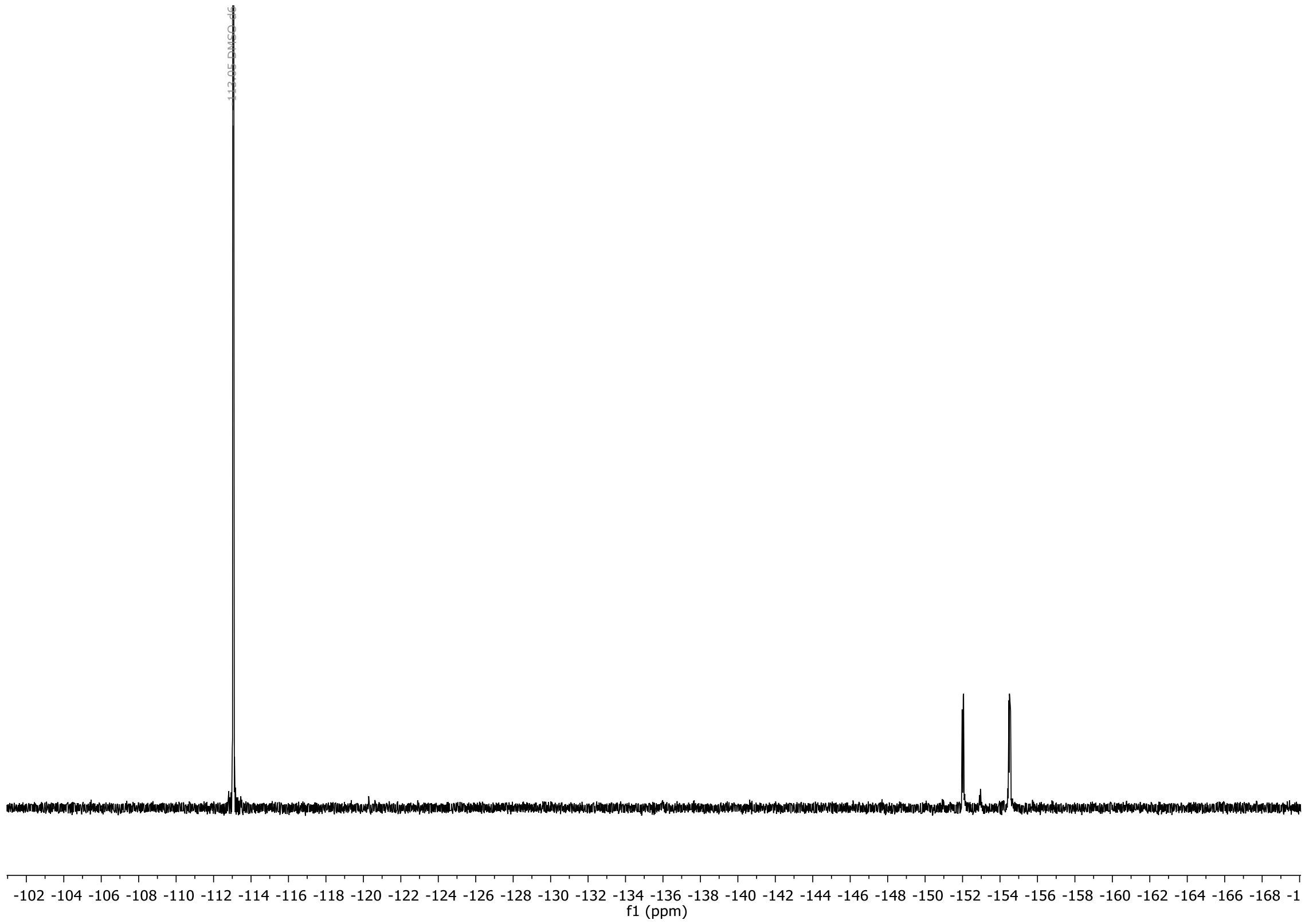

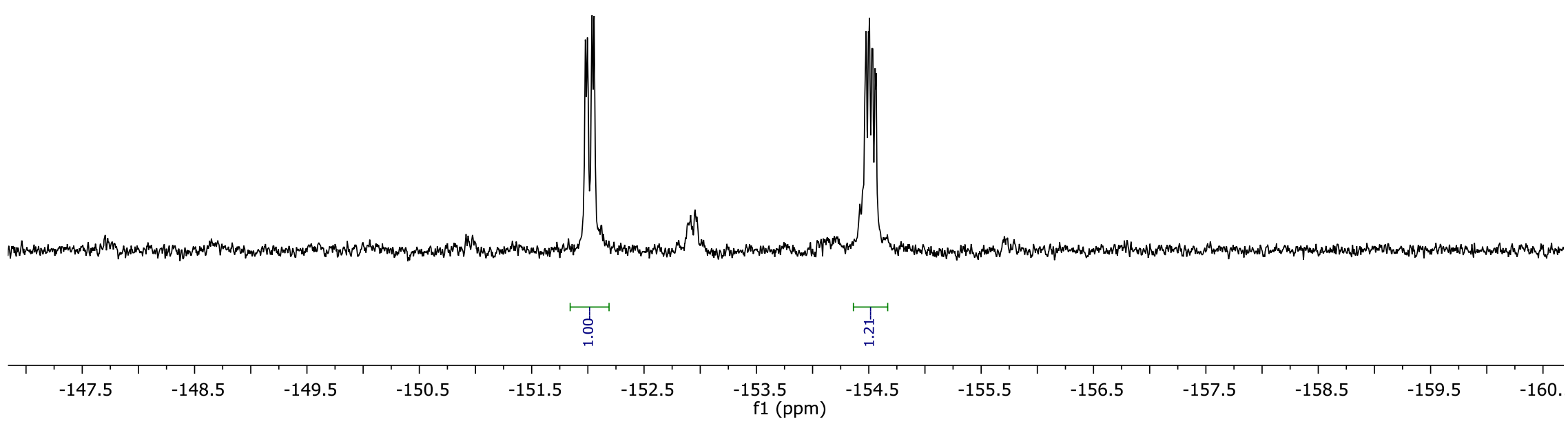

^{1}H NMR (300 MHz, D_2O) of **6b:**

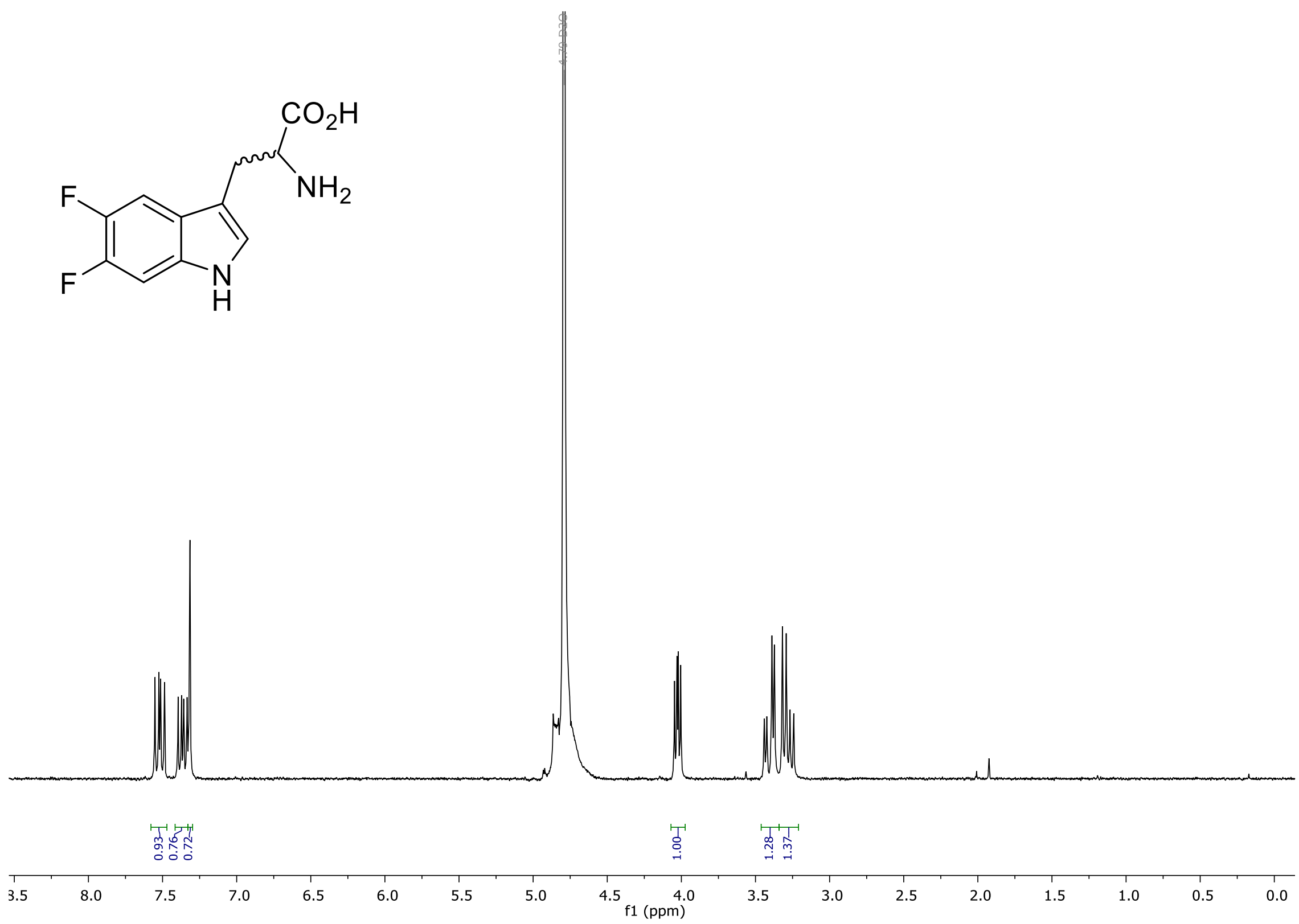

^{13}C{^{1}H} NMR (151 MHz, CD_3OD) of **6b**:

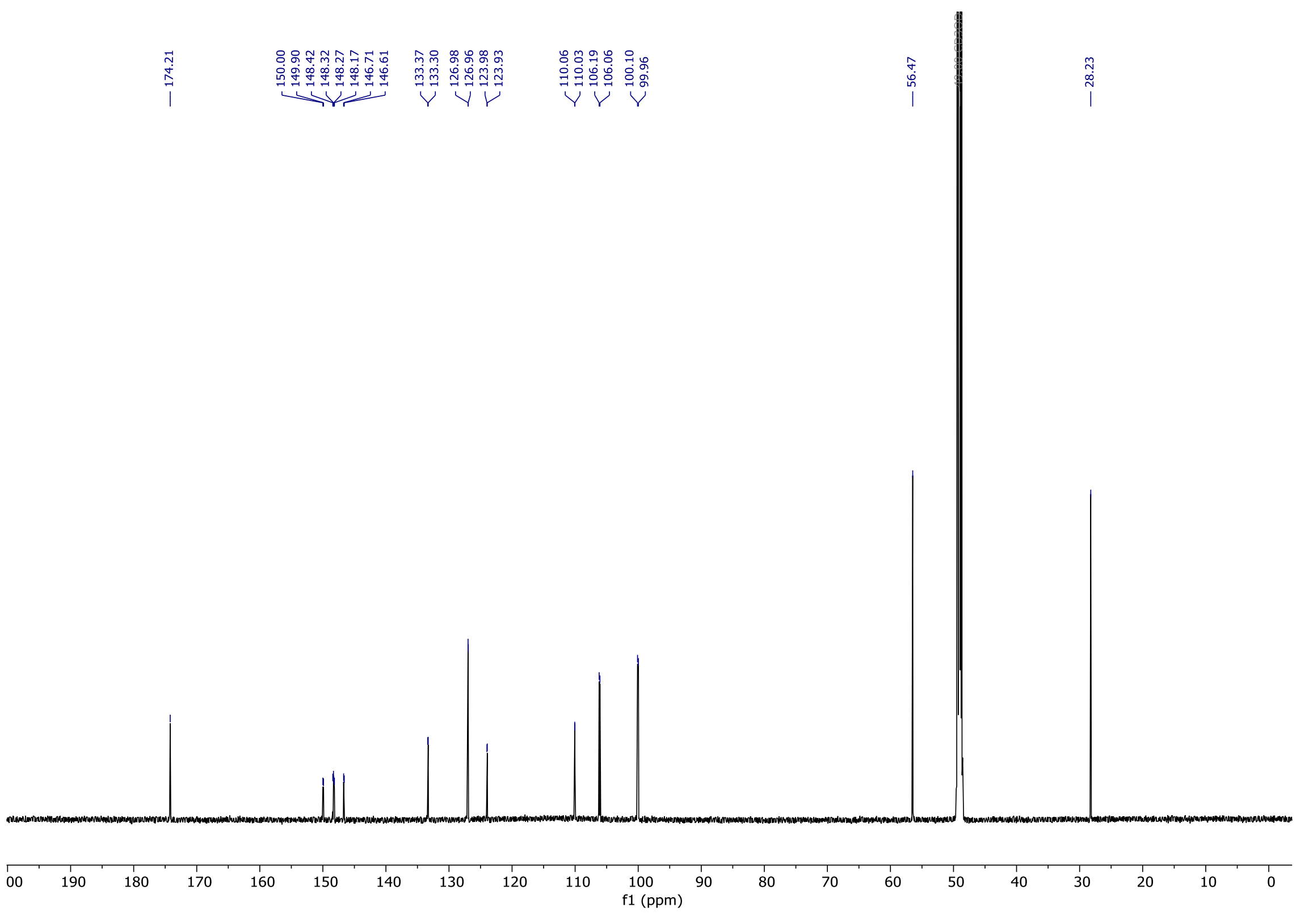

^{1}H-^{13}C HSQC NMR (600 MHz - 151 MHz, CD_3OD) of **6b:**

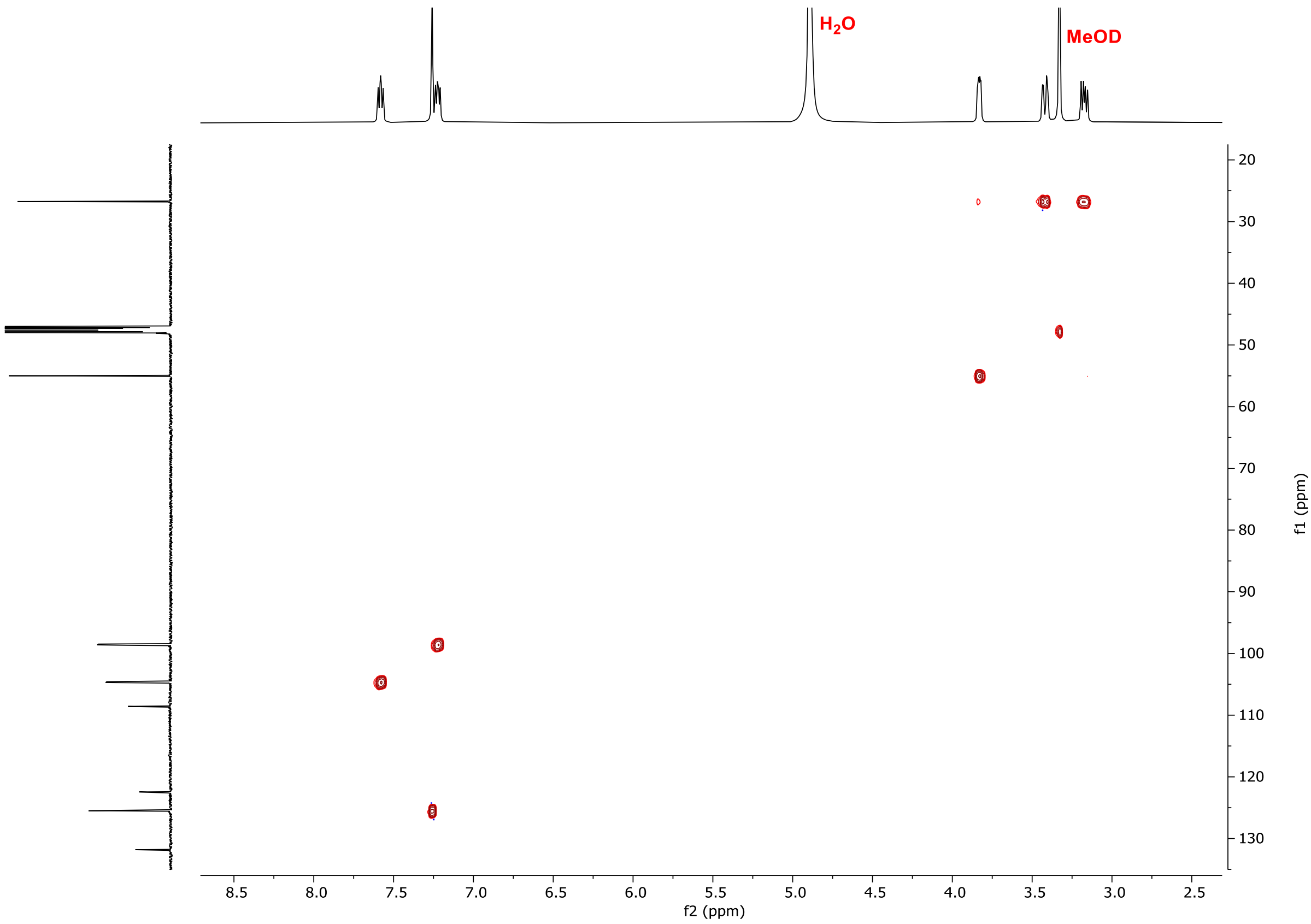

^{1}H-^{13}C HMBC NMR (600 MHz - 151 MHz, CD_3OD) of **6b**:

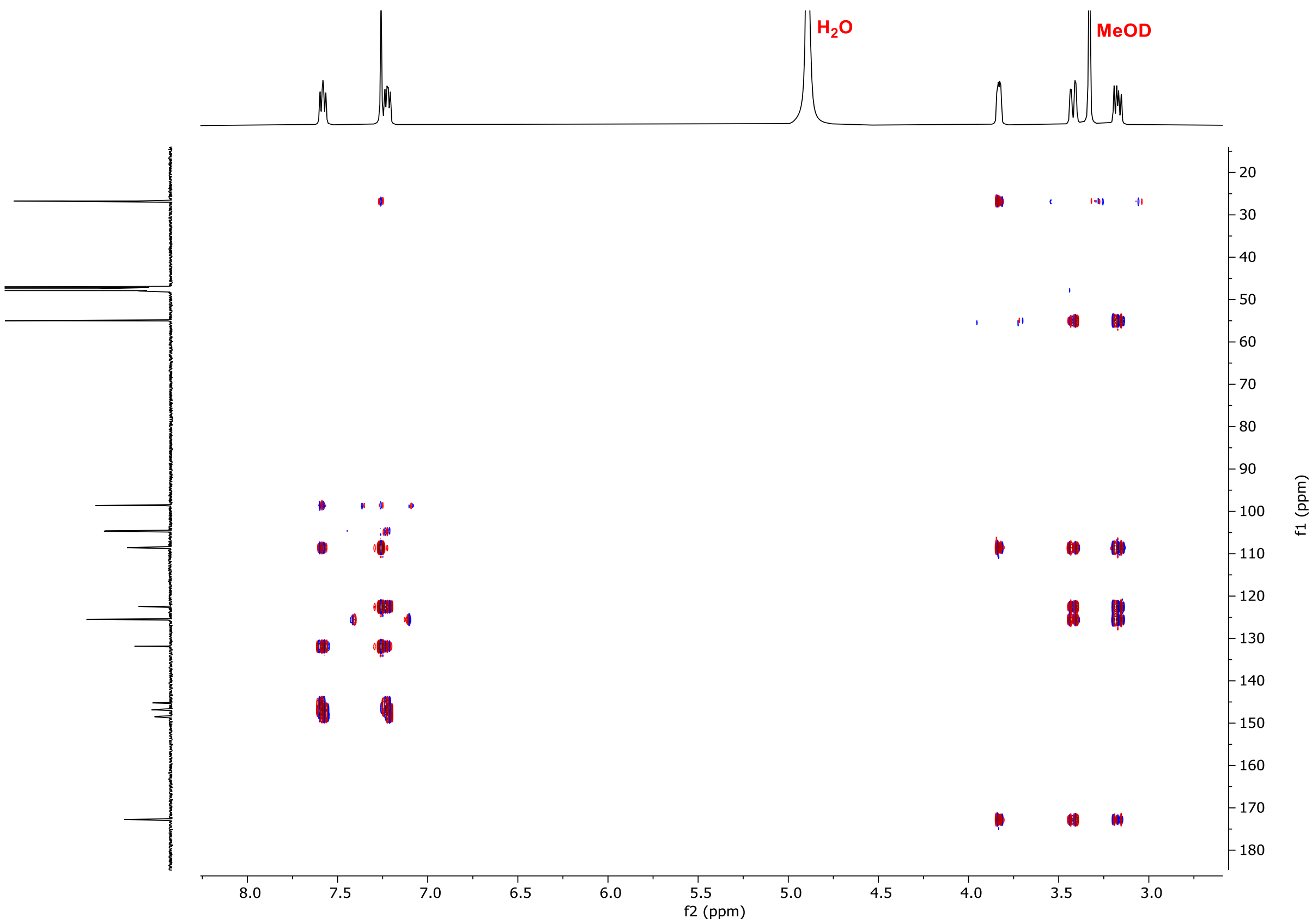

^{19}F NMR (376 MHz, DMSO-d$_6$) of **6b**:

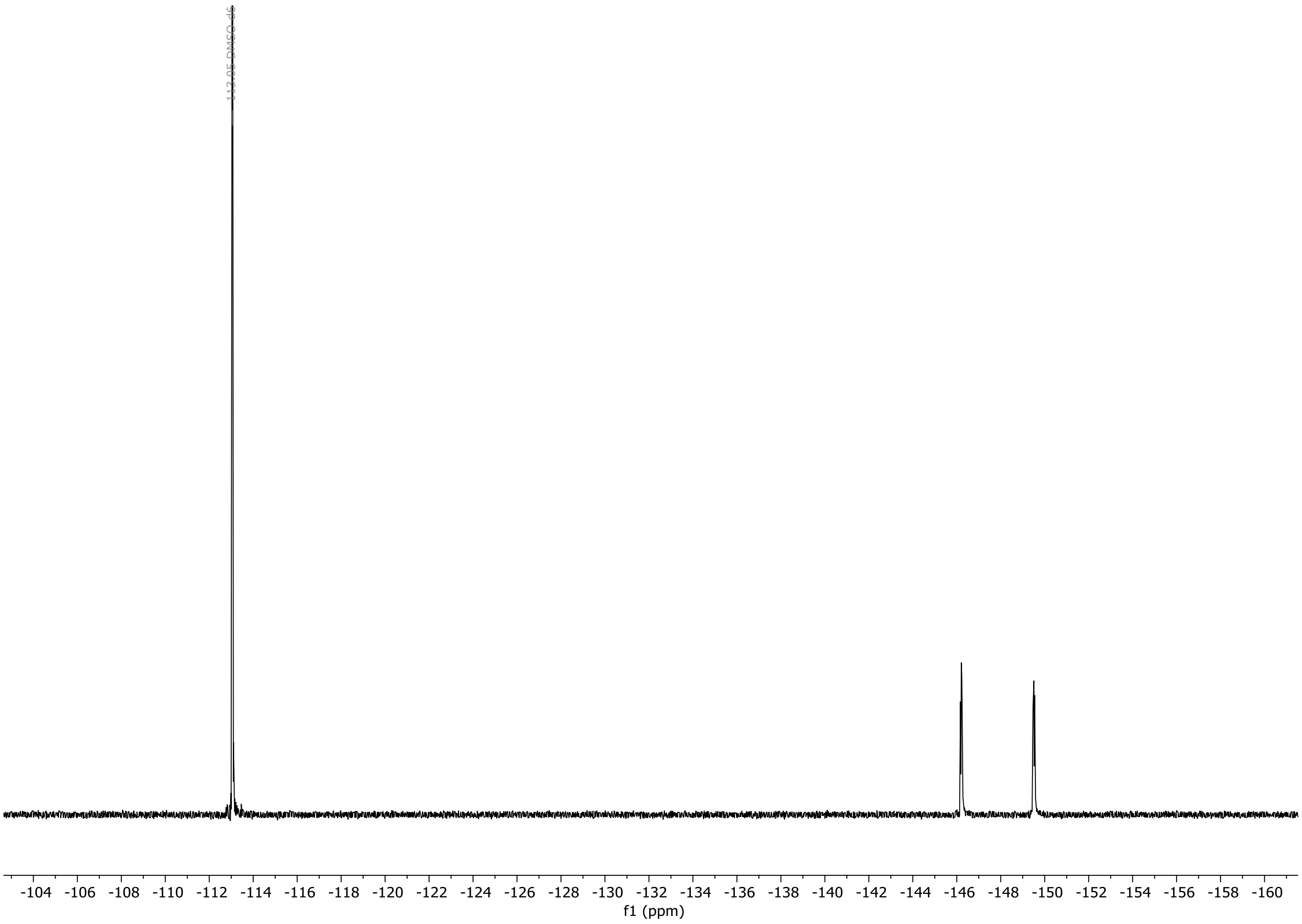

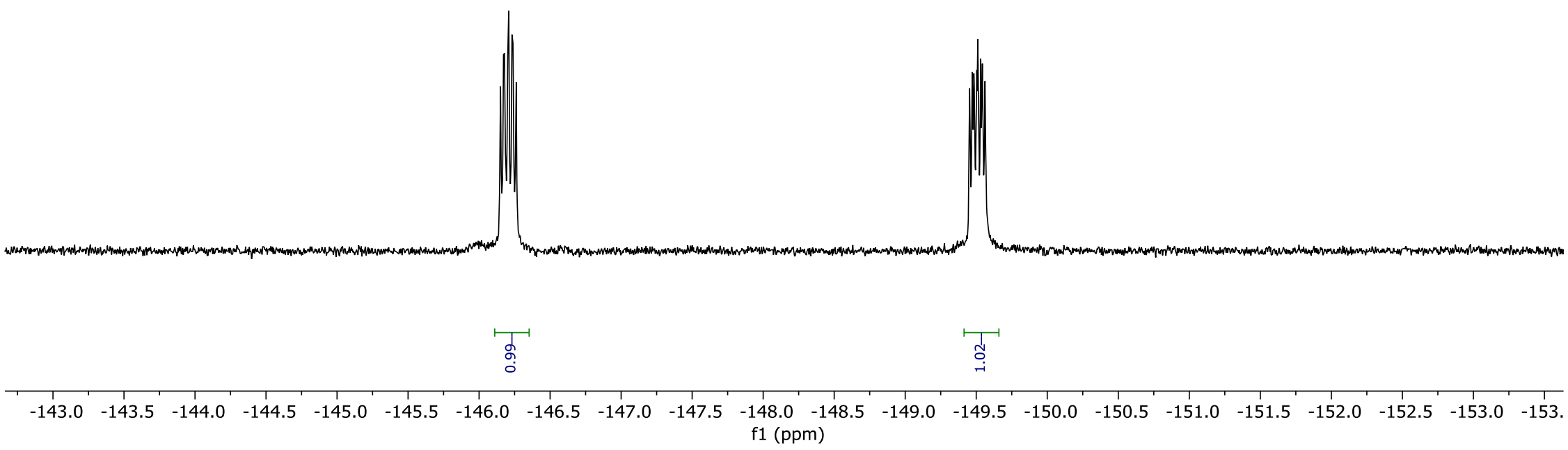

7. High Resolution Mass Spectra (HRMS)

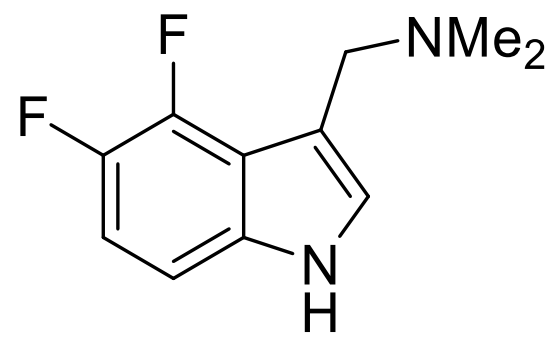

1a

HRMS (ESI) m/z: $[M+H]^+$ Calcd. for $C_{11}H_{13}F_2N_2$ 211.1041; Found 211.1047.

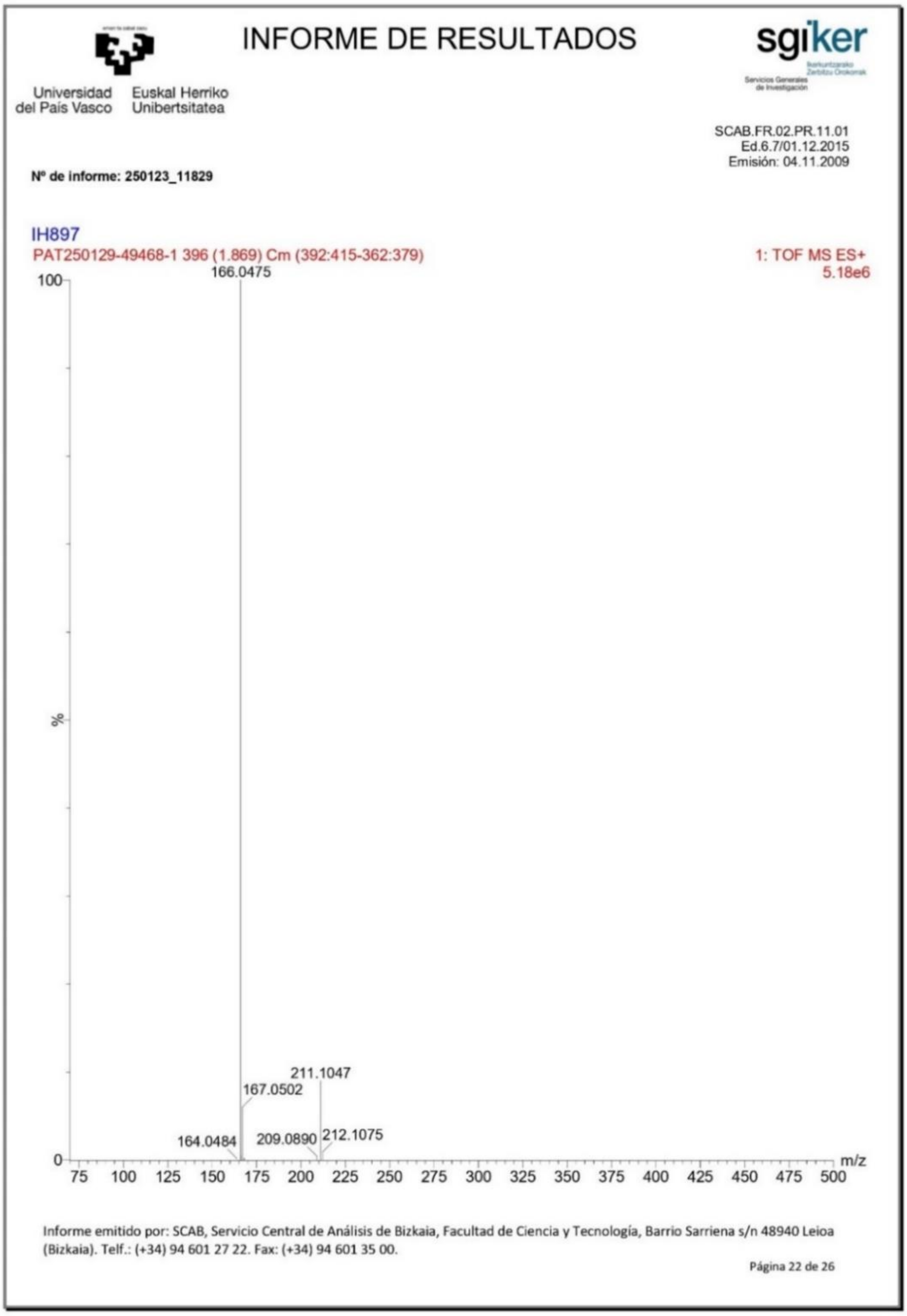

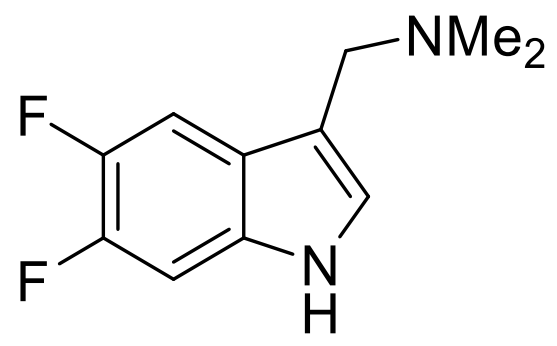

1b

HRMS (ESI) m/z: [M+H]+ Calcd. for $C_{11}H_{13}F_2N_2$ 211.1041 ; Found 211.1052.

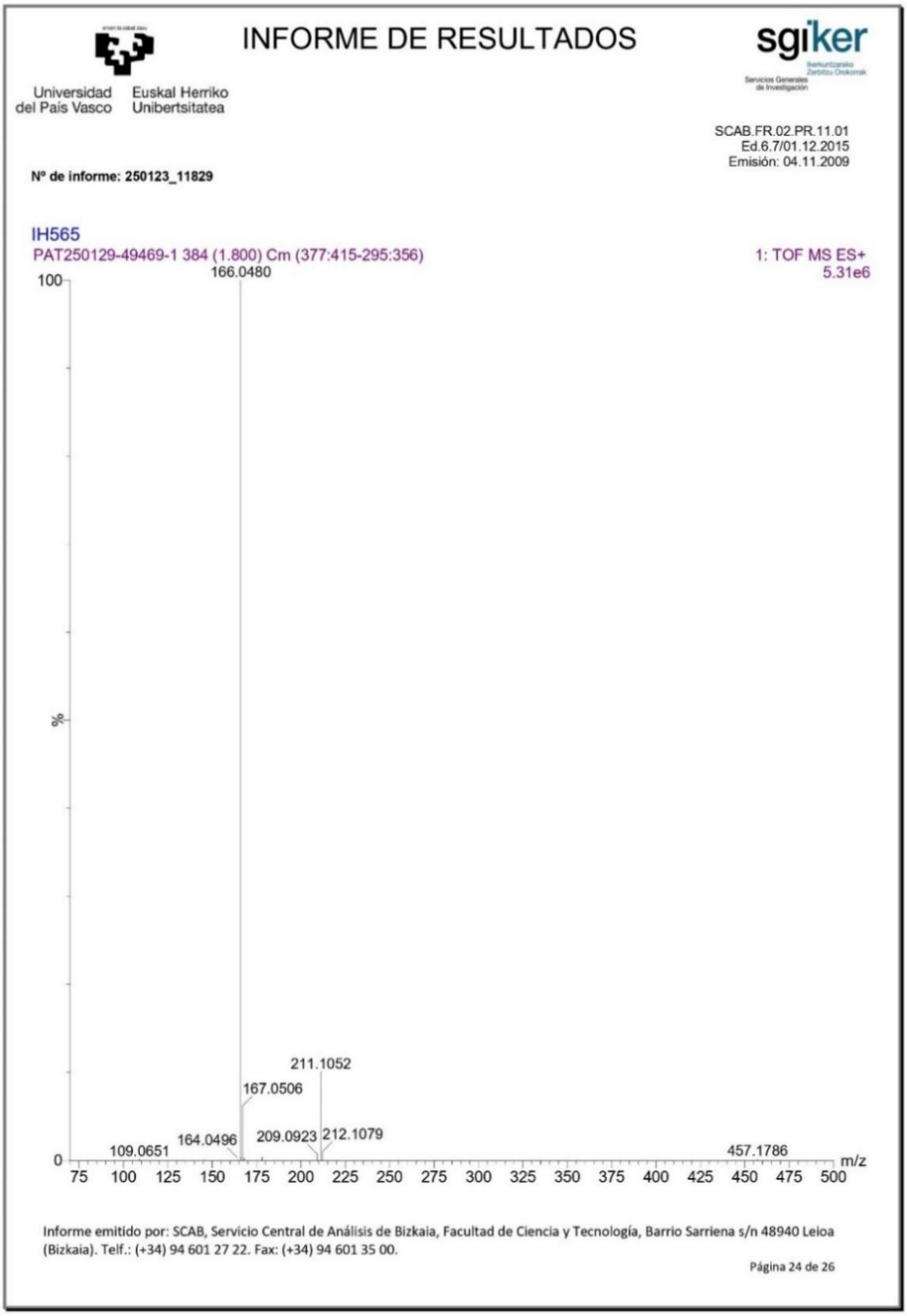

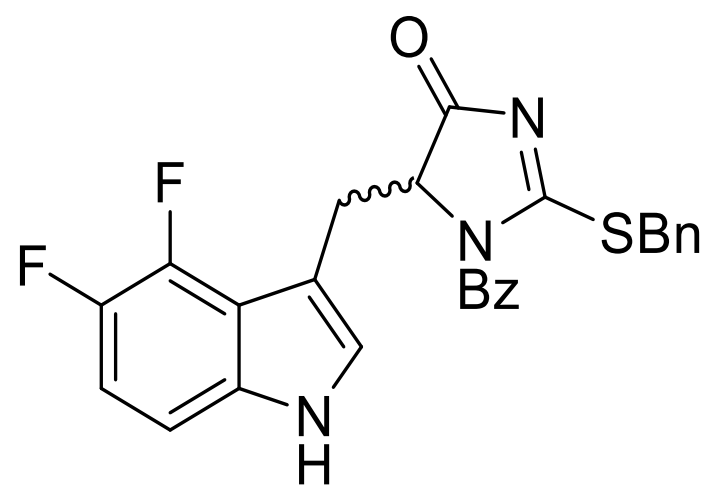

4a

HRMS (ESI) m/z: $[M+Na]^+$ Calcd. for $C_{26}H_{19}F_2N_3O_2SNa$* 498.1058; Found 498.1067.

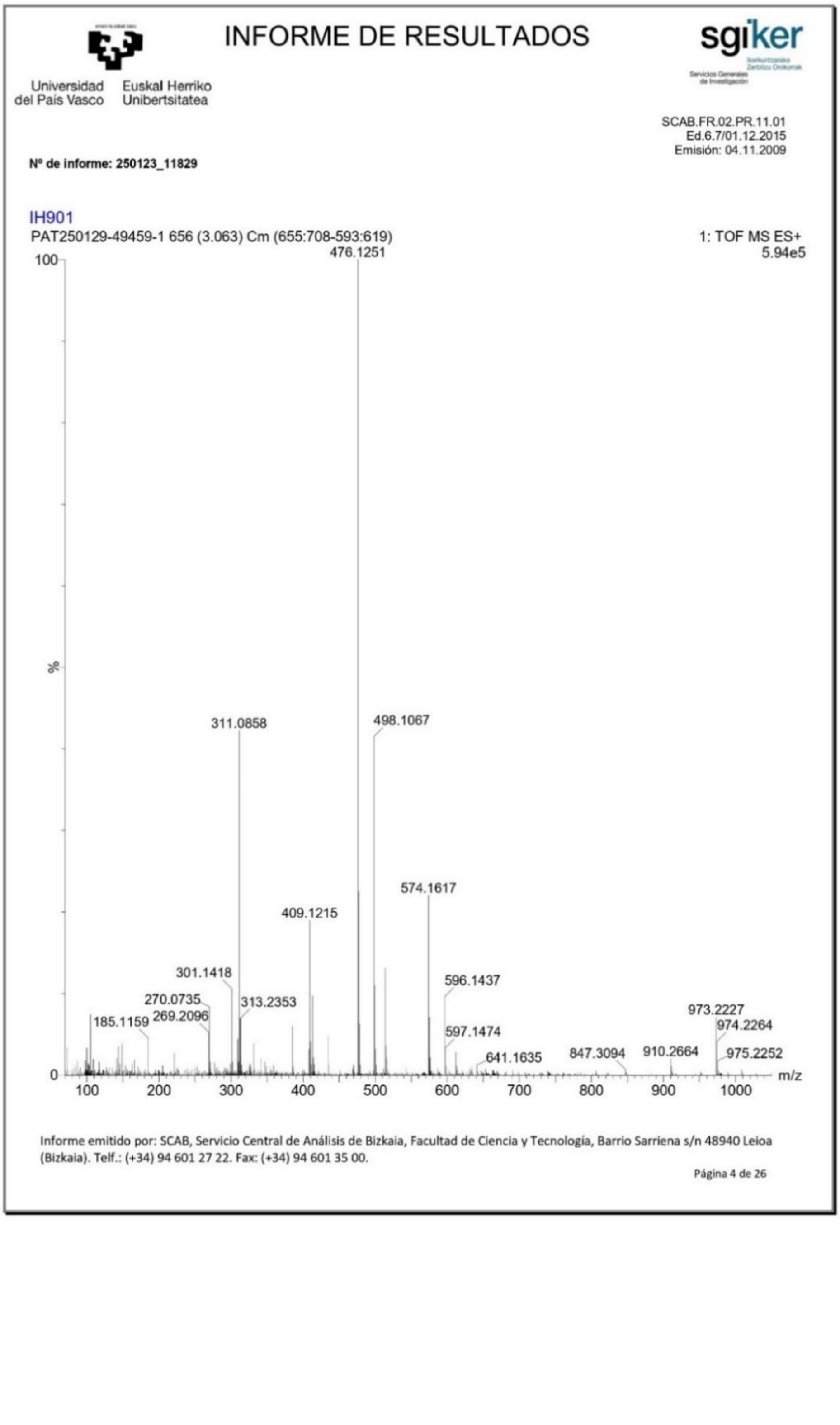

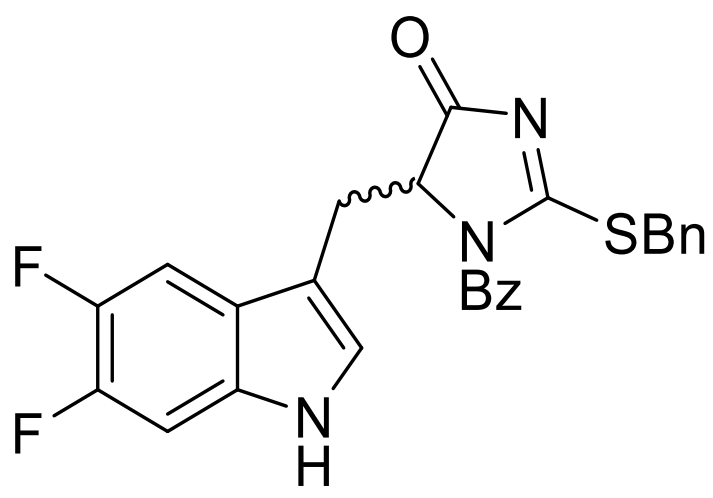

4b

HRMS (ESI) m/z: $[M+H]^+$ Calcd. for $C_{26}H_{20}F_2N_3O_2S$ 476.1239; Found 476.1252.

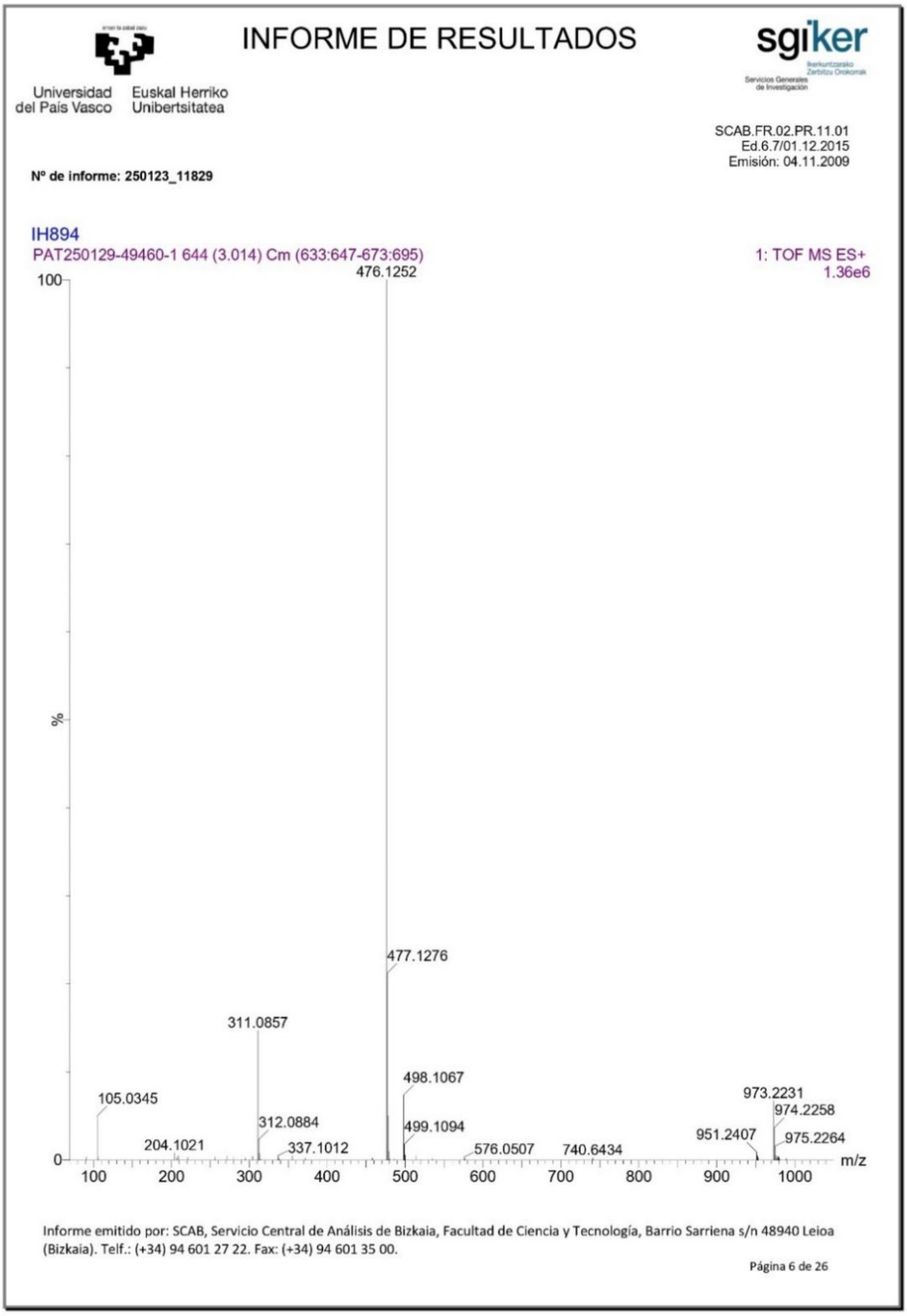

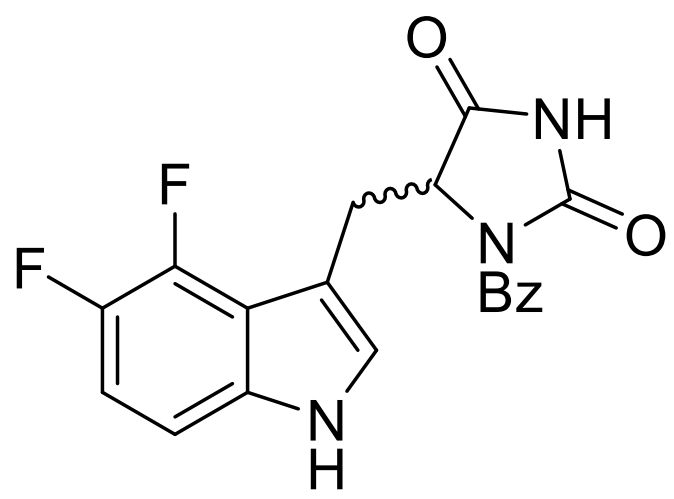

5a

HRMS (ESI) m/z: $[M+H]^+$ Calcd. for $C_{19}H_{14}F_2N_3O_3$ 370.0998 ; Found 370.1004.

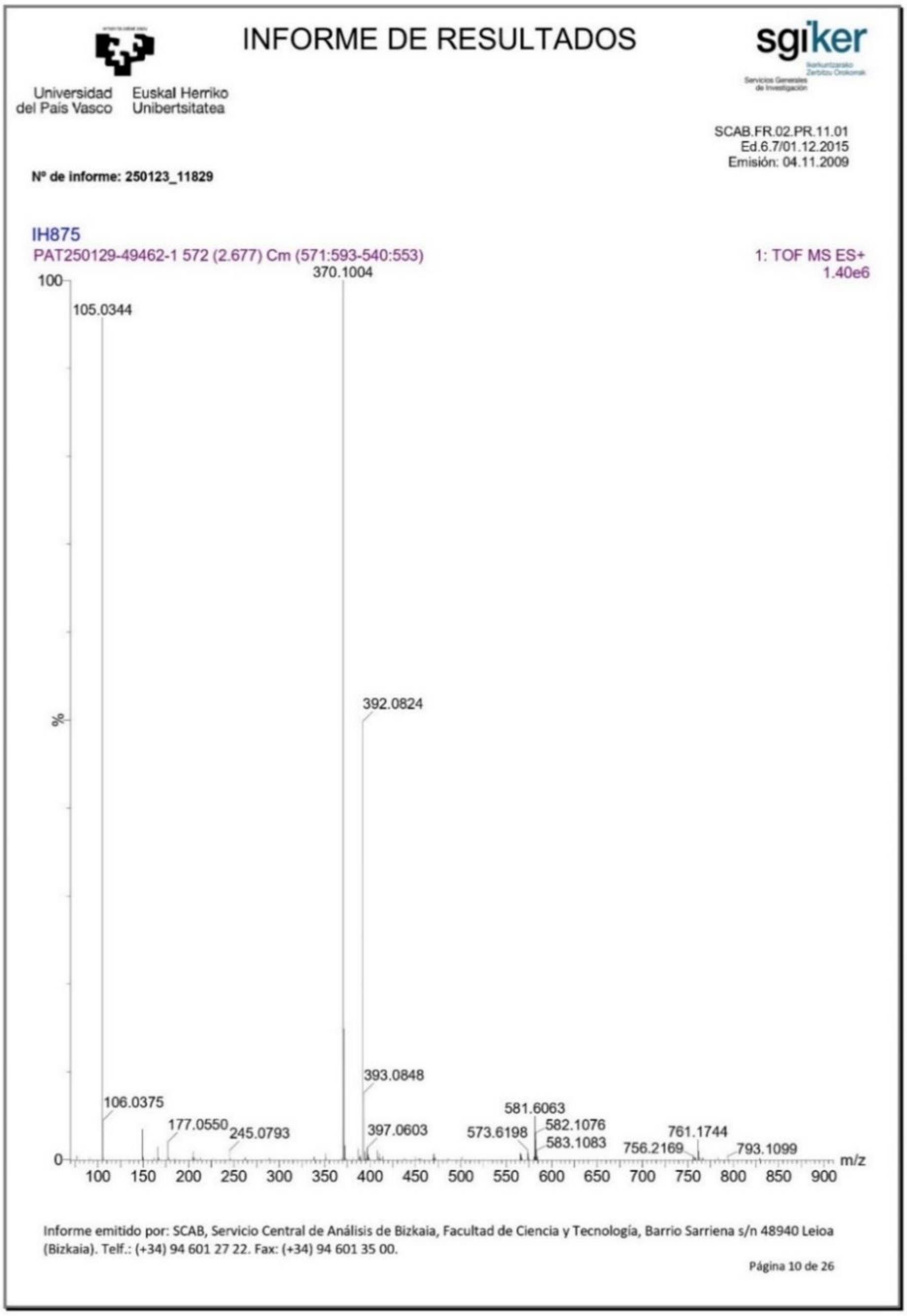

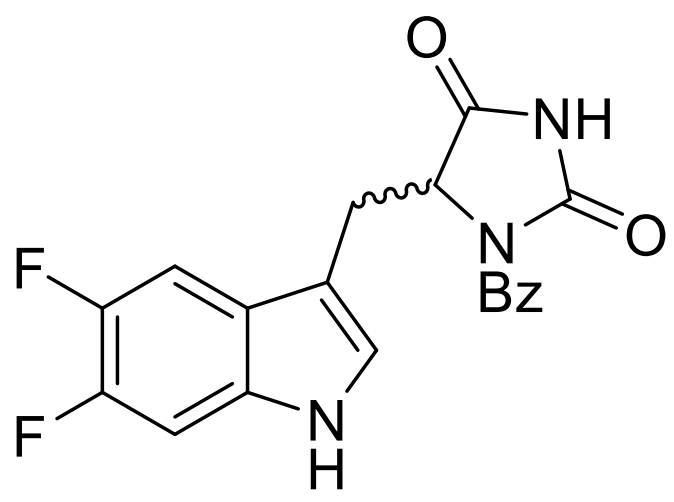

5b

HRMS (ESI) m/z: $[M+H]^+$ Calcd. for $C_{19}H_{14}F_2N_3O_3$ 370.0998; Found 370.0998.

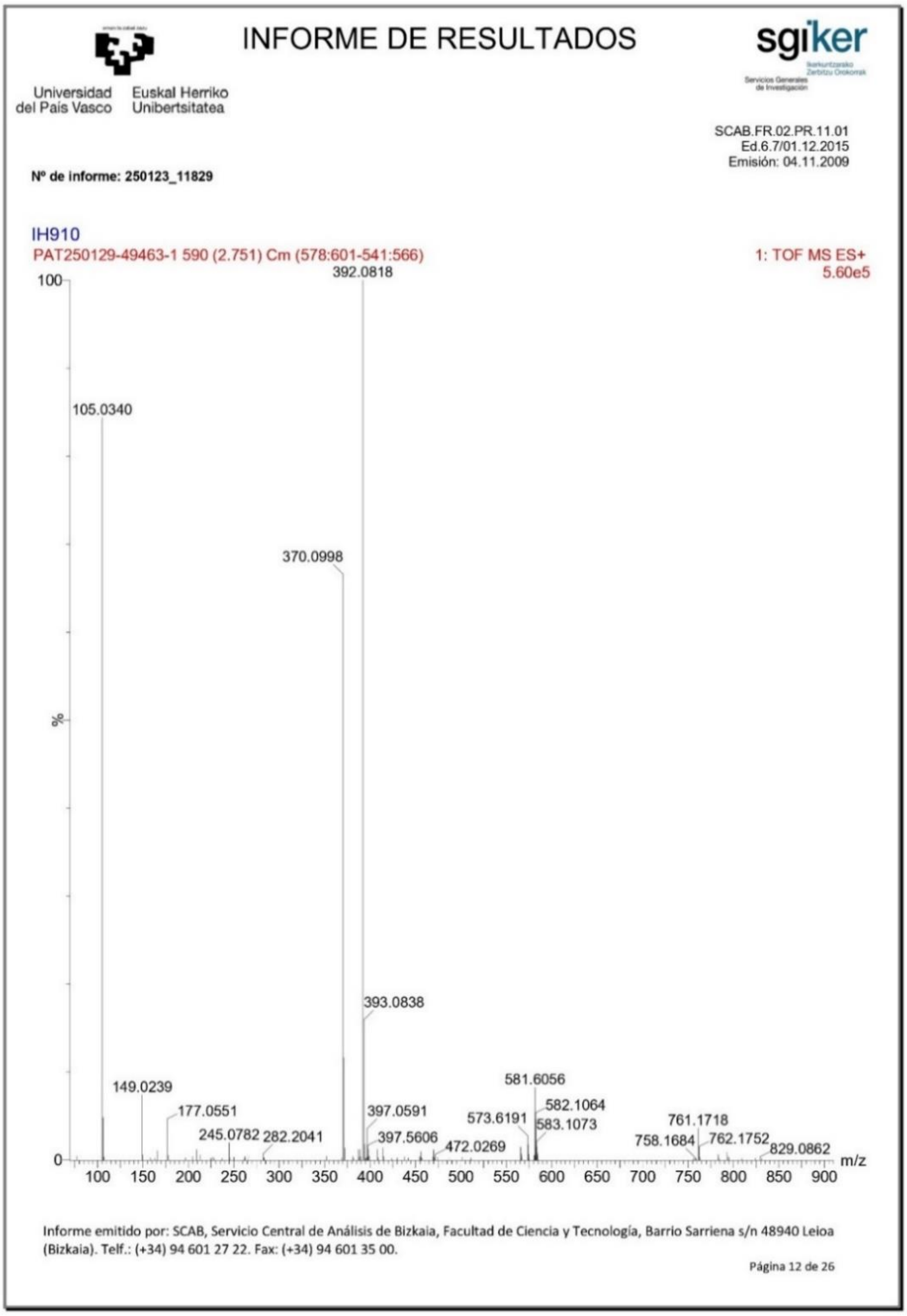

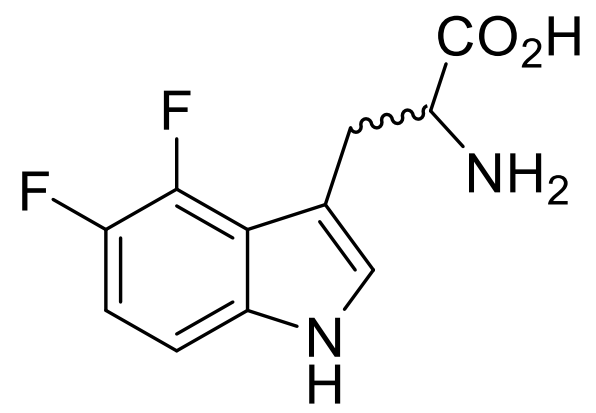

6a

HRMS (ESI) m/z: [M+H]$^+$ Calcd. for $C_{11}H_{11}F_2N_2O_2$ 241.0783; Found 241.0792.

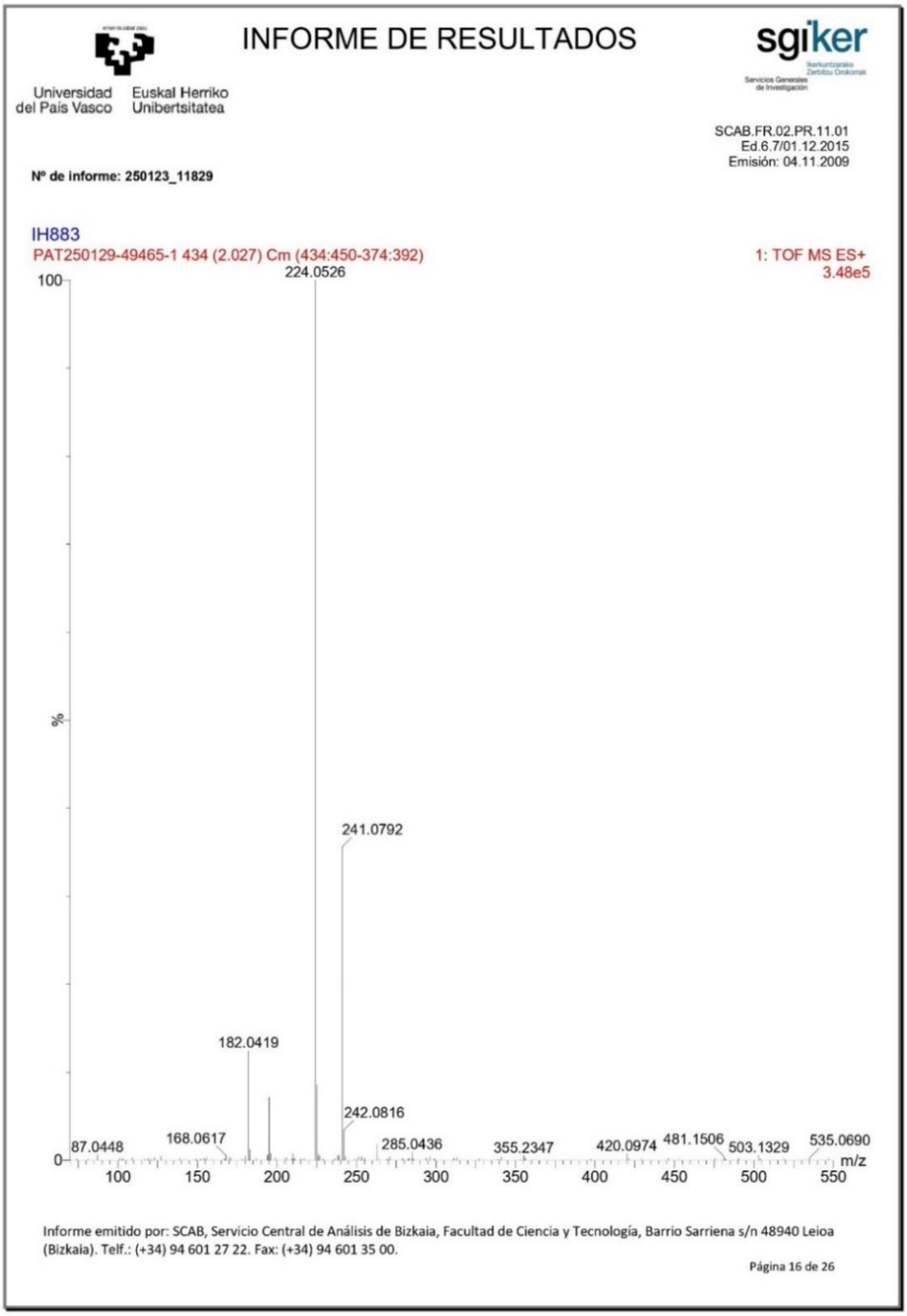

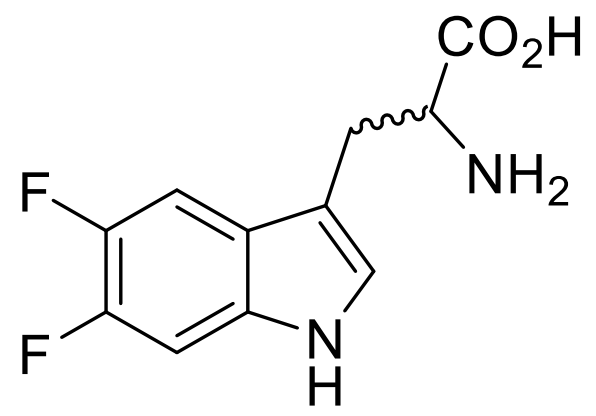

6b

HRMS (ESI) m/z: $[M+H]^+$ Calcd. for $C_{11}H_{11}F_2N_2O_2$ 241.0783; Found 241.0792.

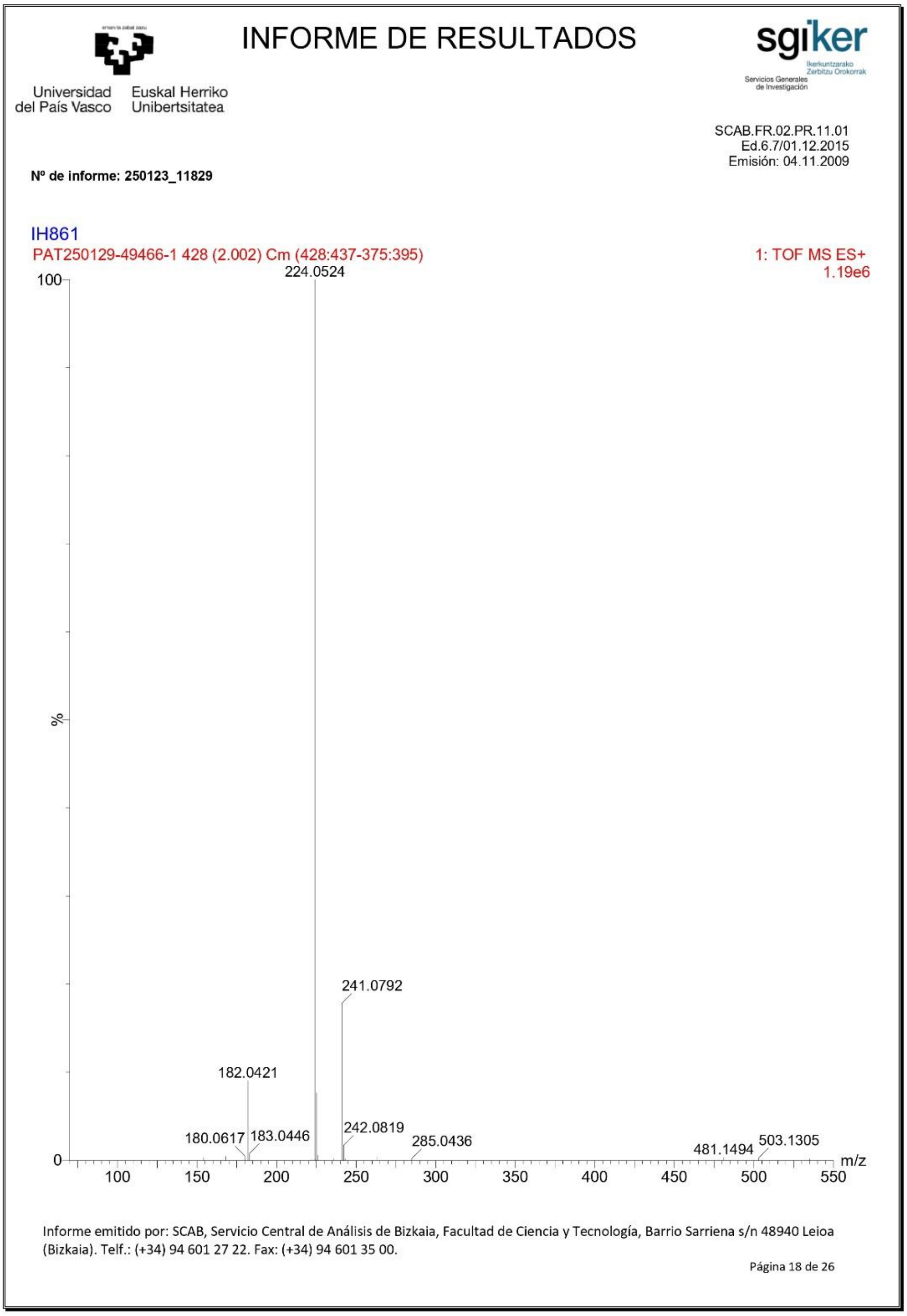

8. Protein preparation protocol via biosynthetic expression in *E. coli*.

BL21(DE3) *E. coli* cells were transformed with a plasmid encoding a construct coding for the CypA protein (pET21CypA EoptV2QFL), following already described experimental procedures.[18,19] Cultures were grown at 37 °C in M9 modified minimal medium containing 100 μg/L Carbenicillin, 1 g/L $^{14}NH_4Cl$, and 2 g/L of glucose as the sole nitrogen and carbon sources, respectively. At OD_{600} of 0.6–0.7, the cultures were supplemented with 1 g/L of glyphosate and 100 mg/L of the two aromatic amino acids, phenylalanine and tyrosine, as well as 50–100 mg/L of the corresponding fluorinated amino acid: 4,5-difluoro tryptophan, 4,6-difluoro tryptophan or 5,6-difluoro tryptophan. The difluorinated Trp analogues were added in racemic form, with *E. coli* selectively incorporating the corresponding L-enantiomers during protein expression. After 45 minutes, protein expression was induced with 1 mM IPTG. The cultures were then grown at 18 °C for an additional 16 h. Cells were harvested by centrifugation at 3,500 g for 30 min at 4 °C. Cell pellets were resuspended in 40 mL lysis buffer (25 mM sodium phosphate, pH 5.8, 5 mM DTT) and lysed by sonication. Cell debris was removed by centrifugation at 33,000 g for 60 min at 4 °C. The clarified lysates were loaded onto a 5 mL ion-exchange column (HiTrap SP HP, 5 mL) and eluted using a gradient from 0–50% 1 mM NaCl in 25 mM sodium phosphate buffer, pH 5.8, 2 mM DTT, 0.02% NaN_3. Proteins were eluted at ~ 10 mS/cm conductivity, and protein-containing fractions were collected, pooled, and concentrated in an Amicon with a 3000 Da cut-off. Concentrated protein fractions were further purified by size-exclusion over a Superdex 75 10/300 GL column (GE Healthcare Life Sciences) using phosphate-buffered saline, pH 7.4, containing 2 mM DTT. Protein purity and fluorine incorporation were assessed by mass spectrometry, SDS-PAGE and ^{19}F NMR.

Employing a fluorinated Trp concentration of 50 mg per cell culture liter, the preparation of 4,5-F_2-Trp CypA yielded 52 mg L^{-1} of purified protein, resulting in an incorporation efficiency of 91% (Figure S1). Under identical conditions, 4,6- and 5,6-F_2-Trp CypA proteins were obtained with lower incorporation efficiencies of 68% (Figure S2) and 80% (Figure S4), respectively. Increasing the Trp concentration to 100 mg L^{-1} improved the incorporation rates to 83% for 4,6-F_2-Trp CypA (Figure S3) and 95% for 5,6-F_2-Trp CypA (Figure S5), yielding 8.6 mg L^{-1} and 4.4 mg L^{-1} of purified protein, respectively. Notably, increasing the concentration of the Trp did not lead to substantial changes in protein yield.

9. Protein Mass Spectra, SDS-PAGE and overlaid 1D ^{19}F NMR.

The theoretical molecular masses used to determine ^{19}F-Trp incorporation in Figures S1–S5 were 18041.5 Da for unlabeled CypA and 18077.5 Da for the F_2-Trp CypA analogues.

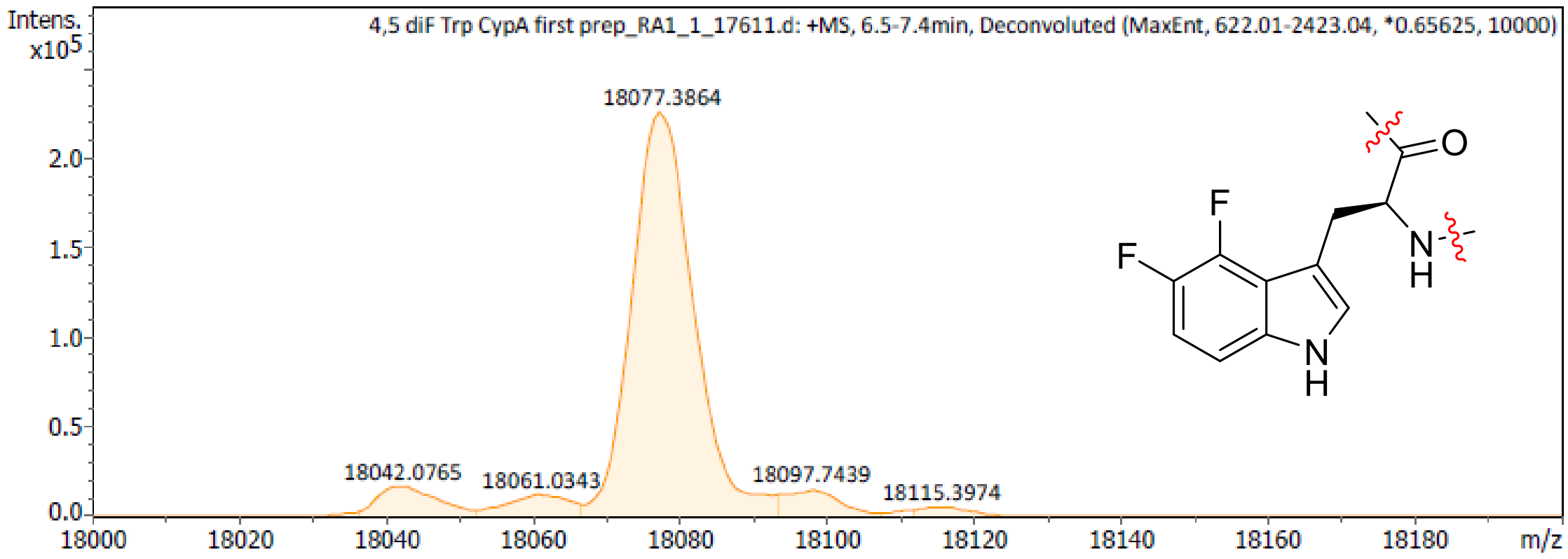

Figure S1: Mass spectrum of 4,5-F_2-Trp CypA prepared from 50 mg of labeled amino acid per liter of cell culture. Reported incorporation: 91%.

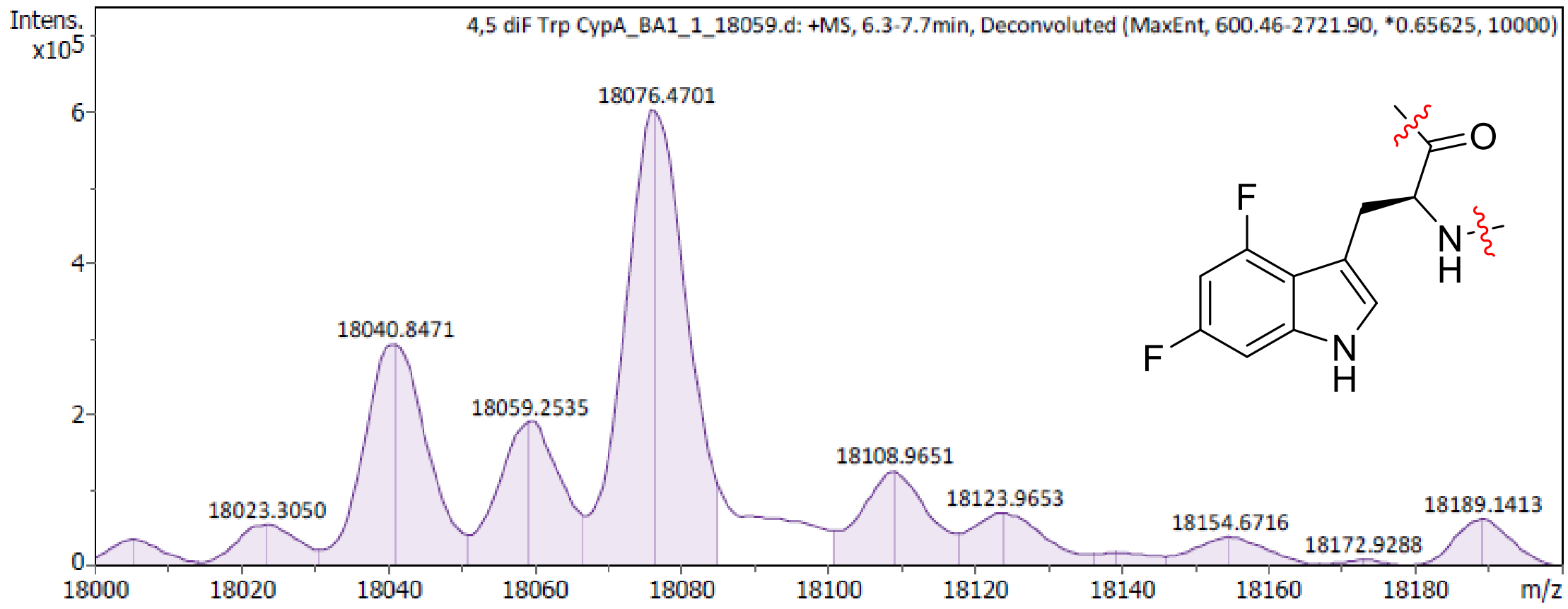

Figure S2: Mass spectrum of 4,6- F_2-Trp CypA prepared from 50 mg of labeled amino acid per liter of cell culture. Reported incorporation: 68%.

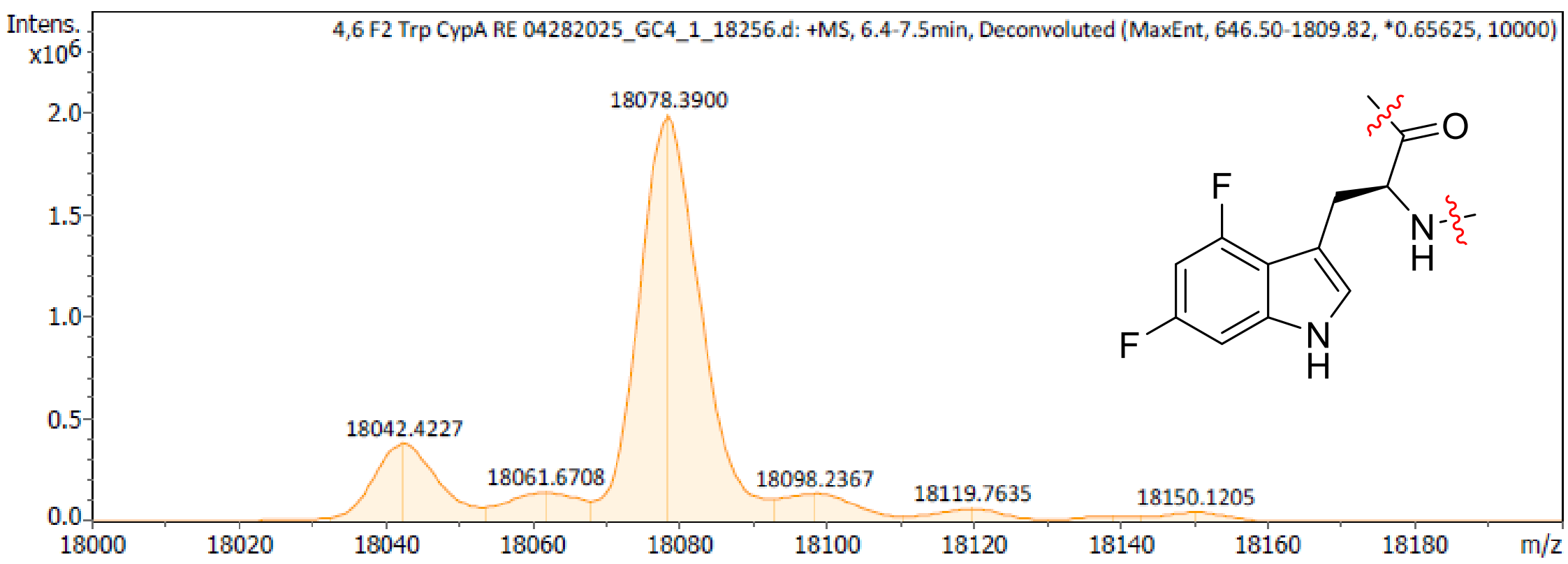

Figure S3: Mass spectrum of 4,6- F_2-Trp CypA prepared from 100 mg of labeled amino acid per liter of cell culture. Reported incorporation: 83%.

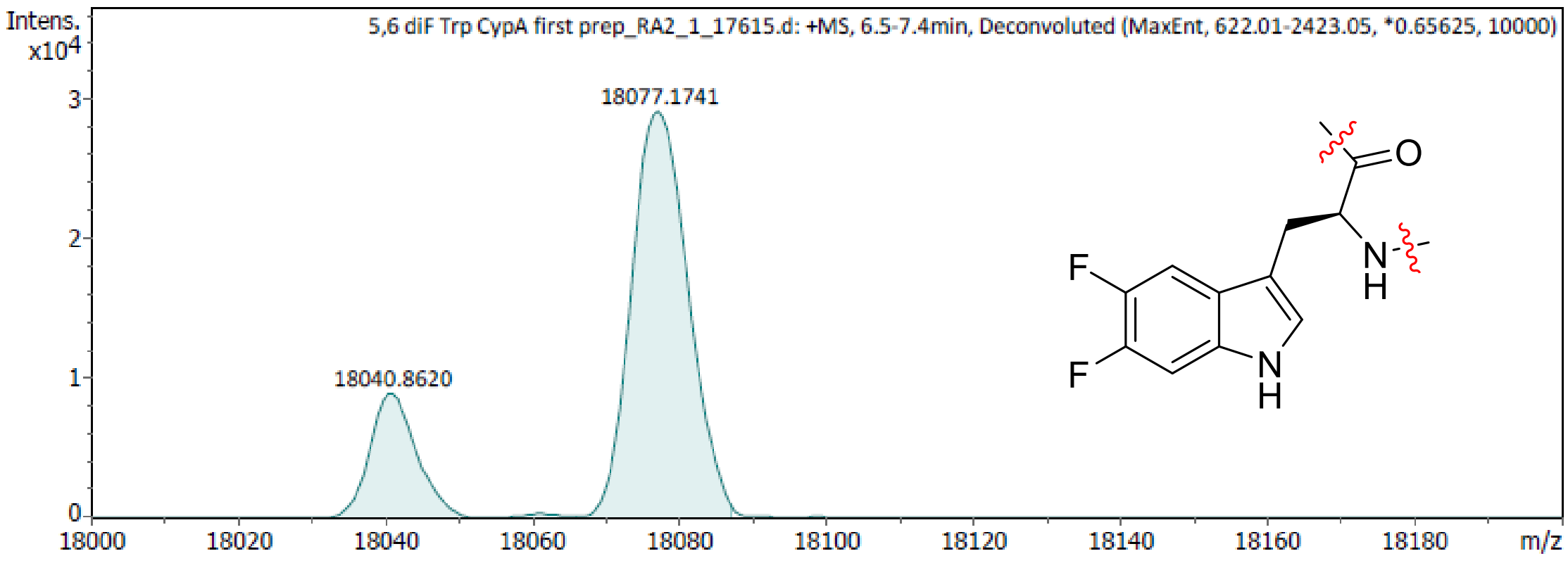

Figure S4: Mass spectrum of 5,6- F_2-Trp CypA prepared from 50 mg of labeled amino acid per liter of cell culture. Reported incorporation: 80%.

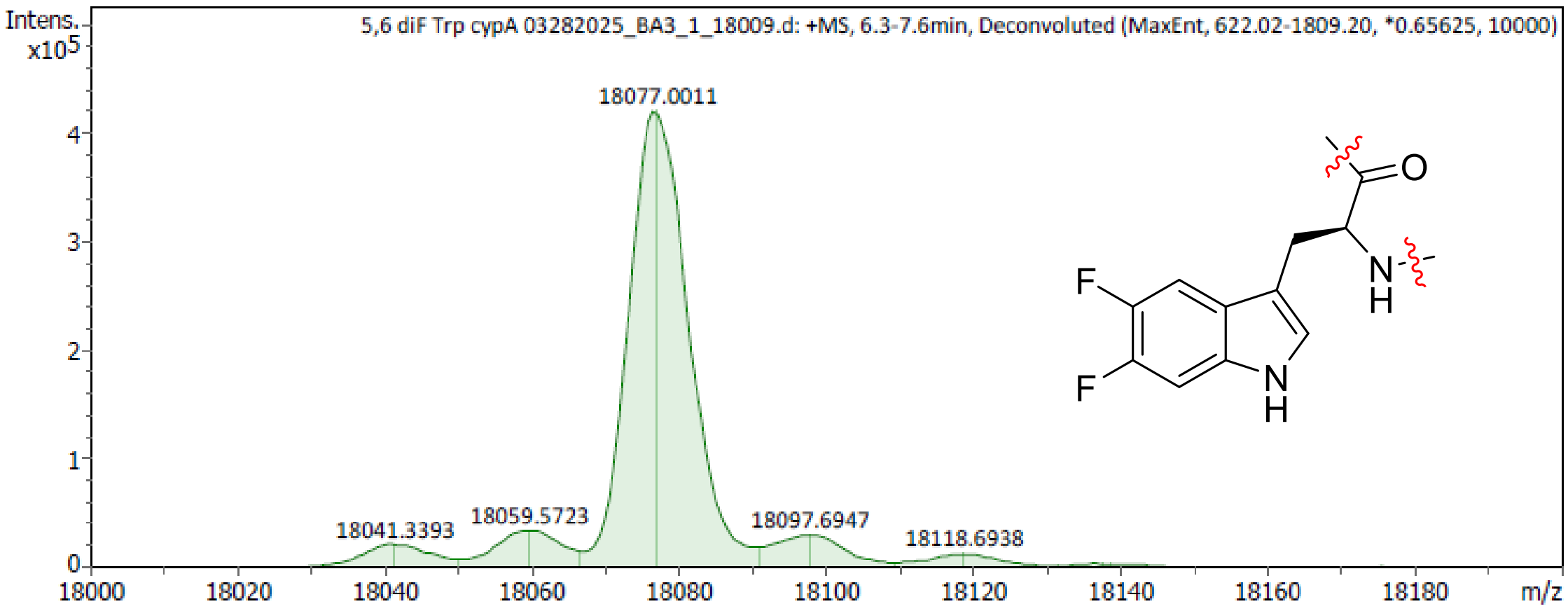

Figure S5: Mass spectrum of 5,6- F_2-Trp CypA prepared from 100 mg of labeled amino acid per liter of cell culture. Reported incorporation: 95%.

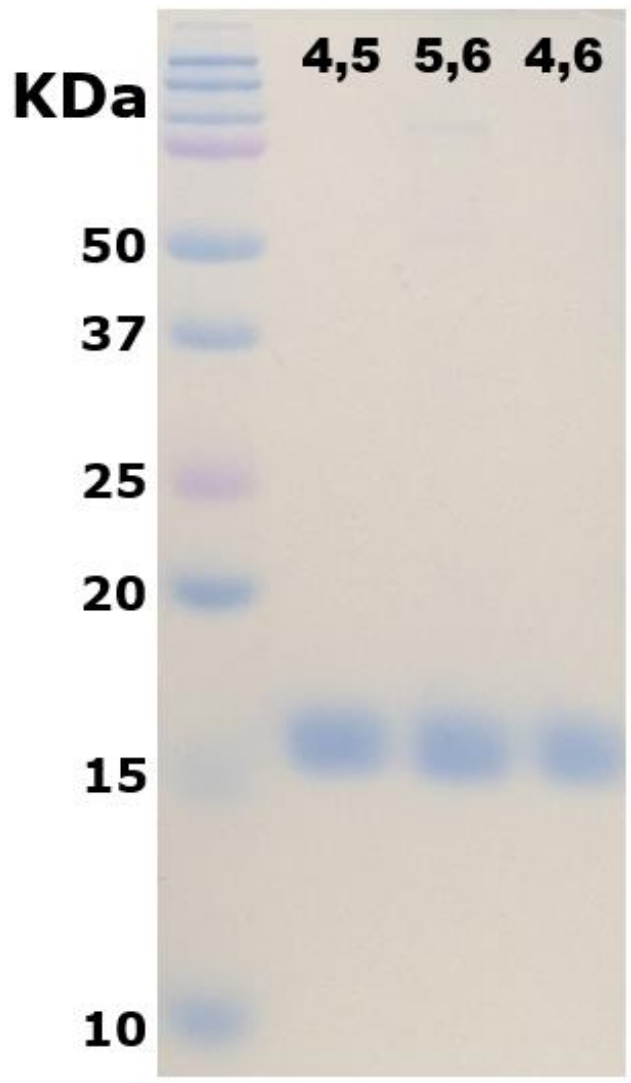

Figure S6: SDS-PAGE analysis to assess the purity of the three proteins after purification by ion-exchange and size-exclusion chromatography.

Figure S7 displays the overlaid 1D ^{19}F NMR spectra of the three fluorinated CypA analogs (each acquired separately). Samples were prepared in 25 mM sodium phosphate buffer, pH 6.5, 1 mM TCEP, 0.02% NaN_3, and 7% D_2O. The spectra show well-resolved signals for 4,6- and 5,6-F_2-Trp CypAs, while some signal overlap is observed for 4,5-F_2-Trp CypA. Sample concentrations were 500 µM, 660 µM, and 307 µM, respectively.

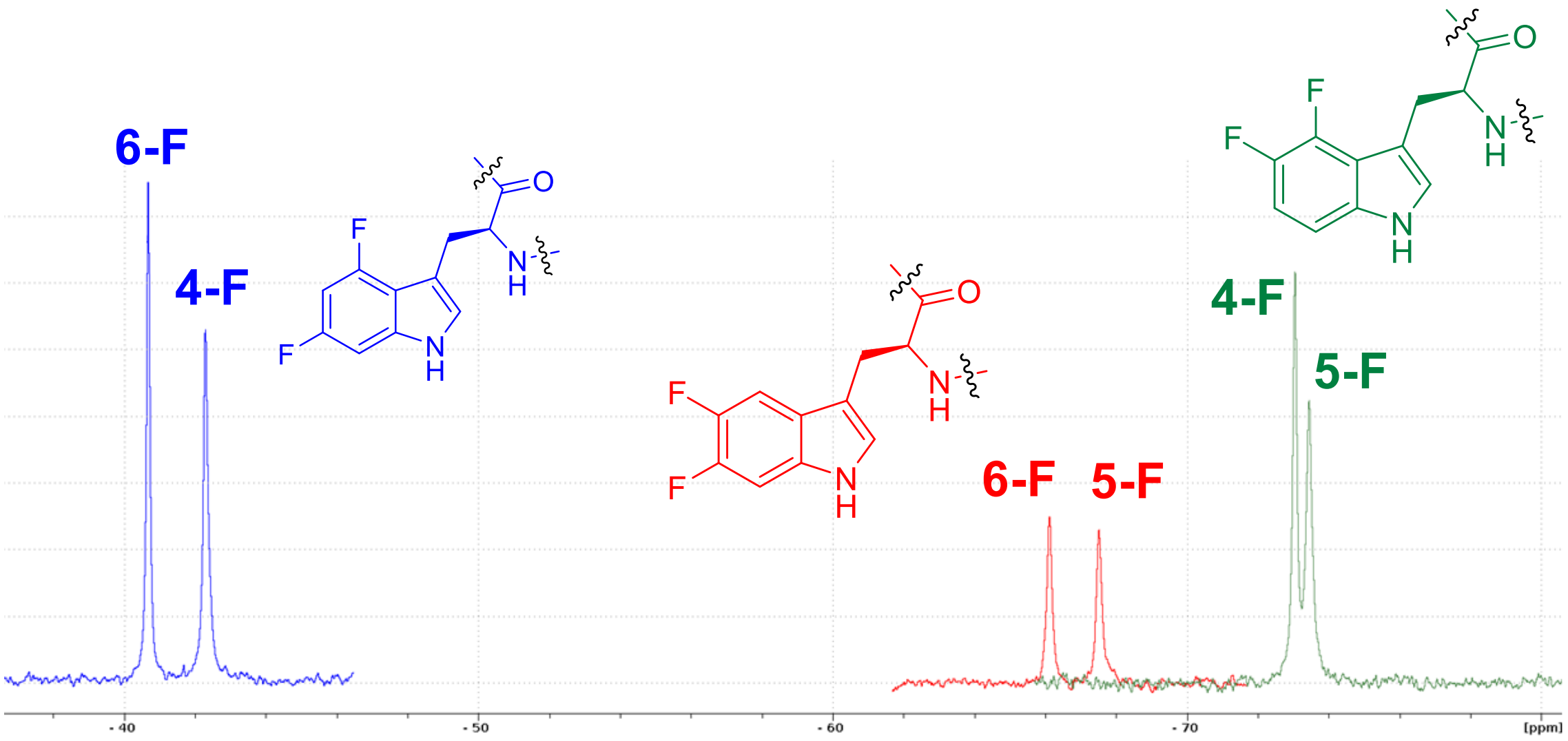

Figure S7: ^{19}F NMR spectra of 4,6-F_2-Trp CypA (blue), 5,6-F_2-Trp CypA (red) and 4,5-F_2-Trp CypA (green) proteins.

The assignment of the individual ^{19}F resonances was established from the spectra of the free F_2-Trp amino acids and subsequently transferred to the corresponding labeled protein spectra. For free 4,5-F_2-Trp and 4,6-F_2-Trp, the individual ^{19}F resonances were assigned from the corresponding ^{1}H–^{19}F coupling patterns observed in the ^{19}F NMR spectra. For free 5,6-F_2-Trp, the assignment was based on the chemical shifts observed for the corresponding free monofluorinated Trp amino acids, in which the 6-F resonance appears at lower field than the 5-F resonance.* This trend has been reported to be maintained upon incorporation of monofluorinated Trp derivatives into CypA, with the 6-F resonance remaining at lower field than the 5-F resonance.[11]

For direct comparison with the protein spectra, additional ^{19}F NMR spectra of the free F_2-Trp derivatives were recorded in H_2O and referenced to TFA. The resulting ^{19}F chemical shifts correspond to those indicated by lines in Figure 1b of the main manuscript.

* The spectra of free 4,5-F_2-Trp and 4,6-F_2-Trp are also consistent with this trend, with 6-F at lower field, 4-F in the middle, and 5-F at higher field.

^{19}F NMR (376 MHz, D_2O) of **6a**:

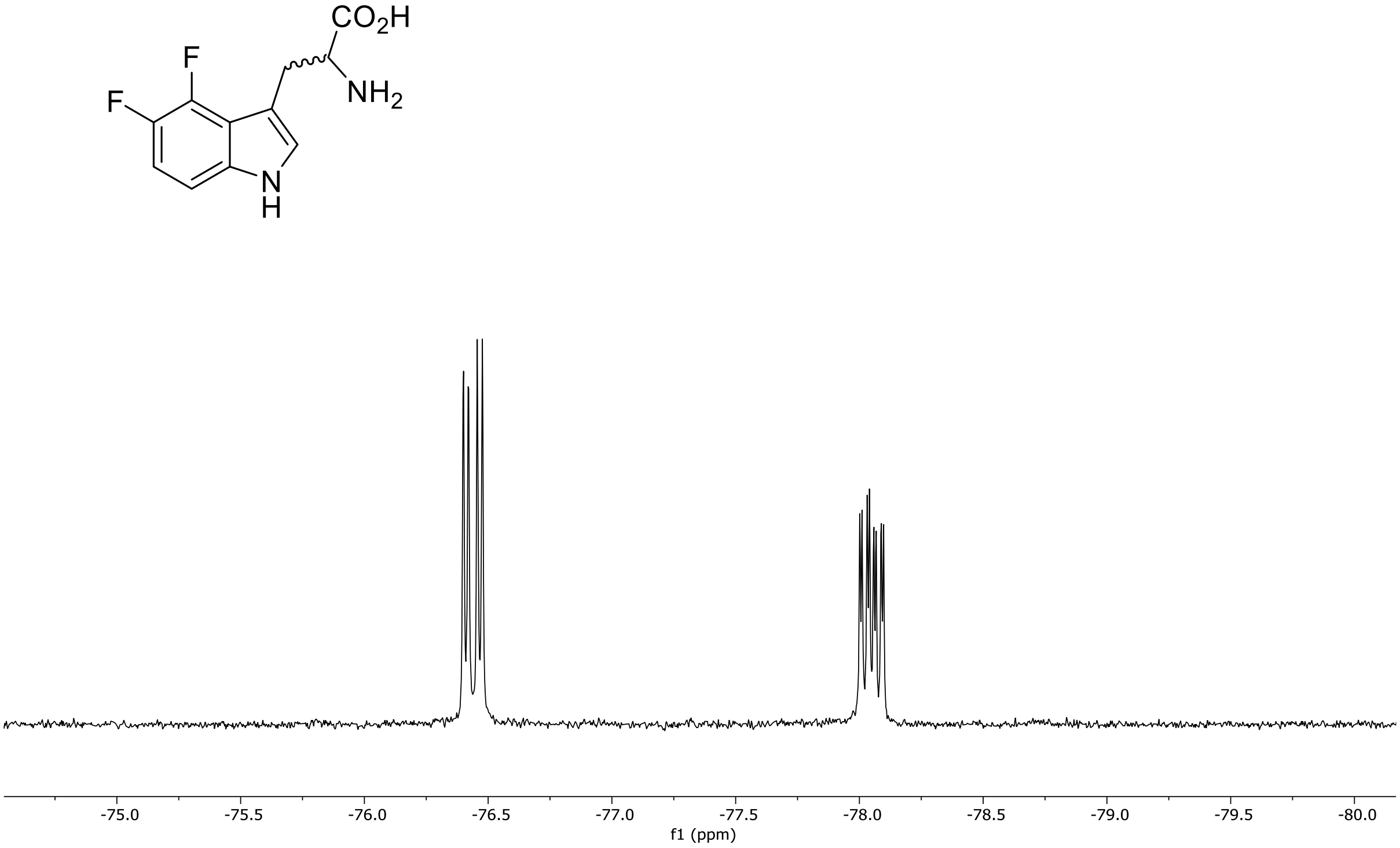

^{19}F NMR (376 MHz, D_2O) of **6b**:

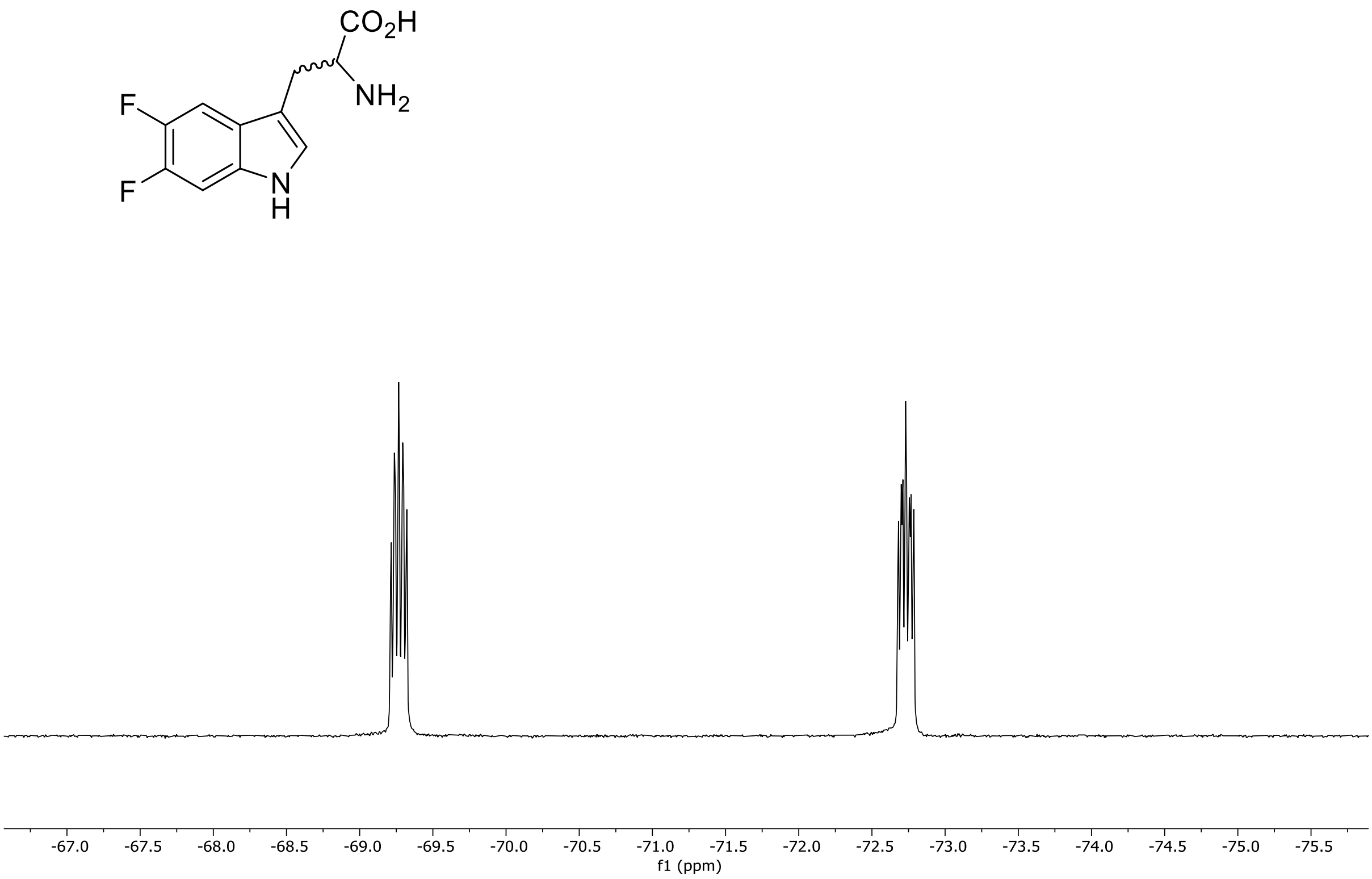

^{19}F NMR (376 MHz, D_2O) of **6c**:

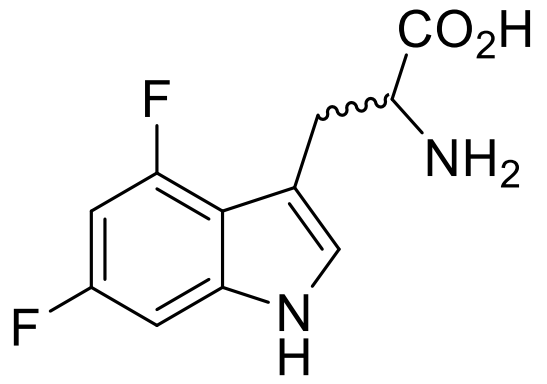

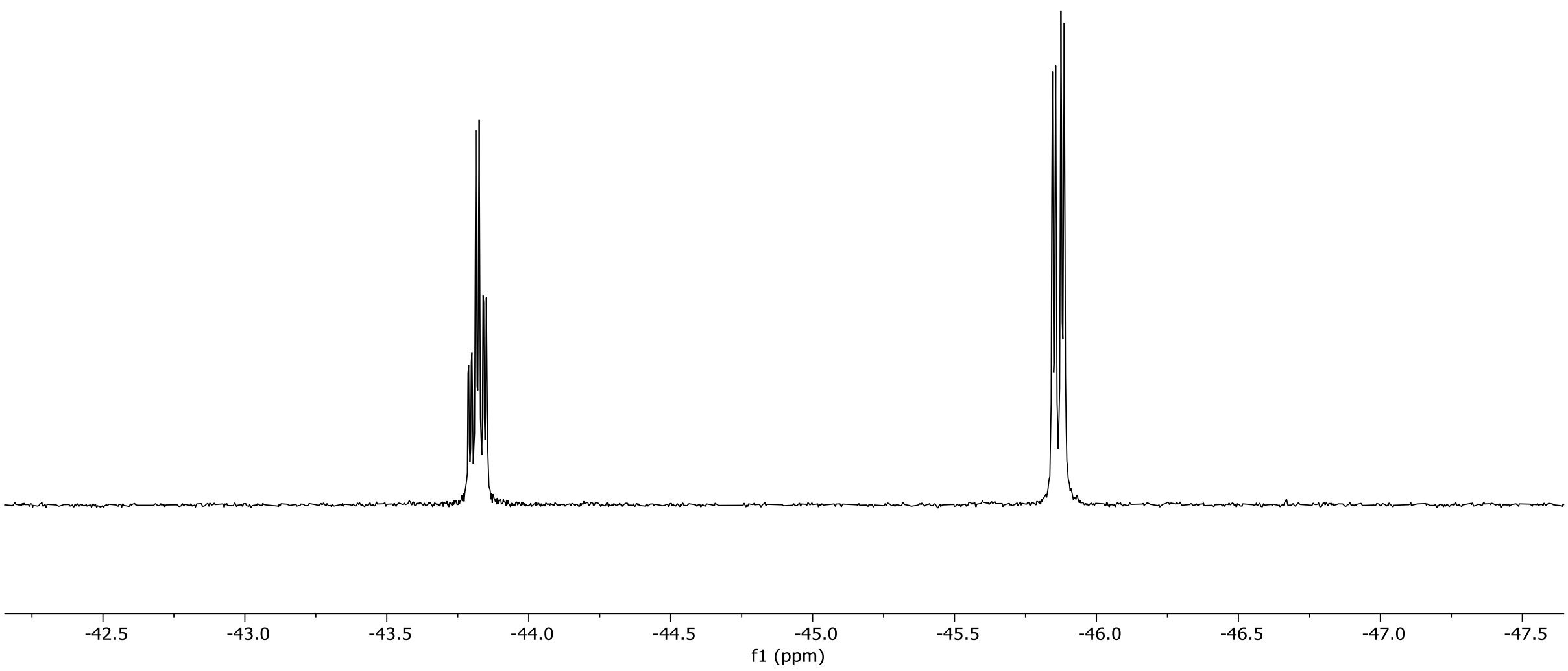

10. Reference distances

Reference structures with fluorination patterns similar to 5,6- and 4,5-F_2-Trp were extracted from the CCDC database and analyzed with Avogadro 1.2.0 and Chem3D 17.0. Table S1 summarizes the corresponding fluorine–fluorine distances.

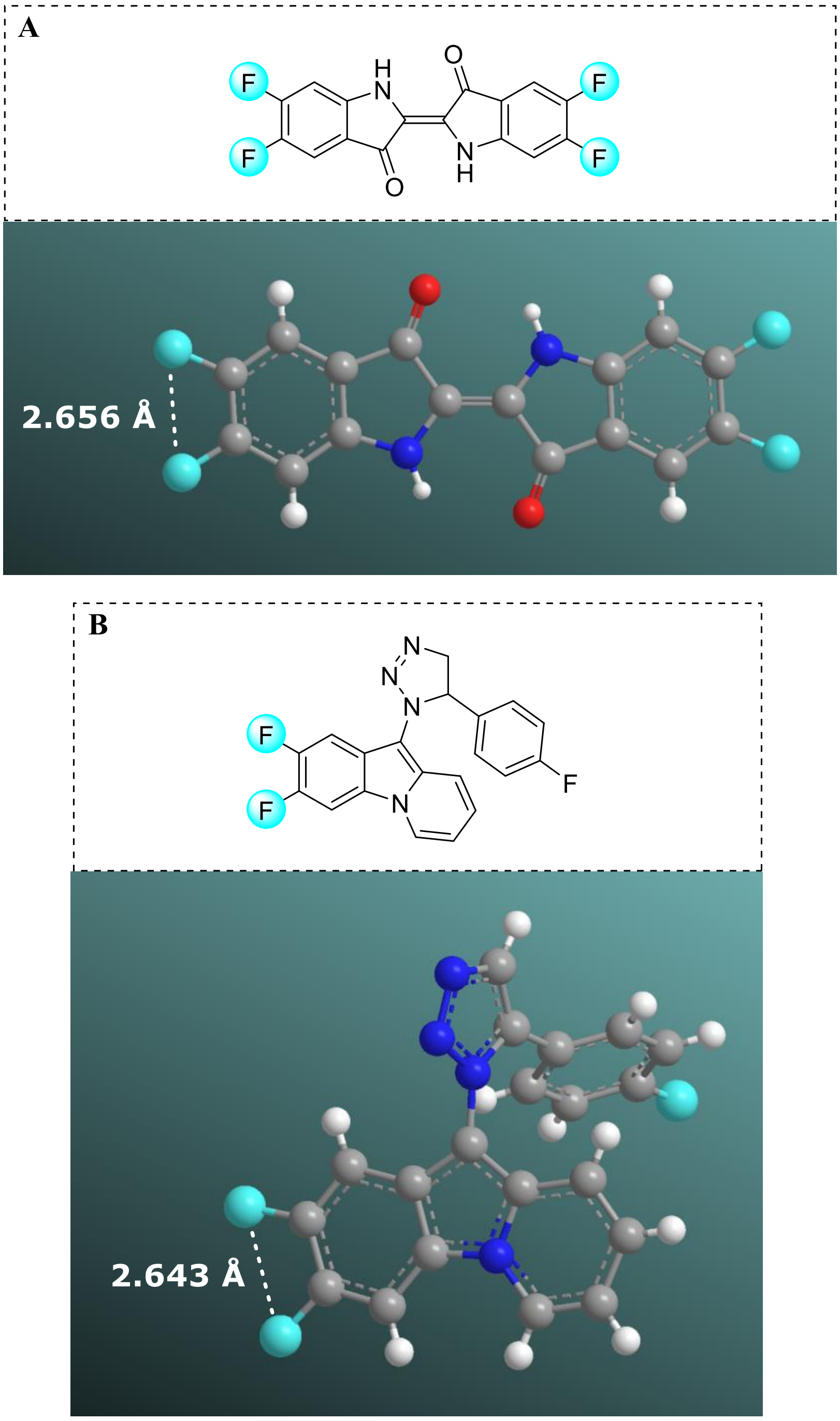

Figure S8: Reference distances for 5,6-F_2-Trp CypA.[34,35]

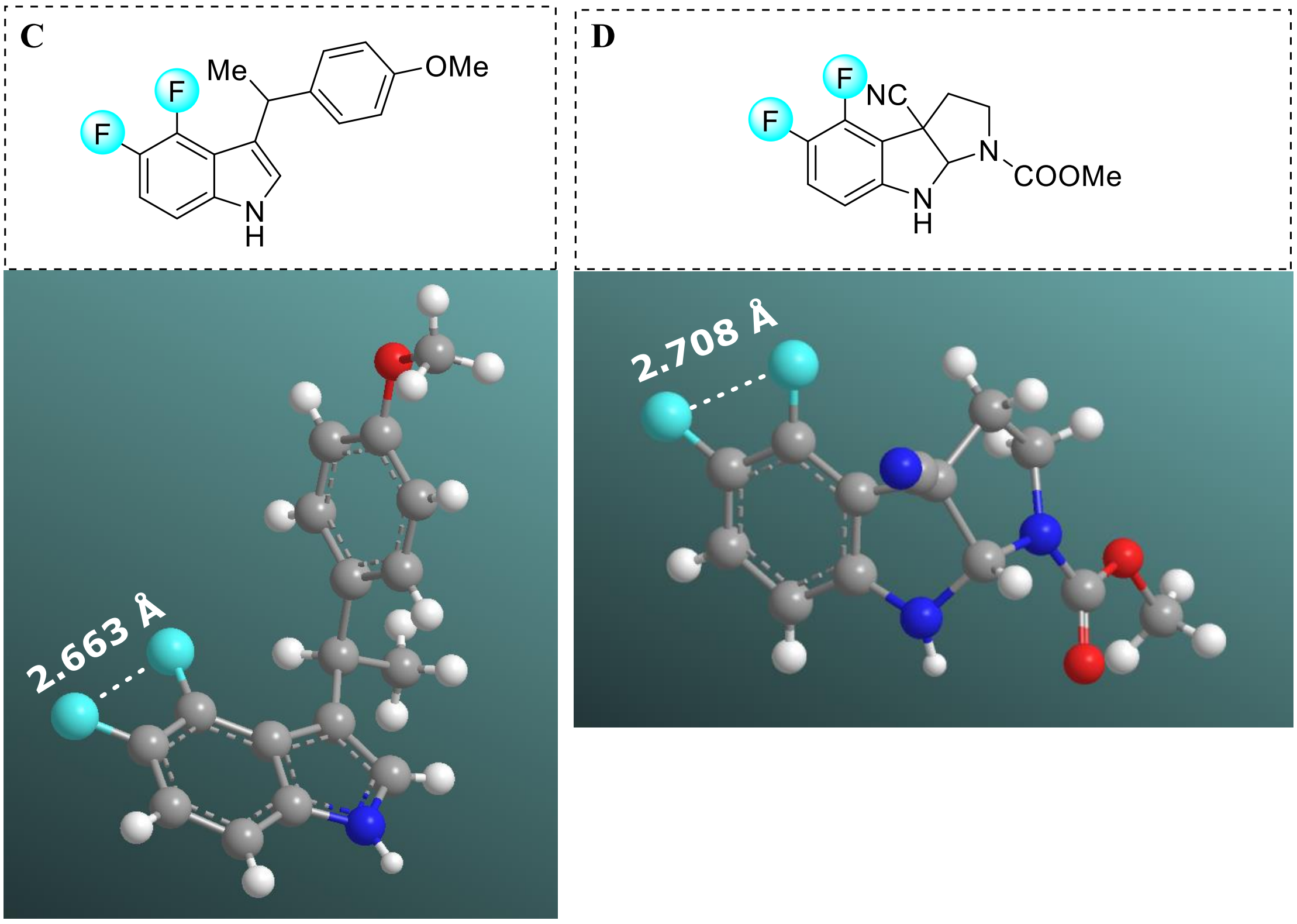

Figure S9: Reference distances for 4,5-F_2-Trp CypA.[36,37]

F–F distances (X-Ray - CCDC)			Average
**5F ↔ 6F**	2.656	2.643	**2.650**
**4F ↔ 5F**	2.663	2.708	**2.686**

Table S1: Summary of the reference distances for 4,5- and 5,6-F_2-Trp.

11. X-Ray analysis of 4,6-F_2-Trp 6c.

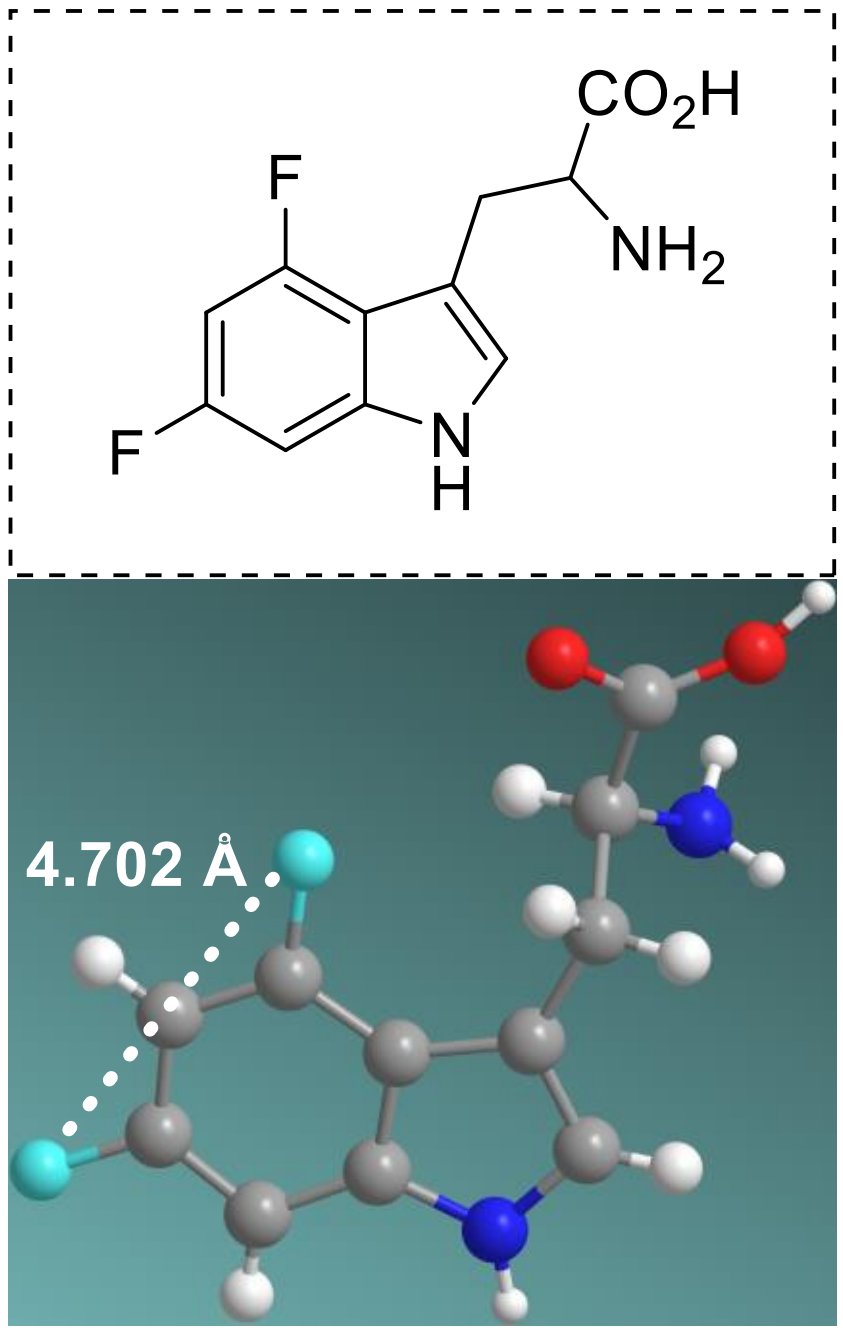

Figure S10: Structure of 4,6-F_2-Trp with labeled F–F distance (measured with Avogadro 1.2.0).

11.1. Method for sample preparation

An analytical solution of each pure amino acid in MeOH was placed into a small vial, which was placed into a bigger vial containing n-hexane. The external vial was covered with a film (aluminum foil) in which a small orifice was introduced to allow the solvent to evaporate slowly.

11.2. Instrumentation

X-Ray analysis measurement was carried out using a four-circle\k-geometry Agilent SuperNova diffractometer provided with a Hybrid Pixel Array detector of the type HyPix with area resol mean of 10 based on '\w scans' method.

11.3. Crystal parameters

Identification code	b20260008_IH46F2TrpDUCu5
Empirical formula	$C_{11}H_{10}F_2N_2O_2$
Formula weight	240.21
Temperature/K	169.99(10)
Crystal system	monoclinic
Space group	$P2_1/n$
a/Å	5.86452(16)
b/Å	5.00639(17)
c/Å	35.2442(9)
α/°	90.0
β/°	90.283(3)
γ/°	90.0
Volume/Å^3	1034.76(5)
Z	4
ρ_{calc}g/cm^3	1.542
μ/mm^{-1}	1.141
F(000)	496.0
Crystal size/mm^3	0.422 × 0.089 × 0.058
Radiation	Cu Kα (λ = 1.54184)
2Θ range for data collection/°	5.014 to 137.976
Index ranges	$-7 \leq h \leq 7$, $-5 \leq k \leq 6$, $-42 \leq l \leq 42$
Index ranges	$-7 \leq h \leq 7$, $-5 \leq k \leq 6$, $-42 \leq l \leq 42$
Reflections collected	17435
Independent reflections	1912 [R_{int} = 0.1627, R_{sigma} = 0.0744]
Data/restraints/parameters	1912/6/163
Goodness-of-fit on F^2	1.161
Final R indexes [I>=2σ (I)]	R_1 = 0.1223, wR_2 = 0.3450
Final R indexes [all data]	R_1 = 0.1306, wR_2 = 0.3513
Largest diff. peak/hole / e Å^{-3}	0.63/-0.53

Table S2. Crystal data and structure refinement for b20260008_IH46F2TrpDUCu5. Weighting scheme: $1/[\sigma^2(Fo^2)+(0.1656P)^2+7.0460P]$ where $P = [Max(Fo^2,0)+2Fc^2]/3$. Secondary extinction expression of the SHELXL type: $Fc^* = kFc[1+0.001Fc^2\lambda^3/sen(2\theta)]^{-1/4}$

Atom	*x*	*y*	*z*	U(eq)
F1	2539(9)	7636(10)	4964.1(12)	53.5(14)
F2	7920(6)	7186(8)	3981.5(10)	33.0(10)
O1	10856(7)	6279(11)	3113.6(13)	36.7(13)
O2	9334(7)	5070(8)	2556.6(11)	23.4(10)
N1	1867(9)	1306(12)	3937.3(14)	29.0(13)
N2	5058(8)	5134(10)	2825.0(13)	20.4(11)
C1	9211(9)	5559(12)	2905.8(16)	19.3(12)
C2	6942(8)	5272(11)	3107.2(15)	16.8(12)
C3	6980(9)	2768(11)	3357.7(16)	20.4(12)
C4	5055(9)	2592(11)	3636.1(15)	19.0(12)
C5	3251(10)	893(13)	3625.1(17)	26.4(14)
C6	2778(9)	3330(13)	4156.6(17)	25.5(14)
C7	2000(12)	4434(15)	4491.7(18)	33.4(16)
C8	3269(13)	6452(15)	4638.8(18)	35.5(16)
C9	5272(12)	7402(14)	4479.6(17)	31.4(15)
C10	5979(9)	6268(13)	4148.9(17)	23.9(13)
C11	4774(9)	4192(12)	3973.8(15)	19.9(12)

Table S3. Fractional atomic coordinates (×10^4) and equivalent isotropic displacement parameters (Å^2×10^3) for b20260008_IH46F2TrpDUCu5. Ueq is defined as 1/3 of the trace of the orthogonalised U_{IJ} tensor.

Atom	U11	U22	U33	U23	U13	U12
F1	63(3)	67(3)	31(2)	-13(2)	11(2)	-4(2)
F2	21.5(19)	40(2)	38(2)	-0.3(16)	-0.5(15)	-16.4(15)
O1	13(2)	59(3)	37(3)	-13(2)	-3.4(17)	-10(2)
O2	19(2)	29(2)	22(2)	-2.1(16)	3.6(15)	-0.5(16)
N1	21(3)	37(3)	29(3)	5(2)	-5(2)	-13(2)
N2	13(2)	26(3)	22(2)	2.1(19)	-3.5(18)	-1.6(19)
C1	11(2)	20(3)	26(3)	-1(2)	-2(2)	0(2)
C2	8(2)	21(3)	22(3)	-2(2)	-4(2)	-1.8(19)
C3	17(3)	19(3)	25(3)	1(2)	-3(2)	4(2)
C4	14(3)	22(3)	22(3)	4(2)	-5(2)	-1(2)
C5	23(3)	31(3)	25(3)	0(3)	-6(2)	-7(2)
C6	13(3)	34(4)	29(3)	8(3)	-8(2)	-6(2)
C7	29(3)	45(4)	26(3)	6(3)	3(3)	-7(3)
C8	43(4)	43(4)	21(3)	1(3)	6(3)	-3(3)
C9	31(3)	38(4)	25(3)	-5(3)	-4(3)	-6(3)
C10	13(3)	33(3)	27(3)	5(2)	-8(2)	-7(2)
C11	15(3)	22(3)	22(3)	5(2)	-6(2)	-7(2)

Table S4. Anisotropic displacement parameters (Å^2×10^3) for b20260008_IH46F2TrpDUCu5. The anisotropic displacement factor exponent takes the form: $-2\pi^2[h^2a^{*2}U_{11}+2hka^*b^*U_{12}+\ldots]$.

Atom	Atom	Length/Å	Atom	Atom	Length/Å
F1	C8	1.362(8)	C3	C4	1.502(8)
F2	C10	1.364(7)	C4	C5	1.358(8)
O1	C1	1.261(7)	C4	C11	1.445(8)
O2	C1	1.257(7)	C6	C7	1.383(9)
N1	C5	1.386(8)	C6	C11	1.407(8)
N1	C6	1.381(8)	C7	C8	1.356(10)
N2	C2	1.484(6)	C8	C9	1.388(10)
C1	C2	1.518(7)	C9	C10	1.363(9)
C2	C3	1.533(8)	C10	C11	1.399(8)

Table S5. Bond lengths for b20260008_IH46F2TrpDUCu5.

Atom	Atom	Atom	Angle/˚	Atom	Atom	Atom	Angle/˚
C6	N1	C5	109.1(5)	N1	C6	C11	106.8(5)
O1	C1	C2	115.2(5)	C7	C6	C11	123.2(6)
O2	C1	O1	125.2(5)	C8	C7	C6	116.2(6)
O2	C1	C2	119.6(5)	F1	C8	C9	117.5(6)
N2	C2	C1	110.0(4)	C7	C8	F1	118.2(6)
N2	C2	C3	110.9(4)	C7	C8	C9	124.4(6)
C1	C2	C3	109.7(4)	C10	C9	C8	117.7(6)
C4	C3	C2	114.5(5)	F2	C10	C11	118.7(5)
C5	C4	C3	127.3(5)	C9	C10	F2	119.2(5)
C5	C4	C11	106.2(5)	C9	C10	C11	122.1(6)
C11	C4	C3	126.5(5)	C6	C11	C4	107.8(5)
C4	C5	N1	110.1(5)	C10	C11	C4	135.7(5)
N1	C6	C7	130.0(6)	C10	C11	C6	116.5(6)

Table S6. Bond angles for b20260008_IH46F2TrpDUCu5.

A	B	C	D	Angle/°	A	B	C	D	Angle/°
F1	C8	C9	C10	-178.4(6)	C5	N1	C6	C7	179.8(7)
F2	C10	C11	C4	0.8(10)	C5	N1	C6	C11	0.2(7)
F2	C10	C11	C6	-179.6(5)	C5	C4	C11	C6	0.7(6)
O1	C1	C2	N2	165.0(5)	C5	C4	C11	C10	-179.6(7)
O1	C1	C2	C3	-72.7(6)	C6	N1	C5	C4	0.3(7)
O2	C1	C2	N2	-15.3(7)	C6	C7	C8	F1	178.6(6)
O2	C1	C2	C3	106.9(6)	C6	C7	C8	C9	-1.3(11)
N1	C6	C7	C8	-179.1(7)	C7	C6	C11	C4	179.8(6)
N1	C6	C11	C4	-0.6(6)	C7	C6	C11	C10	0.0(9)
N1	C6	C11	C10	179.7(5)	C7	C8	C9	C10	1.5(11)
N2	C2	C3	C4	-71.3(6)	C8	C9	C10	F2	178.9(6)
C1	C2	C3	C4	167.0(5)	C8	C9	C10	C11	-0.9(10)
C2	C3	C4	C5	108.9(7)	C9	C10	C11	C4	-179.4(6)
C2	C3	C4	C11	-71.9(7)	C9	C10	C11	C6	0.2(9)
C3	C4	C5	N1	178.6(5)	C11	C4	C5	N1	-0.7(7)
C3	C4	C11	C6	-178.6(5)	C11	C6	C7	C8	0.5(10)
C3	C4	C11	C10	1.1(10)					

Table S7. Torsion angles for b20260008_IH46F2TrpDUCu5.

Atom	*x*	*y*	*z*	U(eq)
H1	608.29	419.33	3987.72	35
H2C	6704.14	6871.35	3272.22	20
H3A	6933.29	1172.98	3191.86	24
H3B	8440.11	2724.63	3499.72	24
H5	2977.78	-391.59	3431.67	32
H7	653.39	3814.97	4611.85	40
H9	6119.7	8794.5	4597.09	38
H2	9980(120)	4260(50)	2357(12)	38
H2A	5400(130)	5030(60)	2577(8)	38
H2B	4800(120)	3410(50)	2786(13)	38

Table S8. Hydrogen atom coordinates (Å×10^4) and isotropic displacement parameters (Å^{2}x10^3) for b20260008_IH46F2TrpDUCu5.

11.4. Experimental

A suitable single crystal of $C_{11}H_{10}F_2N_2O_2$ [b20260008_IH46F2TrpDUCu5] was selected and mounted on a SuperNova, Dual, Cu at home/near, HyPix diffractometer. The crystal was kept at 169.99 K during data collection. Using Olex2[38], the structure was solved with the SHELXT[39] structure solution program using Intrinsic Phasing and refined with the SHELXL[40] refinement package using Least Squares minimisation.

11.5. Crystal structure determination of [b20260008_IH46F2TrpDUCu5]

Crystal data for $C_{11}H_{10}F_2N_2O_2$ (M =240.21 g/mol): monoclinic, space group P21/n (no. 14), a = 5.86452(16) Å, b = 5.00639(17) Å, c = 35.2442(9) Å, β = 90.283(3)°, V = 1034.76(5) Å^3, Z = 4, T =169.99(10) K, μ(Cu Kα) = 1.141 mm^{-1}, $Dcalc$ = 1.542 g/cm^3, 17435 reflections measured (5.014° ≤ 2Θ ≤ 137.976°), 1912 unique (R_{int} = 0.1627, R_{sigma} = 0.0744) which were used in all calculations. The final R_1 was 0.1223 (I > 2σ(I)) and wR_2 was 0.3513 (all data).

11.6. Refinement model description

Number of restraints - 6, number of constraints - unknown.

Details:

Fixed Uiso at 1.2 times of: all C(H) groups, all C(H,H) groups, all C(H,H,H,H) groups, all N(H) groups

Restrained distances:

N2-H2B = N2-H2A, 0.89 with sigma of 0.02

O2-H2, 0.89 with sigma of 0.02

H2A-H2_$1, 2.15 with sigma of 0.02

H2_$2-H2B, 2.15 with sigma of 0.02

H2A-H2B, 1.155 with sigma of 0.02

Ternary CH refined with riding coordinates: C2(H2C)

Secondary CH2 refined with riding coordinates: C3(H3A, H3B)

Aromatic/amide H refined with riding coordinates: N1(H1), C5(H5), C7(H7), C9(H9)

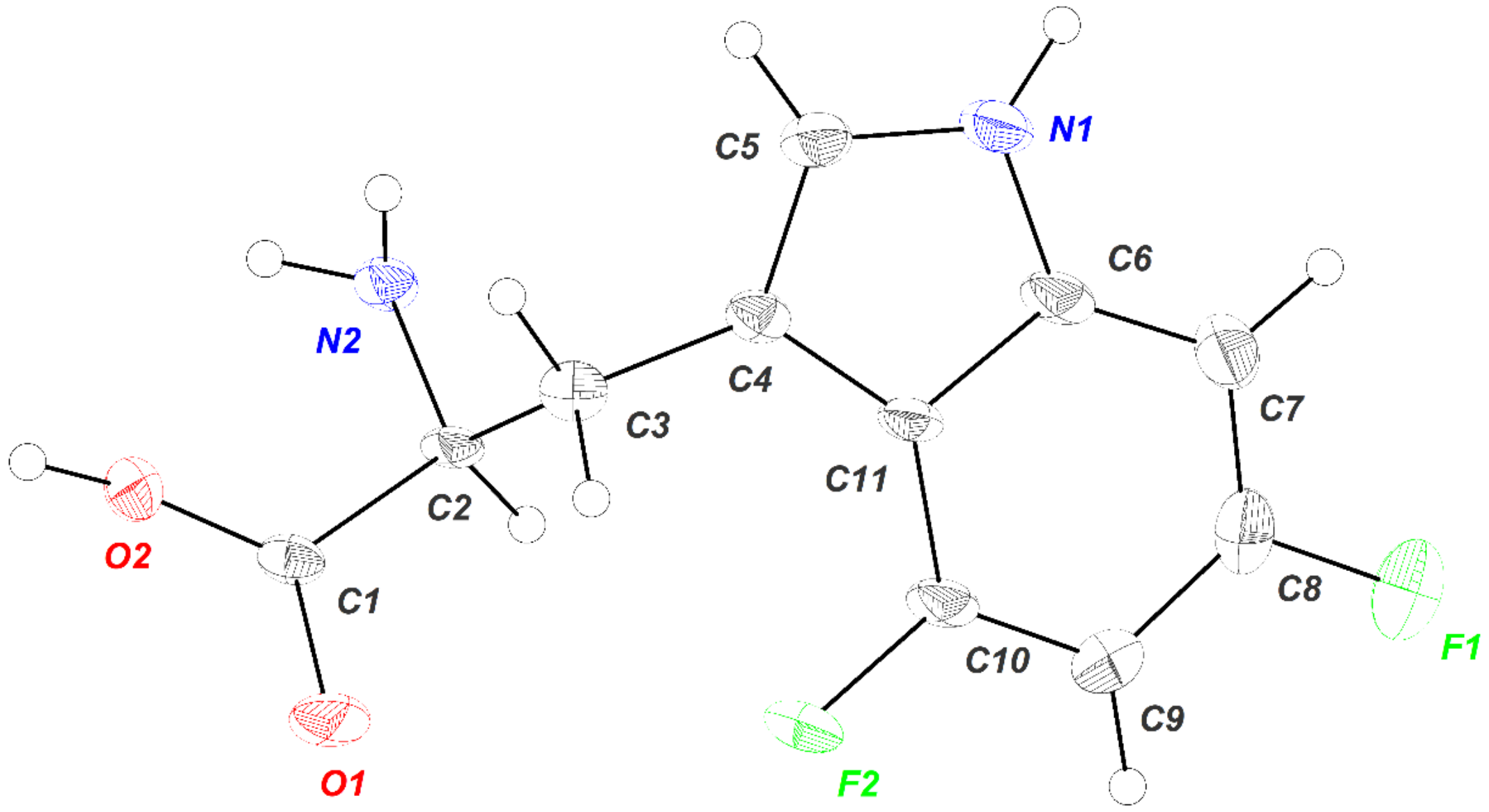

Figure **S11**: Thermal ellipsoid plot (50% contour probability levels) for crystal structure CCDC 2538127.

CCDC entry with accession number 2538127 (4,6-F_2-Trp) contains the supplementary crystallographic data for this paper. These data can be obtained free of charge via www.ccdc.cam.ac.uk/data_request/cif, or by emailing data_request@ccdc.cam.ac.uk, or by contacting The Cambridge Crystallographic Data Centre, 12 Union Road, Cambridge CB2 1EZ, UK; fax +44 1223 336033.

12. Protein 2D ^{19}F–^{19}F NOESY Spectra

12.1. 5,6-F_2-Trp CypA spectra.

2D ^{19}F–^{19}F NOESY spectra were acquired for 5,6-F_2-Trp CypA using increasing mixing times (1 ms – 700 ms) and a recycle delay (D1) of 2.5 seconds, as shown below (Figure S12). Some sample degradation was observed over time, resulting in the appearance of a signal at –66.8 Hz, between the 5F and 6F resonances. The ^{19}F 1D spectrum is shown (Figure S13) for further illustration. Fortunately, this signal did not overlap with the protein resonances and therefore did not affect the NOE measurements. Similar impurity signals resulting from sample degradation have been reported for other fluorine-labeled proteins.[15]

We also tested whether a shorter D1 would still allow sufficient magnetization recovery since this reduces the overall experimental time. Experiments recorded for 5,6-F_2-Trp CypA with a D1 of 1.5 s yielded essentially identical spectra (Figure S14) to those obtained with the 2.5 s delay (Figure S12).

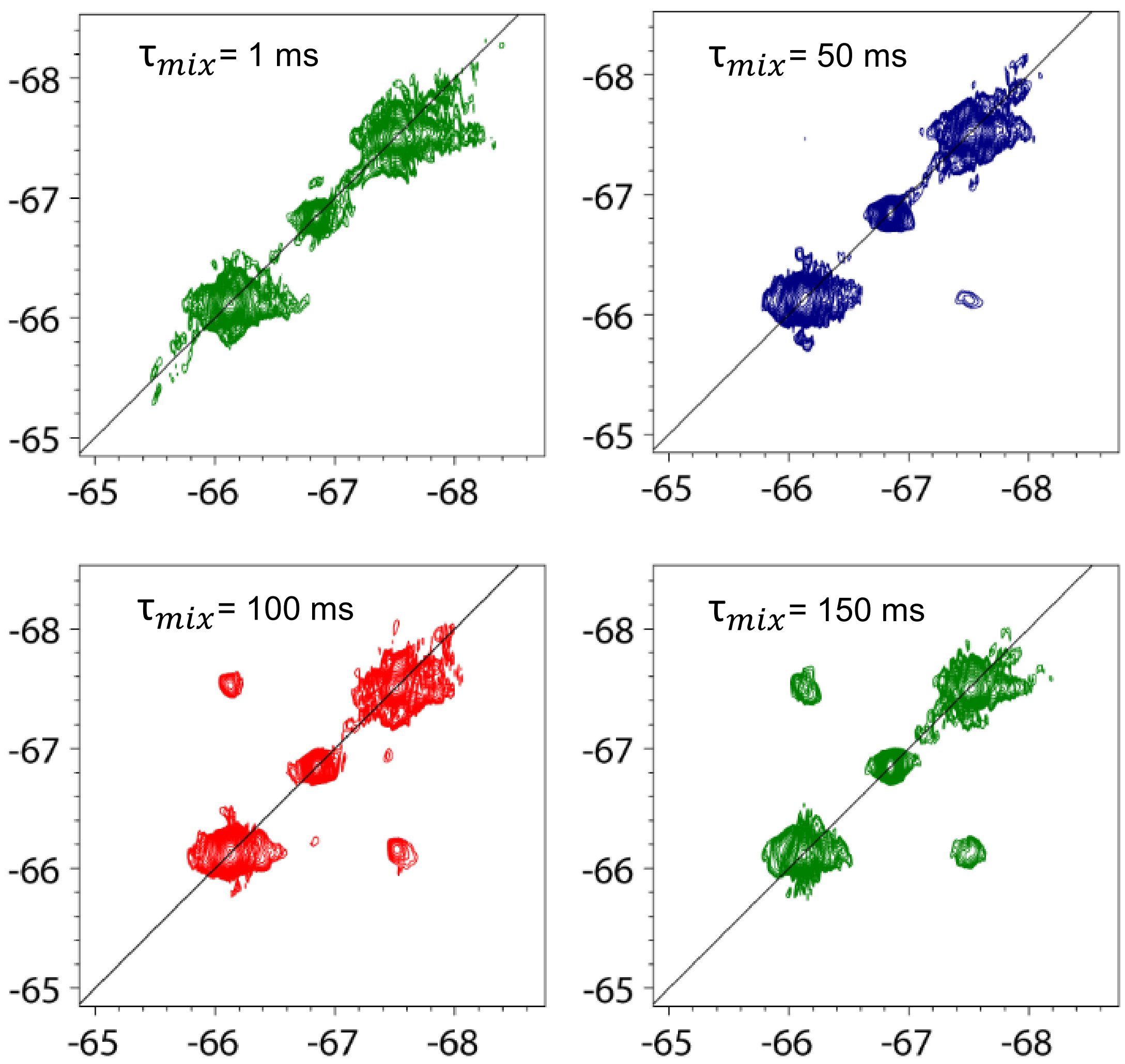

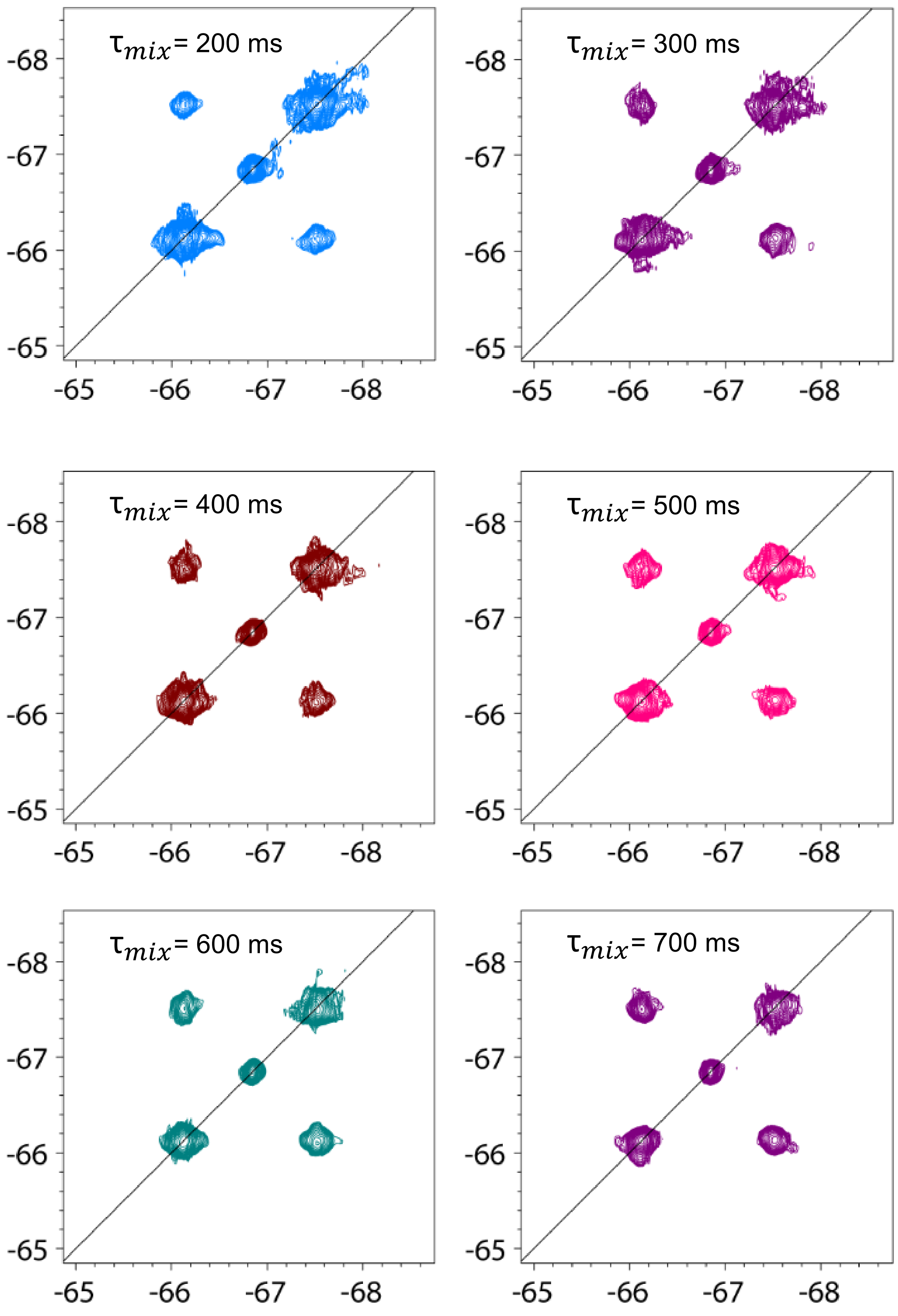

Figure S12: 2D ^{19}F–^{19}F NOESY spectra of 5,6-F_2-Trp CypA using a D1 of 2.5 s.

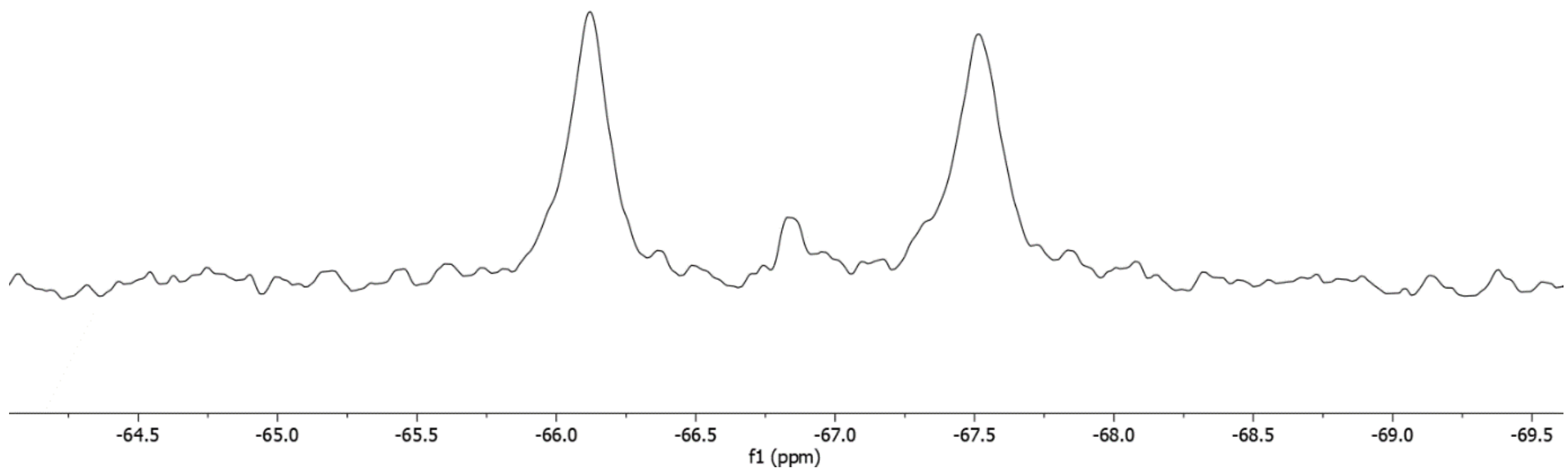

Figure S13: 1D ^{19}F NMR spectra of 5,6-F_2-Trp CypA after completion of the series of NOESY spectra.

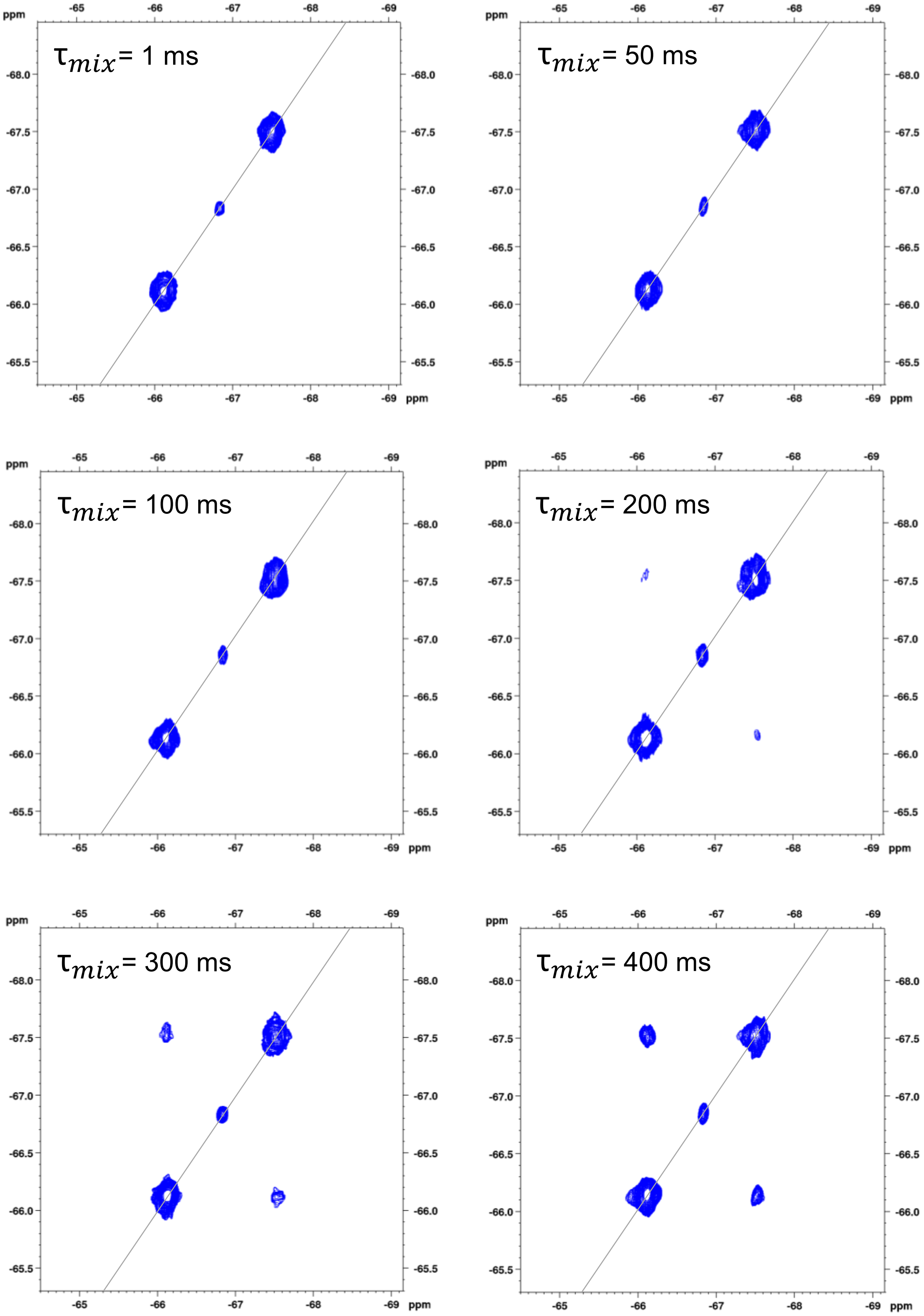
τmix= 1 ms
τmix= 50 ms
τmix= 100 ms
τmix= 200 ms
τmix= 300 ms
τmix= 400 ms
ppm
-65
-66
-67
-68
-69
-68.0
-67.5
-67.0
-66.5
-66.0
-65.5

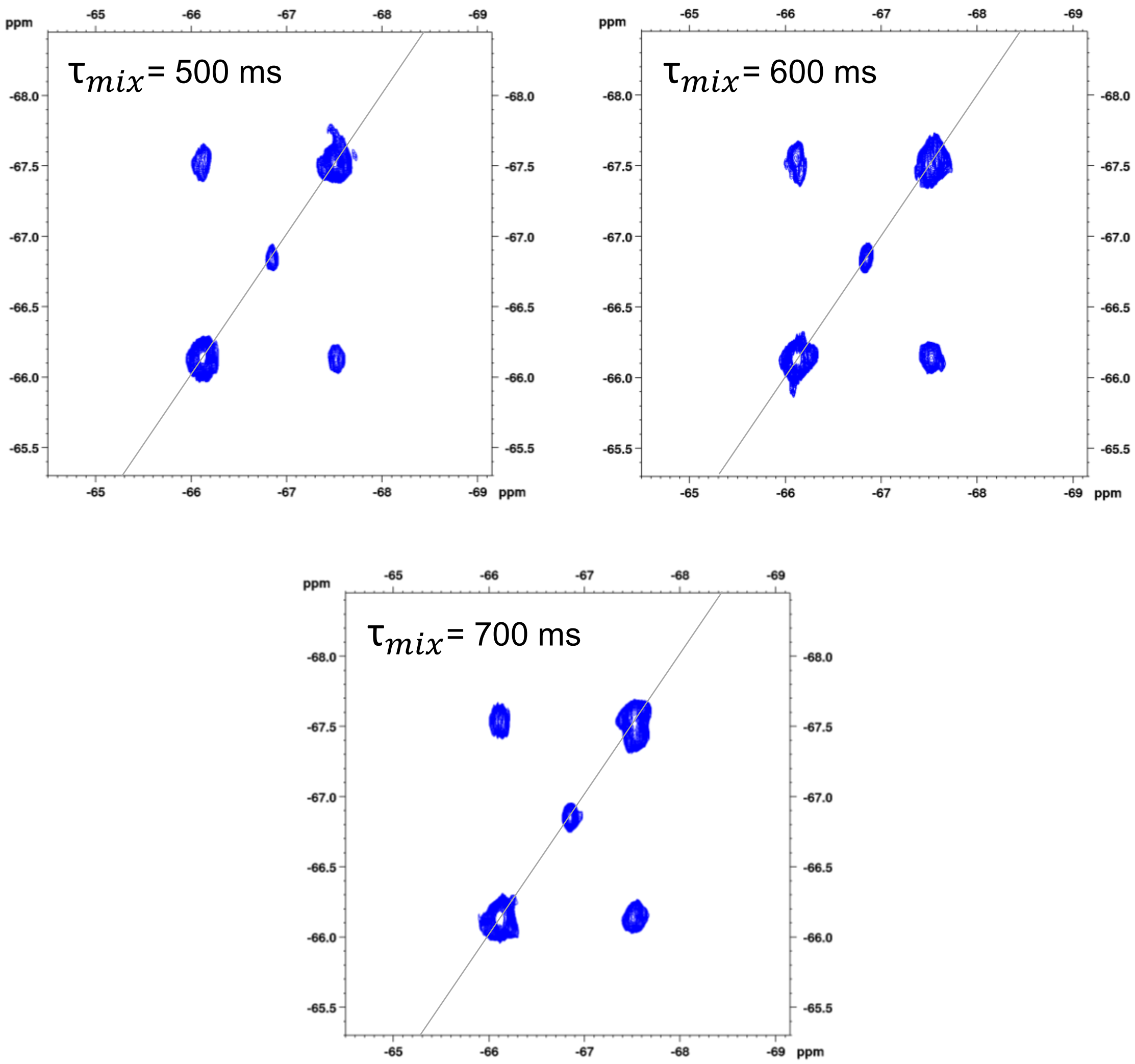

Figure S14: 2D ^{19}F–^{19}F NOESY spectra of 5,6-F_2-Trp CypA using a D1 of 1.5 s.

We also explored the impact of ^{1}H decoupling on the NOE intensities (Figure S15). The signal heights in the ^{1}H-decoupled spectrum are reduced by ~30 % compared to the non-decoupled one. Similar intensity changes have previously been reported and were attributed to the slowed ^{19}F magnetization recovery process, particularly in large proteins.[41]

Finally, for completeness' sake, we also recorded NOESY build-up curves for 5,6-F_2-Trp CypA with ^{1}H decoupling and a 1.5 s D1 recycle delay (Figure S16). Essentially identical build-ups were obtained as seen for the non-decoupled spectra.

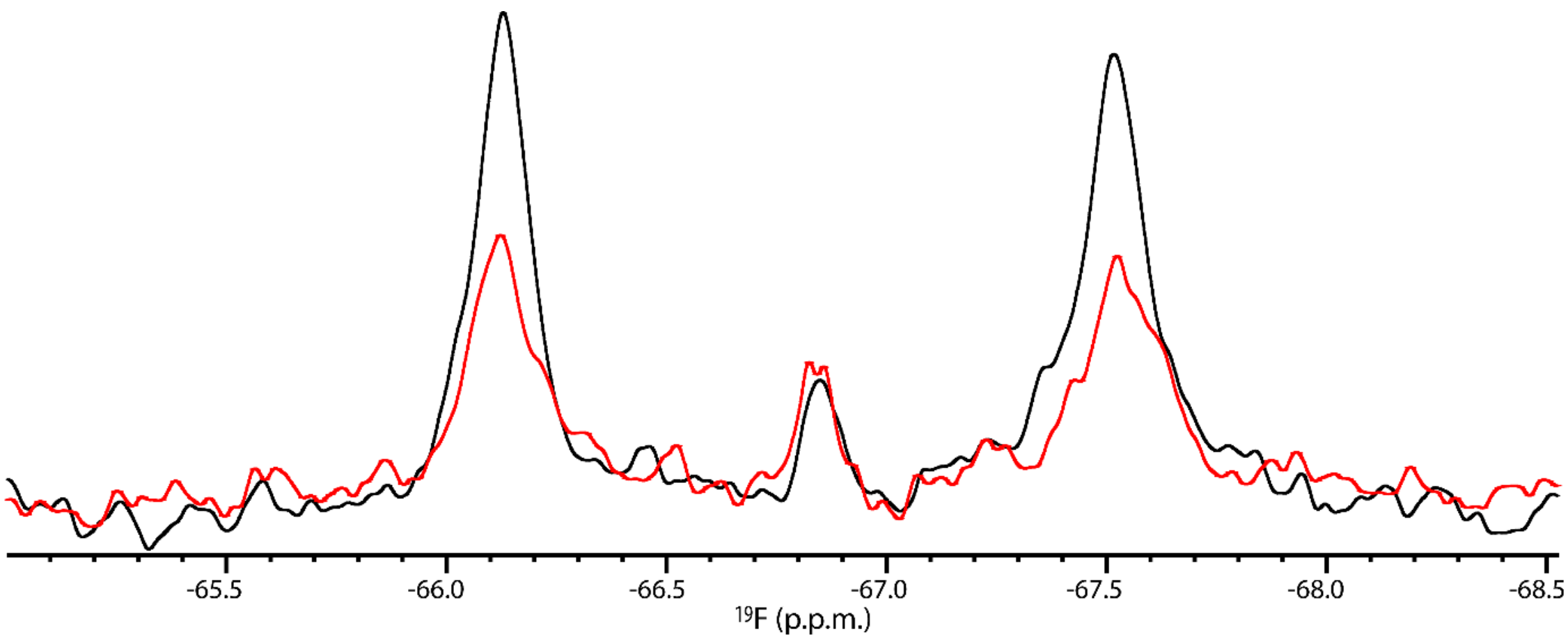

Figure S15: Overlaid ^{19}F NMR spectra of 5,6-F_2-Trp CypA acquired with (red trace) and without (black trace) ^{1}H decoupling.

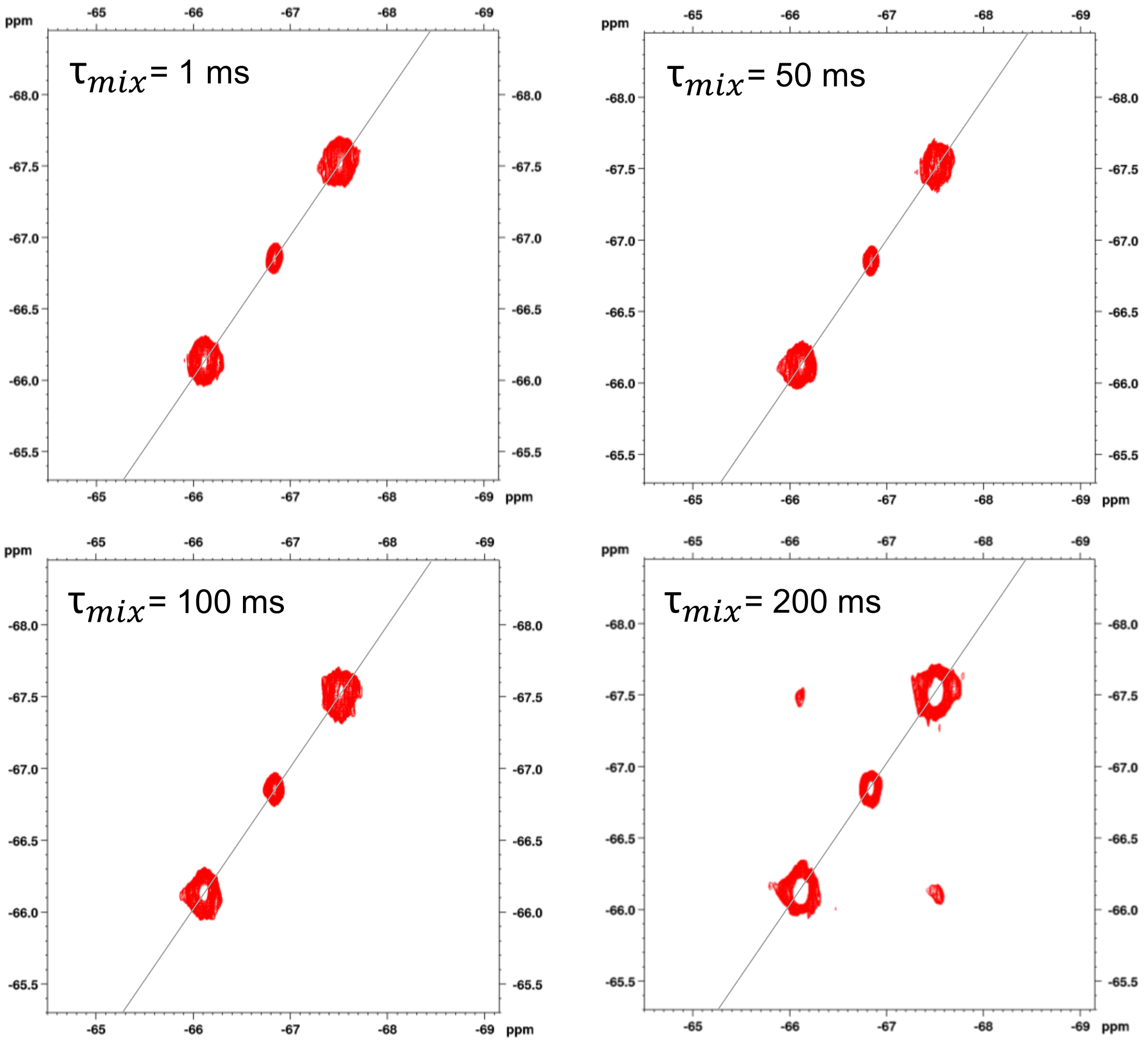

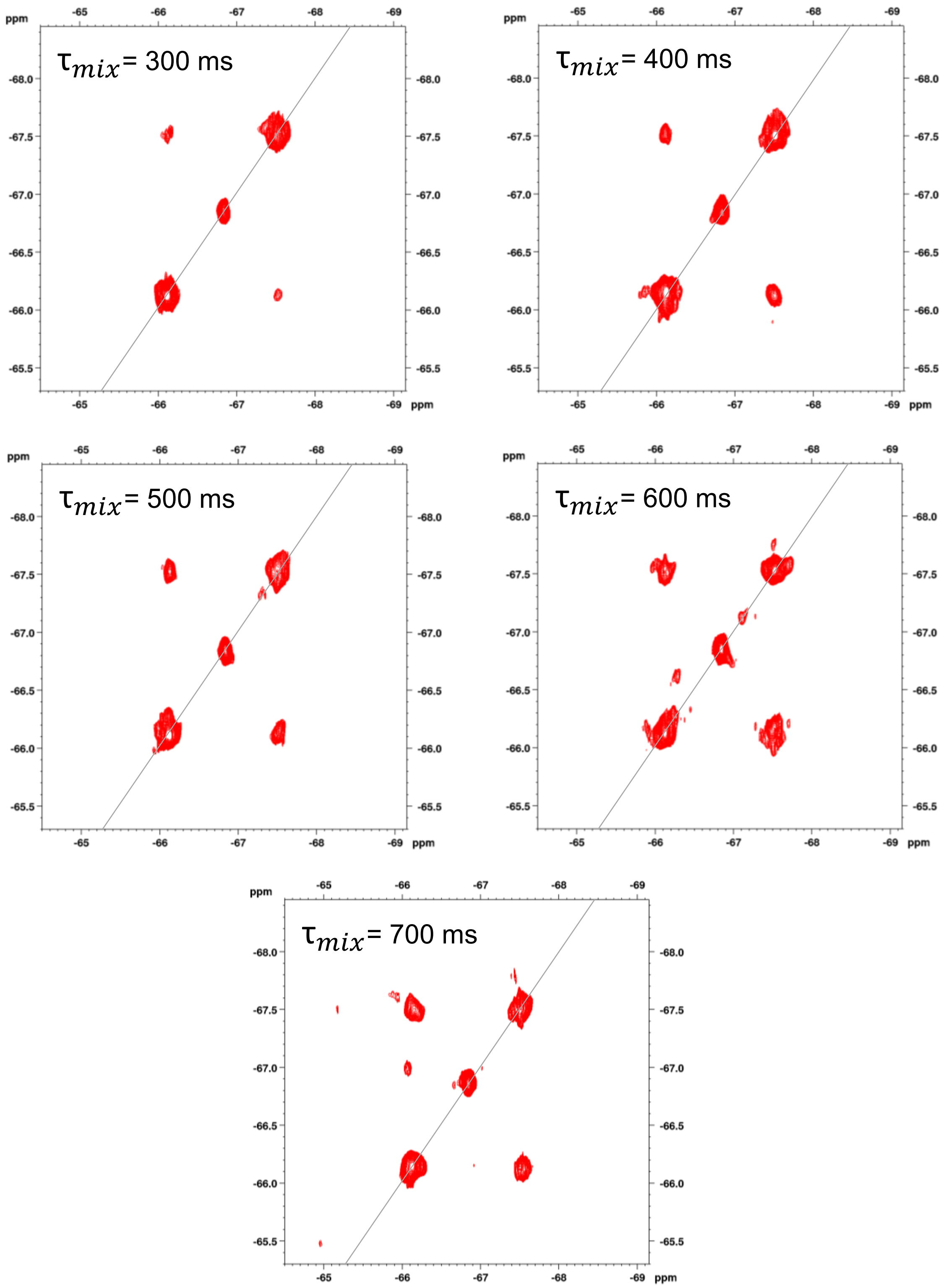

Figure S16: 2D ^{19}F–^{19}F NOESY spectra of 5,6-F_2-Trp CypA with ^{1}H decoupling and a D1 of 1.5 s.

12.2. 4,5-F_2-Trp CypA spectra.

2D ^{19}F–^{19}F NOESY spectra were acquired for 4,5-F_2-Trp CypA at increasing mixing times (from 1 – 800 ms), using a D1 of 2.6 seconds.

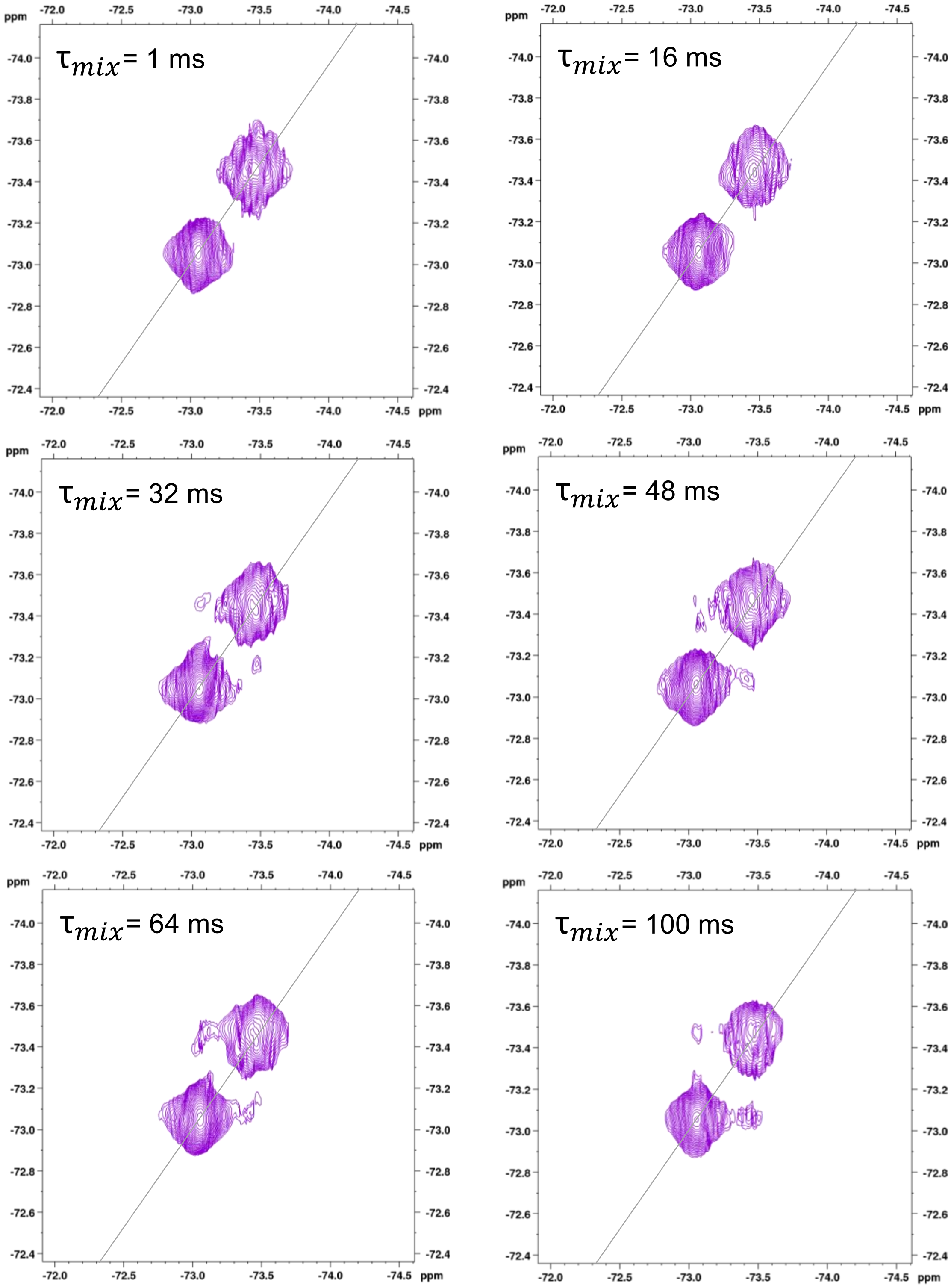

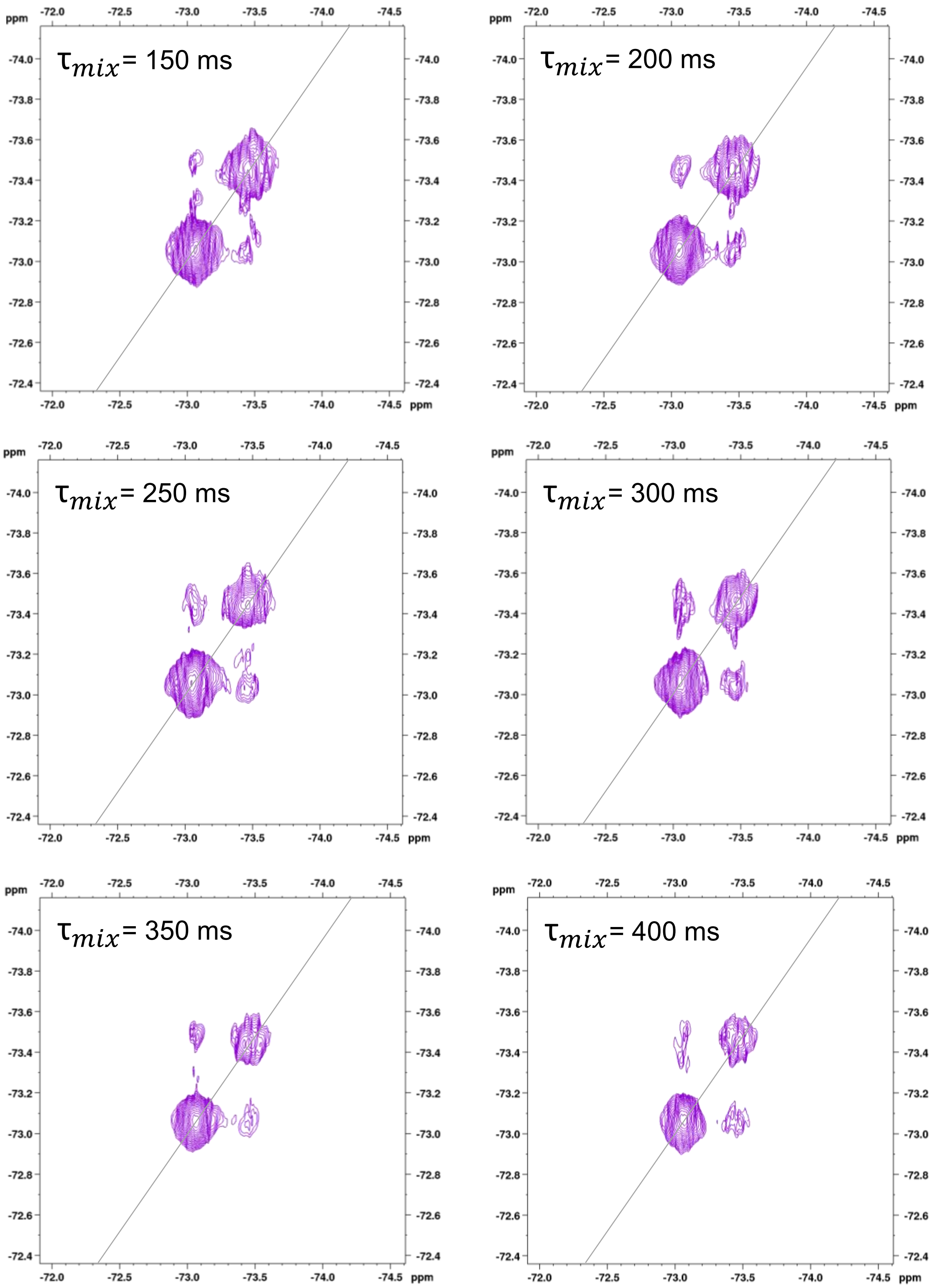

τmix= 150 ms
τmix= 200 ms
τmix= 250 ms
τmix= 300 ms
τmix= 350 ms
τmix= 400 ms
ppm
-72.0
-72.5
-73.0
-73.5
-74.0
-74.5
-74.0
-73.8
-73.6
-73.4
-73.2
-73.0
-72.8
-72.6
-72.4

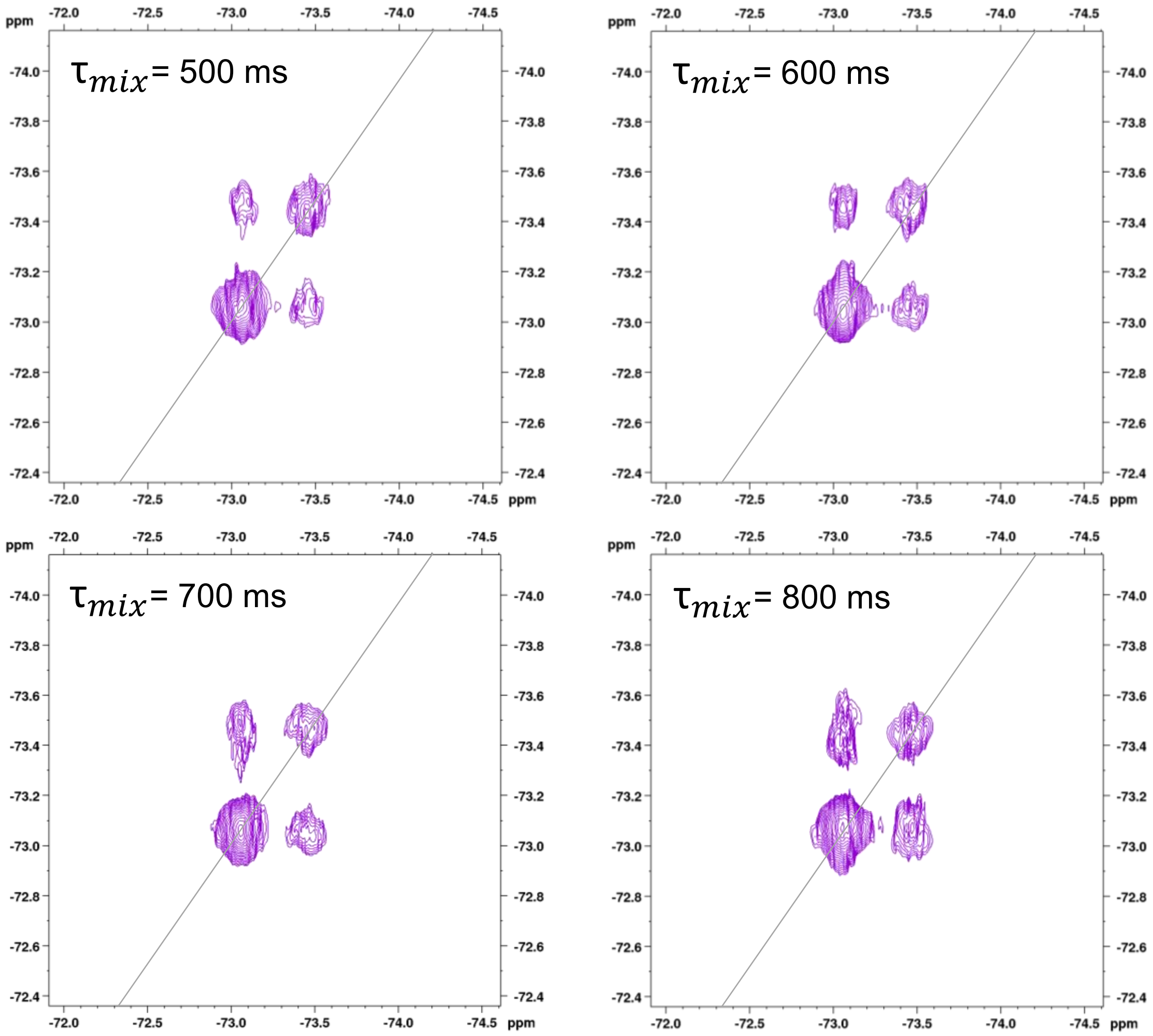

Figure S17: 2D ^{19}F–^{19}F NOESY spectra of 4,5-F_2-Trp CypA using a D1 of 2.6 s.

12.3. 4,6-F_2-Trp CypA spectra.

2D ^{19}F–^{19}F NOESY spectra were acquired for 4,6-F_2-Trp CypA at increasing mixing times (from 100 to 1000 ms), using a D1 of 2.5 seconds. Here again, some degradation was noted, with resonances appearing at –41.4 Hz and –43.5 Hz (Figure S18). These impurity signals did not overlap with the protein resonances and therefore did not affect the NOE measurements.

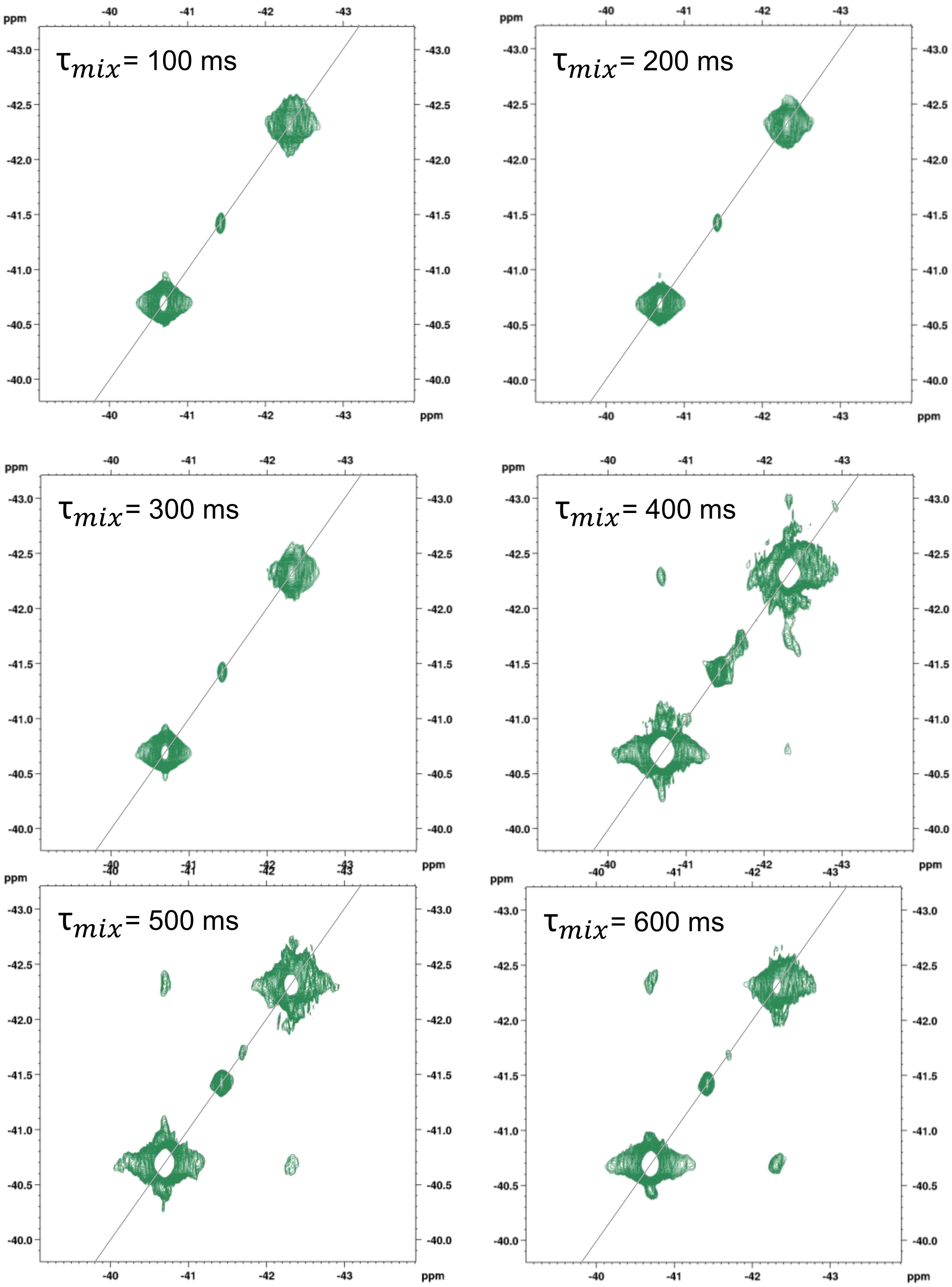

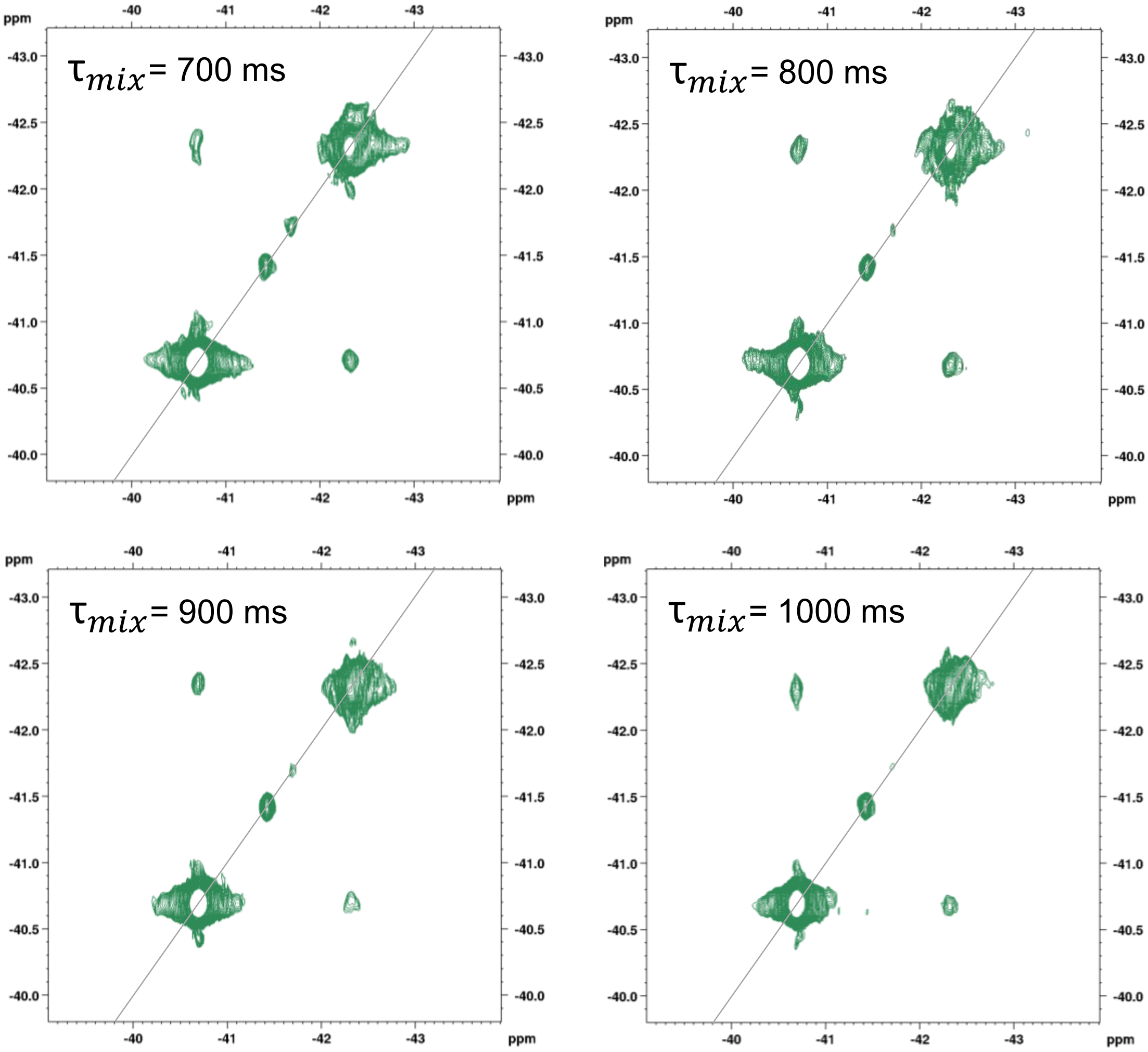

Figure S18: 2D ^{19}F–^{19}F NOESY spectra of 4,6-F_2-Trp CypA using a D1 of 2.5 seconds.

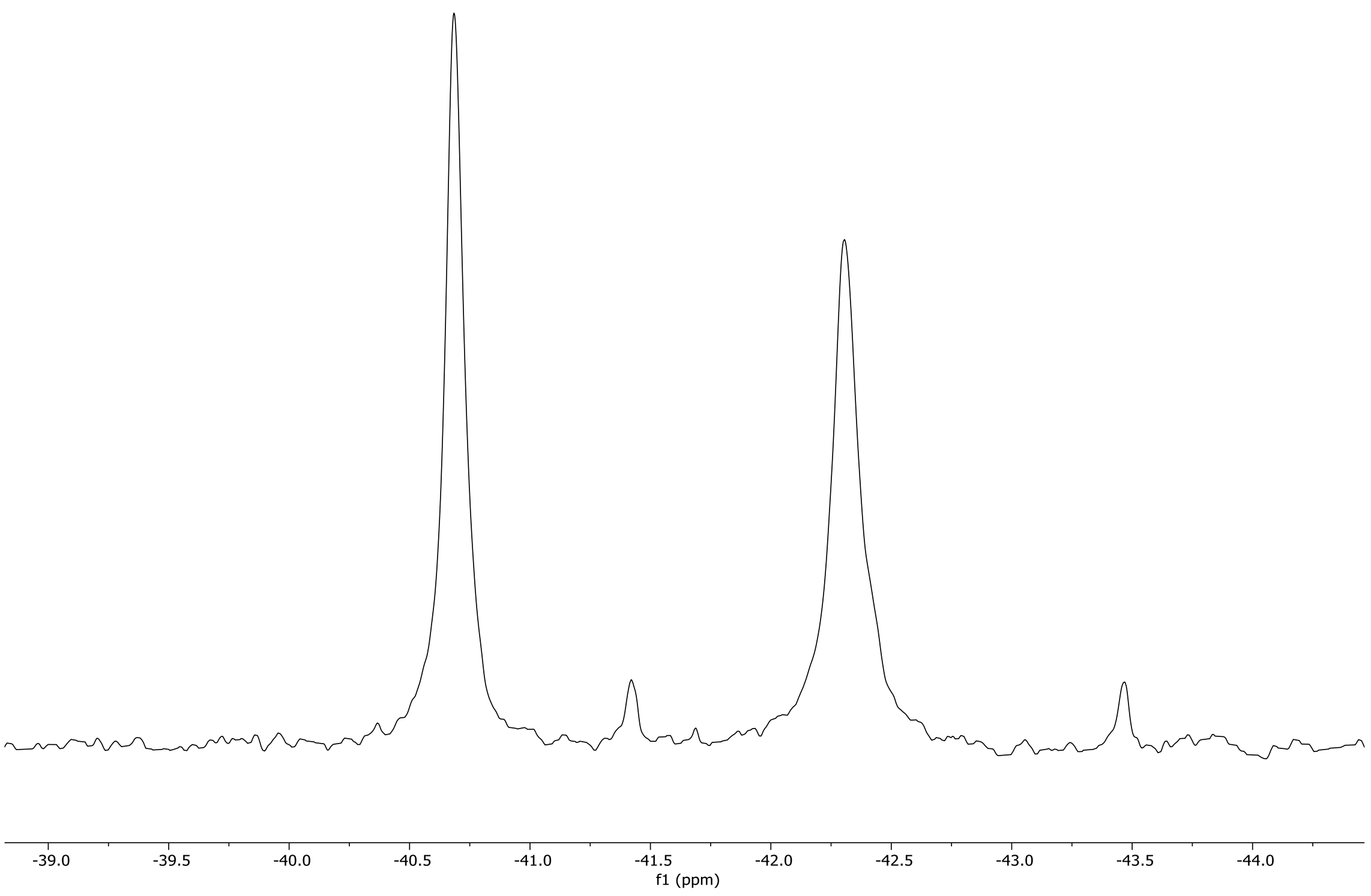

Figure S19: 1D ^{19}FNMR spectra of 4,6-F_2-Trp CypA.

13. NOE data analysis

Shown below are the individual plots from which the cross-relaxation rates reported in Table 1 of the main manuscript were extracted.

	Normalization	Fitted to:	
Protein	$\frac{I_{ij}\,(\tau_{mix})}{I_{ii}\,(\tau_{mix})}$	$I_{NOE}(\tau_{mix}) = Bx$	
**5,6**-F_2-Trp CypA	$\sigma_{5\to6}$= 0.951 ± 0.021		**A**
	$\sigma_{6\to5}$= 0.840 ± 0.020		**B**
**4,5**-F_2-Trp CypA	$\sigma_{4\to5}$= 0.633 ± 0.041		**C**
	$\sigma_{5\to4}$= 1.424 ± 0.045		**D**
**4,6**-F_2-Trp CypA	$\sigma_{4\to6}$= 0.1102 ± 0.0063		**E**
	$\sigma_{6\to4}$= 0.0362 ± 0.0025		**F**

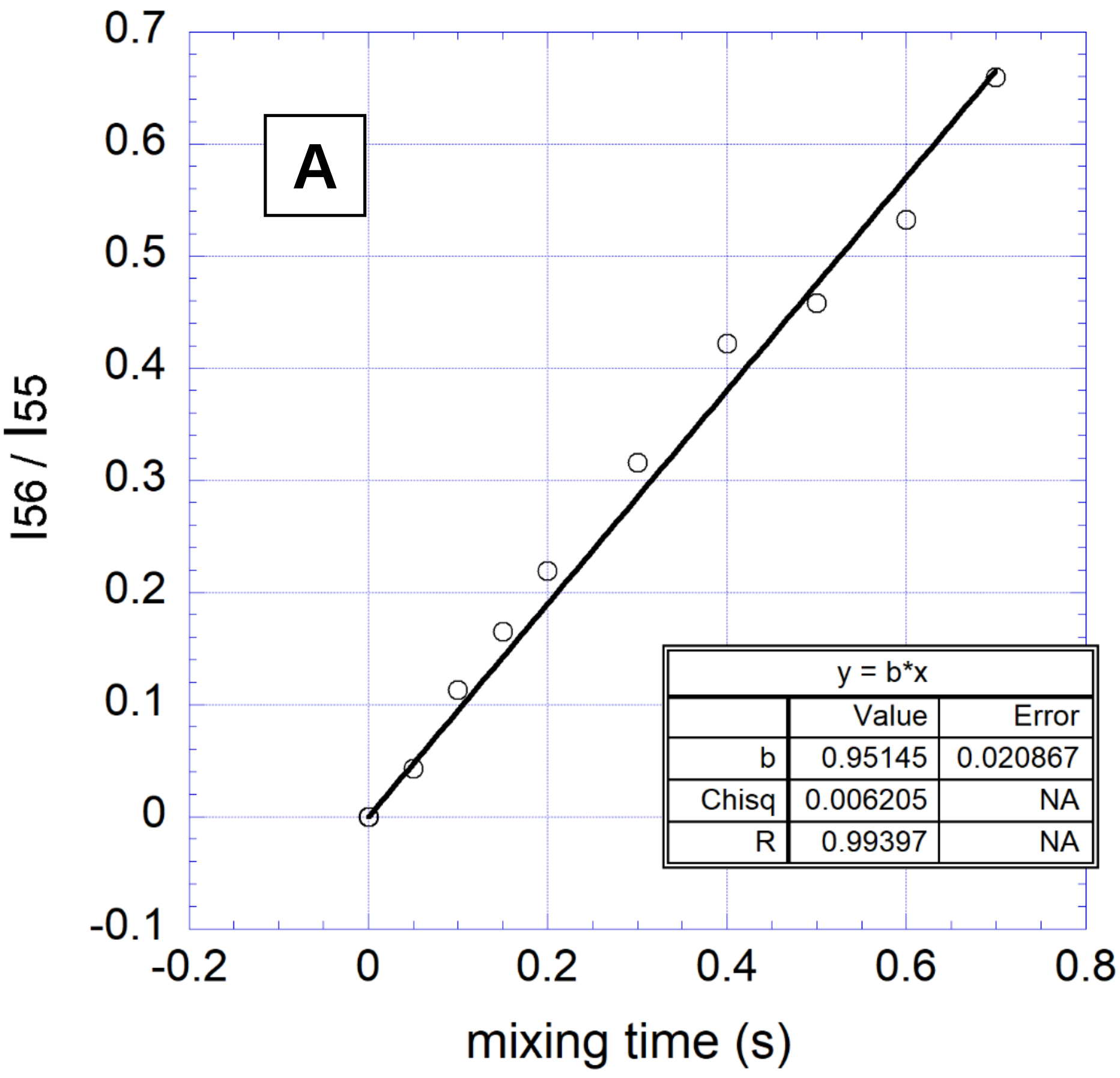

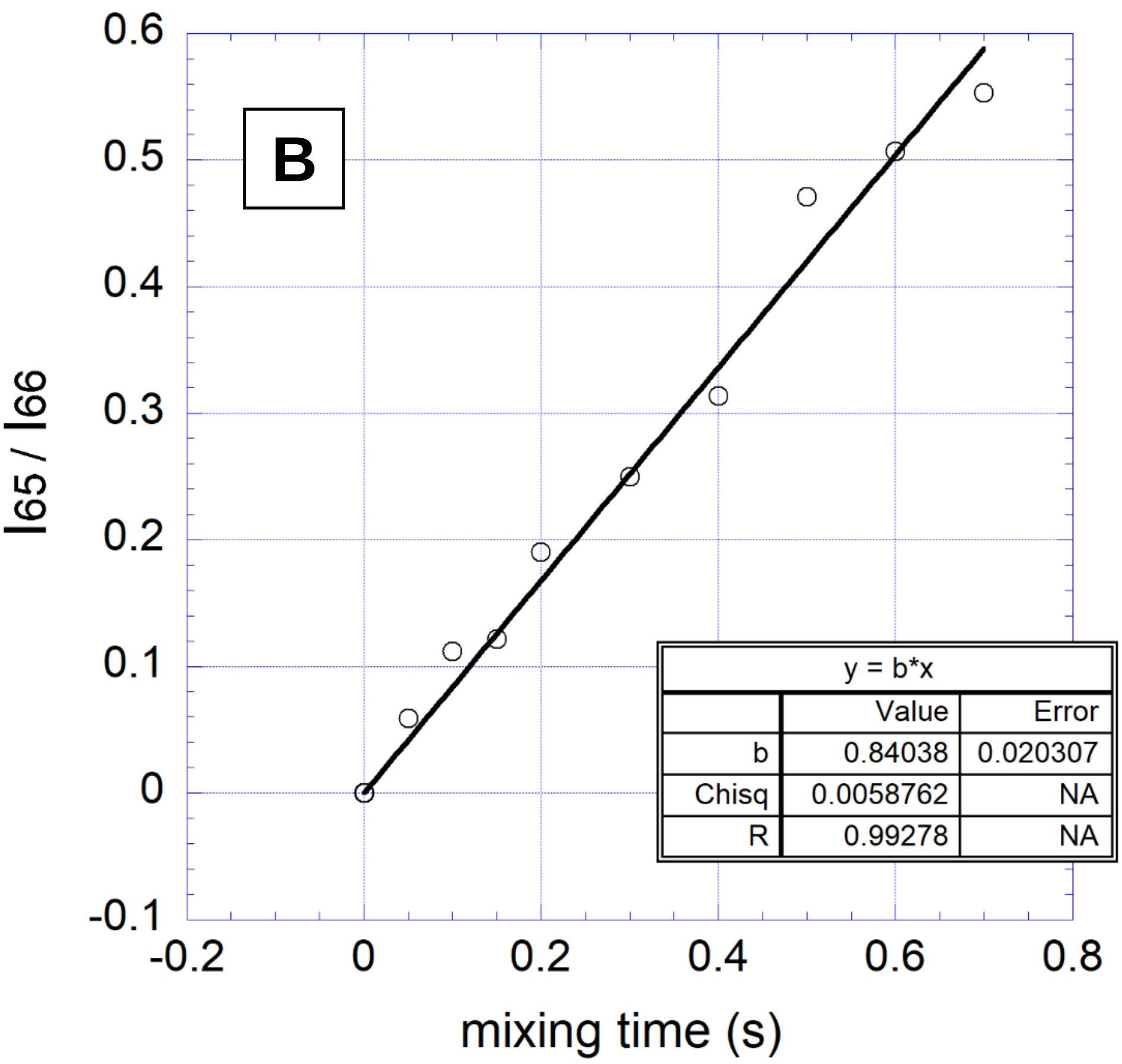
B
I65 / I66
mixing time (s)
0.6
0.5
0.4
0.3
0.2
0.1
0
-0.1
-0.2
0
0.2
0.4
0.6
0.8
y = b*x
Value
Error
b
0.84038
0.020307
Chisq
0.0058762
NA
R
0.99278
NA

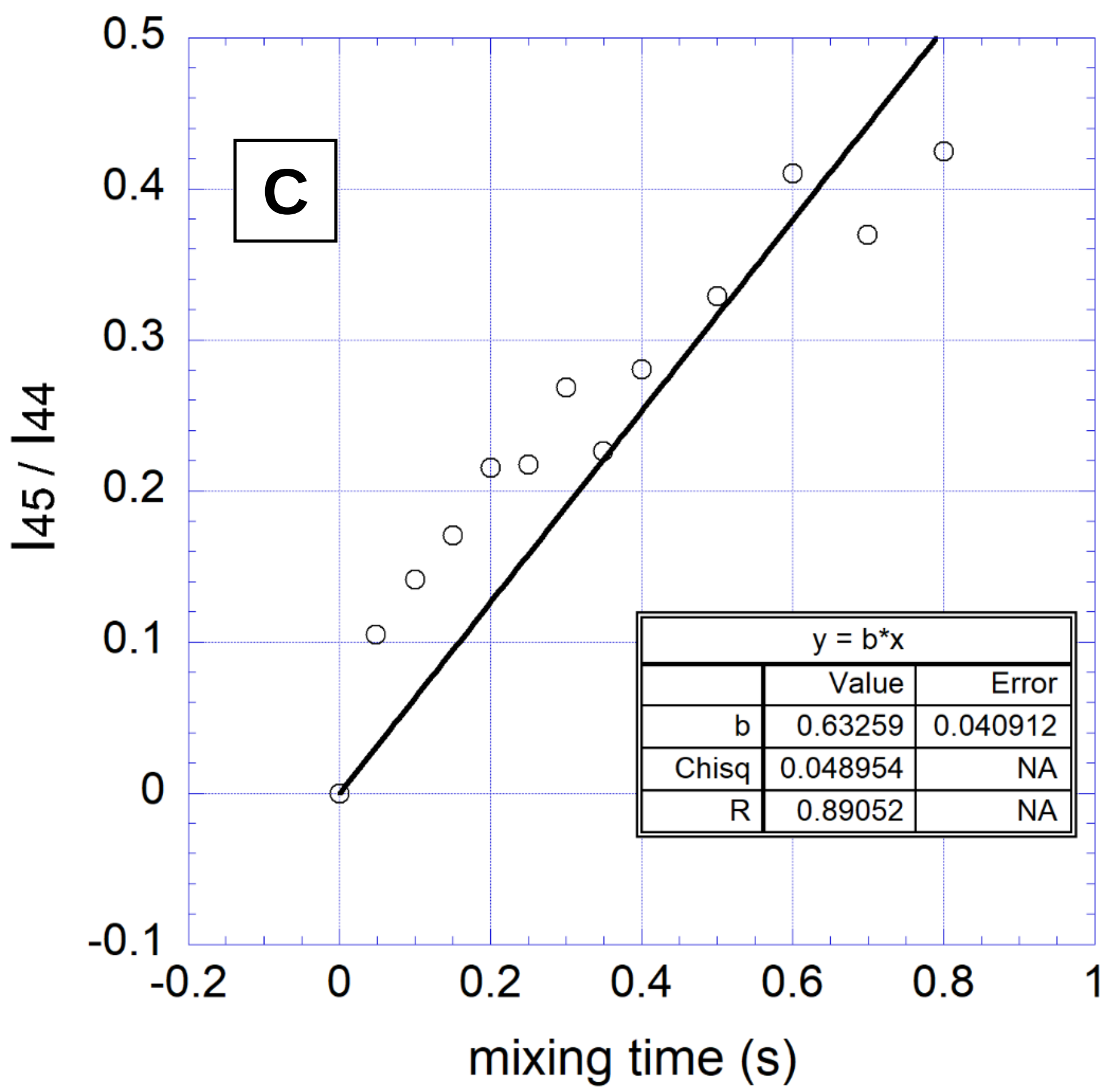
C
I45 / I44
mixing time (s)
0.5
0.4
0.3
0.2
0.1
0
-0.1
-0.2
0
0.2
0.4
0.6
0.8
1
y = b*x
Value
Error
b
0.63259
0.040912
Chisq
0.048954
NA
R
0.89052
NA

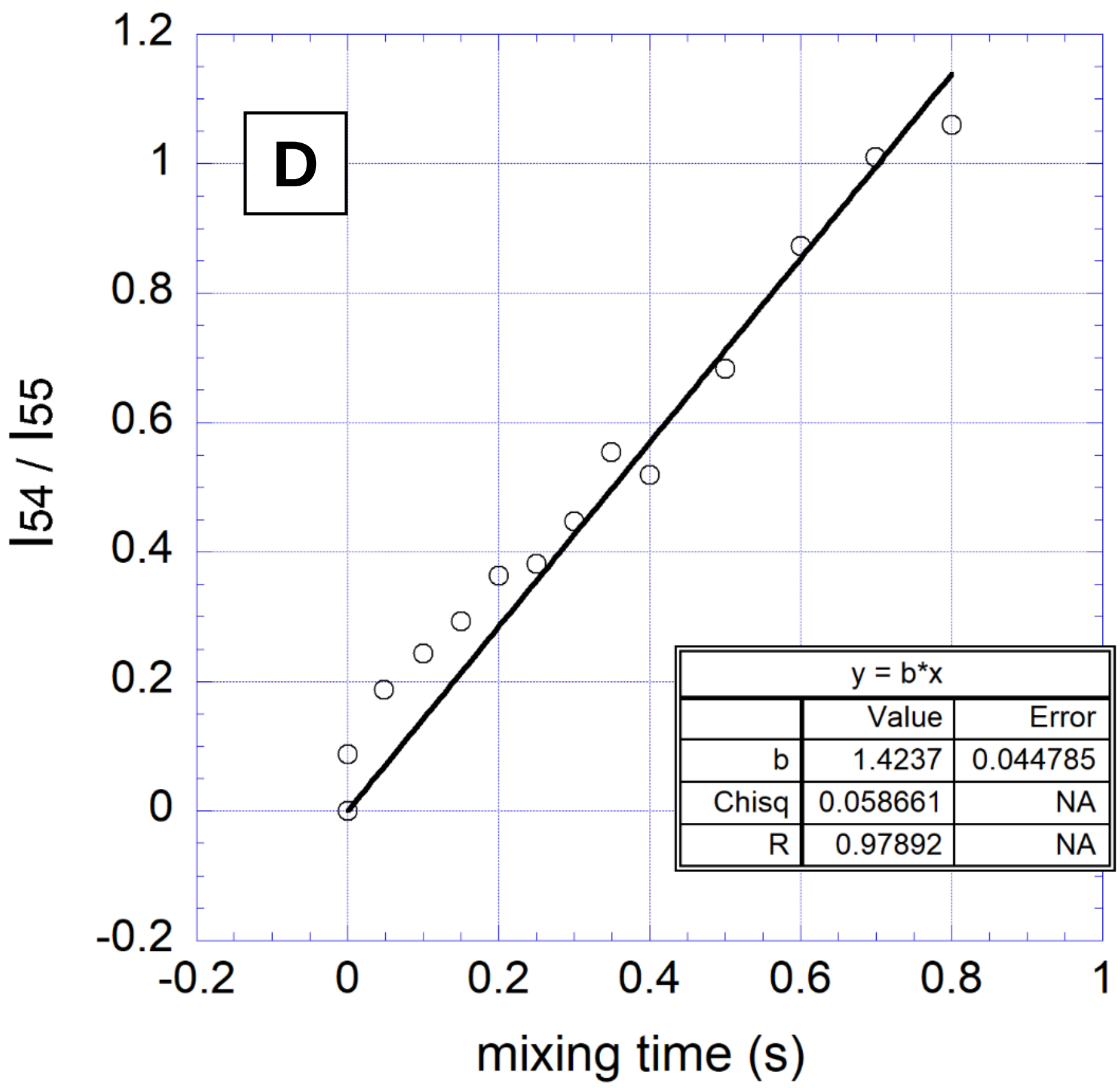
D
1.2
1
0.8
0.6
0.4
0.2
0
-0.2
I54 / I55
-0.2
0
0.2
0.4
0.6
0.8
1
mixing time (s)
y = b*x
Value Error
b 1.4237 0.044785
Chisq 0.058661 NA
R 0.97892 NA

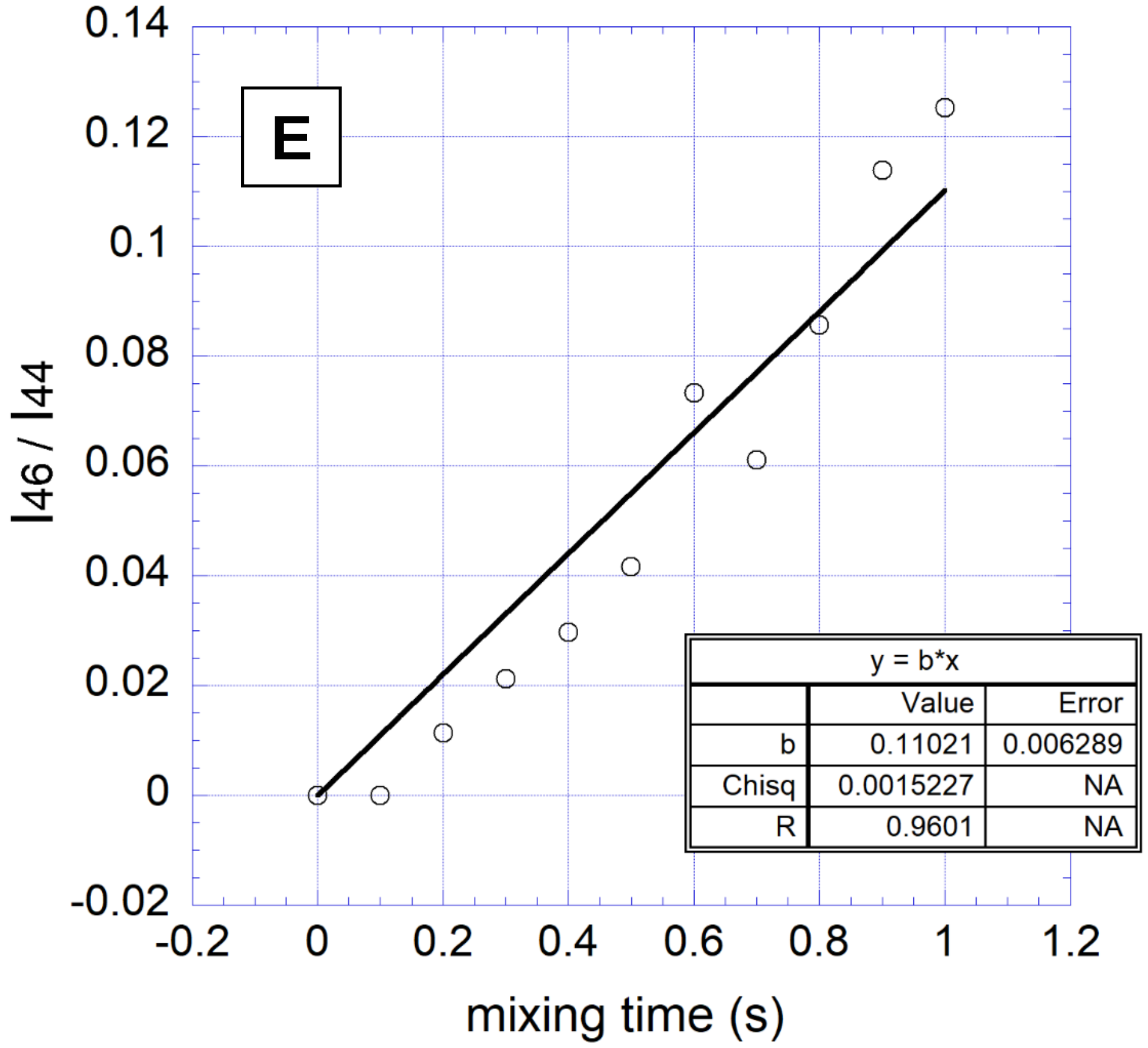
E
0.14
0.12
0.1
0.08
0.06
0.04
0.02
0
-0.02
I46 / I44
-0.2
0
0.2
0.4
0.6
0.8
1
1.2
mixing time (s)
y = b*x
Value Error
b 0.11021 0.006289
Chisq 0.0015227 NA
R 0.9601 NA

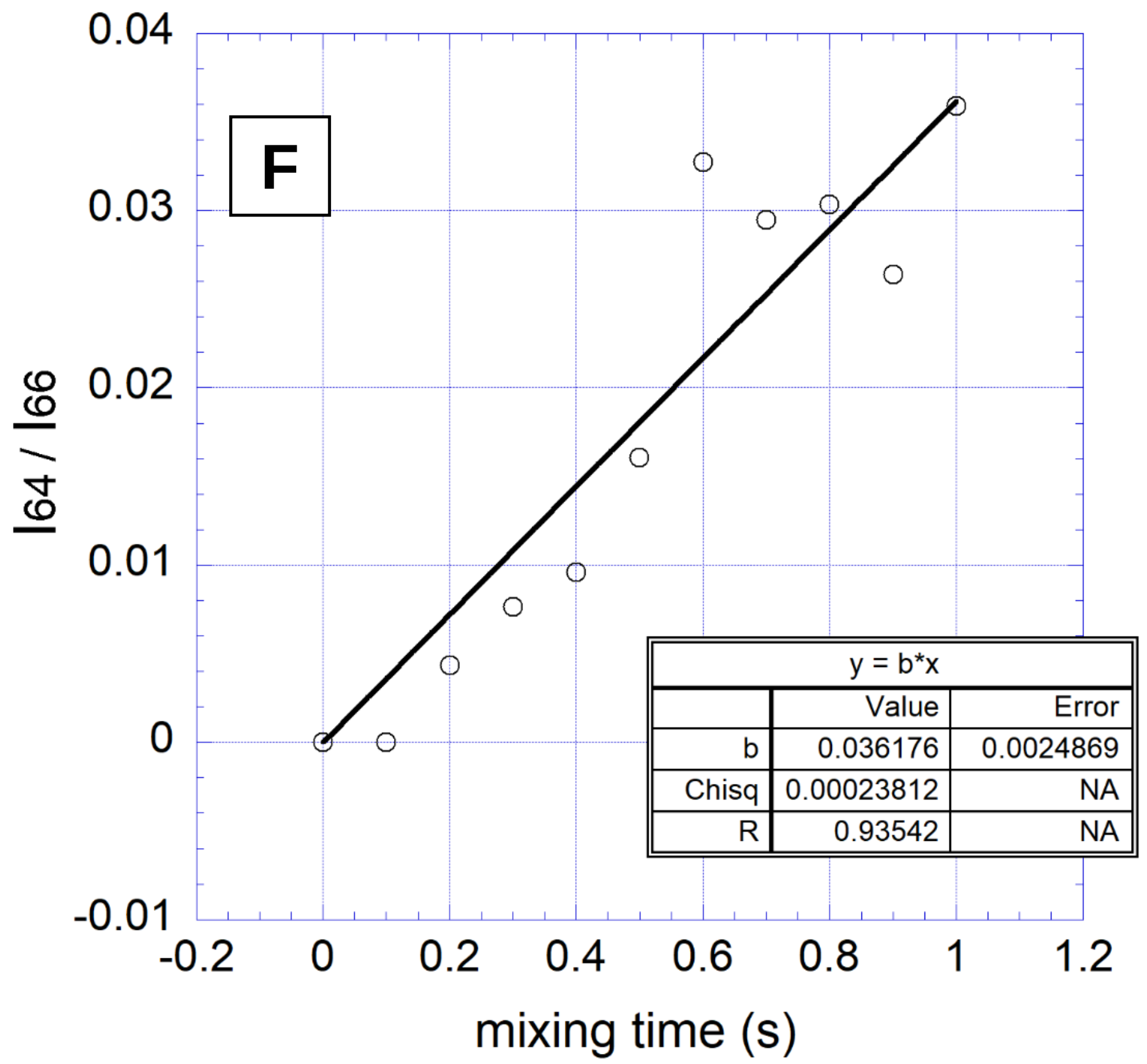

Protein	Normalization	Fitted to:	
	$\frac{I_{ij}(\tau_{mix})}{I_{ii}(\tau_{mix})}$	$I_{NOE}(\tau_{mix}) = \frac{e^{2x}-1}{e^{2x}+1}$	
**5,6**-F2-Trp CypA	$\sigma_{5\to6}$= 1.063 ± 0.021		**G**
	$\sigma_{6\to5}$= 0.914 ± 0.023		**H**
**4,5**-F2-Trp CypA	$\sigma_{4\to5}$= 0.680 ± 0.044		**I**
	$\sigma_{5\to4}$= 1.888 ± 0.133		**J**
**4,6**-F2-Trp CypA	$\sigma_{4\to6}$= 0.1105 ± 0.0064		**K**
	$\sigma_{6\to4}$= 0.0362 ± 0.0025		**L**

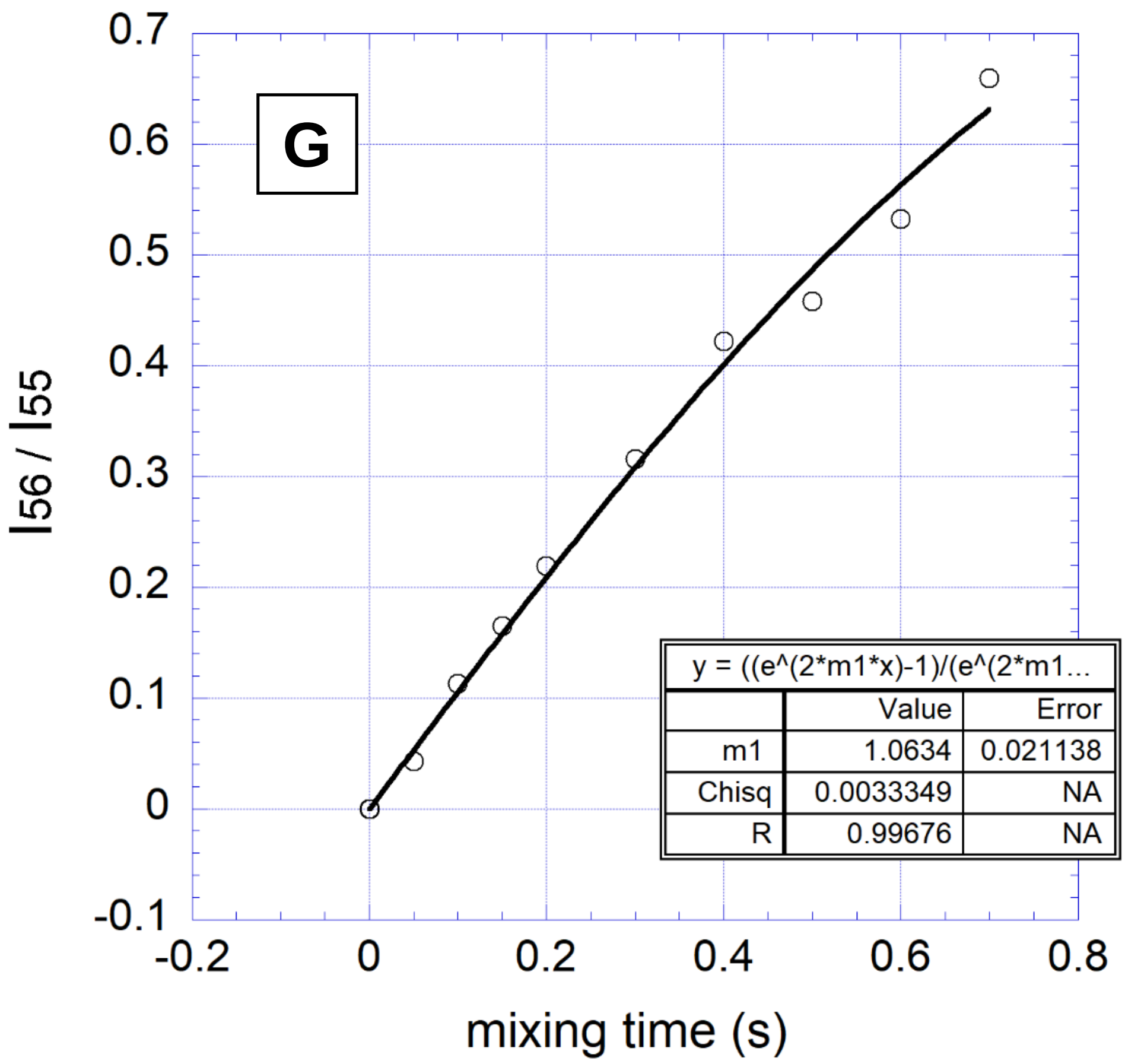
G
I_{56} / I_{55}
mixing time (s)
y = ((e^(2*m1*x)-1)/(e^(2*m1...
Value Error
m1 1.0634 0.021138
Chisq 0.0033349 NA
R 0.99676 NA

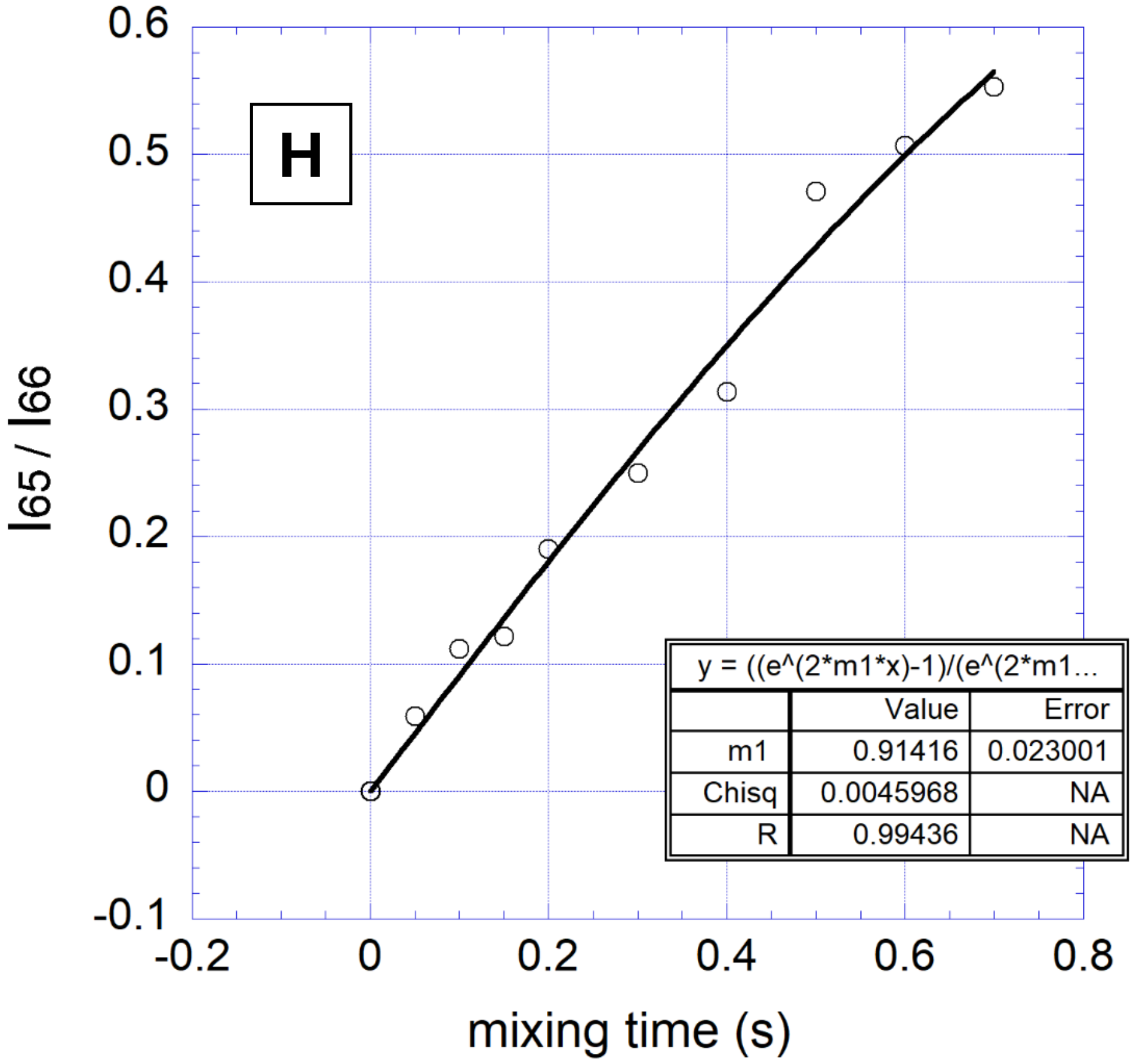
H
I_{65} / I_{66}
mixing time (s)
y = ((e^(2*m1*x)-1)/(e^(2*m1...
Value Error
m1 0.91416 0.023001
Chisq 0.0045968 NA
R 0.99436 NA

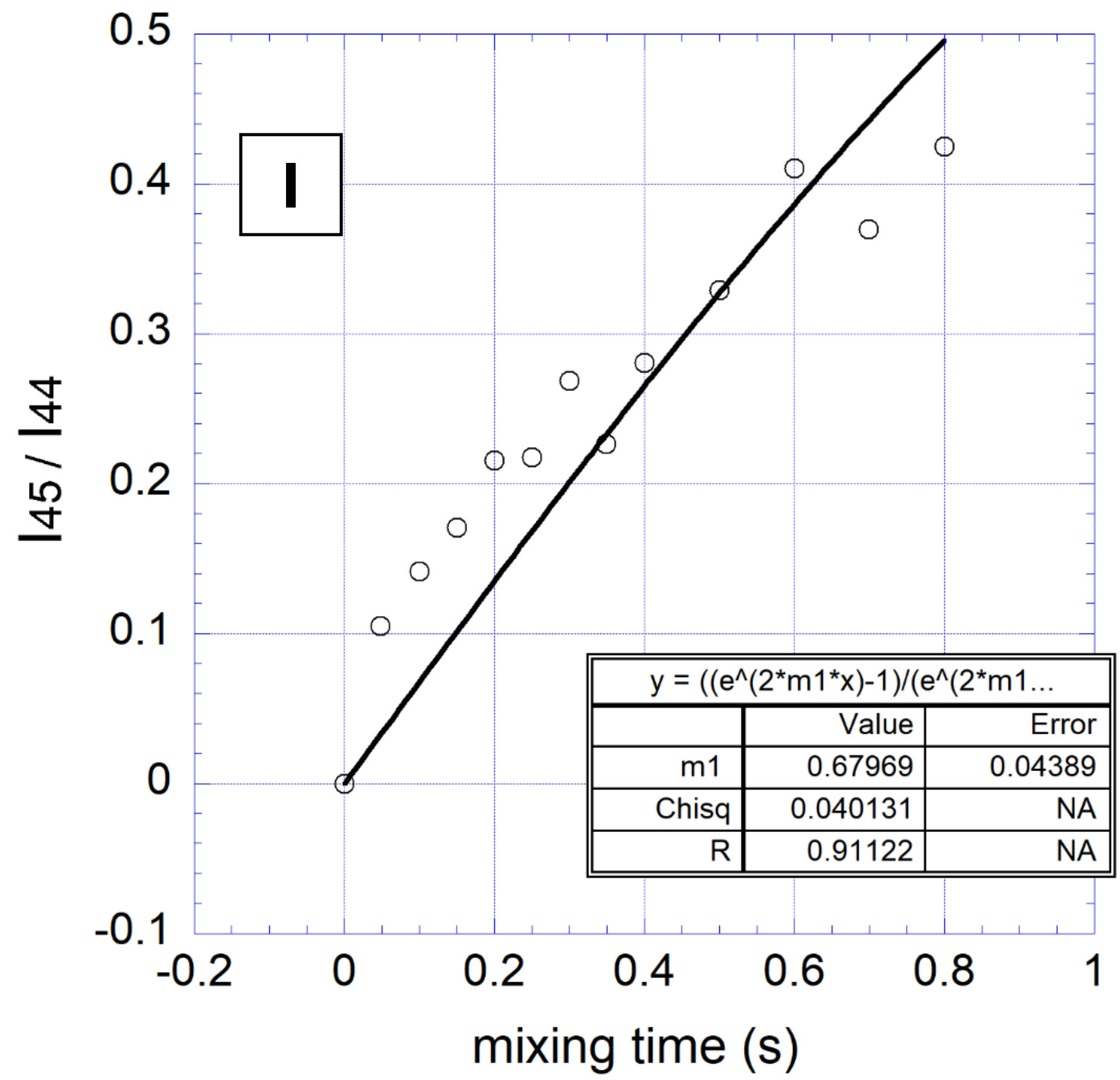
I
y = ((e^(2*m1*x)-1)/(e^(2*m1...
Value
Error
m1
0.67969
0.04389
Chisq
0.040131
NA
R
0.91122
NA
I45 / I44
mixing time (s)

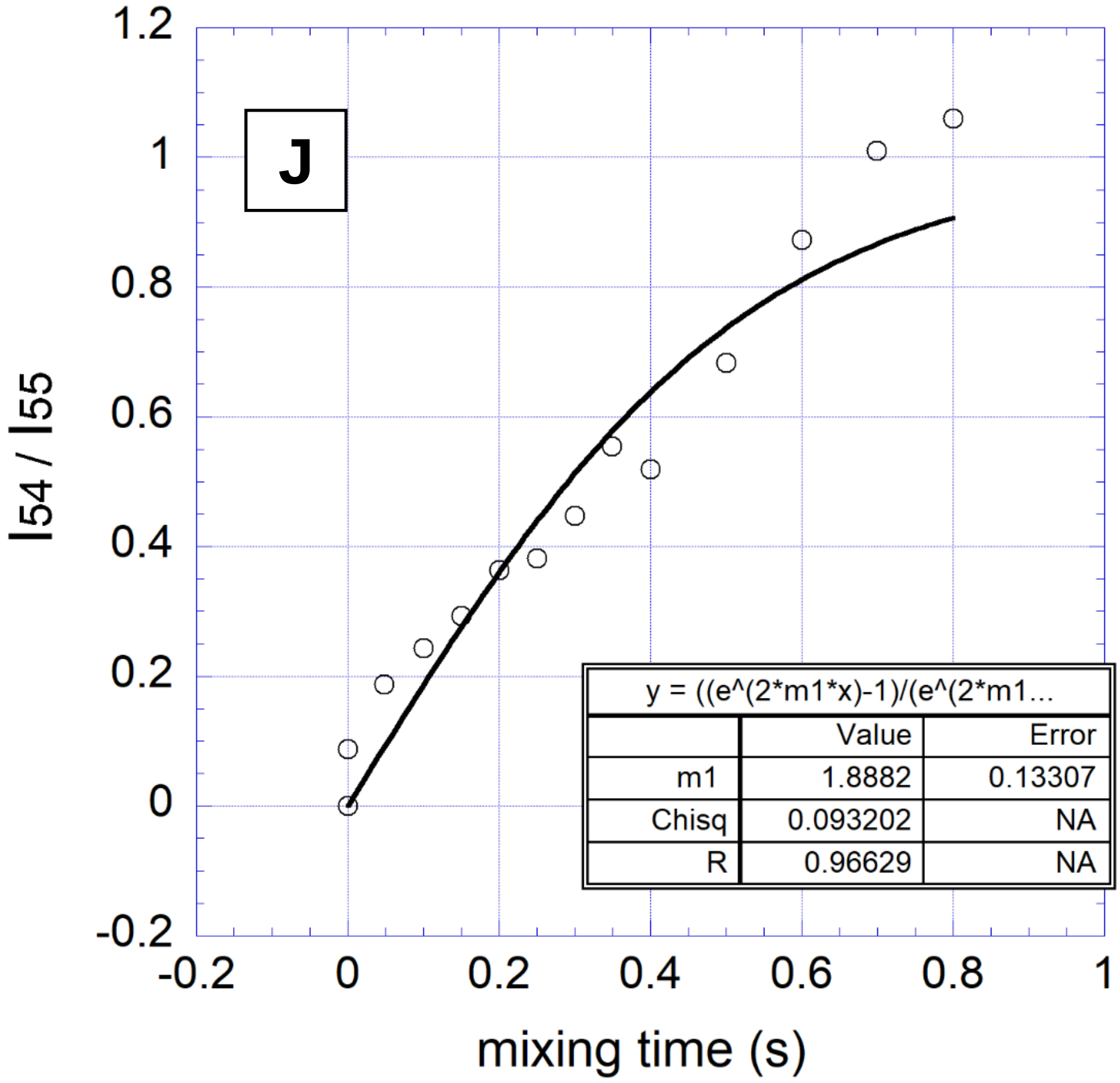
J
y = ((e^(2*m1*x)-1)/(e^(2*m1...
Value
Error
m1
1.8882
0.13307
Chisq
0.093202
NA
R
0.96629
NA
I54 / I55
mixing time (s)

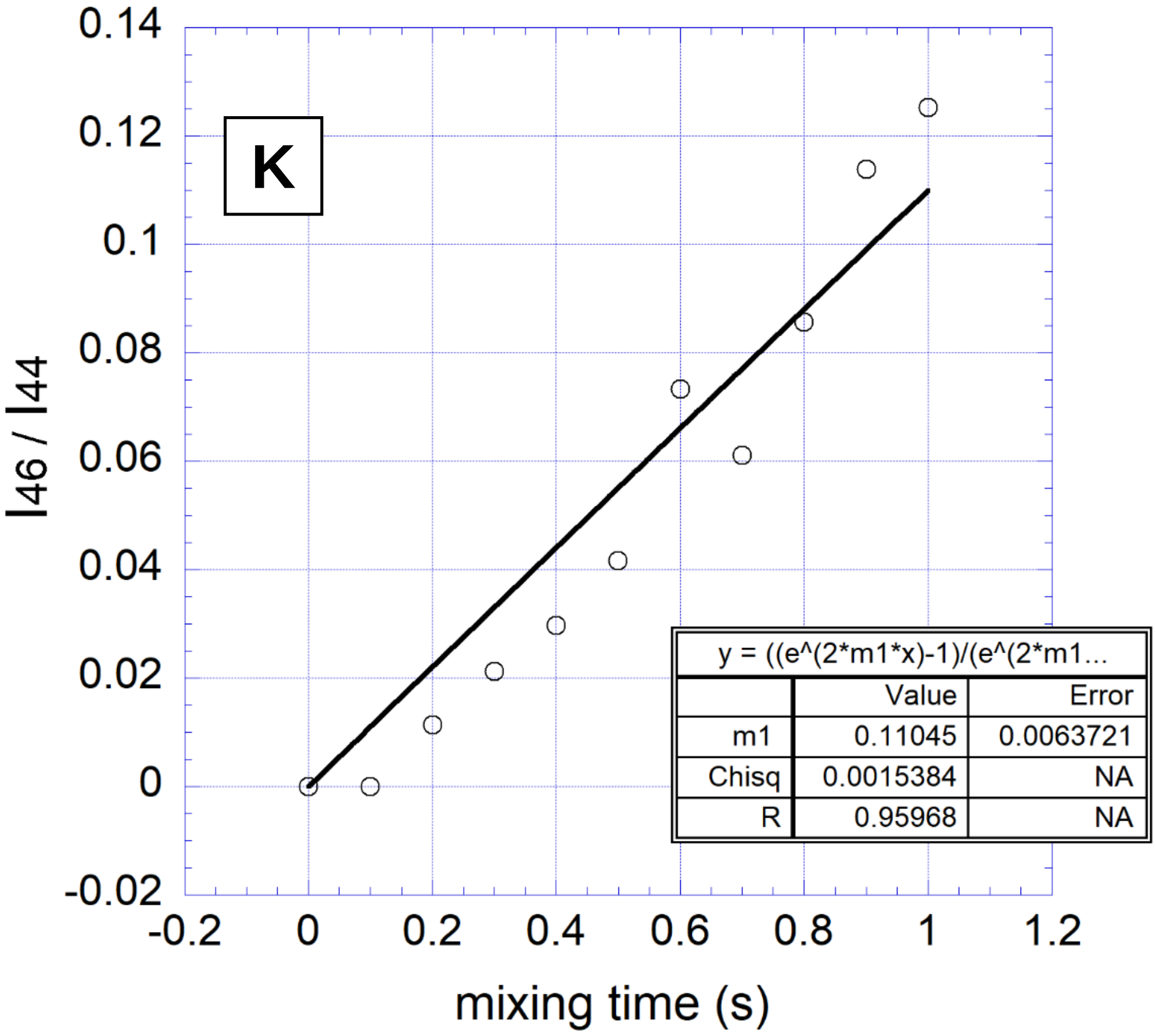
K
0.14
0.12
0.1
0.08
0.06
0.04
0.02
0
-0.02
-0.2
0
0.2
0.4
0.6
0.8
1
1.2
I46 / I44
mixing time (s)
y = ((e^(2*m1*x)-1)/(e^(2*m1...
Value
Error
m1
0.11045
0.0063721
Chisq
0.0015384
NA
R
0.95968
NA

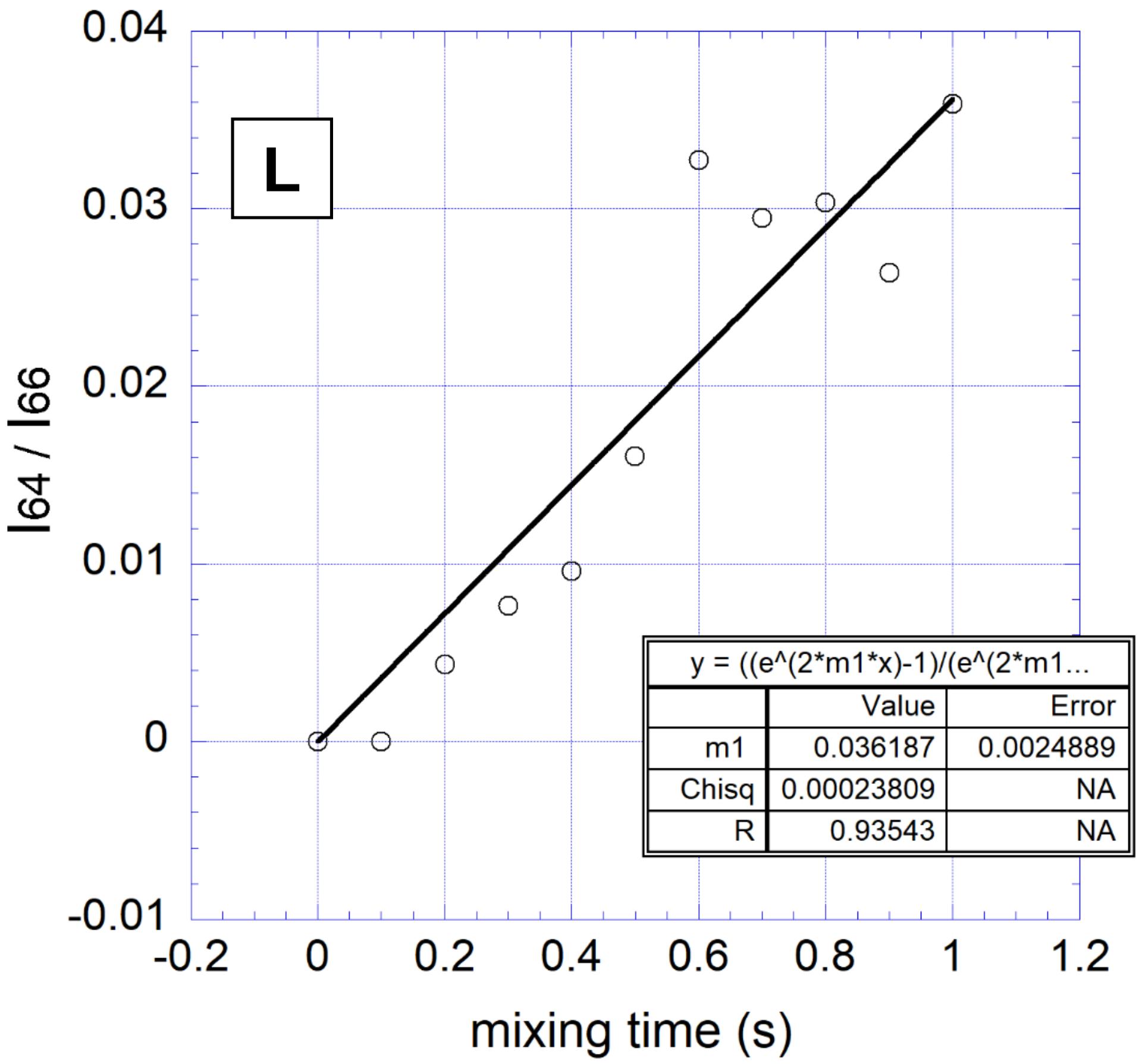
L
0.04
0.03
0.02
0.01
0
-0.01
-0.2
0
0.2
0.4
0.6
0.8
1
1.2
I64 / I66
mixing time (s)
y = ((e^(2*m1*x)-1)/(e^(2*m1...
Value
Error
m1
0.036187
0.0024889
Chisq
0.00023809
NA
R
0.93543
NA

	Normalization	Fitted to:	
Protein	$\frac{2 * I_{ij}(\tau_{mix})}{I_{ii}(\tau_{mix}) + I_{jj}(\tau_{mix})}$	$I_{NOE}(\tau_{mix}) = \frac{e^{2x} - 1}{e^{2x} + 1}$	
**5,6**-F2-Trp CypA	$\sigma_{5\to6}$= 1.013 ± 0.014		**M**
	$\sigma_{6\to5}$= 0.954 ± 0.021		**N**
**4,5**-F2-Trp CypA	$\sigma_{4\to5}$= 1.001 ± 0.057		**O**
	$\sigma_{5\to4}$= 0.996 ± 0.057		**P**
**4,6**-F2-Trp CypA	$\sigma_{4\to6}$= 0.0566 ± 0.0022		**Q**
	$\sigma_{6\to4}$= 0.0536 ± 0.0038		**R**

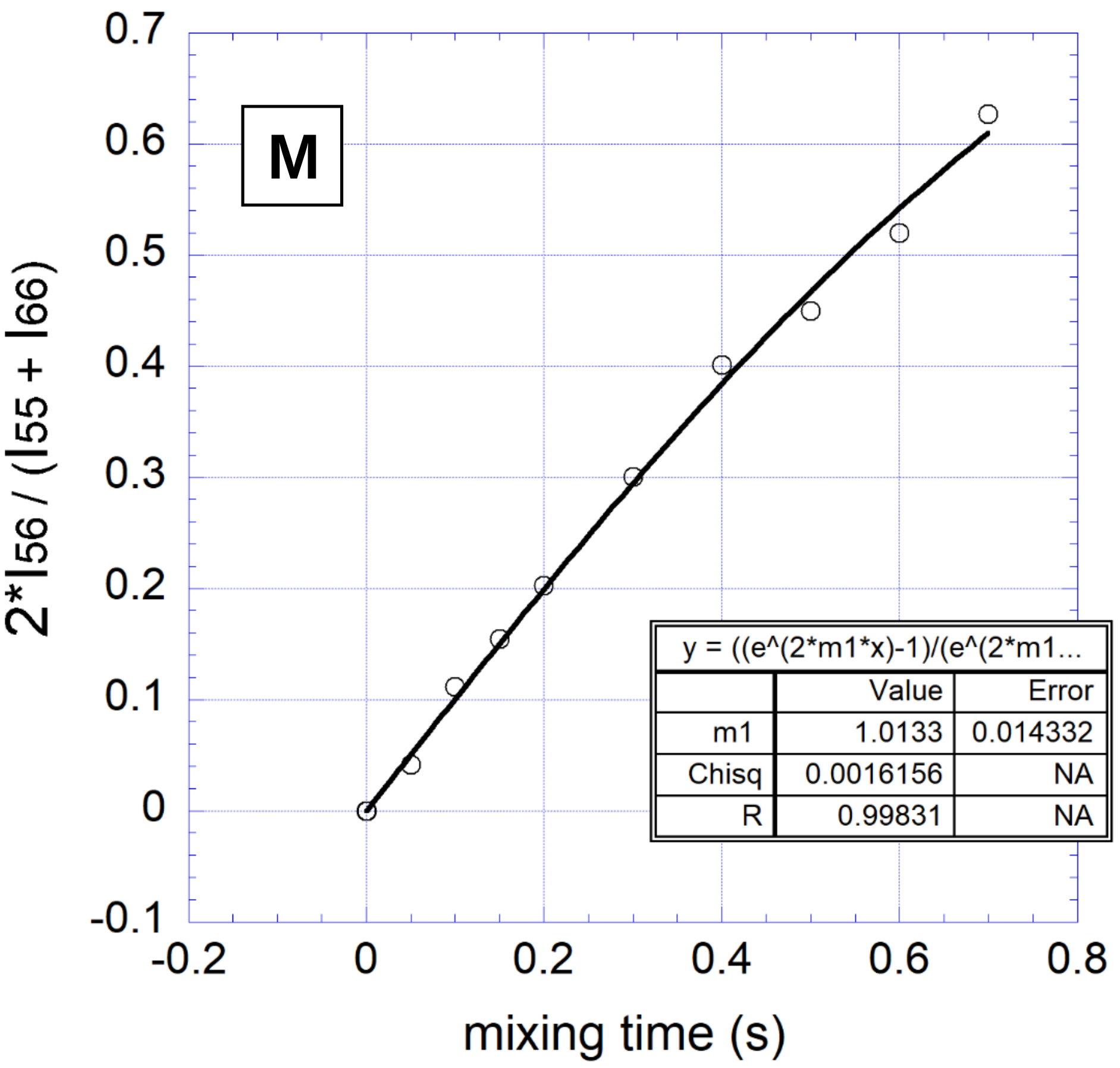

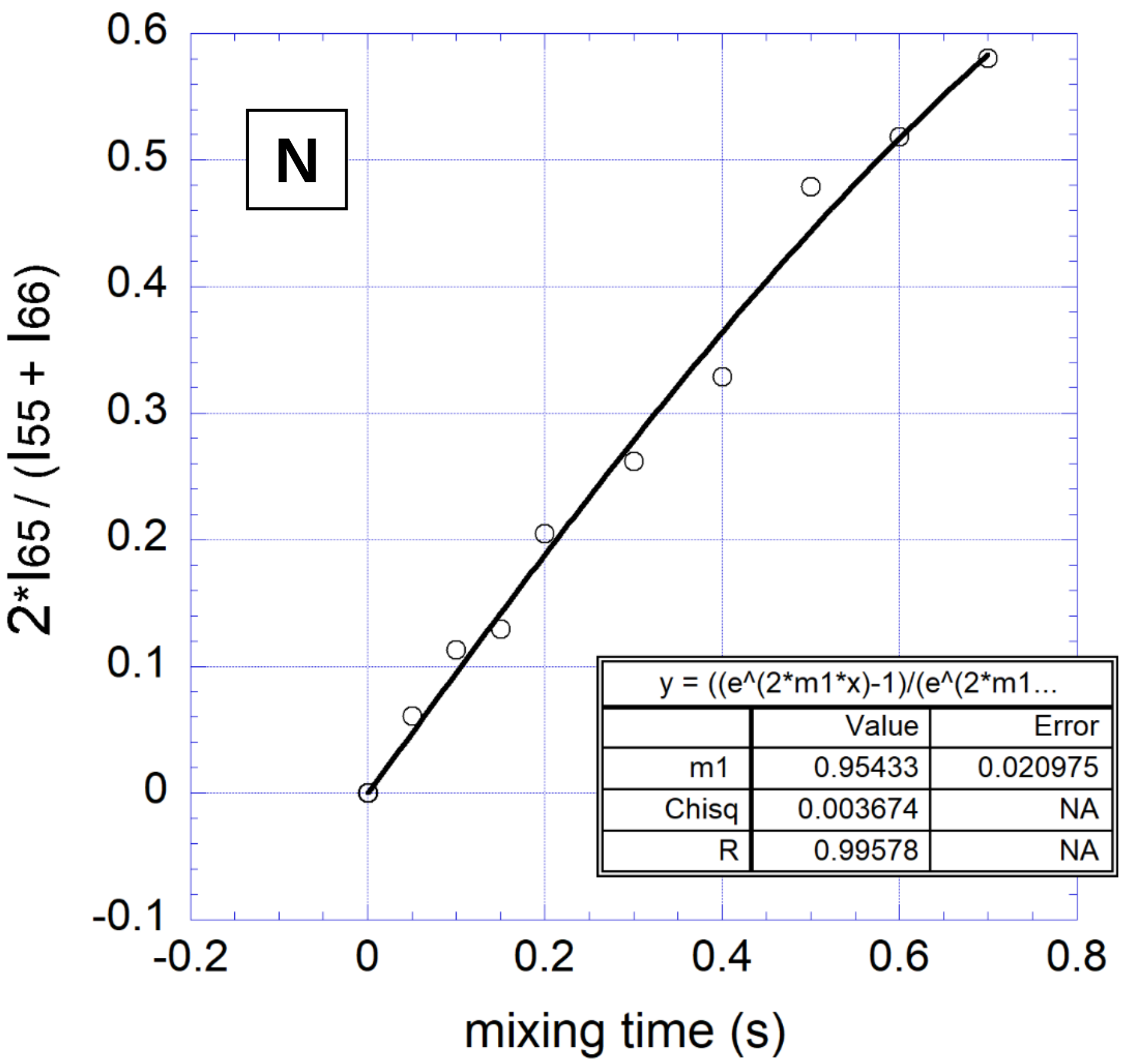
N
2*I65 / (I55 + I66)
mixing time (s)
y = ((e^(2*m1*x)-1)/(e^(2*m1...
Value Error
m1 0.95433 0.020975
Chisq 0.003674 NA
R 0.99578 NA

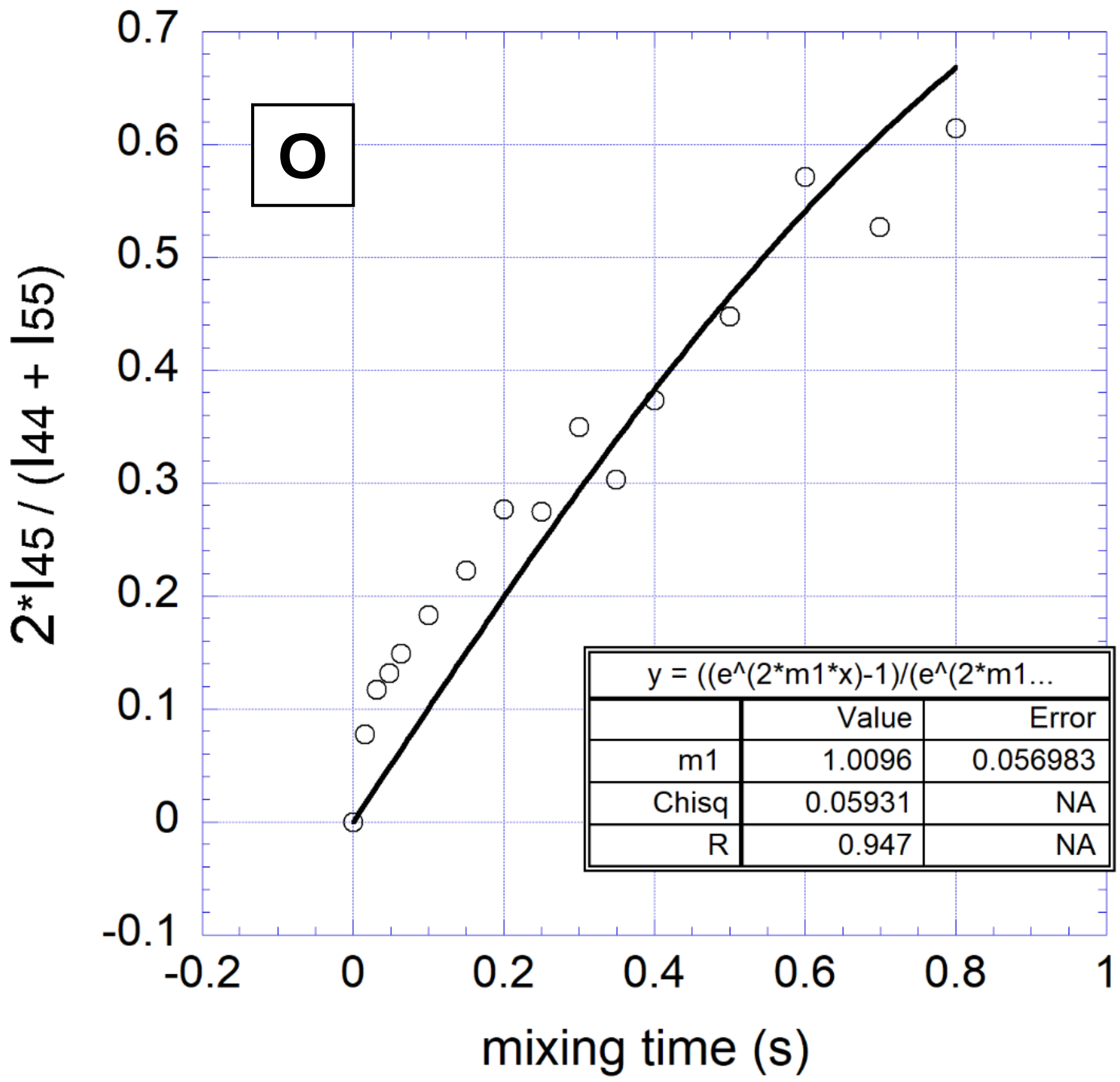
O
2*I45 / (I44 + I55)
mixing time (s)
y = ((e^(2*m1*x)-1)/(e^(2*m1...
Value Error
m1 1.0096 0.056983
Chisq 0.05931 NA
R 0.947 NA

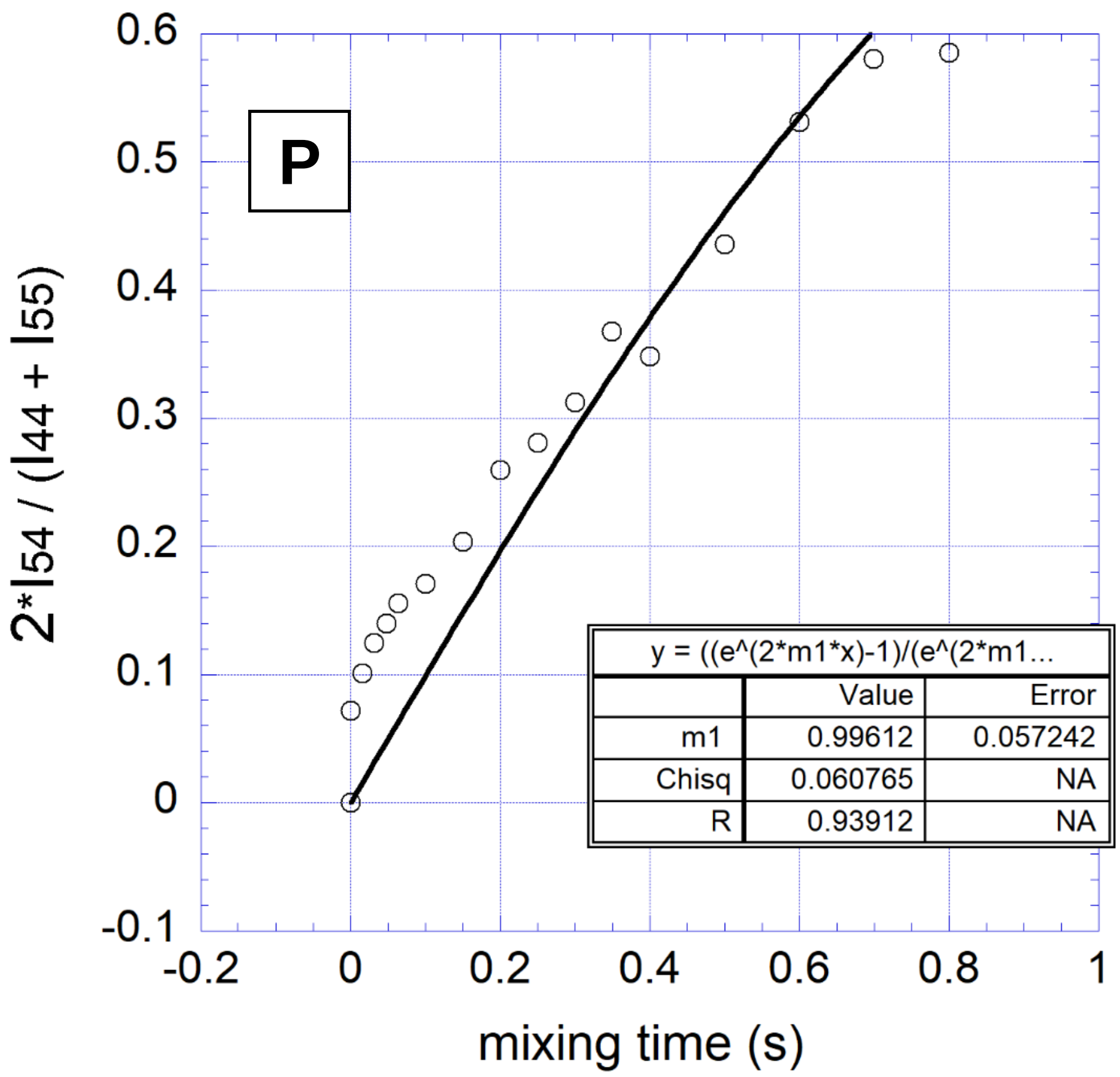
P
2*I54 / (I44 + I55)
mixing time (s)
y = ((e^(2*m1*x)-1)/(e^(2*m1...
Value Error
m1 0.99612 0.057242
Chisq 0.060765 NA
R 0.93912 NA
0.6
0.5
0.4
0.3
0.2
0.1
0
-0.1
-0.2
0
0.2
0.4
0.6
0.8
1

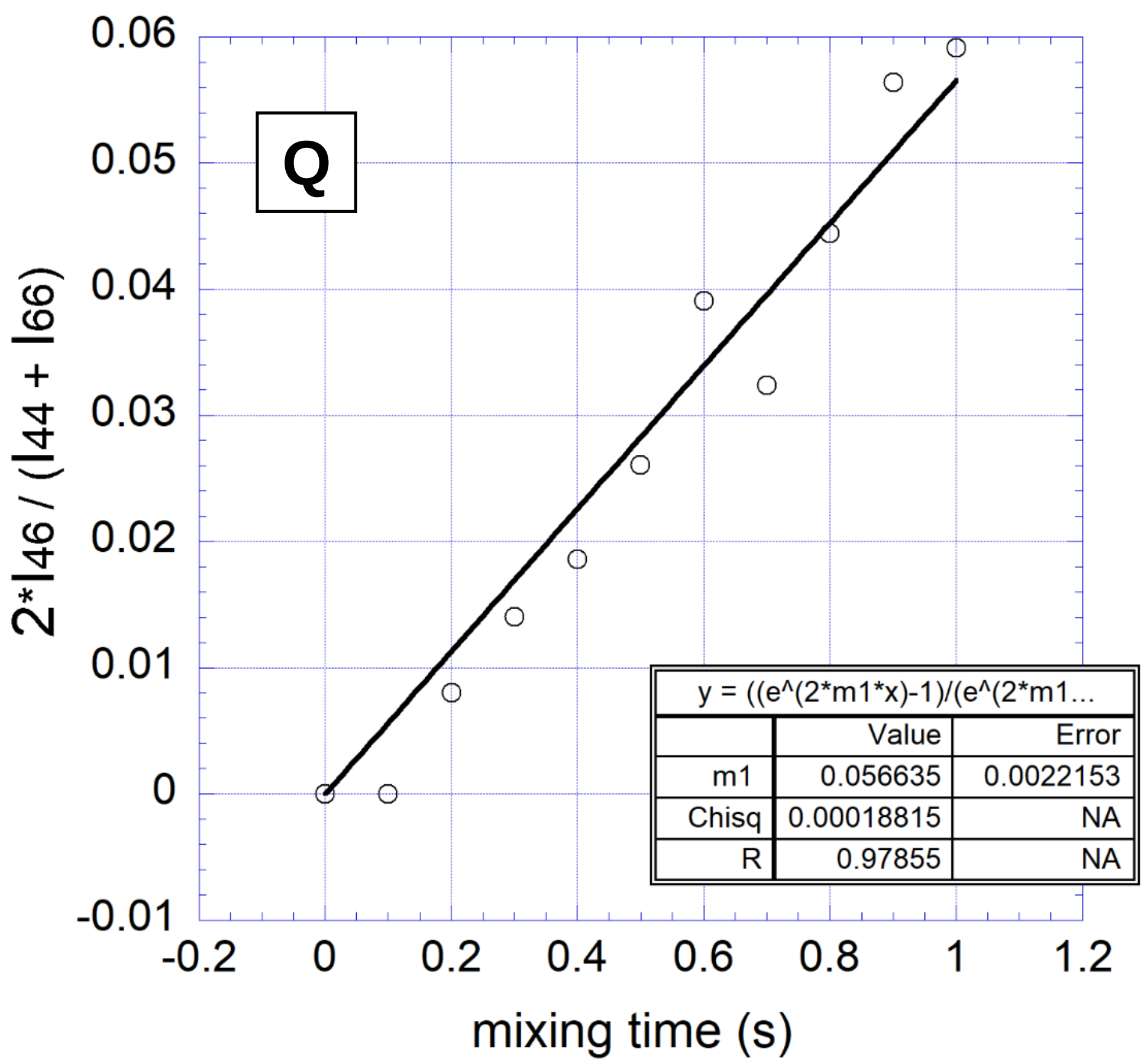
Q
2*I46 / (I44 + I66)
mixing time (s)
y = ((e^(2*m1*x)-1)/(e^(2*m1...
Value Error
m1 0.056635 0.0022153
Chisq 0.00018815 NA
R 0.97855 NA
0.06
0.05
0.04
0.03
0.02
0.01
0
-0.01
-0.2
0
0.2
0.4
0.6
0.8
1
1.2

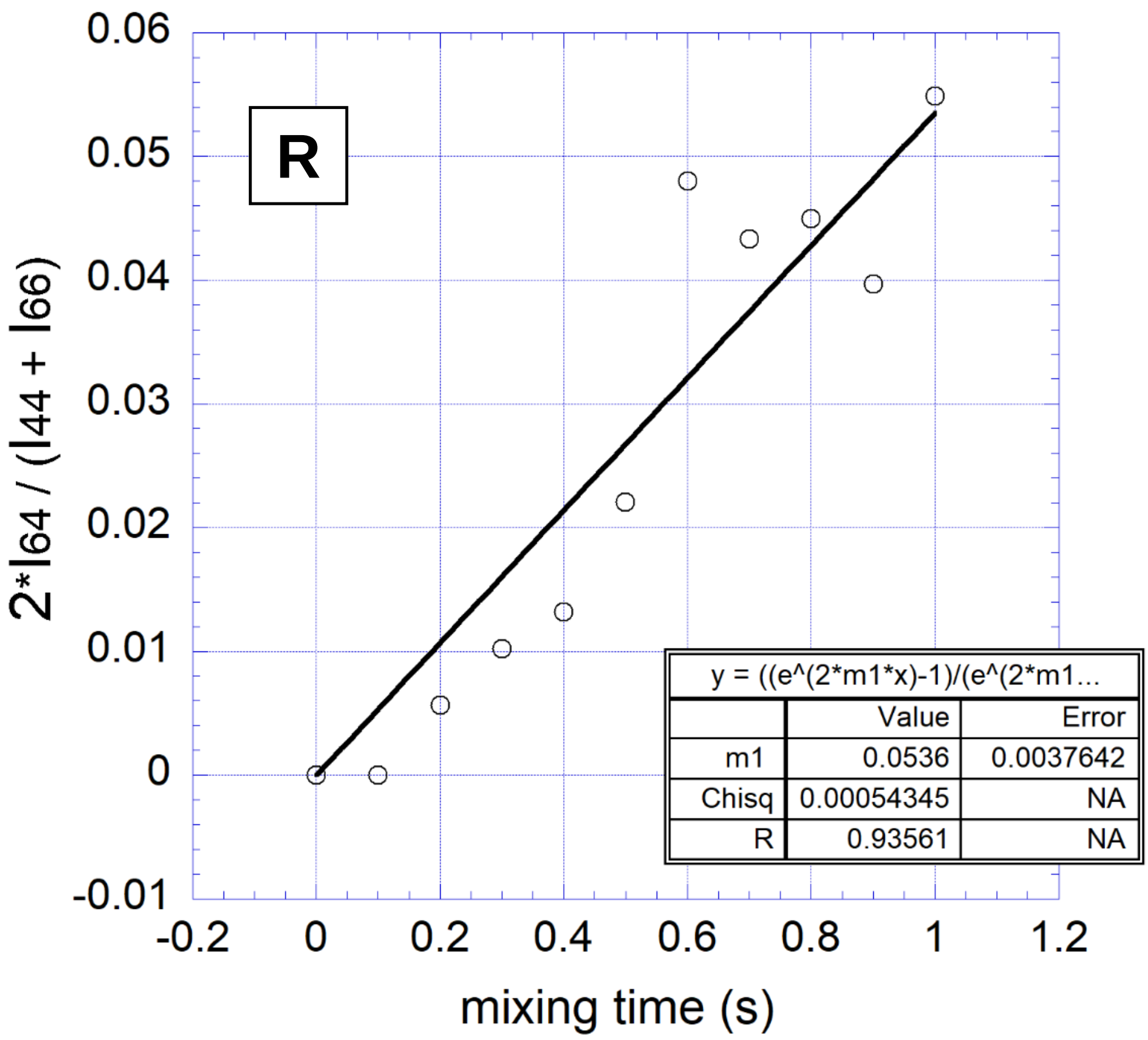

In an alternative approach, cross-peak intensities were averaged and normalized to the averaged initial diagonal intensity for each mixing time,

$$\frac{\left(I_{ij}\,(\tau_{mix}) + I_{ji}\,(\tau_{mix})\right)/2}{\left(I_{ii}(\tau_{mix} = 0.001\ \mathrm{s})\ +\ I_{jj}(\tau_{mix} = 0.001\ \mathrm{s})\right)/2}$$

and the resulting initial-diagonal-normalized cross peak intensities were plotted against mixing time and fitted to equation A:[2]

$$I_{NOE}(\tau_{mix}) = \frac{Cross - peak\ intensity}{M_0} = \frac{1}{2}e^{-R_L\tau_{mix}}(1 - e^{-2R_c\tau_{mix}})$$

$$= \left(\frac{1}{2e^{R_L\tau_{mix}}}\right)\left(1 - \frac{1}{e^{2R_c\tau_{mix}}}\right)$$

Where:

R_c= Initial NOE build-up rate.

R_L= Leakage relaxation rate.

τ_{mix} = Mixing time.

M_0= Initial total magnetization (diagonal-peak intensity)

After substituting R_L by m_2 and R_c by m_1 in the previous expression, we obtain:

$$I_{NOE}(\tau_{mix}) = \left(\frac{1}{2e^{m_2\tau_{mix}}}\right)\left(1 - \frac{1}{2e^{m_1\tau_{mix}}}\right)$$

When $m_1 = m_2 = 1$, $\tau_{mix} = x$

$$I_{NOE}(\tau_{mix}) = \left(\frac{1}{2e^{x}}\right)\left(1 - \frac{1}{2e^{x}}\right) = \frac{1 - e^{-2x}}{2e^{x}} \qquad (A)$$

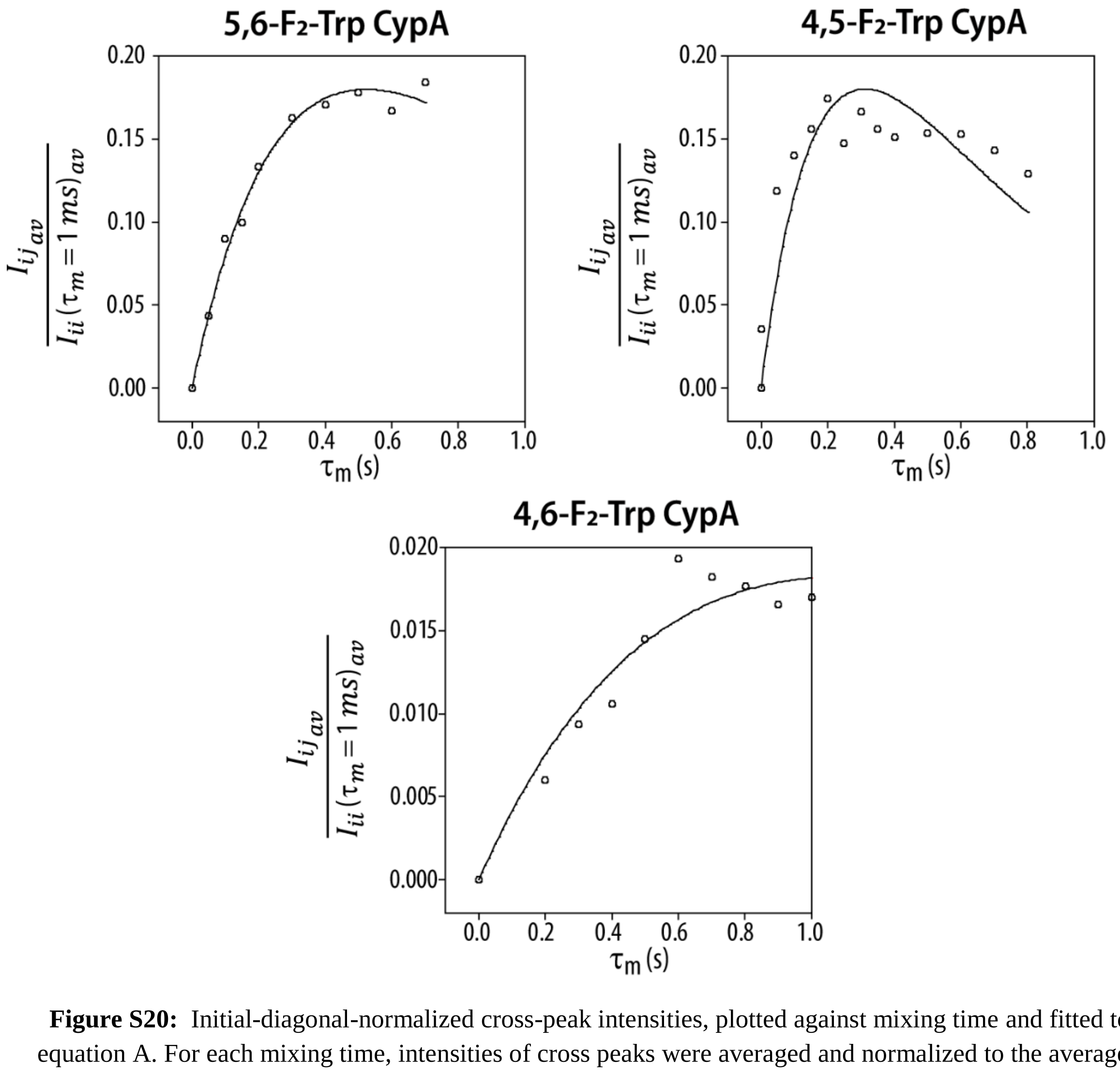

Figure S20: Initial-diagonal-normalized cross-peak intensities, plotted against mixing time and fitted to equation A. For each mixing time, intensities of cross peaks were averaged and normalized to the averaged initial diagonal intensity $\left((I_{ij} + I_{ji})/2\right)/\left((I_{ii}(\tau_{mix} = 0.001\text{ s}) + I_{jj}(\tau_{mix} = 0.001\text{ s}))/2\right)$.

The averaged cross-relaxation rates extracted from these plots, as well as the measured F–F distances are provided in the table below:

	Normalization	Fitted to:	r_{ij}^{eff}
Protein	$\frac{\left(I_{ij}\,(\tau_{mix}) + I_{ji}\,(\tau_{mix})\right)/2}{\left(I_{ii}(\tau_{mix} = 0.001\text{ s}) + I_{jj}(\tau_{mix} = 0.001\text{ s})\right)/2}$	$I_{NOE}(\tau_{mix}) = \frac{1 - e^{-2x}}{2e^{x}}$	$= \left(44.72\frac{8.2}{\sigma_{ij}/s^{-1}}\right)^{\frac{1}{6}}$
**5,6**-F_2-Trp CypA	0.979 ± 0.044		**2.68**
**4,5**-F_2-Trp CypA	1.642 ± 0.198		**2.46**
**4,6**-F_2-Trp CypA	0.0451 ± 0.0059		**4.48**

Using this additional normalization and more complex fitting procedure did not result in a significant improvement when comparing the NMR-derived distances to the X-ray structure distances and thus was not considered further.

14. References

[32] L. Villaescusa, I. Hernández, L. Azcune, et al., “Rigidified Bis(sulfonyl)ethylenes as Effective Michael Acceptors for Asymmetric Catalysis: Application to the Enantioselective Synthesis of Quaternary Hydantoins” *The Journal of Organic Chemistry* **88**, (2023): 972–987, https://doi.org/10.1021/acs.joc.2c02403.

[33] T. Pillaiyar, E. Gorska, G. Schnakenburg, C. E. Müller, “General Synthesis of Unsymmetrical 3,3′-(Aza)diindolylmethane Derivatives” *The Journal of Organic Chemistry* **83**, (2018): 9902–9913, https://doi.org/10.1021/acs.joc.8b01349.

[34] I. V. Klimovich, L. I. Leshanskaya, S. I. Troyanov, et al., “Design of indigo derivatives as environment-friendly organic semiconductors for sustainable organic electronics” *Journal of Materials Chemistry C* **2**, (2014): 7621–7631, https://doi.org/10.1039/C4TC00550C.

[35] A. Rammohan, A. P. Krinochkin, Yu. M. Sayfutdinova, et al., “Reaction of 6*H*-5-(4-Fluorophenyl)-3-(2-pyridyl)-1,2,4-triazine with 4,5-Difluoro-1,2-dehydrobenzene” *Russian Journal of General Chemistry* **94**, (2024): 2255–2263, https://doi.org/10.1134/S1070363224090056.

[36] C. Zhou, M. Huang, Y. Yao, et al., “Transition-metal-free and additive-free intermolecular hydroarylation of alkenes with indoles in hexafluoroisopropanol” *Organic & Biomolecular Chemistry* **21**, (2023): 9534–9541, https://doi.org/10.1039/D3OB01570J.

[37] P. C. Knipe, M. Gredičak, A. Cernijenko, R. S. Paton, M. D. Smith, “Phase-Transfer-Catalysed Synthesis of Pyrroloindolines and Pyridoindolines by a Hydrogen-Bond-Assisted Isocyanide Cyclization Cascade” *Chemistry – A European Journal* **20**, (2014): 3005–3009, https://doi.org/10.1002/chem.201400192.

[38] O. V. Dolomanov, L. J. Bourhis, R. J. Gildea, J. A. K. Howard, H. Puschmann, “*OLEX2*: a complete structure solution, refinement and analysis program” *Journal of Applied Crystallography* **42**, (2009): 339–341, https://doi.org/10.1107/S0021889808042726.

[39] G. M. Sheldrick, “*SHELXT* – Integrated space-group and crystal-structure determination” *Acta Crystallographica Section A* **71**, (2015): 3–8, https://doi.org/10.1107/S2053273314026370.

[40] G. M. Sheldrick, “Crystal structure refinement with *SHELXL*” *Acta Crystallographica Section C* **71**, (2015): 3–8, https://doi.org/10.1107/S2053229614024218.

[41] Y. Toyama, K. Takeuchi, I. Shimada, “Evaluating the effect of ^{1}H decoupling on ^{19}F longitudinal relaxation and signal line shape in 5-Fluorotryptophan” *Journal of Magnetic Resonance* **377**, (2025): 107899, https://doi.org/10.1016/j.jmr.2025.107899.
